# Supplementary material for: Differential dynamics of the mammalian mRNA and protein expression response to misfolding stress
Source: Mol Syst Biol. 2016 Jan 20;12(1):855. doi: 10.15252/msb.20156423 (PMC4731011; doi:10.15252/msb.20156423)
Supplement: Supplementary file 1 — Appendix [file MSB-12-855-s001.pdf]

Appendix

Differential dynamics of mammalian mRNA and protein expression in response to misfolding stress

Zhe Cheng*[,1], Guoshou Teo *[,2], Sabrina Krueger[3], Tara Rock[1], Hiromi W.L. Koh[2], Hyungwon Choi[$,2], Christine Vogel[$,1, #]

*,[$] equally contributing authors
[1] Center for Genomics and Systems Biology, New York University, New York, USA
[2] Saw Swee Hock School of Public Health, National University Singapore and National University Health System, Singapore
[3] Max-Delbruck-Center, Berlin, Germany

Table of Contents

Experimental conditions and cellular health 3
Appendix Figure S1. Experimental design 3
Appendix Figure S2. The half-life of DTT in cell culture medium is approximately 4 hours ... 4
Appendix Figure S3. The majority of the cell population continues to divide despite severe ER stress 5
Validation of quantitative mRNA and protein measurements 7
Appendix Figure S4. Principle Component Analysis classifies the early, intermediate and late phases 7
Appendix Figure S5. Clusters of similar gene expression show functional enrichment related to the stress response 8
Appendix Figure S6. Transcriptome measurements are highly reproducible 9
Appendix Figure S7. Transcriptome measurements are accurate 10
Appendix Figure S8. Combining sub-cellular fractions to increase proteomics coverage 11
Appendix Figure S9. Heatmap of entire protein expression data shows consistent expression patterns of genes with similar functions 12
Appendix Figure S10. Proteomics measurements are consistent across replicates 13
Appendix Figure S11. Proteomics provides appropriate estimates of protein concentrations 14
Appendix Figure S12. Housekeeping genes are comparatively constant in their protein expression 16
Analysis of the integrated mRNA and protein expression data 17
Appendix Figure S13. mRNA and protein concentrations correlate partially across the time course 17
Appendix Figure S14. Complete function annotation shows distinct expression patterns for mRNA and protein 19
Appendix Table S1. Fold-changes and dynamic range of the expression data 20
Appendix Figure S15. Changes between consecutive gene expression mRNA and protein measurements are maximal around 8 hours 21
Appendix Figure S16. Some genes show discordance between mRNA- and protein-level regulation 22
Appendix Figure S17. Splice variants of aminoacyl-tRNA synthetases show some evidence for differential protein expression 25
Appendix Figure S18. The three peptide variants of QARS show different expression patterns 27
Appendix Figure S19. PECA analysis of the extended dataset 29
Appendix Figure S20. Expression changes across entire transcriptome confirm trends for the core dataset 31
Appendix Figure S21. PECA results for entire RNA data confirm the trends for the core dataset 32
Mammalian sequence features and other characteristics of expression regulation 33
Appendix Table S2. Sequence and other features involved in protein regulation 33
PECA analysis of dendritic cells responding to LPS 35
Appendix Figure S22. Heatmap of expression values and PECA output of dendritic cells responding to LPS treatment 35
Appendix Table S3. Significance cutoffs - Jovanovic et al. (Science 2015) 37
Appendix Table S4. Significantly regulated genes identified in data published by Jovanovic et al. (Science 2015) 38
Appendix Figure 23. The LPS response shows switch-like regulation at the RNA level 39
References 40

Experimental conditions and cellular health

The following assays were performed to characterize the experimental conditions and the overall state of the cells under treatment. The experiment was conducted in a triplicate, and two replicates of higher data quality were selected for further analysis.

Appendix Figure S1. Experimental design

We designed the experiment to account for changes in cellular growth over the 30h time period. We grew cells to 70% confluency before treating the first sample for the "30h treatment period". After another six hours, the "24h treatment" was started in a different plate, followed by the other treatments. At the end, all samples were collected simultaneously at "0 hours". The treatment periods were: 0, 0.5, 1, 2, 8, 16, 24, and 30 hours.

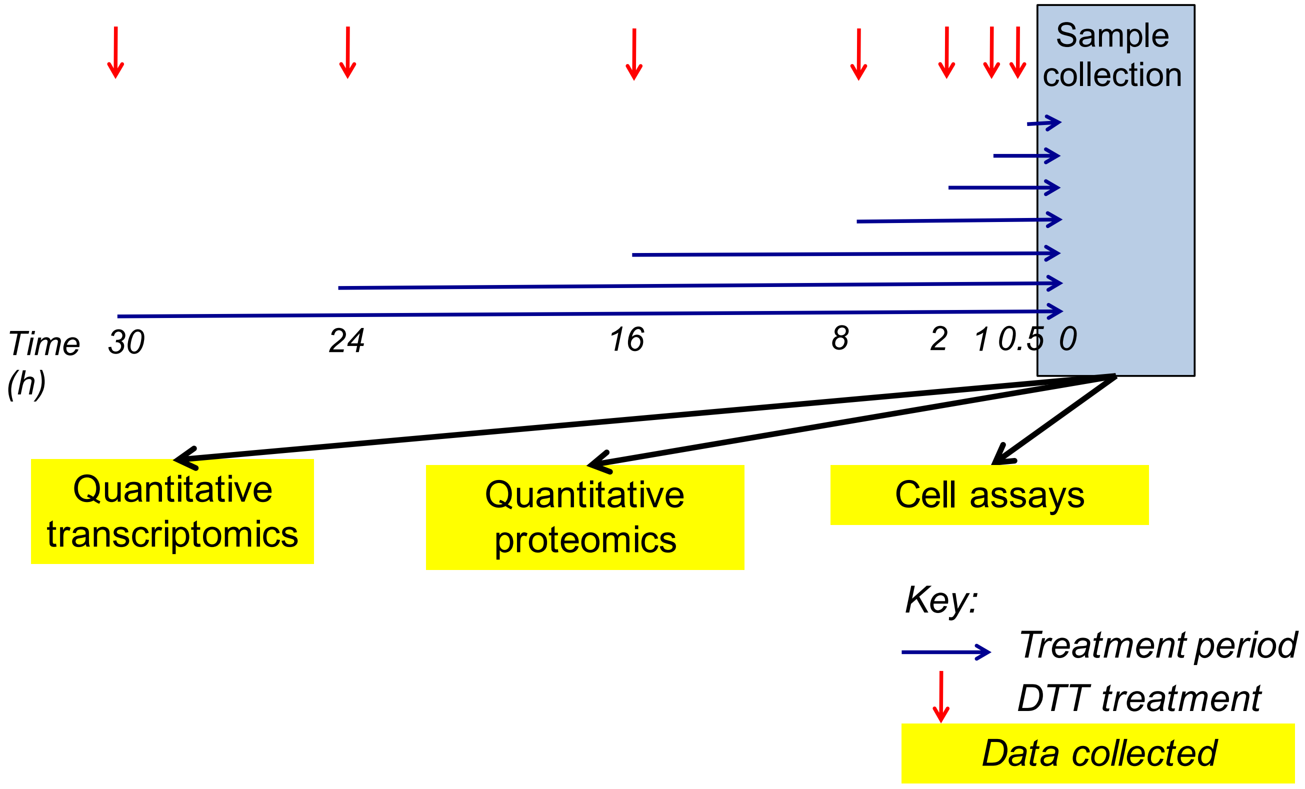

Appendix Figure S2. The half-life of DTT in cell culture medium is approximately 4 hours
We used the small molecule redox reagent dithiothreitol (DTT) to disturb protein folding and induce reductive stress to the cells. DTT reduces disulfide bonds of proteins, and the accumulation of unfolded or misfolded proteins in the lumen of endoplasmic reticulum (ER) causes ER stress and the unfolded protein response (UPR). To define how long the stress lasted in the experiment, we measured the degradation of DTT in the medium. The assay used the fact that DTT reacts with 5,5'-dithiobis-2-nitrogenzoic acid (DTNB) to form 2-nitro-5-mercaptobenzoic acid, which can be monitored spectrophotometrically at 415nm. To quantify DTT concentration, we collected the cell culture medium containing DTT for each time point. We see that the DTT degrades over time, and its half-life is approximately four hours. Shown are the averages and standard deviations across quadruplicate experiments.

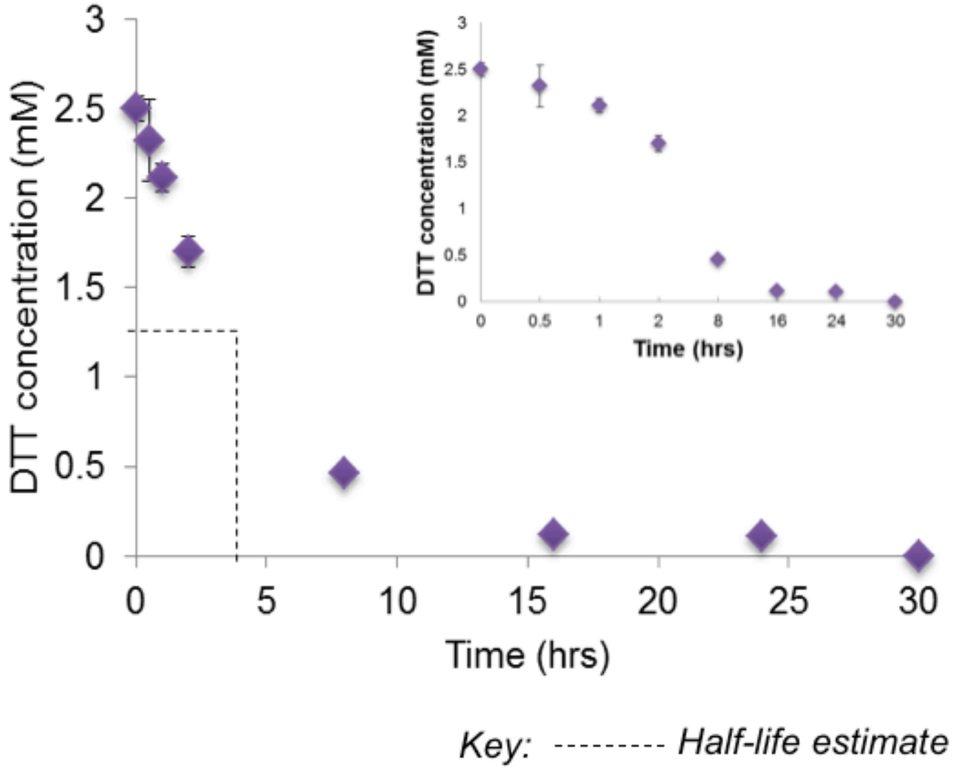

Appendix Figure S3. The majority of the cell population continues to divide despite severe ER stress

The figure displays the complete results for cell assays. The DNA content (A) and active mitosis (B) experiments were conducted using flow cytometry sorting of propidium iodide labeled cells and the immunofluorescence staining of mitotic nuclei in red with anti-phospho-Histone H3 antibody, respectively.

A. To test whether cells are arrested or dead, we fixed the cells with 70% ethanol after stress and quantified DNA content in samples using nucleic acid stain propidium iodide (PI) followed by flow cytometry analysis. As can be seen across all time points, most cells were in G1 stage (2N), some cells were undergoing DNA synthesis (2N-4N), and we also found final mitotic populations (4N). There were apoptotic cells (<2N) arising in the samples after one hour. The proportion of apoptotic cells peaked at two hours at 45% of the observed cell population. The figure shows the percentages of apoptotic *versus* non-apoptotic cells.

B. Although S phase cells were clearly observed in assay (A), the 4N peak identifying mitotic populations is small in the 2h, 8h, and 16h time points. To validate that the cells actively divide during the stress treatment, we tested for the G2/M checkpoint and into M phase using the mitosis marker anti-Phospho-Histone H3 (Ser 10) antibody. Phosphorylation at Ser10 of histone H3 is tightly correlated with chromosome condensation during M phase. The immunofluorescence pictures (B) show M phase nuclei in red (anti-phospho-Histone H3) and nuclei in blue (DAPI). When calculating the ratio between M phase/all nuclei, we find no significant difference in the percentage of cells undergoing mitosis between stressed and control groups. The percentages of mitotic nuclei are 6.2% for 0h, 5.9% for 0.5h, 5.6% for 1h, 5.4% for 2h, 4.8% for 8h, 4.7% for 16h, 4.8% for 24h, and 5.0% for 30h. We observe more apoptotic nuclei debris stained in blue.

A.

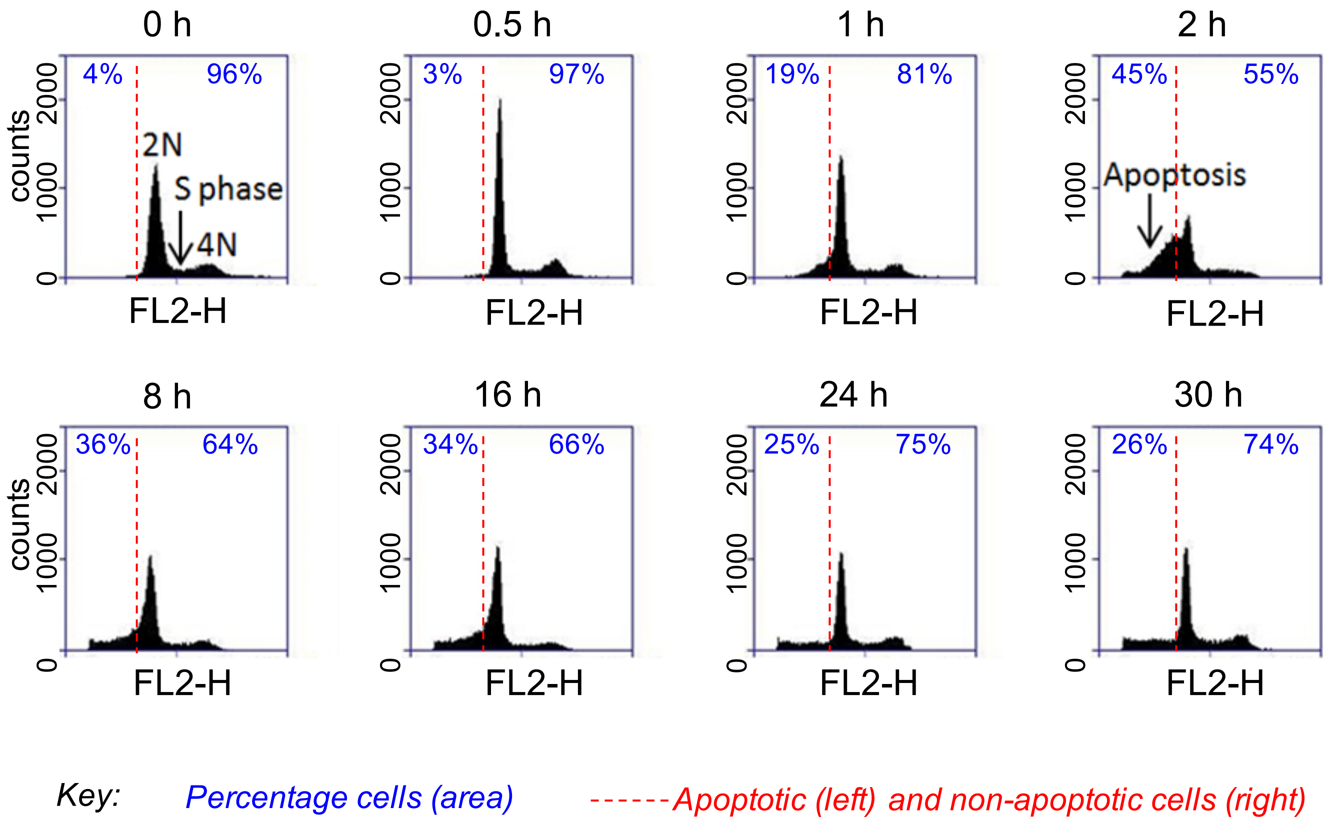

0 h
4%
96%
counts
0
1000
2000
2N
S phase
4N
FL2-H
0.5 h
3%
97%
FL2-H
1 h
19%
81%
FL2-H
2 h
45%
55%
Apoptosis
FL2-H
8 h
36%
64%
FL2-H
16 h
34%
66%
FL2-H
24 h
25%
75%
FL2-H
30 h
26%
74%
FL2-H
Key:
Percentage cells (area)
Apoptotic (left) and non-apoptotic cells (right)

B.

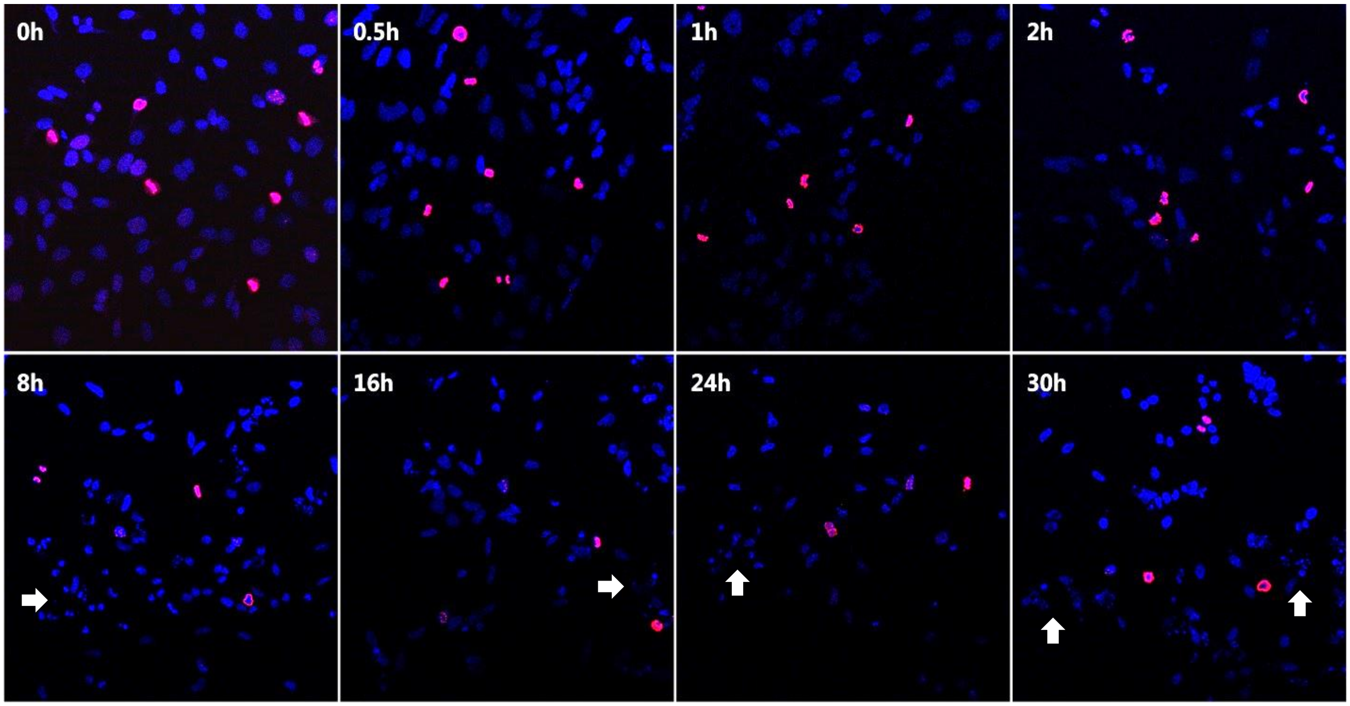

0h
0.5h
1h
2h
8h
16h
24h
30h

Validation of quantitative mRNA and protein measurements

We performed several tests to validate the accuracy of quantitative mRNA and protein measurements. These tests included: Principle Component Analysis, function enrichment analysis, correlation across replicate measurements, and comparison with RNA-seq and western blot data. Based on the phenotypic assays (above) and the time course expression patterns (below), we defined three phases of the dynamic response -- *early* (<2hrs), *intermediate* (2hrs), and *late* (>2hrs) – in which concerted changes happened.

Appendix Figure S4. Principle Component Analysis classifies the early, intermediate and late phases

Principle Component Analysis of the normalized expression values of the RNA (A) and protein (B) data across the time points to describe different phases stress response was performed using the Perseus software tool (http://www.biochem.mpg.de/5111810/perseus). The figures show the first two components show for the RNA (A) and protein (B). While the early and late phase are distinctly different, the intermediate phase (2 to 8 hours) represents a transition period.

A.

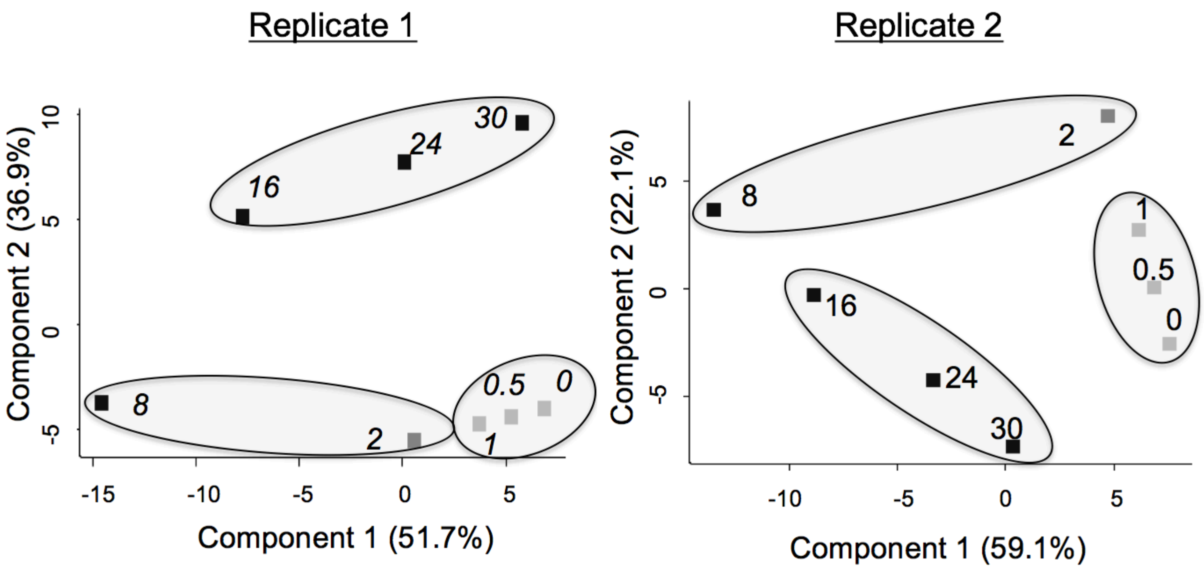

B.

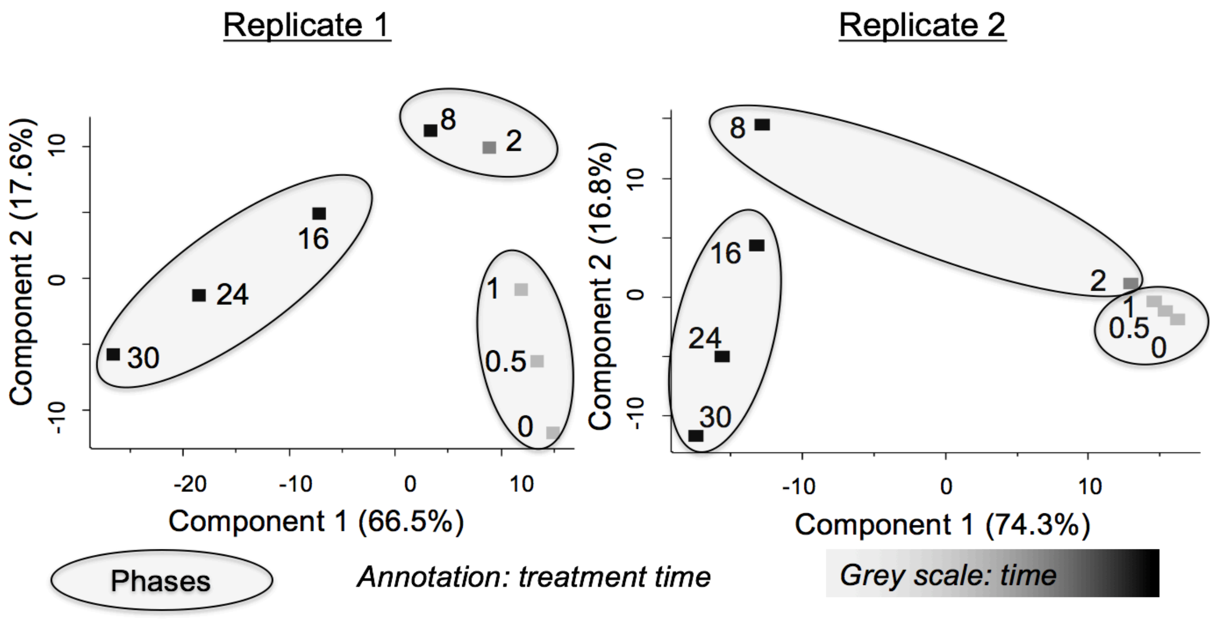

Appendix Figure S5. Clusters of similar gene expression show functional enrichment related to the stress response

We measured mRNA expression changes for 18,983 mRNAs over the eight time points across two replicates, shown as normalized, relative expression data in this figure. The data was divided into 15 clusters with 0.544 as distance threshold, as defined by the Perseus data analysis tool, using hierarchical clustering with an average linkage (see main text). We performed function enrichment analysis using the NCBI DAVID GO term enrichment tool on the six largest clusters (see **Appendix Dataset EV2**)(Huang et al, 2009a; Huang et al, 2009b). Shown are significantly enriched functions, FDR<5%. Genes related to transcription regulation and programed cell death were up-regulated in the early phase after stress (Clusters B and F). During the intermediate phase, genes involved in ER stress and UPR were highly expressed (Cluster E), while at the same time, genes related to translation elongation, RNA splicing and transport, and macromolecular complex assembly were suppressed (Clusters C and D). During the late phase, when the cells were recovering, the genes involved in protein ubiquitination, lysosome, and glycoprotein / transmembrane protein synthesis were highly expressed (Clusters A and D).

The enriched biological functions are consistent with the findings from the cell assays described above (**Appendix Figure S3**). For the majority of the experiment, both apoptosis and stress response occurred simultaneously. A fraction of the cell population died after stress induction, but when DTT degraded after the 8h time point, the cell population started to recover, undergoing unfolded protein response to refold or degrade unfolded or misfolded proteins, and restore homeostasis and cellular functions. Bottom panels E, I, L mark the early, intermediate, and late phase, respectively.

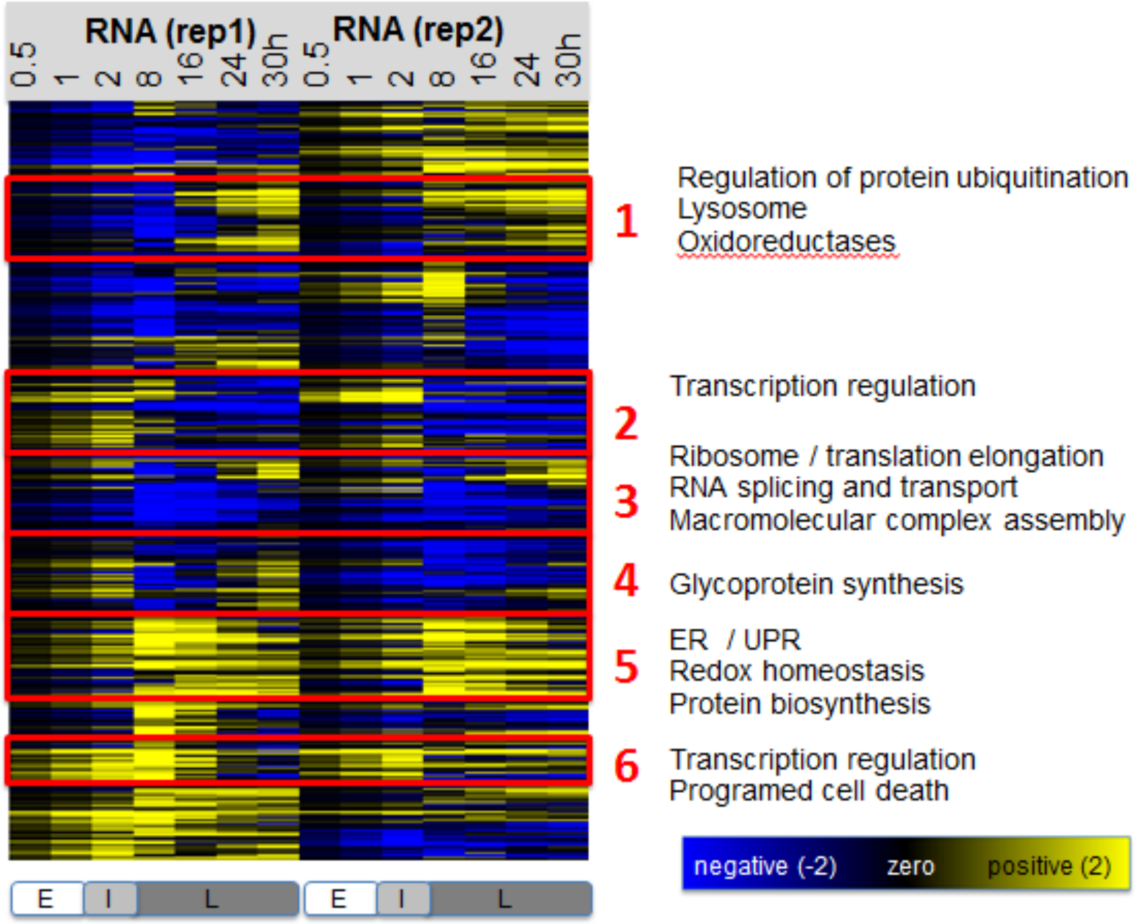

Appendix Figure S6. Transcriptome measurements are highly reproducible

A. The figure shows the agreement between the two replicates, as measured by Pearson correlation coefficients for the complete transcriptomics data (*N*=16,704, normalized, log-transformed intensity measurements). The RNA data is highly reproducible, with *R*>0.97 for all time points. Rep1 and rep2 denote the two replicates.

B. The table shows the average fold-change (and standard deviation) observed between replicate measurements across the time points

A.

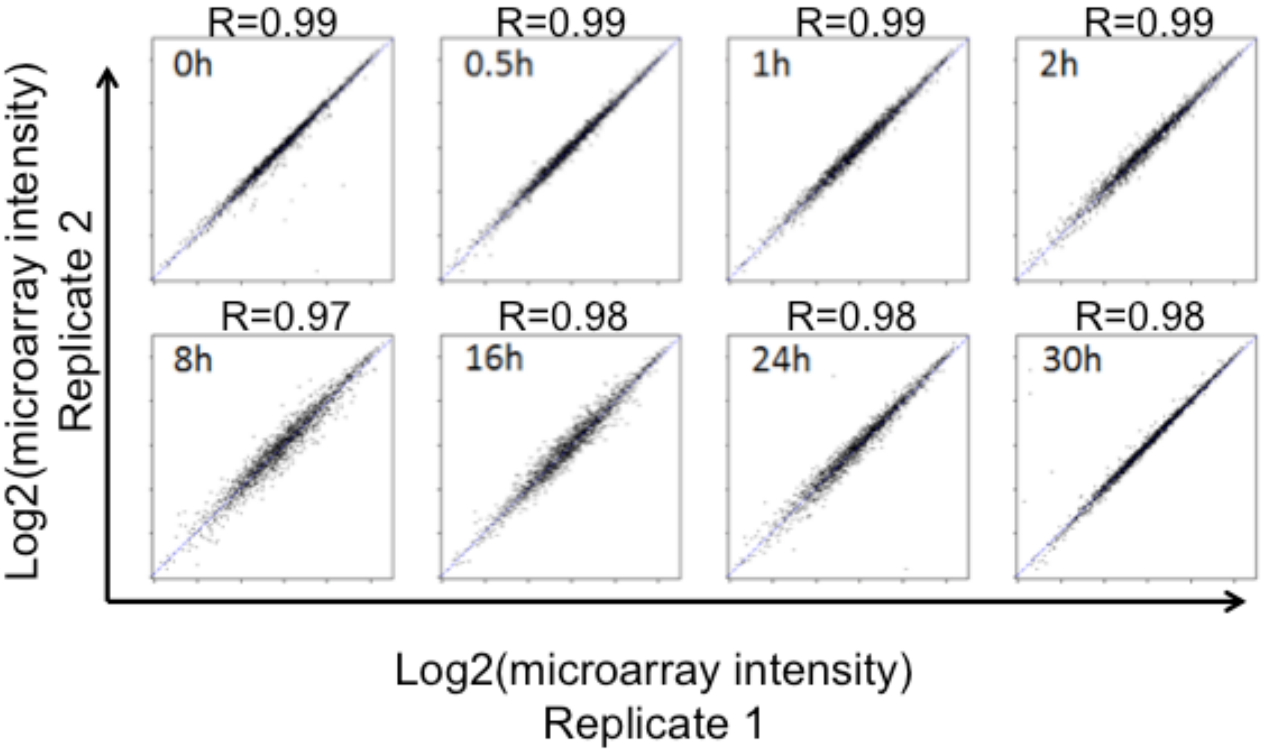

B.

**Time points (hours)**	**0**	**0.5**	**1**	**2**	**8**	**16**	**24**	**30**
**Average fold-change (log base 10)**	-0.04	-0.01	0.02	0.08	-0.10	-0.06	0.00	0.03
**Stdev (log base 10)**	0.25	0.17	0.16	0.33	0.59	0.29	0.23	0.29
**Average fold-change (linear)**	0.96	0.99	1.02	1.09	0.90	0.95	1.00	1.03
**Stdev (linear)**	1.28	1.18	1.17	1.40	1.80	1.33	1.26	1.34

Appendix Figure S7. Transcriptome measurements are accurate

To validate the accuracy of the quantitative measurements of mRNA concentrations, we compared the microarray measurements to RNA-seq data generated from exactly the same sample for two time points. The figure shows the scatter plots of the normalized microarray and RNA-seq data for time point 0 and 2hrs. The upper panel shows all data, the lower panel the filtered data. Filtering was based on the findings by Hebenstreit *et al*. that reported low accuracy for RNA-seq measurements with RPKM<1 (Hebenstreit et al, 2011). The correlation between the microarray and the RNA-seq data was very high with highly significant P-values (Pp and Ps for Rp and Rs, respectively, shown in the figure), suggesting that our quantitative measurements are accurate.

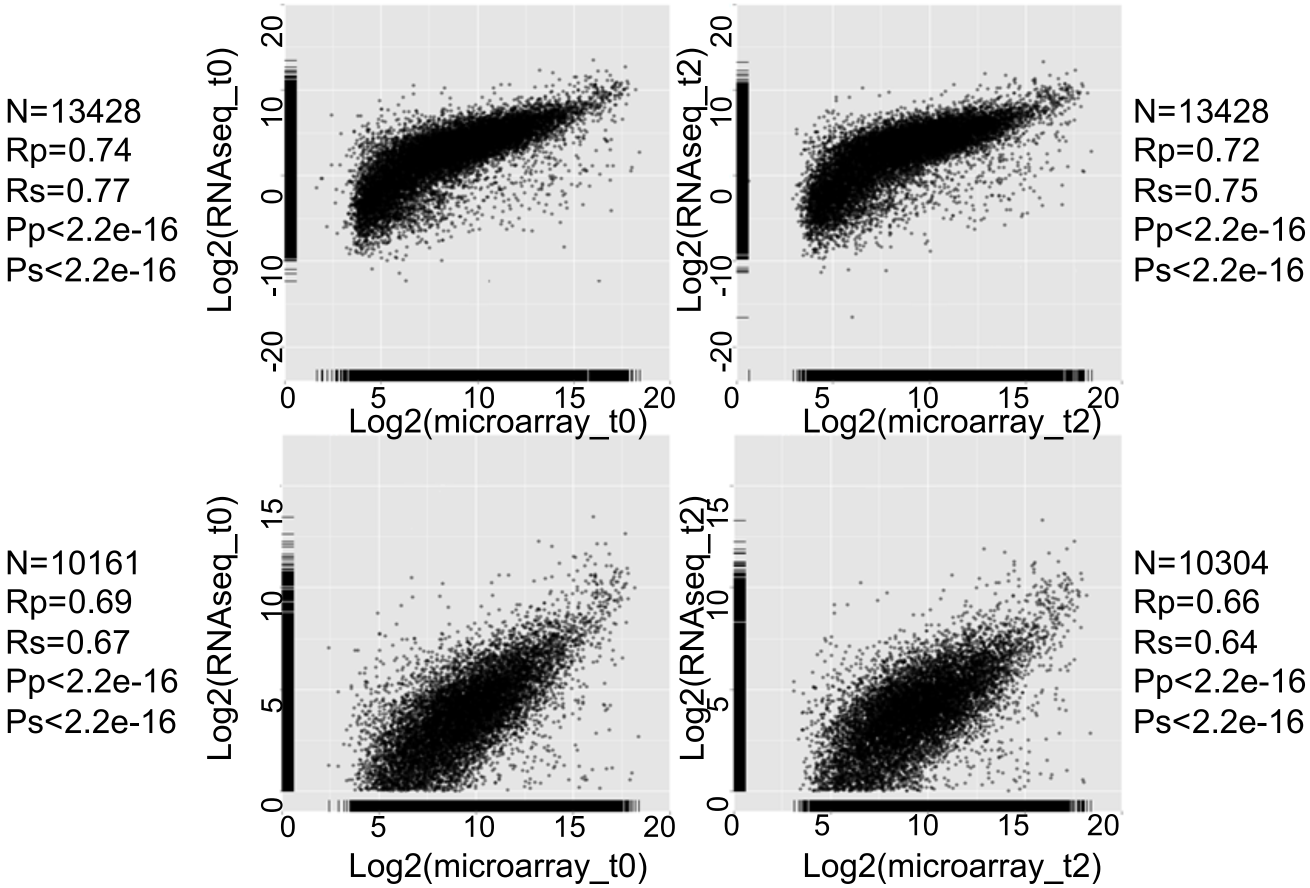

Appendix Figure S8. Combining sub-cellular fractions to increase proteomics coverage

To increase the number of identified proteins, we split each cell sample into cytosolic and nuclear fraction. The 'nuclear' fraction was enriched for membrane organelles and the nucleus, but not a pure isolation of nuclei. **A.** While proteins identified in the two fractions overlap, combining the output from the two fractions increased coverage from ~1,300-~2,200 proteins to a total of ~2,600 per sample. **B.** We tested different methods to combine the two fractions, and to evaluate the results, the protein concentrations were compared to the RNAseq data. Protein concentrations were estimated both based on MS1 intensities (left) and spectral counts (right). The methods we used to quantify the proteins were: using two fractions separately, calculating the average or sum of the two fractions, using the maximum value between the two, and using the MaxQuant software to directly treat the fractions similar to fractions from liquid chromatography. The last method, using MaxQuant provided the best results (greatest coefficient) and was used for further data analysis.

A.

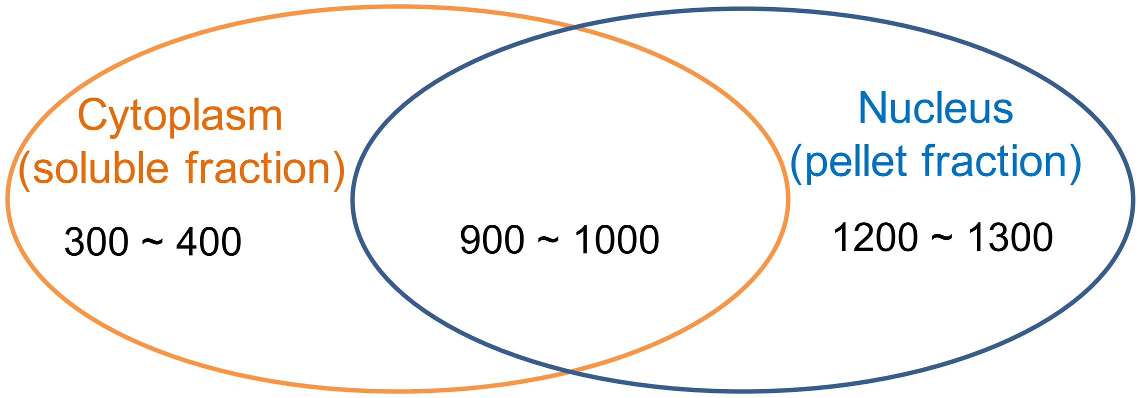

B.

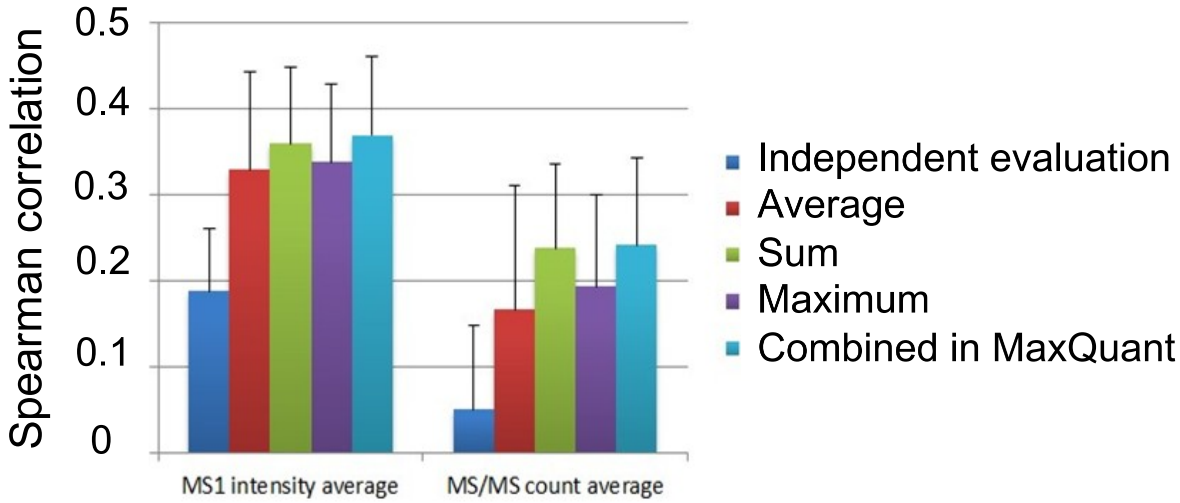

Appendix Figure S9. Heatmap of entire protein expression data shows consistent expression patterns of genes with similar functions

The heatmap shows the normalized, relative protein expression values measured across the time points in two replicates, prior to matching with mRNA data (*N*=1,820). The distance threshold of 0.467 was chosen for total proteome data, and 25 clusters were generated (using the Perseus algorithm as described above). We performed function enrichment analysis using the DAVID GO term enrichment tool on the largest cluster (see **Appendix Dataset EV2**)(Huang et al, 2009a; Huang et al, 2009b). Shown are significantly enriched functions, FDR<5%. Bottom panels E, I, L mark the early, intermediate, and late phase, respectively.

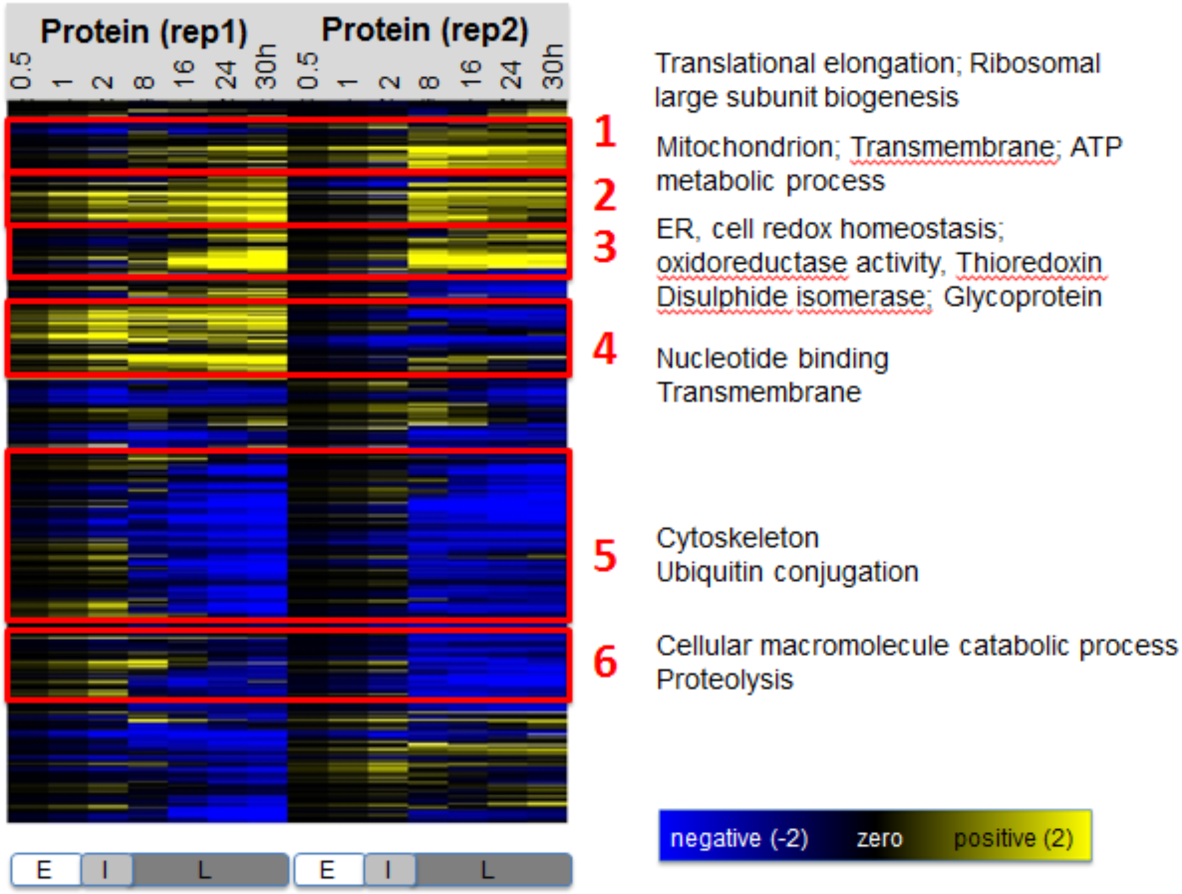

Appendix Figure S10. Proteomics measurements are consistent across replicates

A. We used the MaxQuant software (v. 1.3.0.5) (Cox & Mann, 2008) for protein identification (UniProt human database) and subsequent quantification. We quantified 3,235 proteins in total. To obtain a high-quality dataset, we only retained those proteins without missing values, i.e. 16 measurements across all time points and replicates. This filtering retained 1,820 proteins which were then matched with the RNA expression data. The scatter plots show the correlation between quantitative protein measurements for the normalized, log-transformed data (*N*=1,820). The Pearson correlation coefficient is >0.88 across all comparisons. LFQ intensity – Label Free Quantification, protein intensities as retrieved from MaxQuant.

B. The table shows the average fold-change (and standard deviation) observed between replicate measurements across the time points.

A.

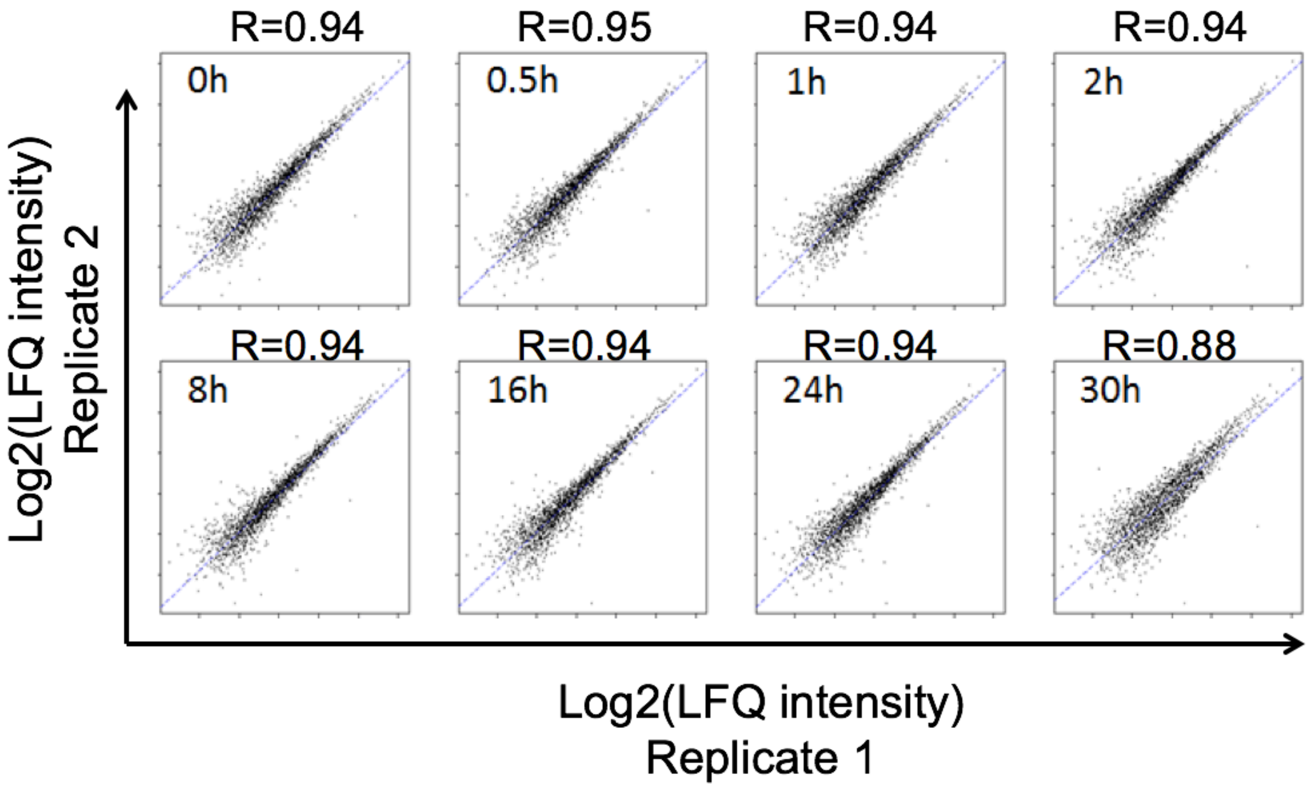

B.

Time points (hours)	0	0.5	1	2	8	16	24	30
Average fold-change (log base 10)	-0.04	-0.04	-0.04	-0.03	0.14	0.04	0.03	0.03
Stdev (log base 10)	0.79	0.70	0.68	0.83	0.89	0.74	0.83	1.06
Average fold-change (linear)	0.96	0.96	0.96	0.97	1.15	1.04	1.03	1.03
Stdev (linear)	2.20	2.01	1.97	2.29	2.43	2.09	2.29	2.88

Appendix Figure S11. Proteomics provides appropriate estimates of protein concentrations

To verify the mass spectrometry (MS) based proteomic measurements, we confirmed protein concentration changes for a selected set of proteins using western blot. The figure shows the western blot data (left), the mass spectrometry data (middle), and the PECA transformed regulatory data (right). Note that the regulatory data includes variation at the mRNA level which explains some descrepancies with the concentration data.

For the western blots, we scraped cells from plates and homogenized them in 50 mM Tris-HCl (pH 7.5), 1% NP-40, 0.5% deoxycholic acid, 1% Triton X-100, 1% sodium dodecyl sulfate (SDS), and 2% complete protease inhibitor cocktail (Roche, Mannheim, Germany). The homogenates were centrifuged and supernatants were collected and measured by Bradford protein assay. We then diluted samples to be at the same concentration, and mixed them 1:1 with electrophoresis sample buffer. Proteins were separated using SDS polyacrylamide gel and transferred to polyvinylidene fluoride membranes (Gelman Sciences Inc., Ann Arbor, Michigan, USA). Non-specific binding was blocked for 1hr at room temperature with 5% non-fat milk or 5% bovine serum albumin. The blots were incubated at 4°C for 8h with the primary antibodies, followed by horseradish peroxidase-conjugated secondary antibodies, and visualized by the enhanced chemiluminescence procedure (Pierce Biotechnology, IL, USA).

We selected ATP synthase subunit delta (ATP5D), 20S proteasome core subunits (PSMA7, PSMB1), and SEC61 complex subunit alpha1 (SEC61 α 1) using their respective antibodies (anti-ATP5D Rabbit pAb, 14893-1-AP, Proteintech; anti-20S Proteasome Core Subunits Rabbit pAb, ST1053, EMD Millipore; anti-SEC61A1 Rabbit pAb, SAB2700328, Sigma-Aldrich). MS-based expression values are estimated from normalized, log-transformed LFQ protein intensities as obtained from MaxQuant.

Stars (*) indicate highly expressed proteins in the western blots and their corresponding values in the proteomics data.

A. ATP5D is a subunit of the ATP synthase complex (main text). The MS data of ATP5D shows that protein concentration increases by more than two fold during the intermediate phase, and the increased expression of ATP5D after 16 hours is confirmed by western blot using an anti-ATP5D antibody.
B. The SEC61 protein complex is an ER membrane protein translocon, functioning in translocating related proteins into or out of the ER. It is a doughnut shaped pore in the ER membrane with three major subunits, α, β and γ. We detected two of the three subunits in the MS data, SEC61α1 and SEC61β, and both of them were up-regulated during ER stress, especially in the late phase (8h-30h). Anti-SEC61α1 primary antibody was used to stain α1 subunit of Sec61 protein complex in the western blot, and it showed similar expression pattern compared to the MS results: expression reaches a maximum after 16 hours.
C. Thirteen of the total 14 proteasome core subunits were detected in the proteomics data, with the exception of subunit α5. The antibody against the 20S proteasome core can detect the α5, α7, β1, and β7 subunits, but cannot distinguish between expression changes of the individual subunits. The MS data for three of the four subunits is shown in the right panel (missing α5). The western blot confirms the expression changes for two of the three detected subunits: expression peaks between one and eight hours of the treatment, and declines afterwards.

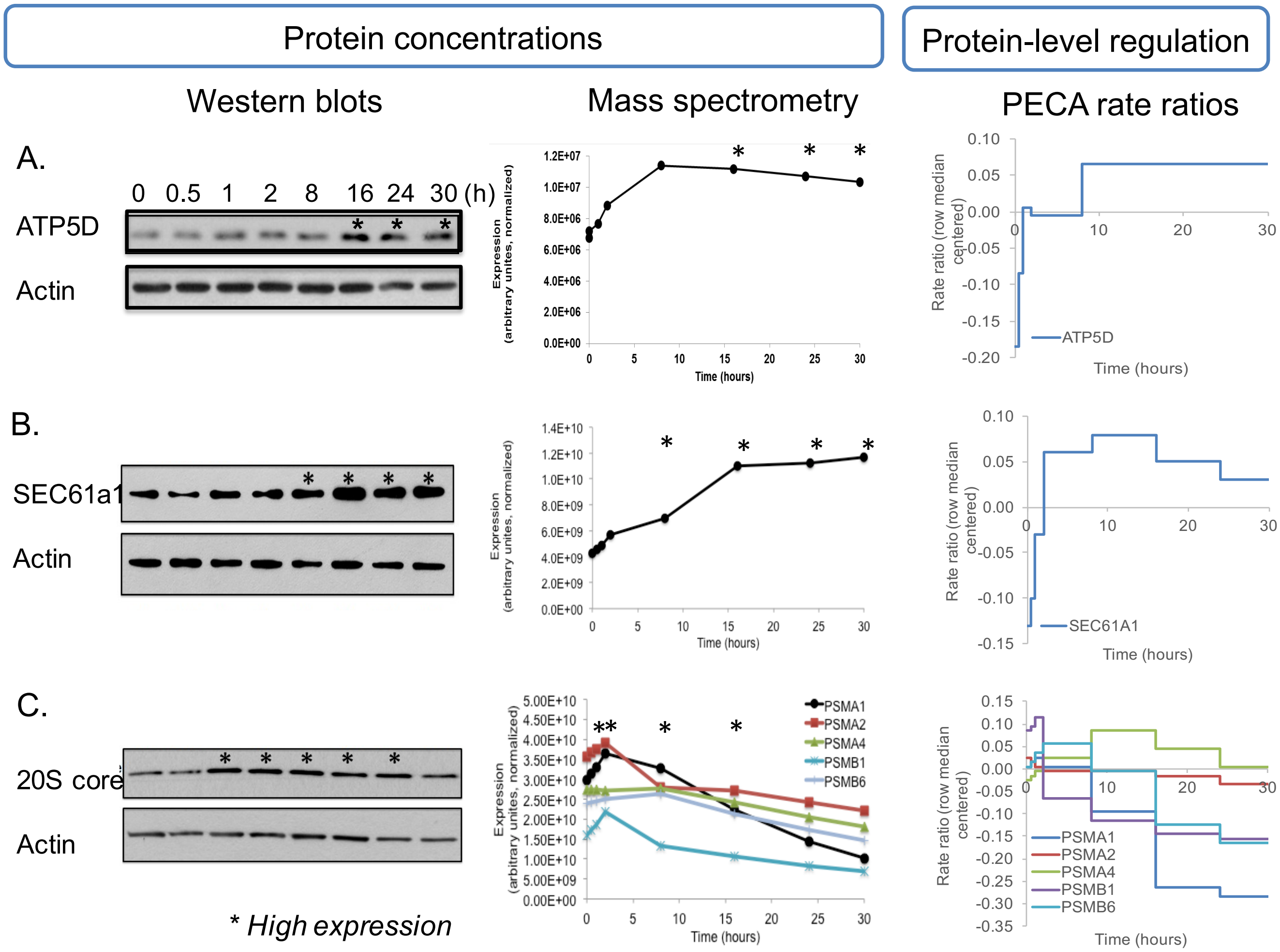

Protein concentrations
Protein-level regulation
Western blots
Mass spectrometry
PECA rate ratios
A.
0 0.5 1 2 8 16 24 30 (h)
ATP5D
Actin
B.
SEC61a1
Actin
C.
20S core
Actin
* High expression
Expression (arbitrary unites, normalized)
Time (hours)
Rate ratio (row median centered)
ATP5D
SEC61A1
PSMA1
PSMA2
PSMA4
PSMB1
PSMB6

Appendix Figure S12. Housekeeping genes are comparatively constant in their protein expression
We extracted the proteomics measurements of the normalized LFQ intensities (as estimated by MaxQuant) for several housekeeping genes (A.-C.). Most of the genes show constant expression across the measurement time indicating that proteomics measurements are correct.

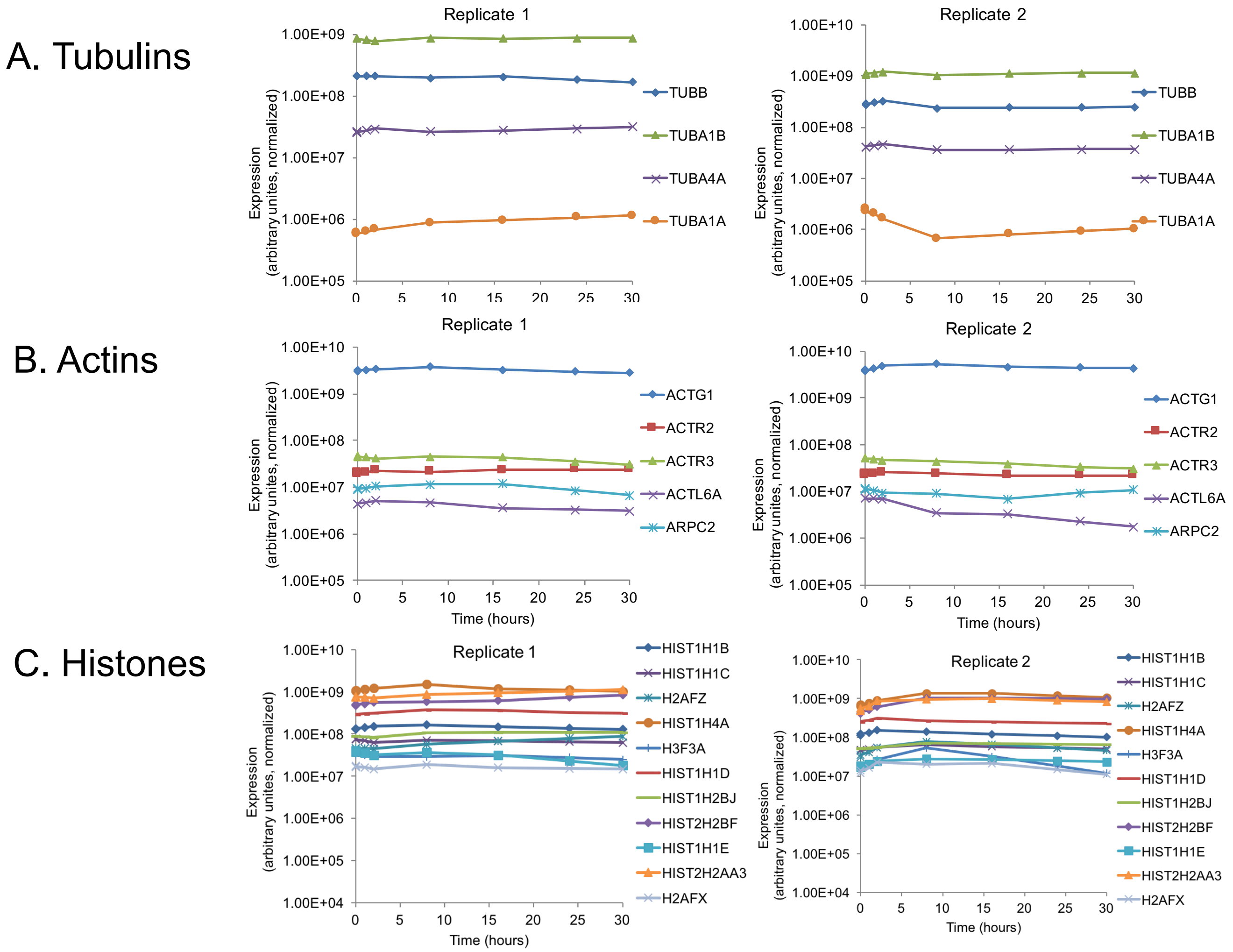

Analysis of the integrated mRNA and protein expression data

The following figures present results in addition to what is presented in the main text, analyzing the high-confidence/core dataset of integrated mRNA and protein expression data (N=1,237).

Appendix Figure S13. mRNA and protein concentrations correlate partially across the time course

A., B. The panels show the relationship between normalized protein and mRNA expression values for each time point and replicate.

C. For each gene, we calculated the Pearson correlation coefficient between the replicate time series measurements of normalized, absolute, log-transformed mRNA and protein concentrations, respectively. These correlation coefficients provide a measure for reproducibility between biological replicates. At a total of eight datapoints, an R=0.7 corresponds to a P-value=0.053. As can be seen, most genes are shifted towards high R-values suggesting good reproducibility. However, a fraction appears to have low correlation between the biological replicates. In many cases, such low correlation can be explained either by very small expression changes that still result in different profiles between the two replicates (e.g. HSC70 with <1.5 fold protein expression change in both replicates but a correlation of R=0.09) or by a peak in expression changes that occurs at two different time points, e.g. many genes show maximal (minimal) changes at 2 hours in replicate 1 and at 8 hours in replicate 2. We would argue that, despite low correlation, these profiles are still equivalent in their overall trend.

A. Replicate 1

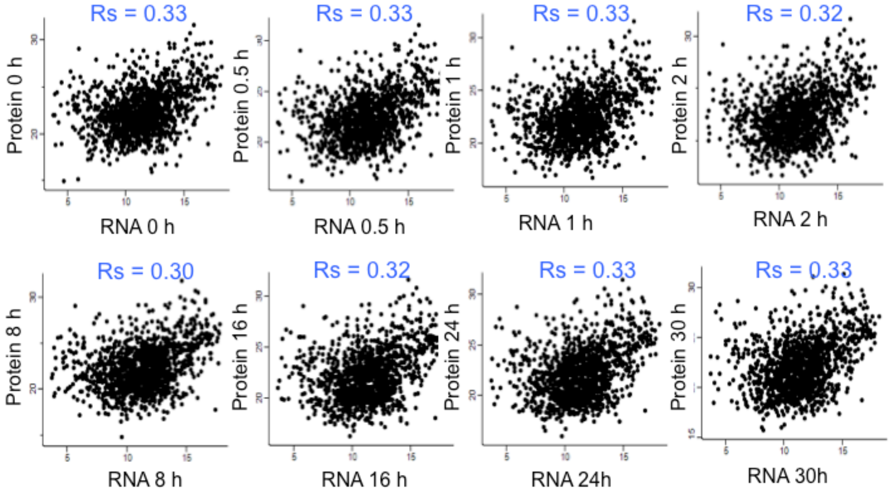

B. Replicate 2

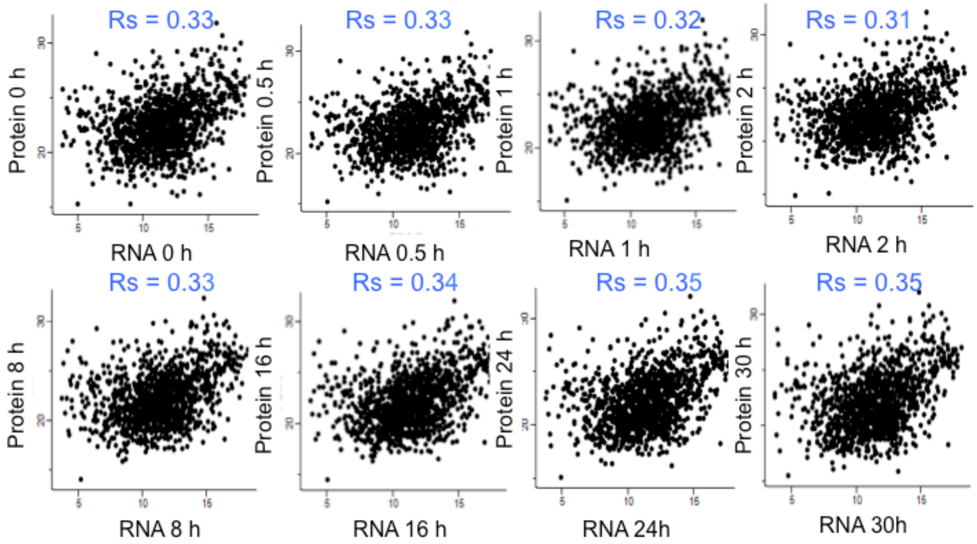

C. Correlation between replicate time series measurements

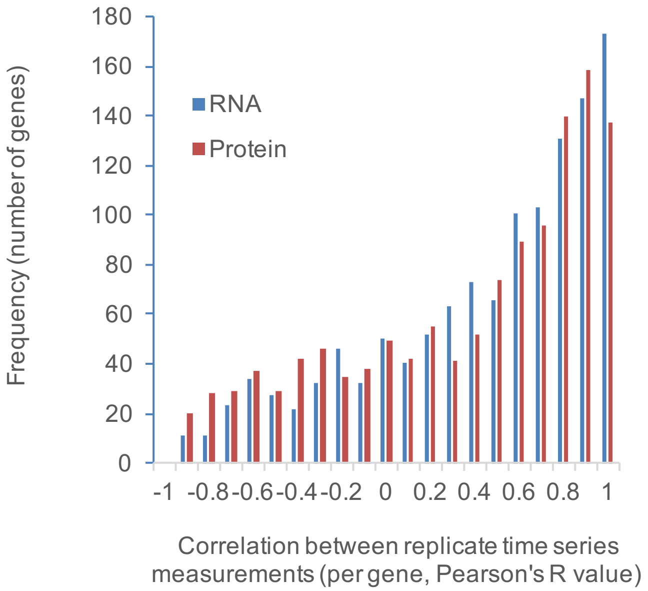

Appendix Figure S14. Complete function annotation shows distinct expression patterns for mRNA and protein

The heatmap shows the normalized, relative expression values for both mRNA and protein measured in one replicate (N=1,237). The eight largest clusters are marked with red boxes (letters A to H). The cluster definition is presented in the **Appendix Dataset EV2**. GO terms enriched in eight major clusters are shown on the right (FDR < 5%). Bottom panels E, I, L mark the early, intermediate, and late phase, respectively.

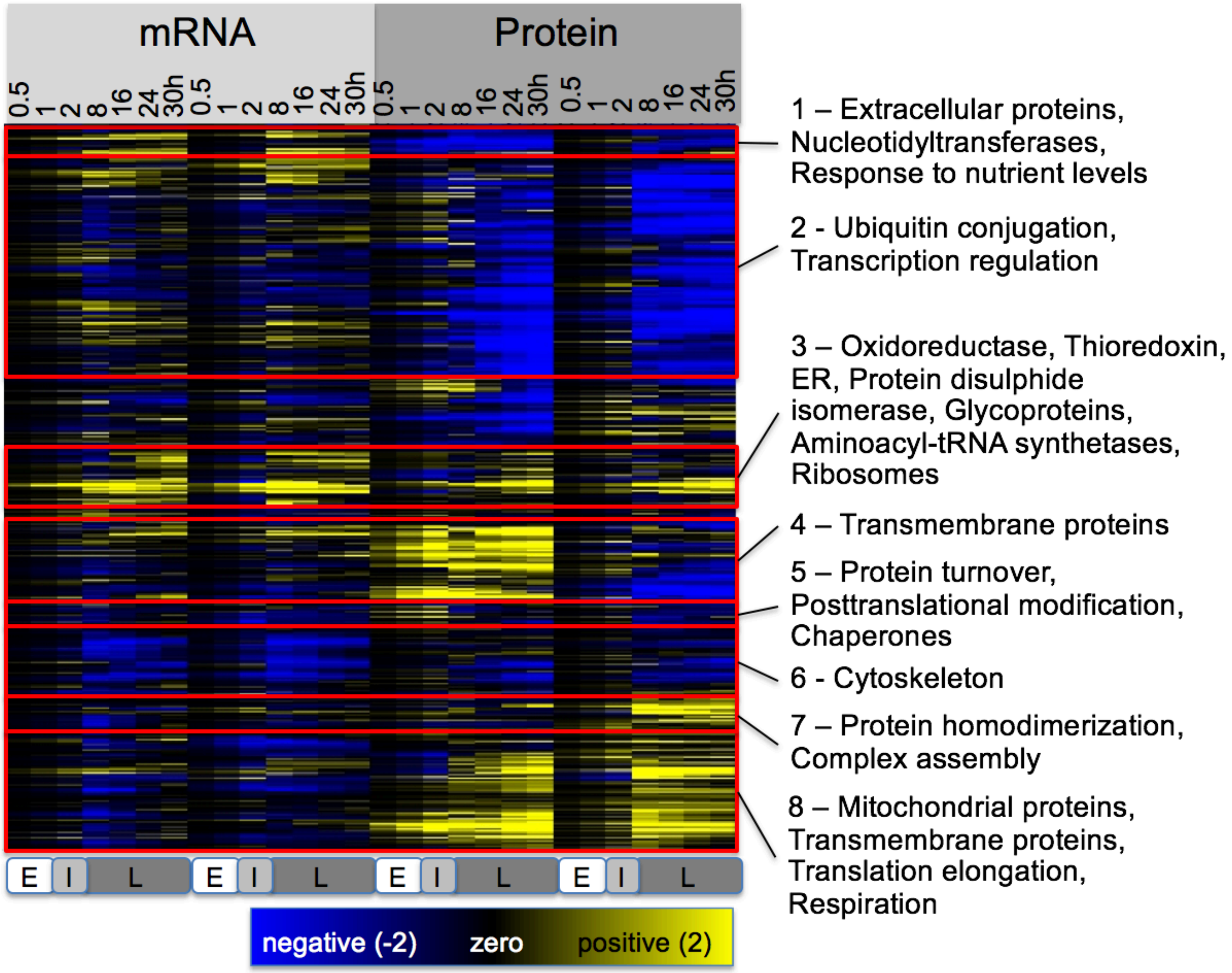

Appendix Table S1. Fold-changes and dynamic range of the expression data

The table shows the overall average of the minimum and maximum fold changes for each replicate of the RNA and protein data from our analysis and the work by Jovanovic et al. (Jovanovic et al, 2015). In our study, the average fold change of protein data is larger than RNA, while in Jovanovic' study it is *vice versa*. This difference is likely due to our study affecting the proteome in major ways, while the LPS response (Jovanovic) is largely a transcriptional response. The dynamic range of absolute molecule concentrations are similar for both mRNA and protein, spanning approximately five orders of magnitude. * Dynamic ranges were not calculated as the proteomics data was reported as SILAC ratios which do not include absolute values.

	**Time average of the minimum fold change (log natural base)**	**Time average of the maximum fold change (log natural base)**	**Range of absolute molecule concentrations (quantile normalized intensity)**	**Approx. dynamic range (orders of magnitude)**
**This study: ER stress response**				
**RNA Replicate 1**	-1.56 ± 0.81	2.51 ± 1.06	1.76 – 3.57 x 10E5	5
**RNA Replicate 2**	-1.76 ± 0.85	2.48 ± 1.06	4.73 – 3.57 x 10E5	5
**Protein Replicate 1**	-3.88 ± 1.75	6.07 ± 2.74	2.70 x 10E4 – 3.86 x 10E9	5
**Protein Replicate 2**	-3.00 ± 1.40	3.08 ± 1.61	1.69 x 10E4 – 5.34 x 10E9	5
**Jovanovic et al. Science 2015: LPS response**				
**RNA Replicate 1**	-2.96 ± 0.81	5.42 ± 2.72	*	*
**RNA Replicate 2**	-3.38 ± 0.96	6.60 ± 0.78	*	*
**Protein Replicate 1**	-1.64 ± 0.14	2.76 ± 0.52	*	*
**Protein Replicate 2**	-2.10 ± 0.50	2.71 ± 0.42	*	*

Appendix Figure S15. Changes between consecutive gene expression mRNA and protein measurements are maximal around 8 hours

The panels show Pearson's R^2 values between normalized, absolute expression values of consecutive time points for the mRNA (A) and protein (B) data. For both RNA and protein, the largest divergence occurred at around the 8h mark. However, as the main text discusses, the RNA concentrations appear to return to the pre-treatment levels, while protein concentrations stabilized to new equilibrium levels. RNA-1, Protein-1 and RNA-2, Protein-2 refer to replicate 1 and 2, respectively.

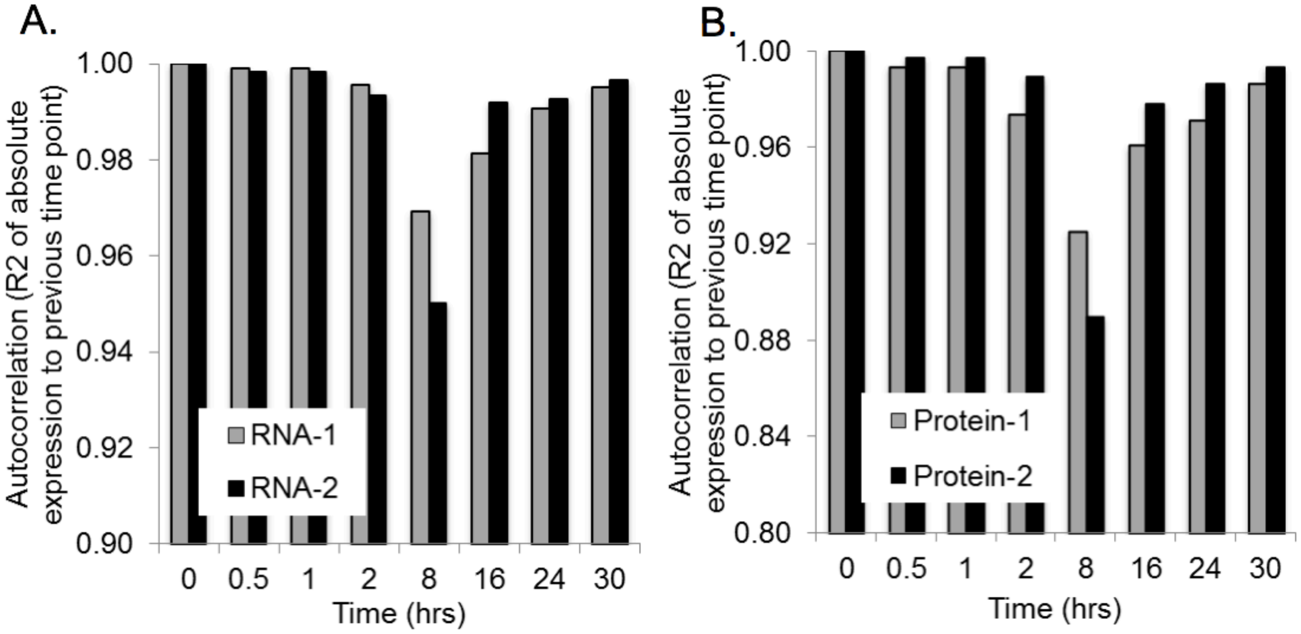

Appendix Figure S16. Some genes show discordance between mRNA- and protein-level regulation

A., B. First, we aimed to assess the extent to which discordant regulation occurs, i.e. in how many genes mRNA- and protein-level regulation occur in opposite directions. To do so, we calculated the Pearson correlation was in each replicate (A., B.) between the time course RNA- and protein-level rate ratios for each gene. Genes with (i) significant RNA- and protein-level CPS (FDR<0.05) at any time point and (ii) rate ratio correlation below -0.5 were considered to be subject to discordant regulation, i.e. counteracting regulation at the mRNA and protein level. Fifty-five genes adhered to this definition in both replicates and are listed in **Dataset EV1**. In the heat map, the rate ratios were normalized against the first time period in both RNA and protein level analyses.

C. In a modified analysis, we attempted to extract genes that were candidates for 'buffering', i.e. in which discordant mRNA- and protein-level regulation results in constant protein concentrations despite changes in mRNA expression. Again, we extracted genes whose PECA analysis resulted in at least one significant change (FDR<0.05) at the mRNA- and one at the protein-level, in opposing directions. We filtered this set further for protein concentration changes of less than 1.5-fold (normalized, relative log-ratios). This procedure resulted in three genes shown in this figure. HSPA8 is also called HSC70 and a constitutively expressed chaperone that suppresses apoptosis through transcription inhibition. Its protein concentration is at high levels throughout the experiment, despite a temporary dip in mRNA concentrations at the 2-hour time point that are countered by increased protein-level regulation.

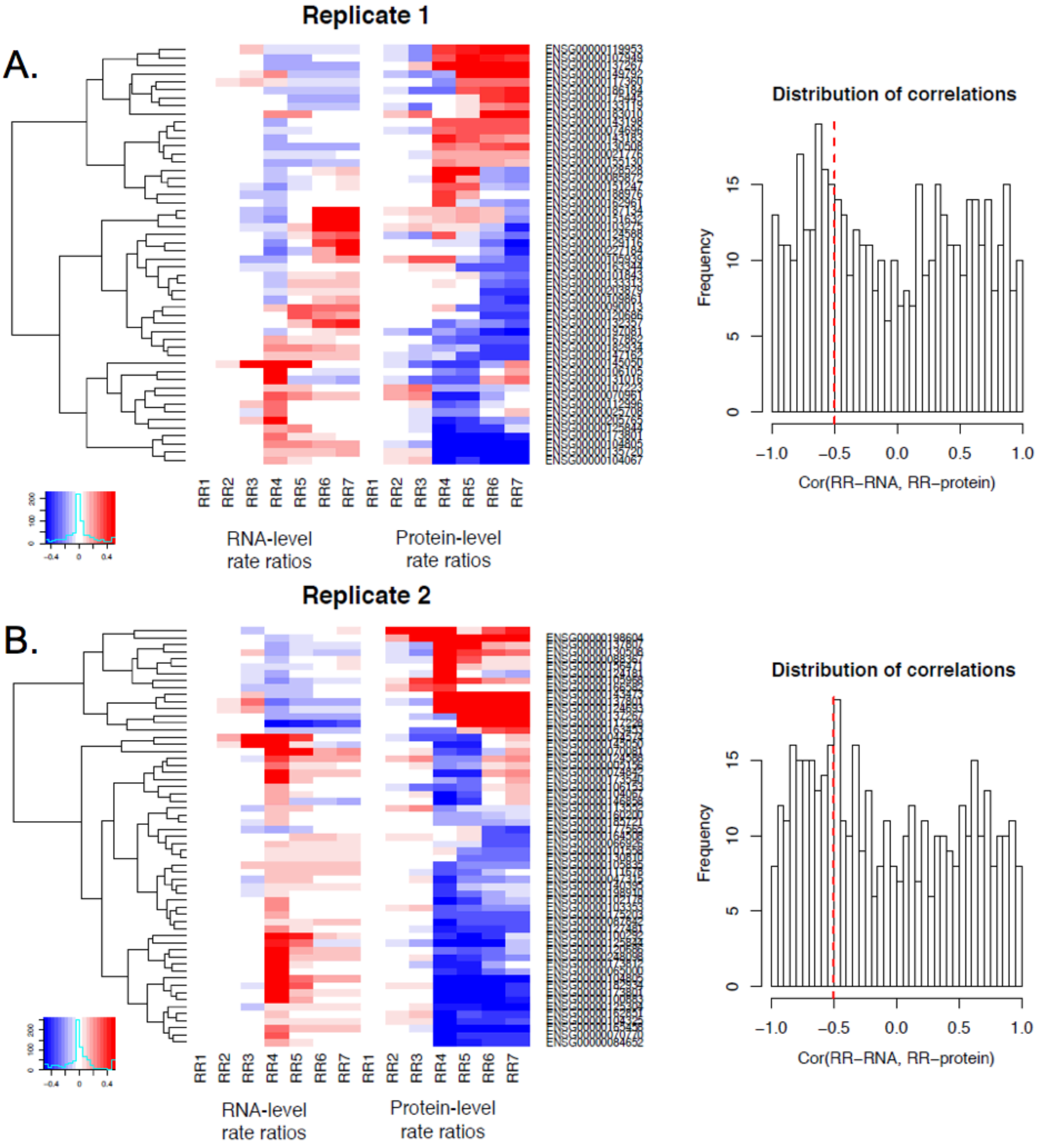

Replicate 1
A.
Distribution of correlations
Frequency
0
5
10
15
−1.0
−0.5
0.0
0.5
1.0
Cor(RR−RNA, RR−protein)
RR1
RR2
RR3
RR4
RR5
RR6
RR7
RR1
RR2
RR3
RR4
RR5
RR6
RR7
RNA-level rate ratios
Protein-level rate ratios
Replicate 2
B.
Distribution of correlations
Frequency
0
5
10
15
−1.0
−0.5
0.0
0.5
1.0
Cor(RR−RNA, RR−protein)
RR1
RR2
RR3
RR4
RR5
RR6
RR7
RR1
RR2
RR3
RR4
RR5
RR6
RR7
RNA-level rate ratios
Protein-level rate ratios

C.

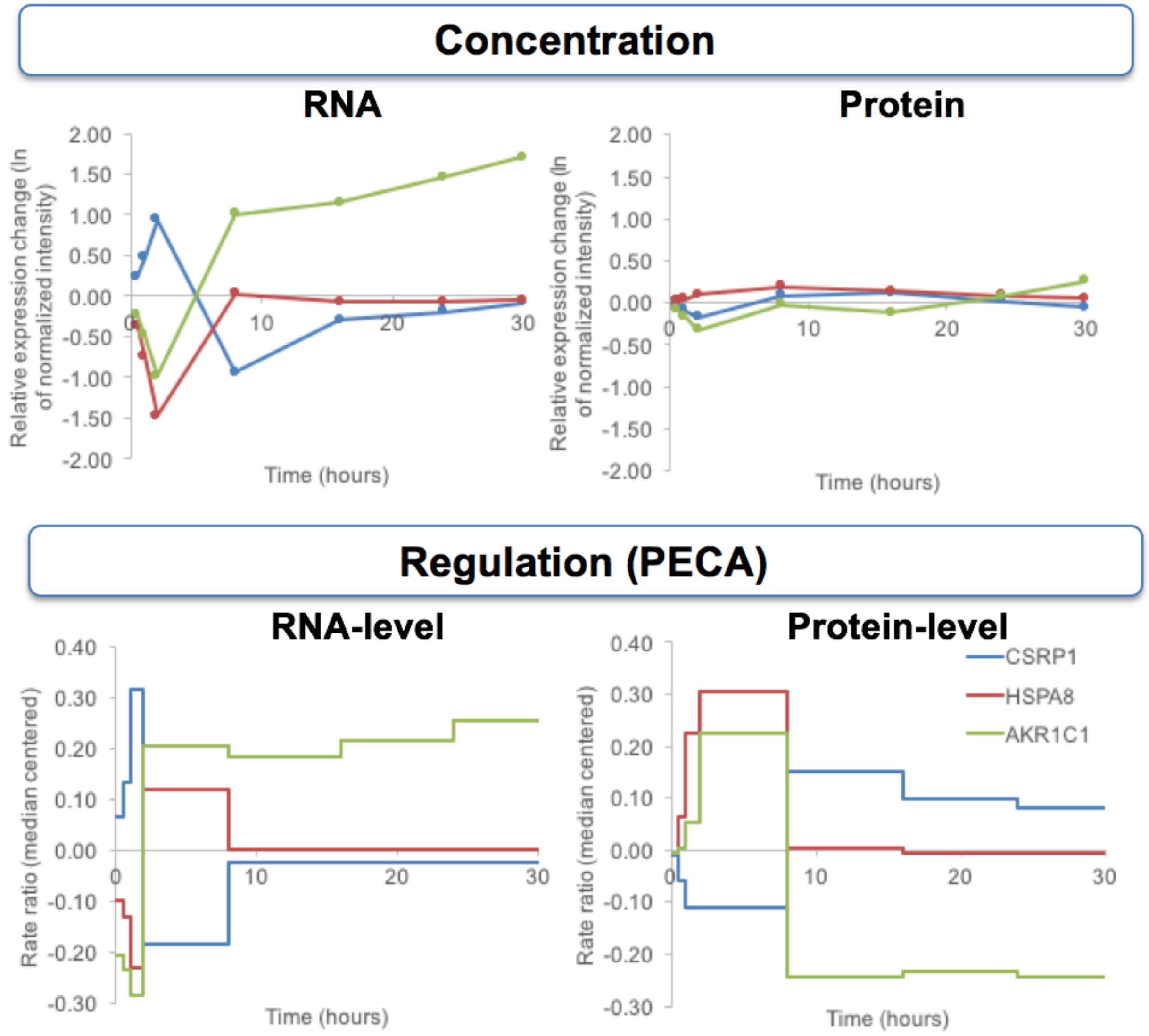
Concentration
RNA
Protein
Relative expression change (ln of normalized intensity)
2.00
1.50
1.00
0.50
0.00
-0.50
-1.00
-1.50
-2.00
0
10
20
30
Time (hours)
Regulation (PECA)
RNA-level
Protein-level
Rate ratio (median centered)
0.40
0.30
0.20
0.10
0.00
-0.10
-0.20
-0.30
CSRP1
HSPA8
AKR1C1

Appendix Figure S17. Splice variants of aminoacyl-tRNA synthetases show some evidence for differential protein expression

A number of aminoacyl-tRNA synthetases show striking gene expression patterns in which, after an initial spike in RNA concentrations, protein synthesis appears to be upregulated in the late phase, despite decreasing RNA concentrations (see main text). Protein synthesis is delayed by several hours, compared to RNA synthesis. The differential regulation of aminoacyl-tRNA synthetases at the post-transcriptional level has been observed before in different contexts (Chen et al, 2012; Guan et al, 2014; Kwon et al, 2011; Park et al, 2012; Wei et al, 2014), but the functional significance and underlying molecular mechanism has been unknown. A recent publication delivered an intriguing explanation: aminoacyl-tRNA synthetases were observed to occur in alternative splice variants that often lack the catalytic domain but which often have, due to additional domains, additional 'moonlighting' functions (Lo et al, 2014).

Based on these findings, we hypothesized that the discrepancy between mRNA and protein expression patterns for some genes in our data might be explained by the differential expression of splice variants. If this hypothesis was true, we would observe different expression patterns for peptides across the gene's alternative splice variants. Since the original mass spectrometry experiment was not designed to target splice variants, we examined the data manually to test the above hypothesis for example proteins.

Note that profiles for the analysis of splice variants are much noisier as they were conducted on a different dataset. For the main analyses, we used MaxQuant's *proteinGroups.txt* output file which sums over all peptides contributing to a protein group. This dataset was then normalized as described in the main text. In comparison, the analyses of splice variants was conducted using the *peptides.txt* file to obtain information on individual peptides that mapped to different splice variants.

Of the 13 cytosolic aminoacyl-tRNA synthetases observed in our experiment, eight were observed with more than one sequence variant as defined by the Uniprot sequence database (SARS, AARS, GARS, YARS, EPRS, IARS, QARS, MARS). We examined the peptide abundances for each of these eight proteins, measured by two different ways: the scaled number of unique observations of the peptide (on the left), and the scaled MS1 intensity of the peptide (on the right). All data was obtained from the MaxQuant output file (peptides.txt). Scaling refers to a normalization procedure in which each value is adjusted to the overall sum of all values in this experiment. The different entries shown for each enzyme are protein variants (their Uniprot IDs). If two variants are listed in the same group, their peptide data was common to both, i.e. we could not distinguish which of the variants the peptides originated from. In comparison, the data between different groups of sequence variants is unique to each group.

For most of the twelve observed aminoacyl-tRNA synthetases, most observed peptides were not informative as they were either shared (common) across all sequence variants, present at very low numbers or not observed at several time points. For three enzymes (AARS, IARS, QARS), enough information could be assembled to identify groups of peptides from distinct sequence variants, and the groups displayed different temporal expression patterns: for AARS, IARS, and QARS (A-C), one set of sequence variants show different average expression across corresponding peptides compared to the rest of the sequence variants. Since the data in the main text averages intensities across all sequence variants, such differential expression of splice variants is masked, but could explain discrepancies observed between RNA and protein expression.

Appendix Figure S18 shows in more detail the example of differential expression of splice variants in QARS.

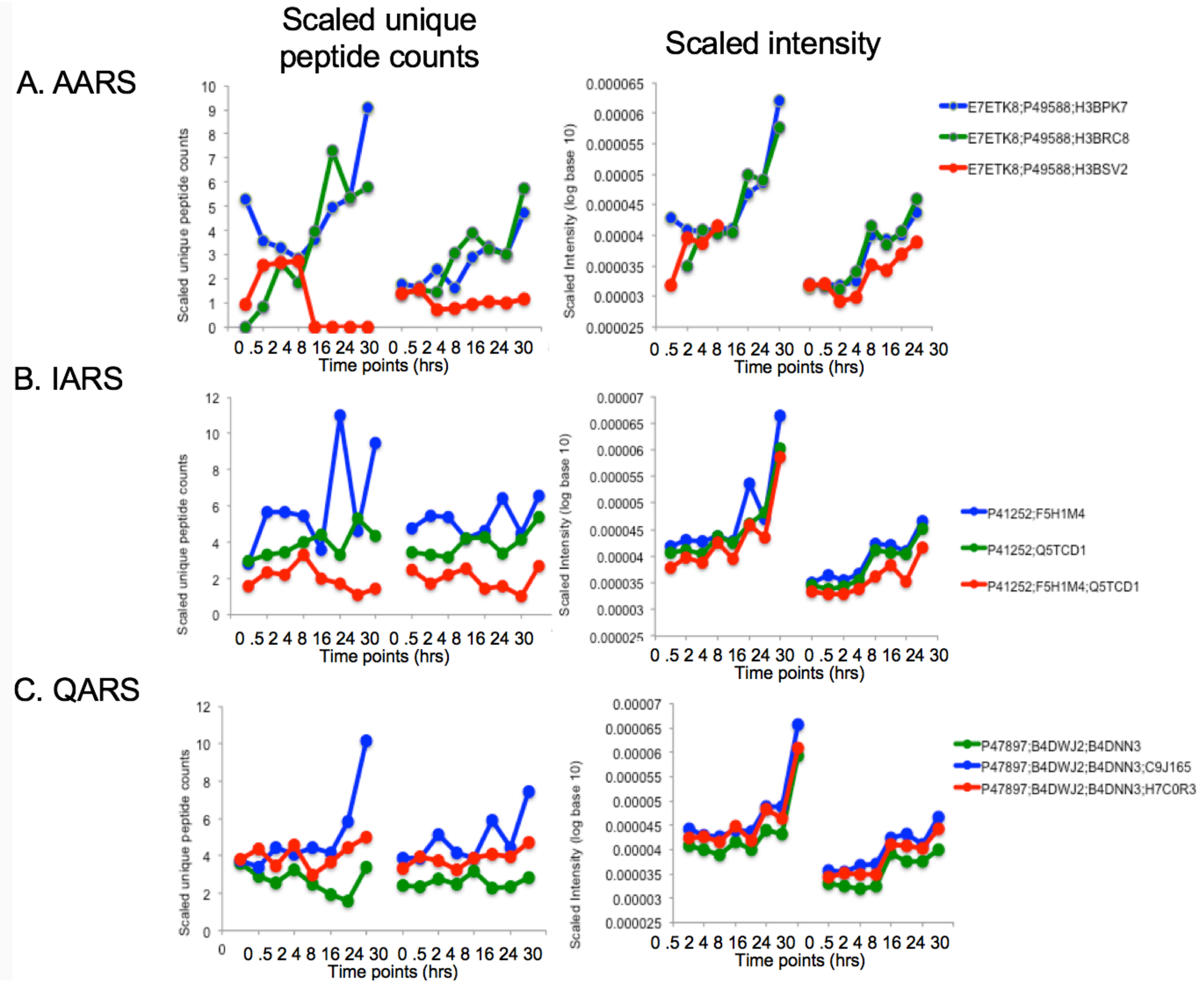
Scaled unique peptide counts
Scaled intensity
A. AARS
B. IARS
C. QARS
Scaled unique peptide counts
Scaled Intensity (log base 10)
Time points (hrs)
E7ETK8;P49588;H3BPK7
E7ETK8;P49588;H3BRC8
E7ETK8;P49588;H3BSV2
P41252;F5H1M4
P41252;Q5TCD1
P41252;F5H1M4;Q5TCD1
P47897;B4DWJ2;B4DNN3
P47897;B4DWJ2;B4DNN3;C9J165
P47897;B4DWJ2;B4DNN3;H7C0R3

Appendix Figure S18. The three peptide variants of QARS show different expression patterns

This figure shows, at the example of the glutaminyl-tRNA synthetase QARS, how differential expression of alternative splice variants could be detected. Uniprot reports several variants for QARS (P47897;B4DWJ2;B4DNN3;H7C0R3;C9J165). Several peptides were detected at different counts (A). P47897, B4DWJ2, and B4DNN3 have identical sequences, hence we show only P47897. The sequences for H7C0R3 and C9J165 are shorter than those for P47897 (B). The observed peptides map to the protein sequences, and some examples are highlighted in different colors (B, blue, green, red). For example, the FDDTNPEKKEEAK peptide (green) occurs in P47897;B4DWJ2;B4DNN3, but not in the other variants. The FHKPGENYK peptide (blue) only occurs in the P47897;B4DWJ2;B4DNN3;C9J165 group, but it does not occur in H7C0R3. The GFHQVPFAPIVFIER peptide (red) does not occur in C9J165. We averaged the group-specific scaled, unique peptide counts, and these are shown in **Appendix Figure S17**. The P47897;B4DWJ2;B4DNN3 group (green) has an expression pattern different to the other two groups: some peptides specific to the H7C0R3 and C9J165 containing groups (blue and red) are up-regulated in the later phase of the experiment (**Appendix Figure S17C**).

Since the two shorter splice variants appear to be up-regulated towards the later phase of the experiment (**Appendix Figure S17C**), we asked if their sequences and domain architecture may reveal anything about their function. Panel C shows the domain annotation for the three sequences (note that the coloring of the domains has a different meaning to the colors in A and B). The domain annotation and mapping of the splice variants shows that both shorter sequence variants may be dysfunctional with respect to catalyzing the aminoacylation of the respective tRNA: the catalytic domains are mostly truncated. The upregulation of these proteins during ER stress may have roles other than stimulating translation.

A. Peptides

**P47897;B4DWJ2;B4DNN3**	
**FDDTNPEKEEAK**	16
LGYFSVDPDSHQGK	15
FSEGEATLR	15
AINFNFGYAK	14
QHLEITGGQVR	14
ANNGICFLR	13
**P47897;B4DWJ2;B4DNN3;C9J165**	
**FHKPGENYK**	16
TPGYVVTPHTMNLLK	14
**P47897;B4DWJ2;B4DNN3;H7C0R3**	
**GFHQVPFAPIVFIER**	16
VIITNFPAAK	16
SLDIQVPNFPADETK	16
GPSGCVESLEVTCR	16
LFTLTALR	16
DVLNDTAPR	16
VGVTVAQTTMEPHLLEACVR	15
LAWGQPVGLR	15
HTGYVIELQHVVK	15
TDFKEEPEPGFKR	15

B. Sequences

```
>sp|P47897|SYQ_HUMAN Glutamine--tRNA ligase OS=Homo sapiens
GN=QARS PE=1 SV=1
MAALDSLSLFTSLGLSEQKARETLKNSALSAQLREAATQAQQTLGSTIDKATGILLYGLA
SRLRDTRRLSFLVSYIASKKIHTEPQLSAALEYVRSHPLDPIDTVDFERECGVGVIVTPE
QIEEAVEAAINRHRPQLLVERYHFNMGLLMGEARAVLKWADGKMIKNEVDMQVLHLLGPK
LEADLEKKFKVAKARLEETDRRTAKDVVENGETADQTLSLMEQLRGEALKFHKPGENYKT
PGYVVTPHTMNLLKQHLEITGGQVRTRFPPEPNGILHIGHAKAINFNFGYAKANNGICFL
RFDDTNPEKEEAKFFTAICDMVAWLGYTPYKVTYASDYFDQLYAWAVELIRRGLAYVCHQ
RGEELKGHNTLPSPWRDRPMEESLLLFEAMRKGKFSEGEATLRMKLVMEDGKMDPVAYRV
KYTPHHRTGDKWCIYPTYDYTHCLCDSIEHITHSLCTKEFQARRSSYFWLCNALDVYCPV
QWEYGRLNLHYAVVSKRKILQLVATGAVRDWDDPRLFTLTALRRRGFPPEAINNFCARVG
VTVAQTTMEPHLLEACVRDVLNDTAPRAMAVLESLRVIITNFPAAKSLDIQVPNFPADET
KGFHQVPFAPIVFIERTDFKEEPEPGFKRLAWGQPVGLRHTGYVIELQHVVKGPSGCVES
LEVTCRRADAGEKPKAFIHWVSQPLMCEVRLYERLFQHKNPEDPTEVPGGFLSDLNLASL
HVVDAALVDCSVALAKPFDKFQFERLGYFSVDPDSHQGKLVFNRTVTLKEDPGKV

>tr|C9J165|C9J165_HUMAN Glutamine--tRNA ligase (Fragment)
OS=Homo sapiens GN=QARS PE=4 SV=1
MAALDSLSLFTSLGLSEQKARETLKNSALSAQLREAATQAQQTLGSTIDKATGILLYGLA
SRLRDTRRLSFLVSYIASKKIHTEPQLSGWWCQGLTFQEARLSLEFHEIRTLSVHFLEQR
CHKLTGSFFSAALEYVRSHPLDPIDTVDFERECGVGVIVTPEQIEEAVEAAINRHRPQLL
VERYHFNMGLLMGEARAVLKWADGKMIKNEVDMQVLHLLGPKLEADLEKKFKVAKARLEE
TDRRTAKDVVENGETADQTLSLMEQLRGEALKFHKPGENYKTPGYVVTPHTMNLLKQHLE
ITGGQ

>tr|H7C0R3|H7C0R3_HUMAN Glutamine--tRNA ligase (Fragment)
OS=Homo sapiens GN=QARS PE=4 SV=1
QWEYGRLNLHYAVVSKRKILQLVATGAVRDWDDPRLFTLTALRRRGFPPEAINNFCARVG
VTVAQTTMEPHLLEACVRDVLNDTAPRAMAVLESLRVIITNFPAAKSLDIQVPNFPADET
KGFHQVPFAPIVFIERTDFKEEPEPGFKRLAWGQPVGLRHTGYVIELQHVVKGPSGCVES
LEVTCRRADAGEKPKAFIHWVSQPLMCEVRLYERLFQHKNPEDPTEVPGGFLSDLNLLVF
NRTVTLKEDPGKV
```

C. Domain architectures

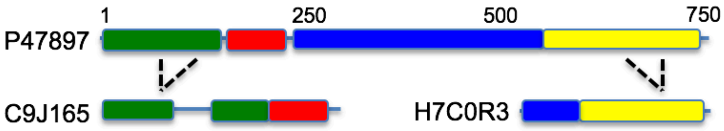

Note: Coloring of protein domains is INDEPENDENT of coloring of peptides and sequences.

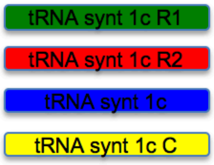

Appendix Figure S19. PECA analysis of the extended dataset

To assess the generality of our findings, we constructed an extended dataset with 2,131 RNA/protein pairs which is close to twice as big as the high-confidence/core dataset.

A. As the extended dataset comprises genes with missing data points and increased noise levels in the measurements, we constructed an additional post-processing procedure. To smooth the continuous intensity data across the time points and impute missing values, we fitted a Gaussian process (GP) regression model described in (Rasmussen, 2006). GP models require estimation of kernel parameters that determine the smoothness of the estimated curve. Since the time series is short (i.e. the number of data points per protein is small), we have to borrow information for parameter estimation across the proteins. To do so, we first perform agglomerative clustering with maximum linkage and Euclidean distance to divide the proteins into a small number of groups (i.e. 20 groups in this data) and estimate the kernel within each cluster. Using this model, we impute missing values and adjust the observed intensity values to the value on the curve for smoothing purposes. The shows an example of a gene in which there is one missing data in each replicate, represented by black and blue curves respectively, and the red circle and triangle represent the imputed values.
B. Next further processed the data as described for the core dataset and performed a PECA analysis similar to that in **Figure 5** in the main text: after grouping the normalized and centered rate ratios for the LPS experiment into ten clusters (left) using the 'Correlation' distance measure and 'Complete' linkage clustering as available in the Perseus software tool. We then calculated the average rate ratios for the four largest clusters (right) which account almost 60% of the data (1,241 genes). Note that the amplitude of the rate ratios is not meaningful, but changes between consecutive intervals. Examination of the patterns confirms the findings from **Figure 5**, albeit in a noisier way: RNA rate ratios show a spike during the intermediate phase, while most protein rate ratios change only once around the two-hours mark, and remain at the new steady-state level throughout the remainder of the experiment.

A. Example gene with missing value imputation

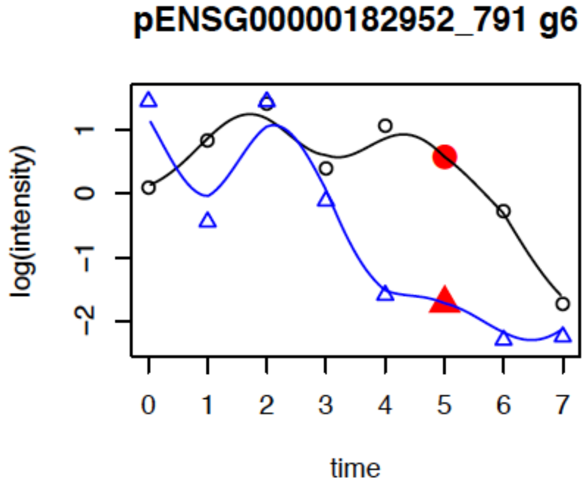

B. PECA analysis of the extended dataset

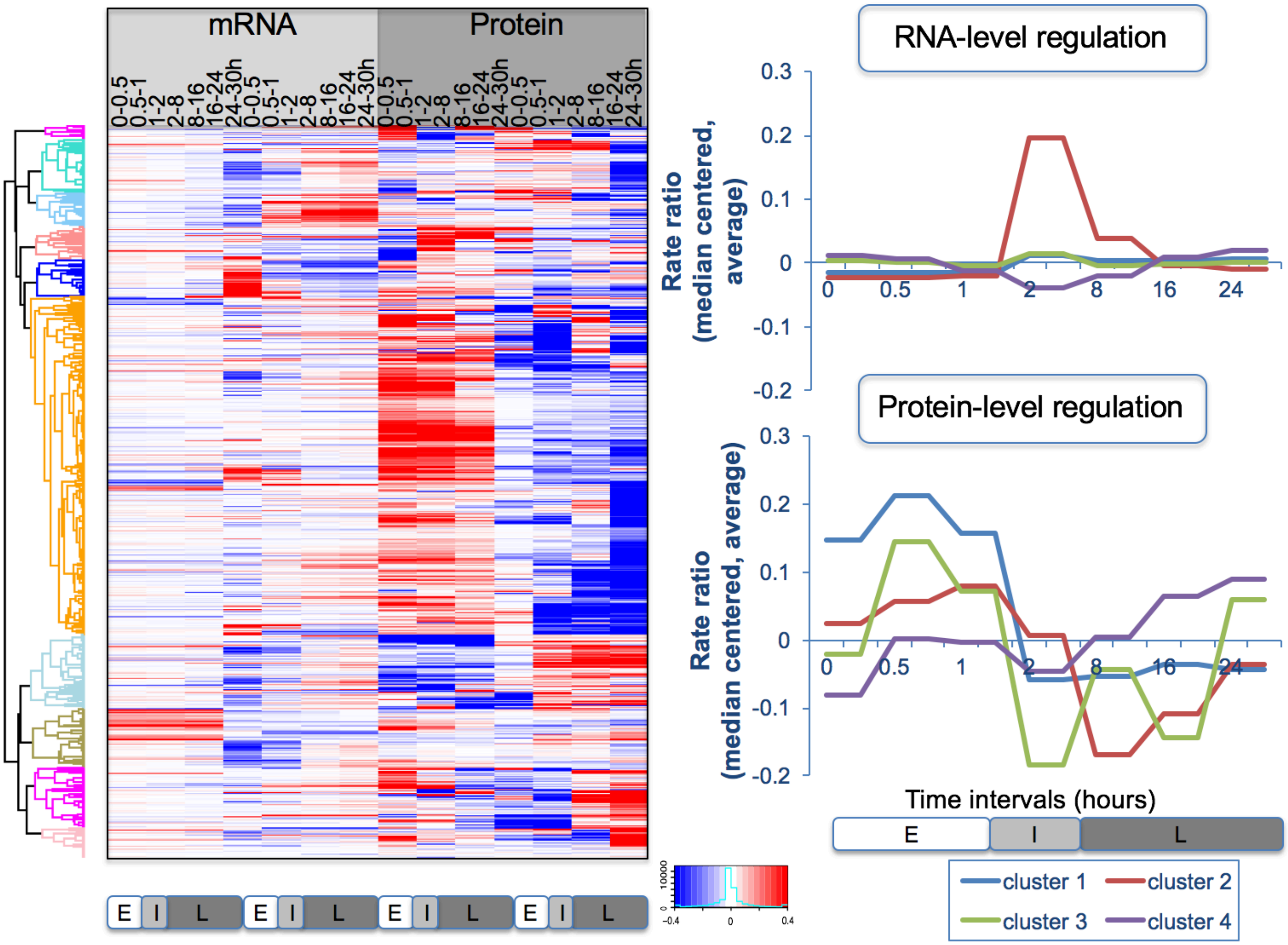

Appendix Figure S20*. *Expression changes across entire transcriptome confirm trends for the core dataset

To confirm the findings presented in the main text that analyze a subset of the mRNA data, we also performed PECA on the entire mRNA data (N>18,000 genes)(**Appendix Figures S20, S21**). We find that all trends hold true across the entire genome. A. The average fold-change (and standard deviation) of normalized, relative mRNA expression values is very small. B. Correlation (Pearson's R^2) between normalized, absolute total RNA expression values of time 0 and the respective time points has its lowest value at 8 hrs, after which mRNA expression returns to original values. RNA-All-1and RNA-All-2 refer to replicate 1 and 2, respectively.

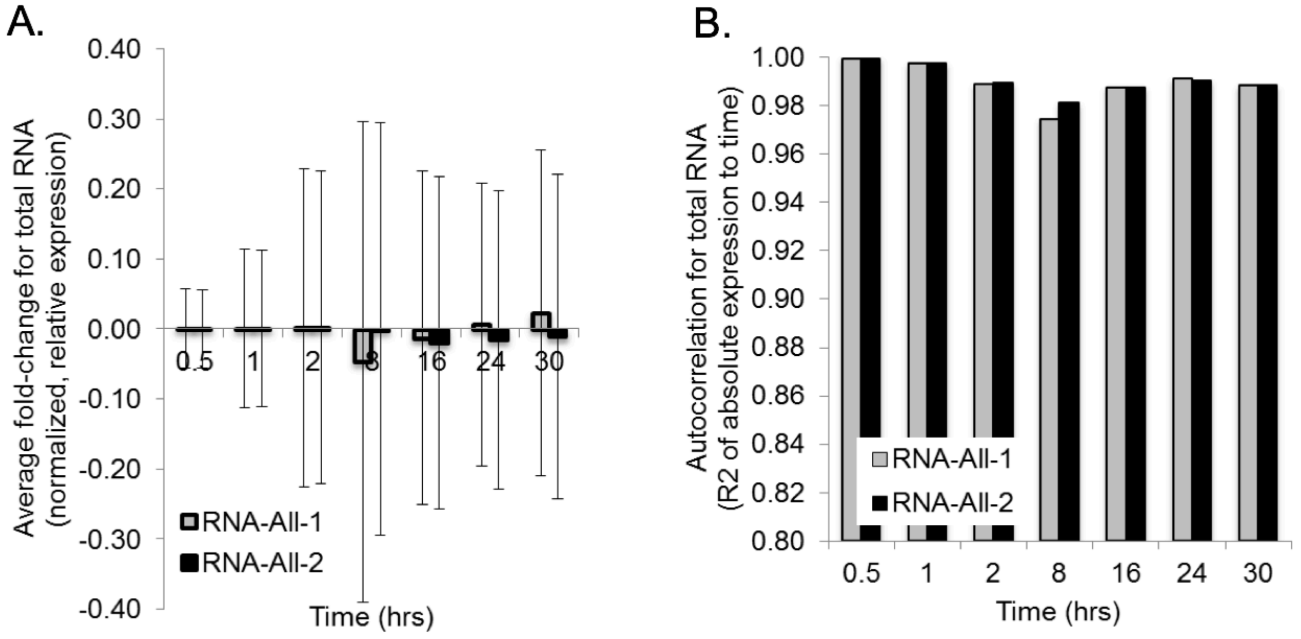

Appendix Figure S21. PECA results for entire RNA data confirm the trends for the core dataset
The PECA analysis of the entire mRNA data confirms the pattern reported in the main text: mRNA expression changes in a 'spike' like fashion in during the 2 to 8hrs interval. A. The heatmaps of the RNA expression data with >18,000 genes (blue-yellow color). B. Rate ratio estimates from PECA (blue-red color), and the CPS scores (white-yellow-red color). The largest rate ratio changes occurred between 2 hours and 8 hours (B, C), while the expression levels at the end of the experiment remained within two-fold for the majority of the genes (A).

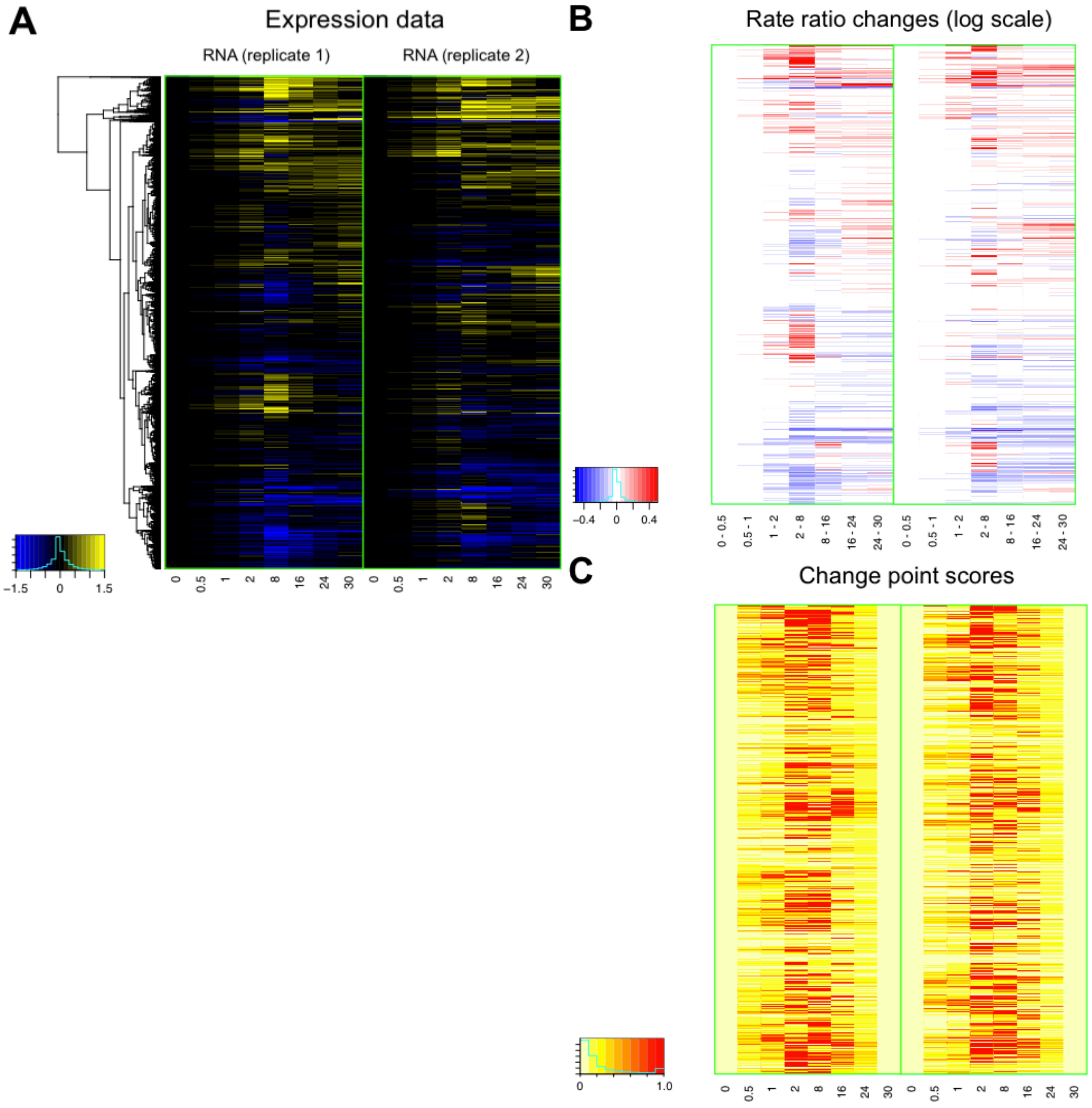

Mammalian sequence features and other characteristics of expression regulation

Appendix Table S2. Sequence and other features involved in protein regulation

To generate hypotheses on possible regulatory pathways that produce the observed mRNA and protein expression patterns, we assembled >160 sequence and other features that have been linked to expression regulation. The Human (GRCg38.p2) sequences were downloaded from Ensembl website. The features are listed in the **Appendix Dataset EV1**, and were tested for significant enrichment across gene clusters.

Features	Rationale	Reference
Nucleotide composition of CDS, 5'UTR and 3'UTR	Sequence composition impacts secondary structure and therefore binding of miRNA/RBPs/ribosomes and RNA stability.	http://www.ensembl.org/index.html and (Branco-Price et al, 2005)
Length of CDS, 5'UTR, 3'UTR	Length of sequence influences translation and protein folding.	http://www.ensembl.org/index.html
RNA secondary structures in CDS, 5', 3'UTR	Secondary structure impacts binding of ribosomes / miRNAs / RBPs and therefore impacts RNA stability and translation	RNA fold energy in Vienna RNA package to predict from sequence (Lorenz et al, 2011)
Sequence motifs in 5', 3' UTR and CDS	Sequence motifs may be binding sites for miRNAs or RBPs that are not described yet, and therefore impacts translation and RNA stability.	AU-rich elements (ARE) in 3'UTR (http://rna.tbi.univie.ac.at/cgi-bin/AREsite.cgi)
uORFs and alternative Translation Initiation Sites (TIS) and IRES	Elements of the 5'UTR affecting translation through binding of ribosomes and other translation proteins.	a) Lee et al (Lee et al, 2012) b) Perl scripts to predict AUG and CUG with or without in frame stop codon in 5'UTR
Motif and targets of microRNAs	miRNAs repress translation	a) TargetScan; b) miTAR -- databases of miRNAs and their targets (Friedman et al, 2009; Garcia et al, 2011; Grimson et al, 2007; Lewis et al, 2005)
Targets of RNA-binding proteins (RBPs)	RBPs regulate translation	a) doRiNA; b) RBPDB; (Cook et al, 2011) c) literature; d) CATRAPID (Agostini et al, 2013); e) TR Hughes (Ray et al, 2013)
Amino acid composition	For example, cysteine rich proteins may be post-translationally modified and be non-observable to mass spectrometry. Enrichment in PEST sequences can serve as protein degradation signals.	Sequences were downloaded from http://www.ensembl.org/index.html and composition was calculated by our own scripts
Protein length	Protein length affects the translation and stability of proteins.	http://www.ensembl.org/index.html
Disordered regions	Proteins with many disordered regions are less stable.	DisoPred prediction from sequence (COILS, HOTLOOPS, and REM465) (Jones & Cozzetto, 2015).
Protein aggregation	Aggregation-prone proteins may form insoluble clusters that cannot be detected by mass spectrometry.	TANGO based prediction based on sequence (Fernandez-Escamilla et al, 2004; Linding et al, 2004; Rousseau et al, 2006)
Sequence motifs	Motifs for post-translational modification, e.g. phosphorylation.	ELM: Eukaryotic Linear Motif resource for Functional Sites in Proteins (Dinkel et al, 2012)

Other protein features	Transcript counts, low complexity regions, transmembrane domains affect translation, MS detection, protein folding and degradation.	Information directly downloaded from the Ensembl database http://www.ensembl.org/index.html
Prokaryote / eukaryote derived genes in human	Prokaryote derived genes are preferred to be translated near mitochondria.	Data from Alvarez-Ponce et al. (Alvarez-Ponce & McInerney, 2011)
Subcellular localization	Localization information for protein is often found in the N or C termini. The subcellular location of a protein is important for its translation, processing, and activity.	TargetP and SignalP to detect localization signals based on protein sequence (Emanuelsson et al, 2007)
Ubiquitination	Ubiquitination is a common degradation and protein re-localization signal.	Assembled dataset for human (collection of public MS datasets on general ubiquitination) (Kim et al, 2011)
SUMOylation	Small Ubiquitin-like Modifiers (SUMOs) are common modifications influencing protein solubility, interactions, and activity.	Collection of SUMO proteins in human: a) based on experiments (Blomster et al, 2009; Blomster et al, 2010; Bruderer et al, 2011; Galisson et al, 2011; Golebiowski et al, 2009; Grant, 2010; Lamoliatte et al, 2013; Manza et al, 2004; Matic et al, 2010; Rosas-Acosta et al, 2005; Schimmel et al, 2008; Tatham et al, 2011; Vertegaal et al, 2006; Vertegaal et al, 2004); b) based on sequence prediction. The human reference proteome was downloaded from European Molecular Biology Laboratory-European Bioinformatics Institute (EMBL-EBI) release 2014_4 (http://www.ebi.ac.uk/reference_proteomes), and the SUMOylation sites were predicted by GPS_SUMO (Ren et al, 2009; Zhao et al, 2014).
Phosphorylation, Acetylation, Methylation, Kinase and kinase substrate	Phosphorylation and other PTMs happen to regulate protein solubility and activity/signal transduction.	PhosphoSitePlus (Hornbeck et al, 2015)

PECA analysis of dendritic cells responding to LPS

In the original publication, PECA was applied to yeast time course datasets (Teo et al, 2014). In this paper, we have further optimized model parameters in PECA for the analysis of mammalian cells responding to ER stress through elicitation of prior distributions allowing for larger noise components. To gain insights into the generality of our findings (see main text), we also used PECA to analyze a published dataset on dendritic cells responding to LPS treatment (Jovanovic et al, 2015).

Appendix Figure S22. Heatmap of expression values and PECA output of dendritic cells responding to LPS treatment

We downloaded Appendix Tables 1 and 2 from the publication by Jovanovic et al. (Jovanovic et al, 2015), containing the modified pulsed SILAC proteomics and RNA-seq transcriptomics data, respectively. For PECA analysis, we extracted 2,332 genes that are present in both RNA and protein data for LPS and MOCK treatment. For the proteomics data, we used the sum of medium-light label ratio and heavy-light ratio (M/L + H/L) as the protein abundance, without separating newly synthesized and existing protein copies, normalizing each biological replicate separately. We then applied PECA with default parameters for RNA- and protein-level analysis for each replicate and treatment. The figure shows the PECA results. A. The heatmaps of the first replicate of LPS stimulation with the RNA and protein expression data (blue-yellow color) on the left, rate ratio estimates from PECA (blue-red color) in the middle, and the CPS scores (white-yellow-red color) on the right. B. The heatmaps of the second LPS stimulation replicate, with the same color settings. Protein regulation is very active upon LPS stimulation in both replicates, as illustrated by largely changing rate ratios between consecutive time points. In the early hours, the stimulation triggers many protein-level concentration changes, and then RNA starts reacting after 2-4 hours. Our result shows that both mRNA and protein levels of regulation are very active, in different ways at early and late time points.

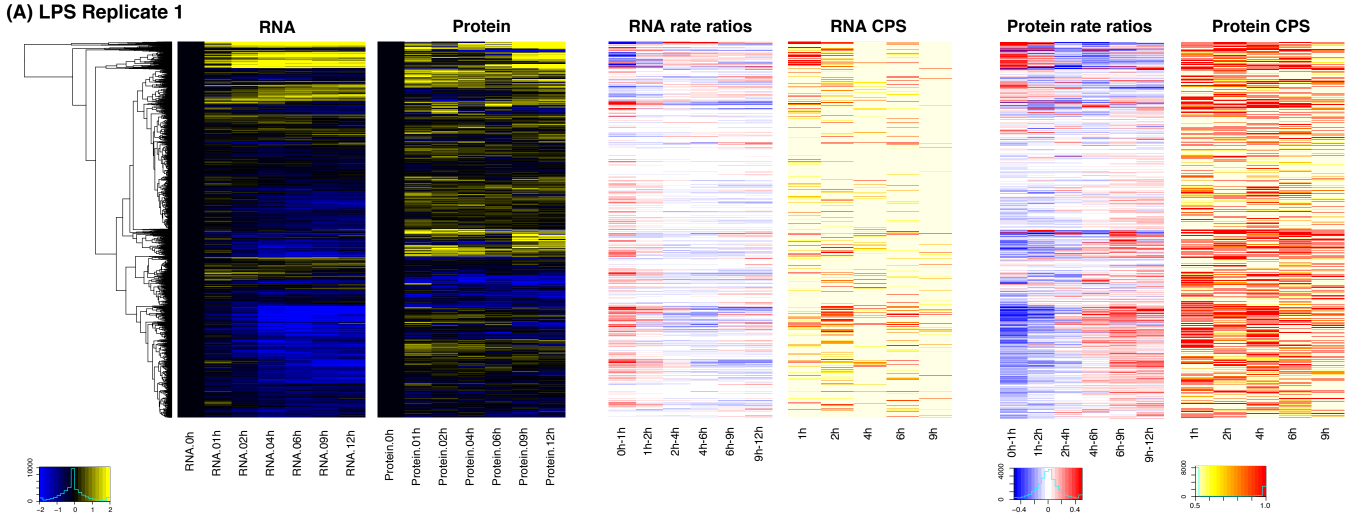

(A) LPS Replicate 1
RNA
Protein
RNA rate ratios
RNA CPS
Protein rate ratios
Protein CPS
RNA.0h
RNA.01h
RNA.02h
RNA.04h
RNA.06h
RNA.09h
RNA.12h
Protein.0h
Protein.01h
Protein.02h
Protein.04h
Protein.06h
Protein.09h
Protein.12h
0h-1h
1h-2h
2h-4h
4h-6h
6h-9h
9h-12h
1h
2h
4h
6h
9h

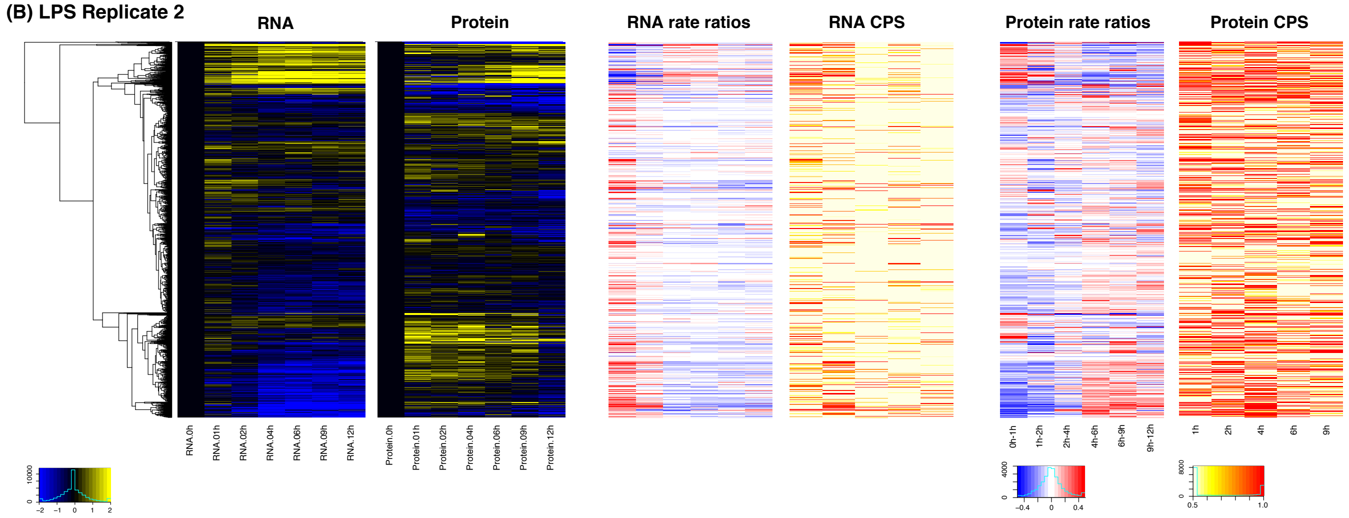

(B) LPS Replicate 2
RNA
Protein
RNA rate ratios
RNA CPS
Protein rate ratios
Protein CPS

Appendix Table S3. Significance cutoffs - Jovanovic et al. (Science 2015)

The table lists the CPS thresholds associated with 1% FDR for each analysis of the data from Jovanovic et al. (Science 2015). mRNAs and proteins with a PECA CPS above this threshold were considered changing significantly in their temporal expression. The estimated rate ratios were highly consistent between the two replicates with LPS stimulation. The RNA rate ratios and protein rate ratios had median correlation between replicates 0.819 and 0.616 respectively.

	RNA	Protein
LPS, Replicate 1	0.961	0.935
LPS, Replicate 2	0.962	0.935
MOCK, Replicate 1	0.955	0.941
MOCK, Replicate 2	0.946	0.933

Appendix Table S4. Significantly regulated genes identified in data published by Jovanovic et al. (Science 2015)

As reported in the table, many PECA-defined regulatory events were detected at the protein level even though the concentrations changed much more in the RNA data. There are two caveats with the PECA analysis of this dataset. First, the PECA model could have underestimated regulation in the RNA data since the fluctuation in the concentrations changed much more in the RNA data than in the protein data, and the hierarchical Bayes model in PECA penalizes concentration variation with mild fold changes (e.g. 50% changes) during the process of borrowing statistical information across proteins. Second, protein concentrations were estimated from the sum of two ratio data (M/L + H/L), which has its faults.

Despite these drawbacks, the regulation landscape we observe shows some general principles that cast additional insights to the interpretation of the data beyond what we discuss in the main text. Although it is clear that, in agreement with the authors' interpretation, much of the protein-level concentration changes are driven by RNA concentration changes, the overall protein-level concentration seems to remain stable and much of the RNA-level changes seems to have no effect on many proteins. This resistance of proteins to mRNA changes implies that either there is an elaborate protein-level regulation via tuning of translation and protein degradation, or that newly synthesized transcripts simply have limited access to translational machinery in general.

Not – not significant. Sig. – significant regulation according to cutoffs presented in **Appendix Table S3**.

			1h		2h		4h		6h		9h	
			Protein									
			Not	Sig	Not	Sig	Not	Sig	Not	Sig	Not	Sig
LPS Replicate 1	RNA	Not	1635	592	1734	495	1696	606	1807	475	2012	302
		Sig	48	57	77	26	16	14	35	15	15	3
LPS Replicate 2		Not	1627	520	1627	578	1677	625	1897	374	1822	463
		Sig	68	117	82	45	15	15	49	12	31	16

Appendix Figure 23. The LPS response shows switch-like regulation at the RNA level

To assess the generality of our findings, we performed an analysis similar to that of **Figure 5** in the main text: after grouping the normalized and centered rate ratios for the LPS experiment into six clusters (left), we calculated the average rate ratios per cluster (right). The average profiles are different to those detected in the ER stress response: mRNA-level regulation shows a switch-like behavior, while protein-level regulation shows mixed patterns, both switch- and spike-like behavior. From these two different studies, we conclude that the *dominant* regulatory level, i.e. transcription in the case of LPS treatment, and translation/protein degradation in the case of the ER stress response might show a switch-like behavior that leads to establishment of a new steady state. In comparison, the minor regulatory level – translation in the case of LPS response and transcription for the ER stress response – shows a mix of patterns which include spikes. Future investigation of additional conditions will show if the interpretation is true.

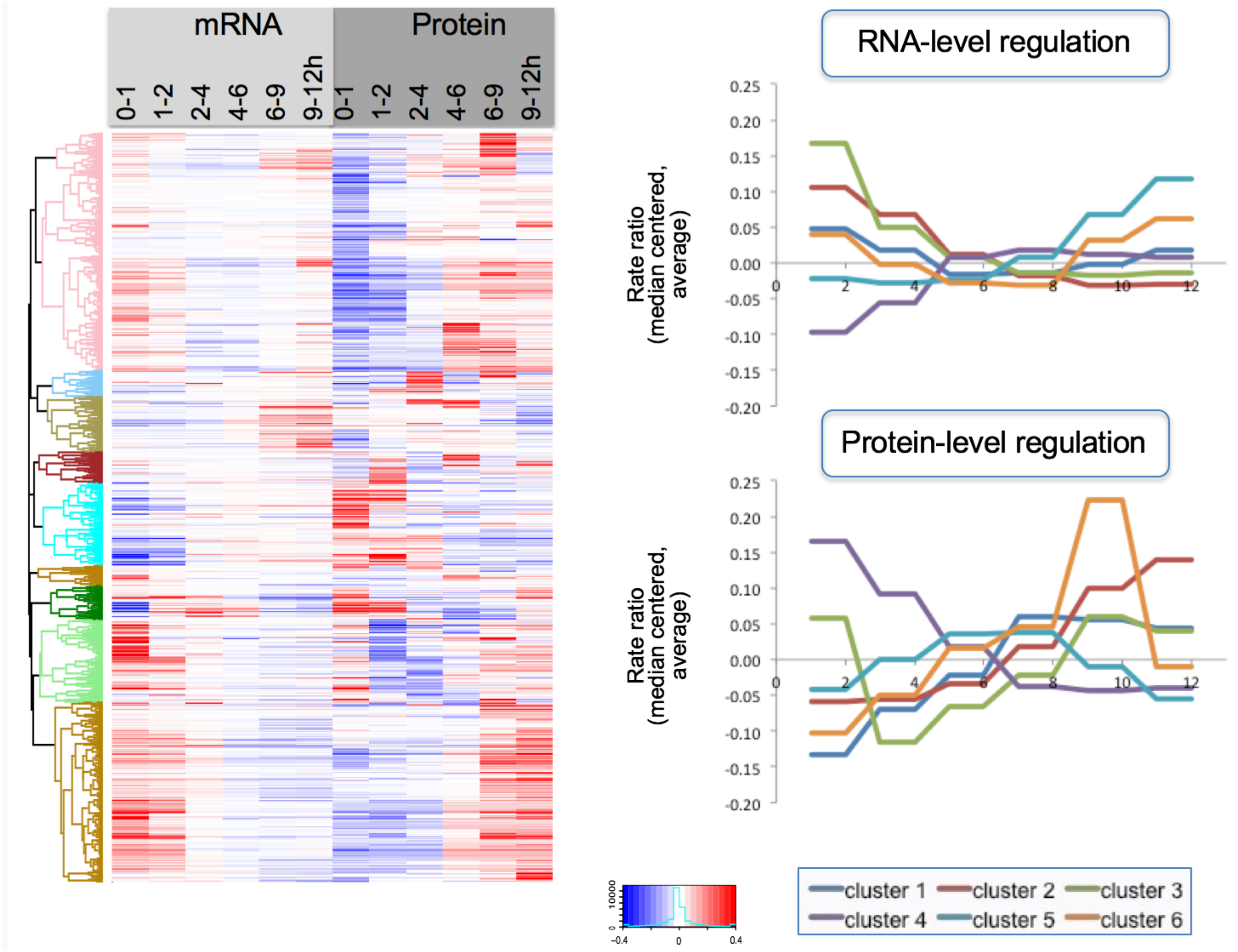

References

Agostini F, Zanzoni A, Klus P, Marchese D, Cirillo D, Tartaglia GG (2013) catRAPID omics: a web server for large-scale prediction of protein-RNA interactions. *Bioinformatics* **29:** 2928-2930

Alvarez-Ponce D, McInerney JO (2011) The Human Genome Retains Relics of Its Prokaryotic Ancestry: Human Genes of Archaebacterial and Eubacterial Origin Exhibit Remarkable Differences. *Genome Biol Evol* **3:** 782-790

Blomster HA, Hietakangas V, Wu JM, Kouvonen P, Hautaniemi S, Sistonen L (2009) Novel Proteomics Strategy Brings Insight into the Prevalence of SUMO-2 Target Sites. *Molecular & Cellular Proteomics* **8:** 1382-1390

Blomster HA, Imanishi SY, Siimes J, Kastu J, Morrice NA, Eriksson JE, Sistonen L (2010) In vivo identification of sumoylation sites by a signature tag and cysteine-targeted affinity purification. *The Journal of biological chemistry* **285:** 19324-19329

Branco-Price C, Kawaguchi R, Ferreira RB, Bailey-Serres J (2005) Genome-wide analysis of transcript abundance and translation in Arabidopsis seedlings subjected to oxygen deprivation. *Annals of botany* **96:** 647-660

Bruderer R, Tatham MH, Plechanovova A, Matic I, Garg AK, Hay RT (2011) Purification and identification of endogenous polySUMO conjugates. *EMBO reports* **12:** 142-148

Chen SJ, Wu YH, Huang HY, Wang CC (2012) Saccharomyces cerevisiae possesses a stress-inducible glycyl-tRNA synthetase gene. *PloS one* **7:** e33363

Cook KB, Kazan H, Zuberi K, Morris Q, Hughes TR (2011) RBPDB: a database of RNA-binding specificities. *Nucleic acids research* **39:** D301-D308

Cox J, Mann M (2008) MaxQuant enables high peptide identification rates, individualized p.p.b.-range mass accuracies and proteome-wide protein quantification. *Nature biotechnology* **26:** 1367-1372

Dinkel H, Michael S, Weatheritt RJ, Davey NE, Van Roey K, Altenberg B, Toedt G, Uyar B, Seiler M, Budd A, Jodicke L, Dammert MA, Schroeter C, Hammer M, Schmidt T, Jehl P, McGuigan C, Dymecka M, Chica C, Luck K et al (2012) ELM--the database of eukaryotic linear motifs. *Nucleic acids research* **40:** D242-251

Emanuelsson O, Brunak S, von Heijne G, Nielsen H (2007) Locating proteins in the cell using TargetP, SignalP and related tools. *Nat Protoc* **2:** 953-971

Fernandez-Escamilla AM, Rousseau F, Schymkowitz J, Serrano L (2004) Prediction of sequence-dependent and mutational effects on the aggregation of peptides and proteins. *Nature biotechnology* **22:** 1302-1306

Fernandez J, Yaman I, Sarnow P, Snider MD, Hatzoglou M (2002) Regulation of internal ribosomal entry site-mediated translation by phosphorylation of the translation initiation factor eIF2alpha. *The Journal of biological chemistry* **277:** 19198-19205

Friedman RC, Farh KKH, Burge CB, Bartel DP (2009) Most mammalian mRNAs are conserved targets of microRNAs. *Genome Res* **19:** 92-105

Galisson F, Mahrouche L, Courcelles M, Bonneil E, Meloche S, Chelbi-Alix MK, Thibault P (2011) A Novel Proteomics Approach to Identify SUMOylated Proteins and Their Modification Sites in Human Cells. *Molecular & Cellular Proteomics* **10**

Garcia DM, Baek D, Shin C, Bell GW, Grimson A, Bartel DP (2011) Weak seed-pairing stability and high target-site abundance decrease the proficiency of lsy-6 and other microRNAs. *Nat Struct Mol Biol* **18:** 1139-U1175

Golebiowski F, Matic I, Tatham MH, Cole C, Yin Y, Nakamura A, Cox J, Barton GJ, Mann M, Hay RT (2009) System-wide changes to SUMO modifications in response to heat shock. *Science signaling* **2:** ra24

Grant MM (2010) Identification of SUMOylated proteins in neuroblastoma cells after treatment with hydrogen peroxide or ascorbate. *BMB reports* **43:** 720-725

Grimson A, Farh KKH, Johnston WK, Garrett-Engele P, Lim LP, Bartel DP (2007) MicroRNA targeting specificity in mammals: Determinants beyond seed pairing. *Molecular cell* **27:** 91-105

Guan BJ, Krokowski D, Majumder M, Schmotzer CL, Kimball SR, Merrick WC, Koromilas AE, Hatzoglou M (2014) Translational control during endoplasmic reticulum stress beyond phosphorylation of the translation initiation factor eIF2alpha. *The Journal of biological chemistry* **289:** 12593-12611

Hebenstreit D, Fang M, Gu M, Charoensawan V, van Oudenaarden A, Teichmann SA (2011) RNA sequencing reveals two major classes of gene expression levels in metazoan cells. *Molecular systems biology* **7:** 497

Hornbeck PV, Zhang B, Murray B, Kornhauser JM, Latham V, Skrzypek E (2015) PhosphoSitePlus, 2014: mutations, PTMs and recalibrations. *Nucleic acids research* **43:** D512-520

Huang DW, Sherman BT, Lempicki RA (2009a) Bioinformatics enrichment tools: paths toward the comprehensive functional analysis of large gene lists. *Nucleic acids research* **37:** 1-13

Huang DW, Sherman BT, Lempicki RA (2009b) Systematic and integrative analysis of large gene lists using DAVID bioinformatics resources. *Nat Protoc* **4:** 44-57

Jones DT, Cozzetto D (2015) DISOPRED3: precise disordered region predictions with annotated protein-binding activity. *Bioinformatics* **31:** 857-863

Jovanovic M, Rooney MS, Mertins P, Przybylski D, Chevrier N, Satija R, Rodriguez EH, Fields AP, Schwartz S, Raychowdhury R, Mumbach MR, Eisenhaure T, Rabani M, Gennert D, Lu D, Delorey T, Weissman JS, Carr SA, Hacohen N, Regev A (2015) Immunogenetics. Dynamic profiling of the protein life cycle in response to pathogens. *Science* **347:** 1259038

Kim W, Bennett EJ, Huttlin EL, Guo A, Li J, Possemato A, Sowa ME, Rad R, Rush J, Comb MJ, Harper JW, Gygi SP (2011) Systematic and quantitative assessment of the ubiquitin-modified proteome. *Molecular cell* **44:** 325-340

Kwon NH, Kang T, Lee JY, Kim HH, Kim HR, Hong J, Oh YS, Han JM, Ku MJ, Lee SY, Kim S (2011) Dual role of methionyl-tRNA synthetase in the regulation of translation and tumor suppressor activity of aminoacyl-tRNA synthetase-interacting multifunctional protein-3. *Proceedings of the National Academy of Sciences of the United States of America* **108:** 19635-19640

Lamoliatte F, Bonneil E, Durette C, Caron-Lizotte O, Wildemann D, Zerweck J, Wenshuk H, Thibault P (2013) Targeted Identification of SUMOylation Sites in Human Proteins Using Affinity Enrichment and Paralog-specific Reporter Ions. *Molecular & Cellular Proteomics* **12:** 2536-2550

Lee S, Liu B, Lee S, Huang SX, Shen B, Qian SB (2012) Global mapping of translation initiation sites in mammalian cells at single-nucleotide resolution. *Proceedings of the National Academy of Sciences of the United States of America* **109:** E2424-2432

Lewis BP, Burge CB, Bartel DP (2005) Conserved seed pairing, often flanked by adenosines, indicates that thousands of human genes are microRNA targets. *Cell* **120:** 15-20

Linding R, Schymkowitz J, Rousseau F, Diella F, Serrano L (2004) A comparative study of the relationship between protein structure and beta-aggregation in globular and intrinsically disordered proteins. *J Mol Biol* **342:** 345-353

Lo WS, Gardiner E, Xu Z, Lau CF, Wang F, Zhou JJ, Mendlein JD, Nangle LA, Chiang KP, Yang XL, Au KF, Wong WH, Guo M, Zhang M, Schimmel P (2014) Human tRNA synthetase catalytic nulls with diverse functions. *Science* **345:** 328-332

Lorenz R, Bernhart SH, Honer Zu Siederdissen C, Tafer H, Flamm C, Stadler PF, Hofacker IL (2011) ViennaRNA Package 2.0. *Algorithms for molecular biology : AMB* **6:** 26

Manza LL, Codreanu SG, Stamer SL, Smith DL, Wells KS, Roberts RL, Liebler DC (2004) Global shifts in protein sumoylation in response to electrophile and oxidative stress. *Chemical research in toxicology* **17:** 1706-1715

Matic I, Schimmel J, Hendriks IA, van Santen MA, van de Rijke F, van Dam H, Gnad F, Mann M, Vertegaal ACO (2010) Site-Specific Identification of SUMO-2 Targets in Cells Reveals an Inverted SUMOylation Motif and a Hydrophobic Cluster SUMOylation Motif. *Molecular cell* **39:** 641-652

Park MC, Kang T, Jin D, Han JM, Kim SB, Park YJ, Cho K, Park YW, Guo M, He W, Yang XL, Schimmel P, Kim S (2012) Secreted human glycyl-tRNA synthetase implicated in defense against ERK-activated tumorigenesis. *Proceedings of the National Academy of Sciences of the United States of America* **109:** E640-647

Rasmussen C, Williams C. Gaussian Processes for Machine Learning. *The MIT Press*, 2006.

Ray D, Kazan H, Cook KB, Weirauch MT, Najafabadi HS, Li X, Gueroussov S, Albu M, Zheng H, Yang A, Na H, Irimia M, Matzat LH, Dale RK, Smith SA, Yarosh CA, Kelly SM, Nabet B, Mecenas D, Li WM et al (2013) A compendium of RNA-binding motifs for decoding gene regulation. *Nature* **499:** 172-177

Ren J, Gao XJ, Jin CJ, Zhu M, Wang XW, Shaw A, Wen LP, Yao XB, Xue Y (2009) Systematic study of protein sumoylation: Development of a site-specific predictor of SUMOsp 2.0. *Proteomics* **9:** 3409-3412

Roobol A, Roobol J, Bastide A, Knight JR, Willis AE, Smales CM (2015) p58IPK is an inhibitor of the eIF2alpha kinase GCN2 and its localization and expression underpin protein synthesis and ER processing capacity. *The Biochemical journal* **465:** 213-225

Rosas-Acosta G, Russell WK, Deyrieux A, Russell DH, Wilson VG (2005) A universal strategy for proteomic studies of SUMO and other ubiquitin-like modifiers. *Molecular & Cellular Proteomics* **4:** 56-72

Rousseau F, Schymkowitz J, Serrano L (2006) Protein aggregation and amyloidosis: confusion of the kinds? *Current opinion in structural biology* **16:** 118-126

Schimmel J, Larsen KM, Matic I, van Hagen M, Cox J, Mann M, Andersen JS, Vertegaal ACO (2008) The Ubiquitin-Proteasome System Is a Key Component of the SUMO-2/3 Cycle. *Molecular & Cellular Proteomics* **7:** 2107-2122

Tatham MH, Matic I, Mann M, Hay RT (2011) Comparative Proteomic Analysis Identifies a Role for SUMO in Protein Quality Control. *Science signaling* **4**

Teo G, Vogel C, Ghosh D, Kim S, Choi H (2014) PECA: a novel statistical tool for deconvoluting time-dependent gene expression regulation. *Journal of proteome research* **13:** 29-37

Vertegaal ACO, Andersen JS, Ogg SC, Hay RT, Mann M, Lamond AI (2006) Distinct and overlapping sets of SUMO-1 and SUMO-2 target proteins revealed by quantitative proteomics. *Molecular & Cellular Proteomics* **5:** 2298-2310

Vertegaal ACO, Ogg SC, Jaffray E, Rodriguez MS, Hay RT, Andersen JS, Mann M, Lamond AI (2004) A proteomic study of SUMO-2 target proteins. *Journal of Biological Chemistry* **279:** 33791-33798

Wei N, Shi Y, Truong LN, Fisch KM, Xu T, Gardiner E, Fu G, Hsu YS, Kishi S, Su AI, Wu X, Yang XL (2014) Oxidative stress diverts tRNA synthetase to nucleus for protection against DNA damage. *Molecular cell* **56:** 323-332

Yan W, Frank CL, Korth MJ, Sopher BL, Novoa I, Ron D, Katze MG (2002) Control of PERK eIF2alpha kinase activity by the endoplasmic reticulum stress-induced molecular chaperone P58IPK. *Proceedings of the National Academy of Sciences of the United States of America* **99:** 15920-15925

Zhang L, Fok JJ, Mirabella F, Aronson LI, Fryer RA, Workman P, Morgan GJ, Davies FE (2013) Hsp70 inhibition induces myeloma cell death via the intracellular accumulation of immunoglobulin and the generation of proteotoxic stress. *Cancer letters* **339:** 49-59

Zhao Q, Xie Y, Zheng Y, Jiang S, Liu W, Mu W, Liu Z, Zhao Y, Xue Y, Ren J (2014) GPS-SUMO: a tool for the prediction of sumoylation sites and SUMO-interaction motifs. *Nucleic acids research* **42:** W325-330
