# Supplementary material for: Differential dynamics of the mammalian mRNA and protein expression response to misfolding stress
Source: Mol Syst Biol. 2016 Jan 20;12(1):855. doi: 10.15252/msb.20156423 (PMC4731011; doi:10.15252/msb.20156423)
Supplement: Supplementary file 5 — Dataset EV4 [file MSB-12-855-s005.zip › Dataset_EV4/Cheng_Dataset_EV4_norm_filtered_lowess_data_geneplot.pdf]

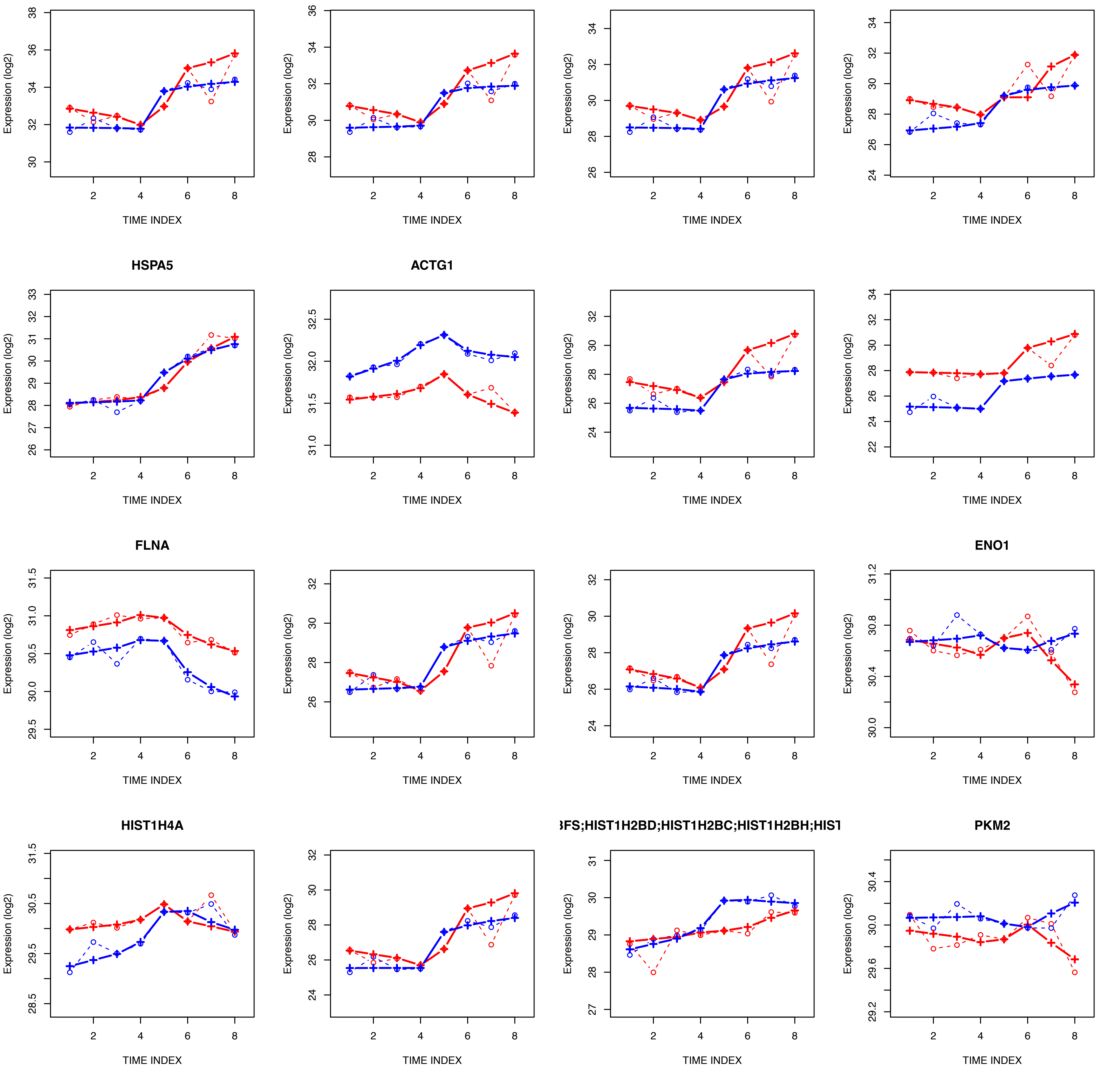
HSPA5
ACTG1
FLNA
ENO1
HIST1H4A
3FS;HIST1H2BD;HIST1H2BC;HIST1H2BH;HIST
PKM2
Expression (log2)
TIME INDEX

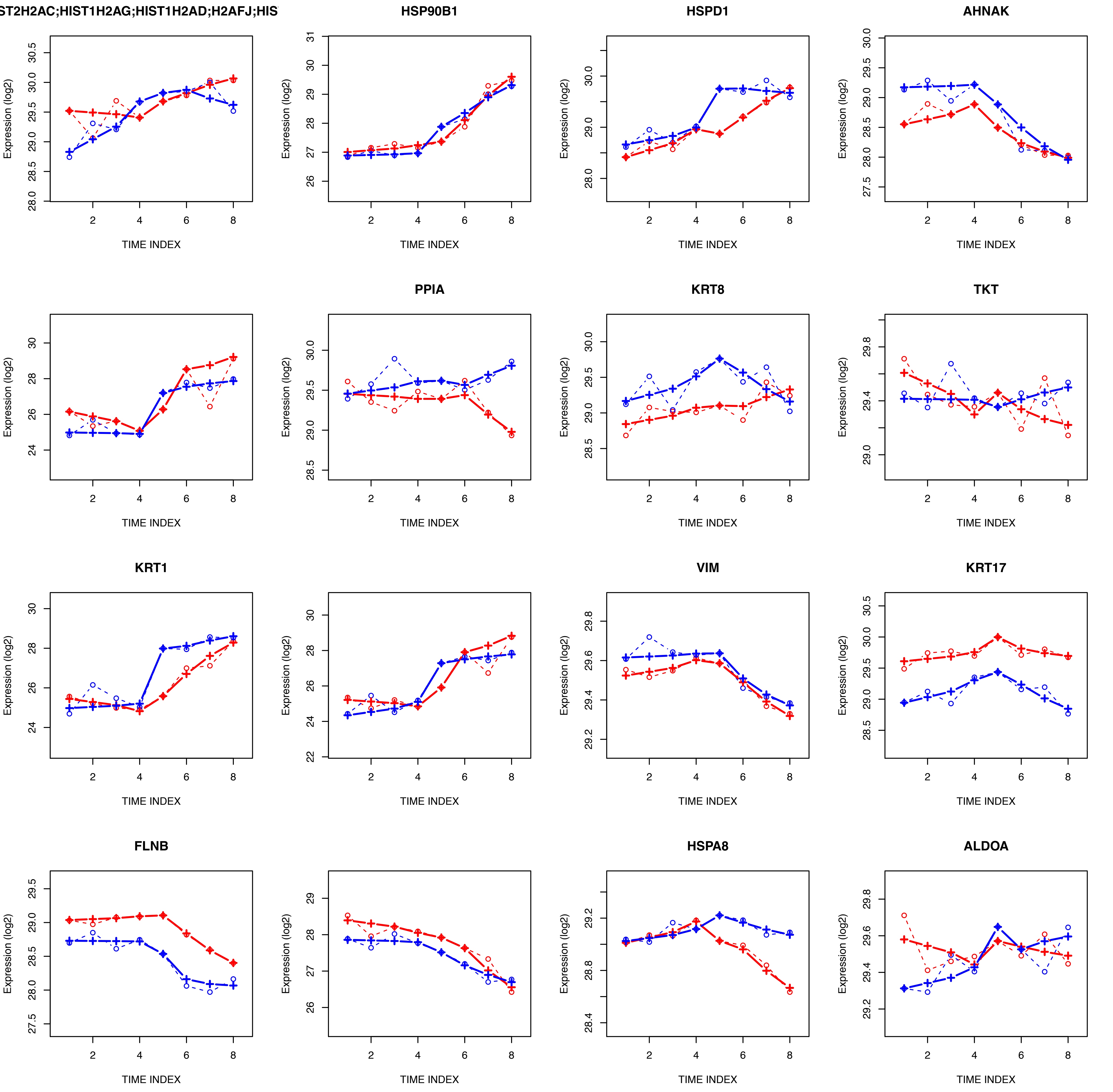
ST2H2AC;HIST1H2AG;HIST1H2AD;H2AFJ;HIS
HSP90B1
HSPD1
AHNAK
PPIA
KRT8
TKT
KRT1
VIM
KRT17
FLNB
HSPA8
ALDOA
Expression (log2)
TIME INDEX

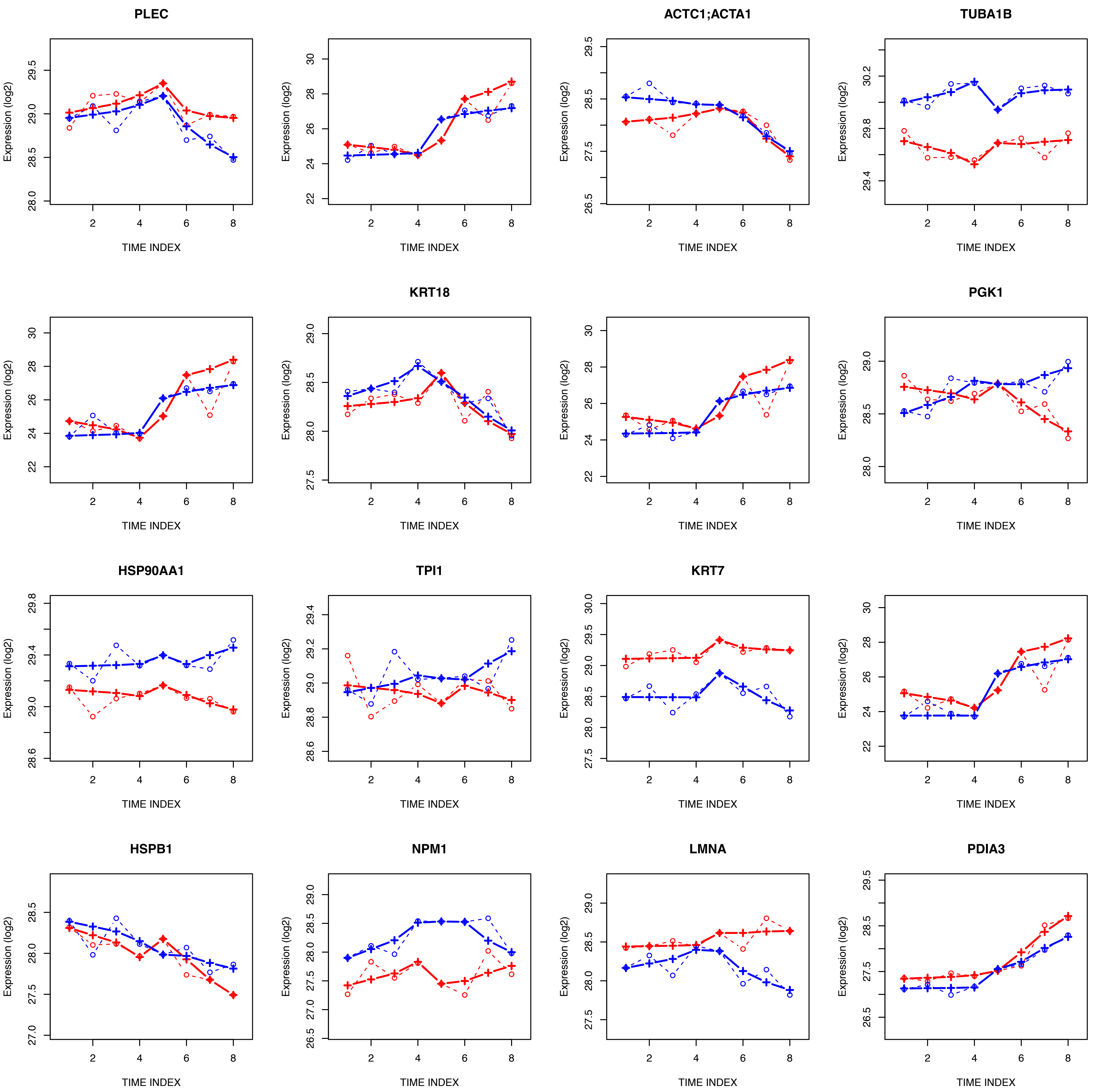

PLEC
ACTC1;ACTA1
TUBA1B
KRT18
PGK1
HSP90AA1
TPI1
KRT7
HSPB1
NPM1
LMNA
PDIA3
Expression (log2)
TIME INDEX

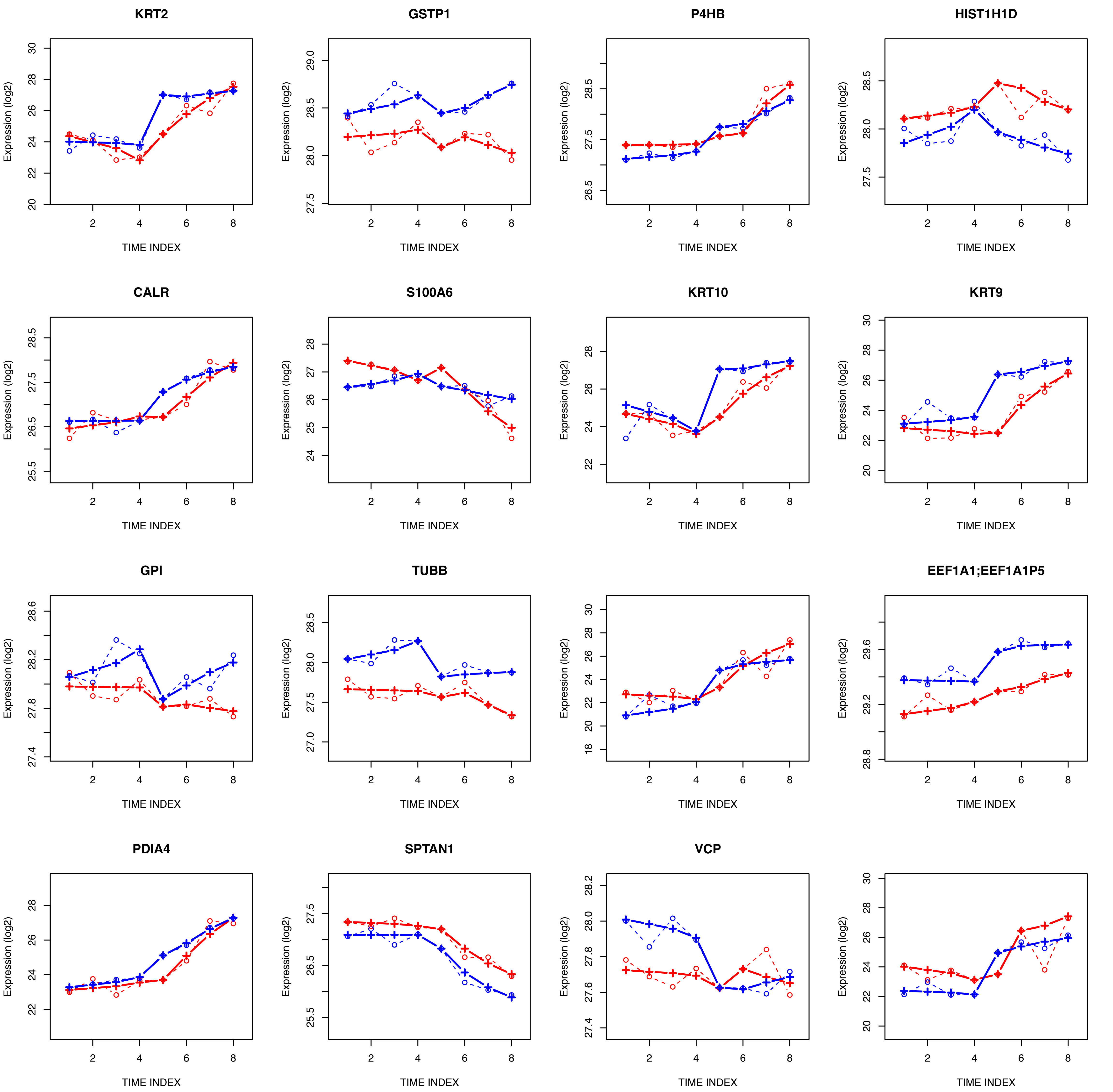
KRT2
GSTP1
P4HB
HIST1H1D
CALR
S100A6
KRT10
KRT9
GPI
TUBB
EEF1A1;EEF1A1P5
PDIA4
SPTAN1
VCP
Expression (log2)
TIME INDEX

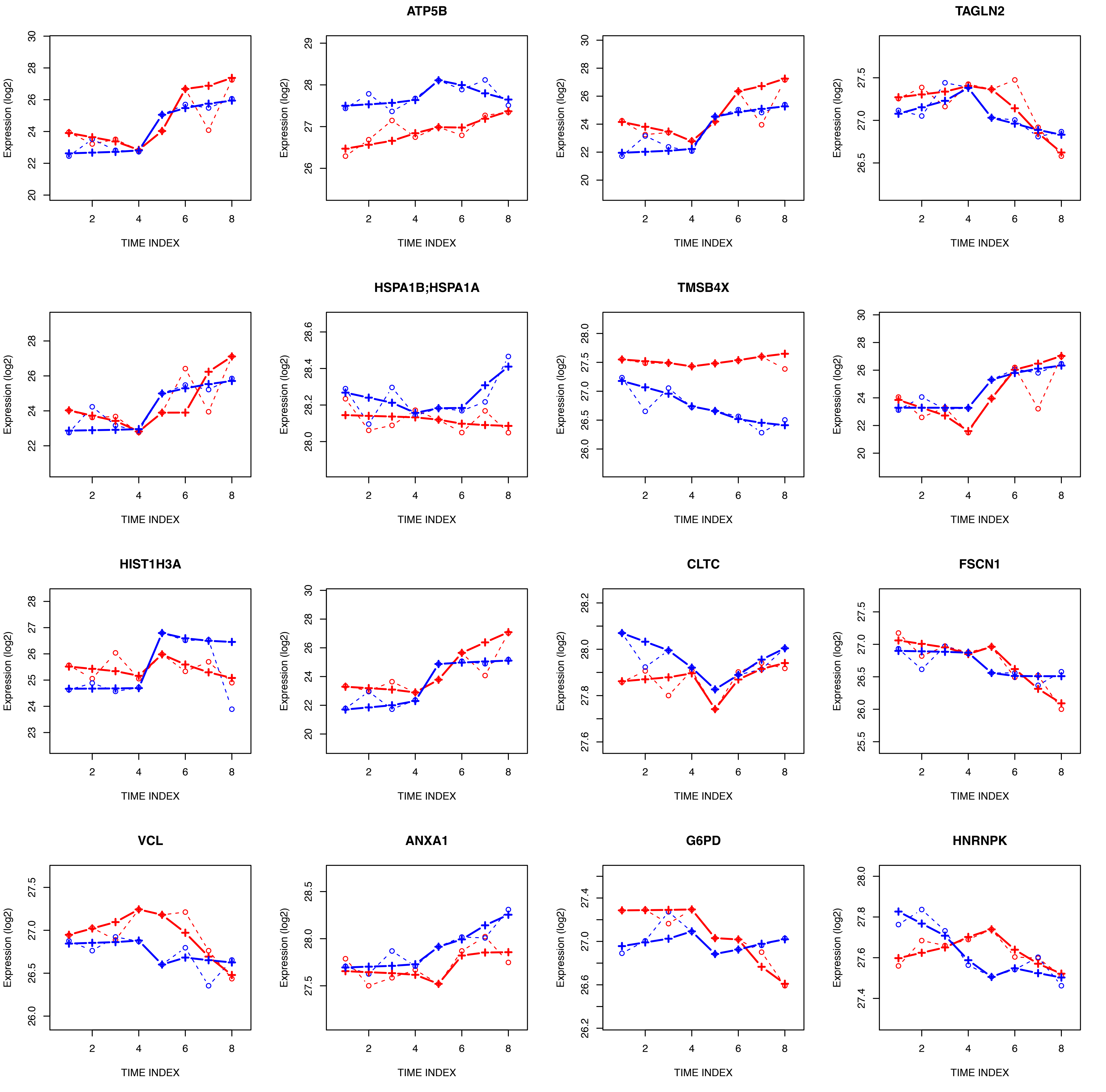
ATP5B
TAGLN2
HSPA1B;HSPA1A
TMSB4X
HIST1H3A
CLTC
FSCN1
VCL
ANXA1
G6PD
HNRNPK
Expression (log2)
TIME INDEX

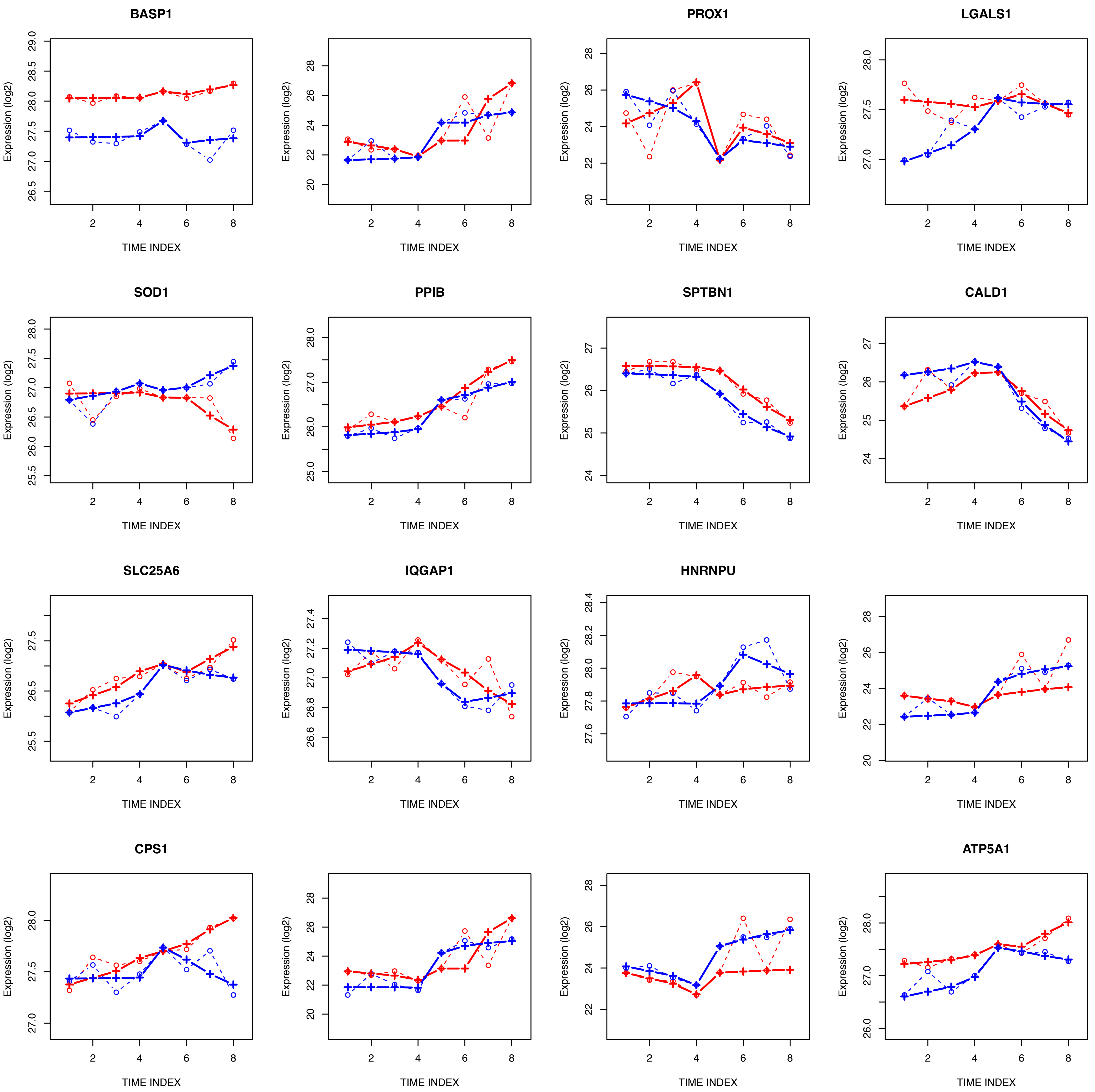
BASP1
PROX1
LGALS1
SOD1
PPIB
SPTBN1
CALD1
SLC25A6
IQGAP1
HNRNPU
CPS1
ATP5A1
Expression (log2)
TIME INDEX

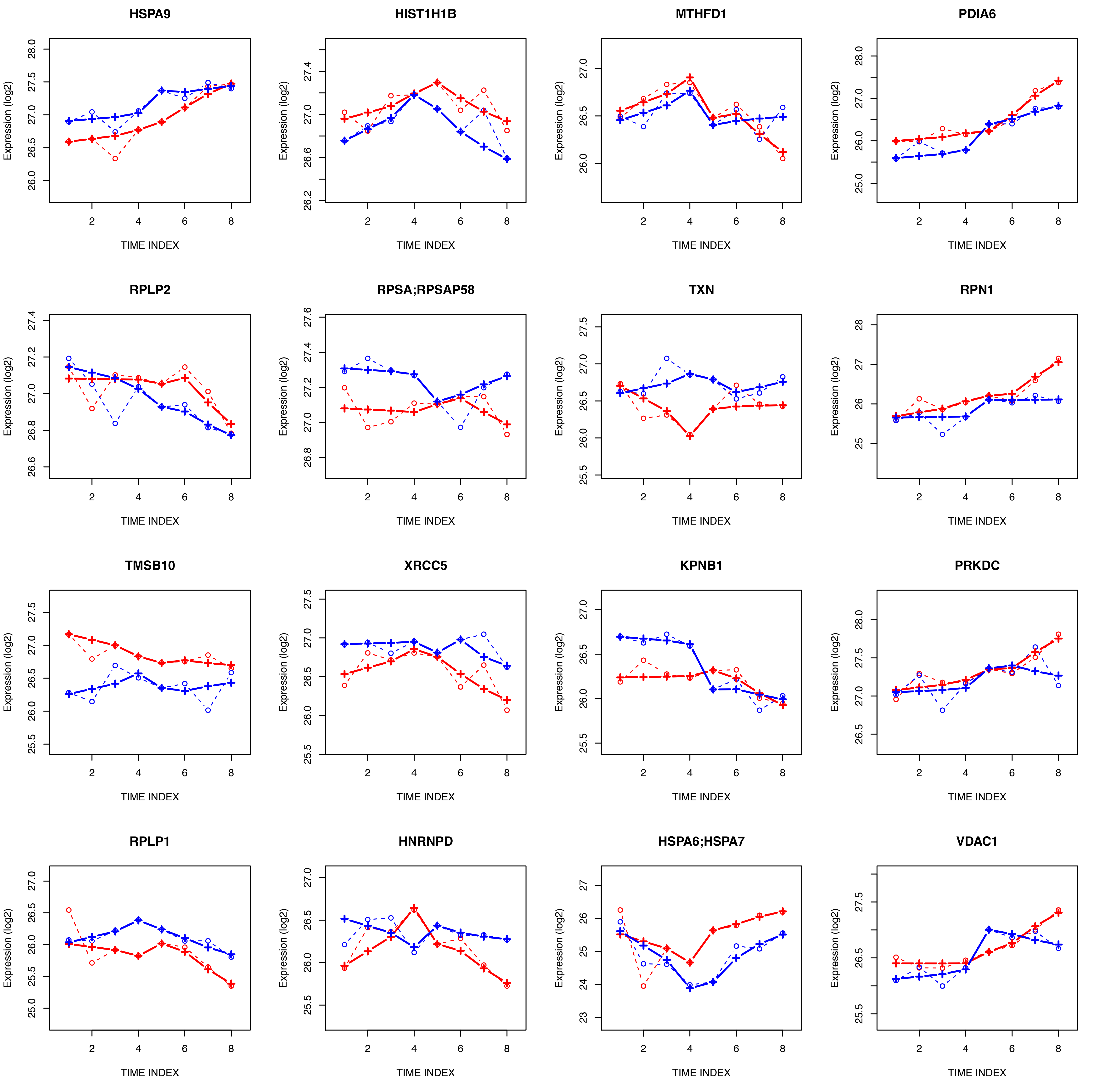
HSPA9
HIST1H1B
MTHFD1
PDIA6
RPLP2
RPSA;RPSAP58
TXN
RPN1
TMSB10
XRCC5
KPNB1
PRKDC
RPLP1
HNRNPD
HSPA6;HSPA7
VDAC1
Expression (log2)
TIME INDEX

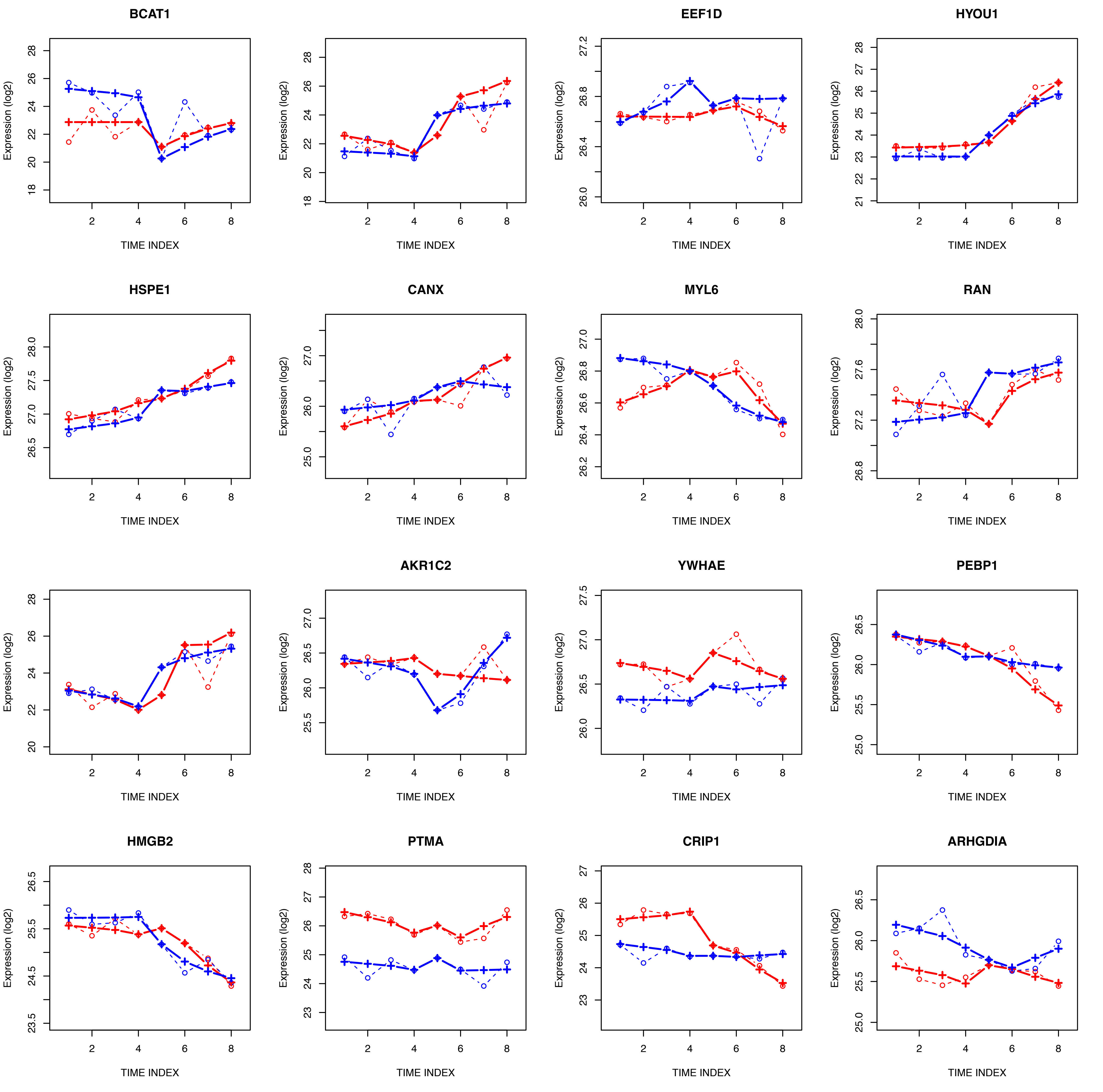
BCAT1
EEF1D
HYOU1
HSPE1
CANX
MYL6
RAN
AKR1C2
YWHAE
PEBP1
HMGB2
PTMA
CRIP1
ARHGDIA
Expression (log2)
TIME INDEX

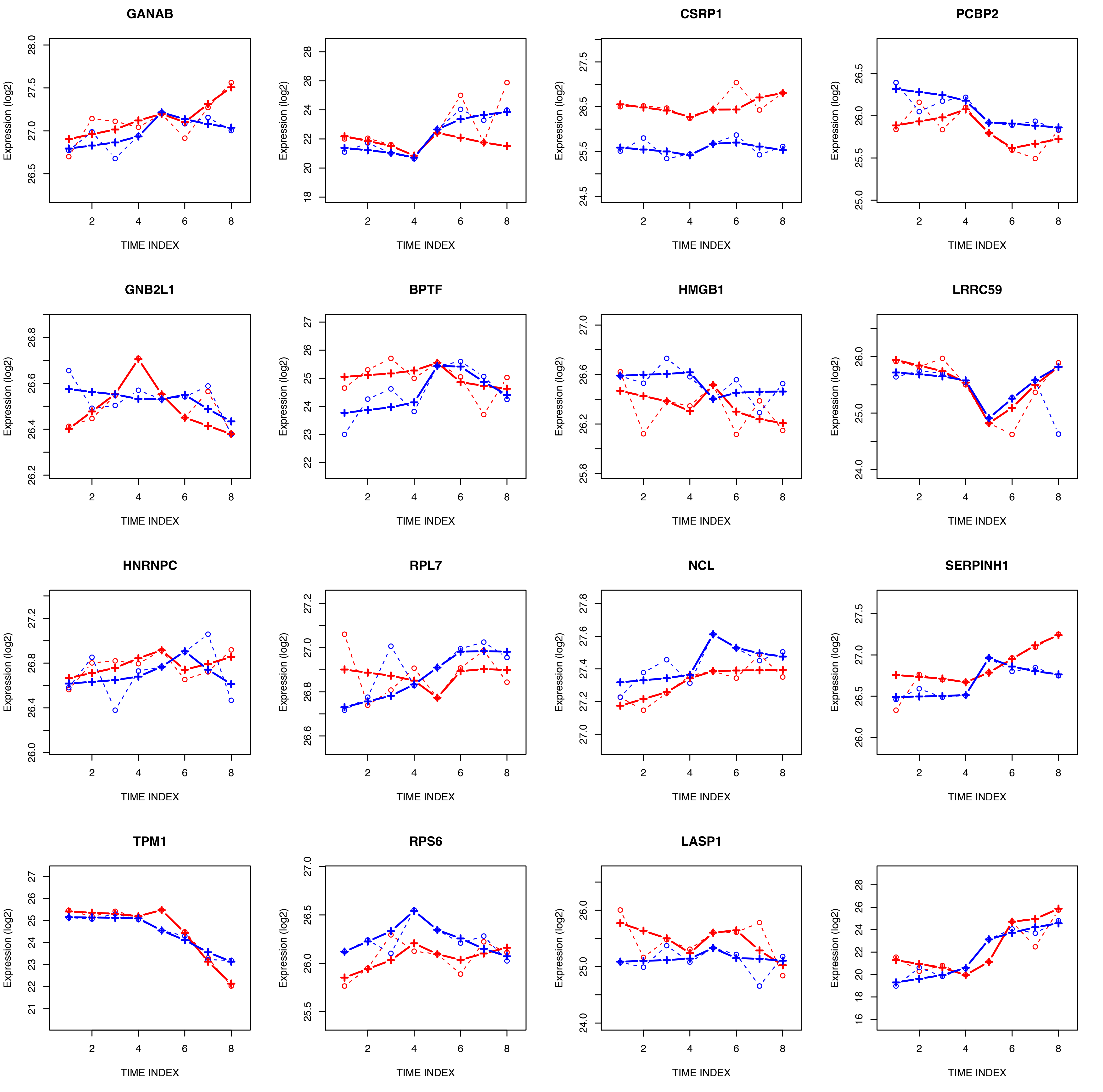
GANAB
CSRP1
PCBP2
GNB2L1
BPTF
HMGB1
LRRC59
HNRNPC
RPL7
NCL
SERPINH1
TPM1
RPS6
LASP1
Expression (log2)
TIME INDEX

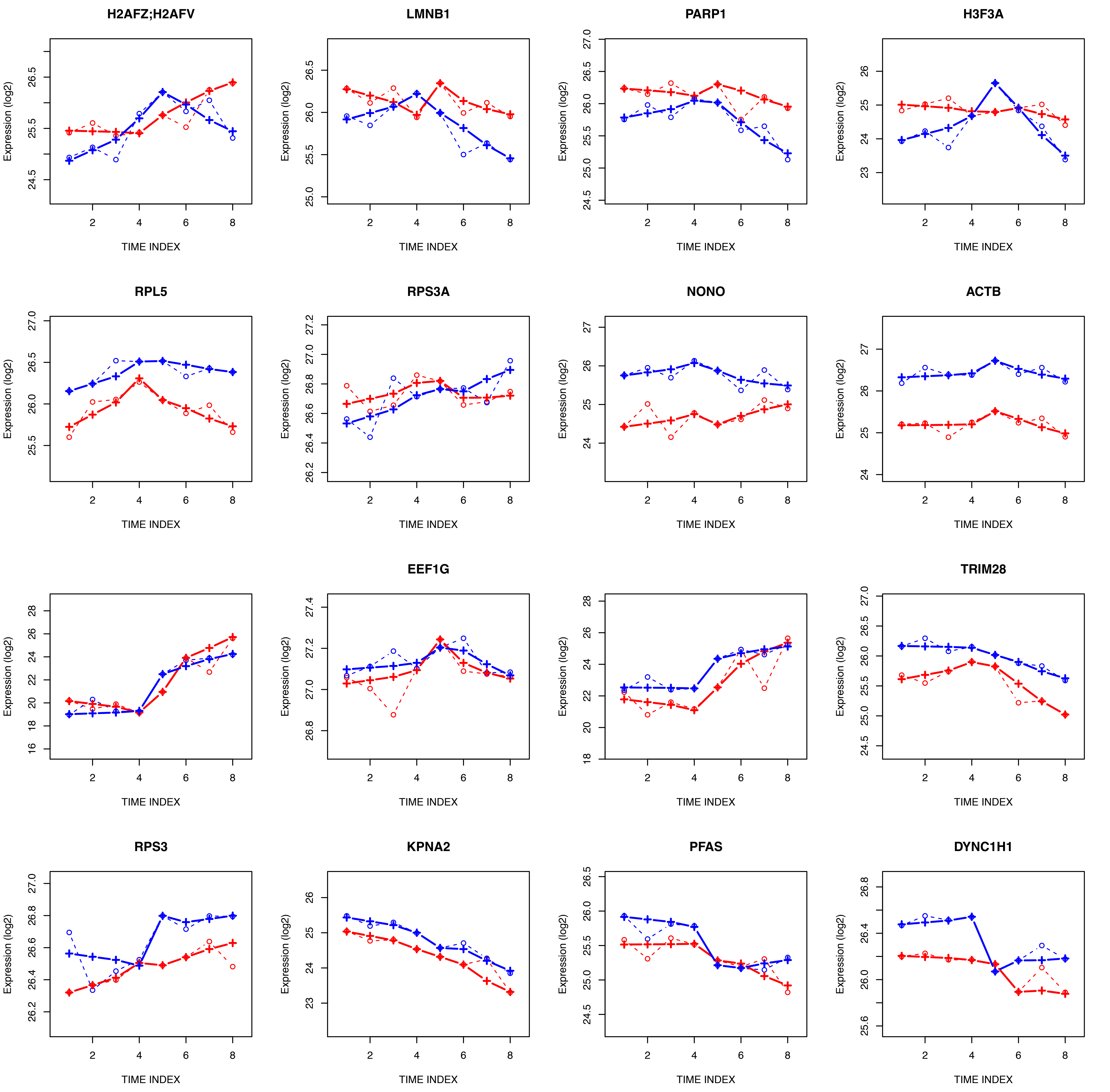

H2AFZ;H2AFV
LMNB1
PARP1
H3F3A
RPL5
RPS3A
NONO
ACTB
EEF1G
TRIM28
RPS3
KPNA2
PFAS
DYNC1H1
Expression (log2)
TIME INDEX

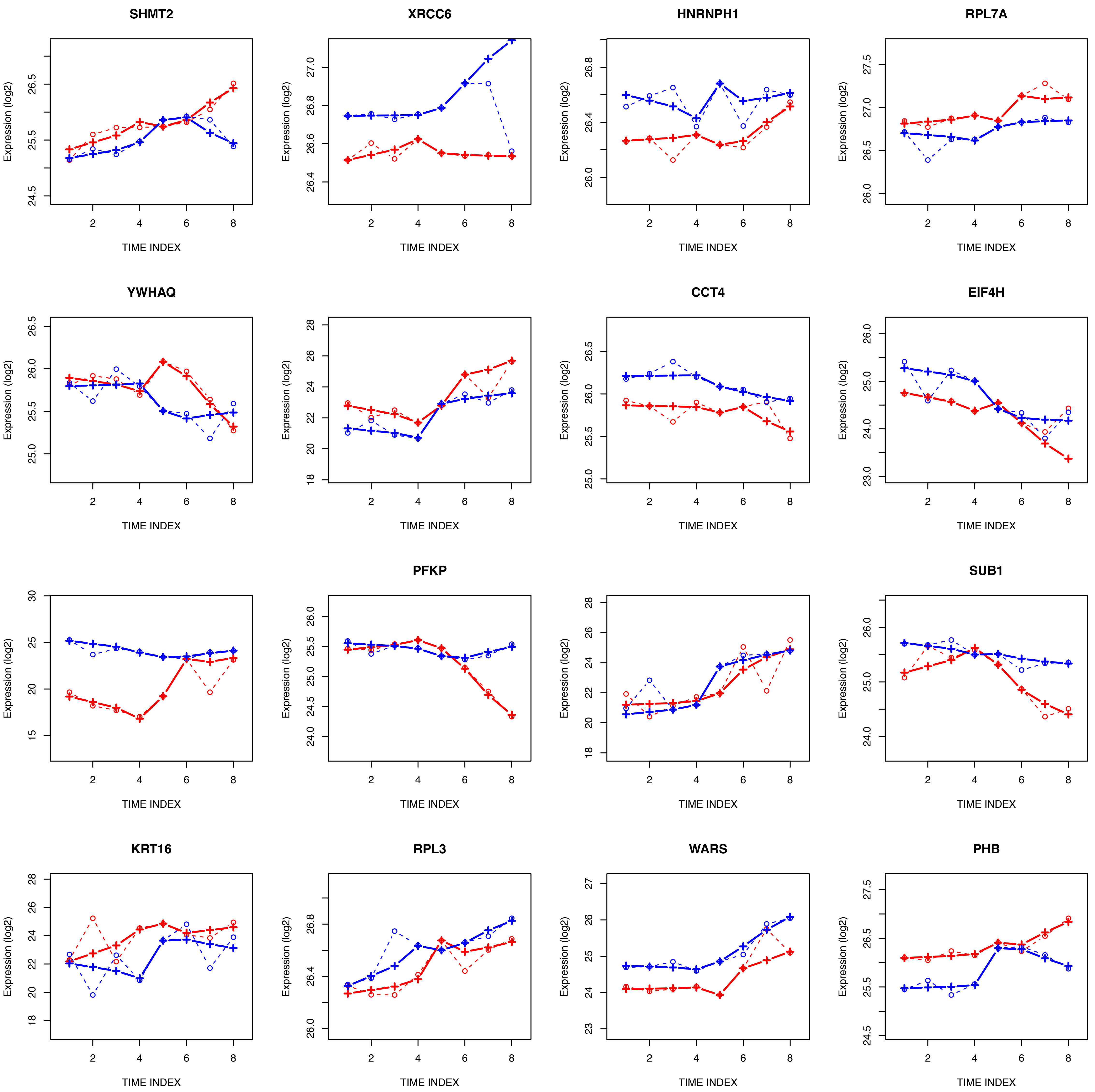
SHMT2
Expression (log2)
24.5
25.5
26.5
2
4
6
8
TIME INDEX
XRCC6
Expression (log2)
26.4
26.6
26.8
27.0
2
4
6
8
TIME INDEX
HNRNPH1
Expression (log2)
26.0
26.4
26.8
2
4
6
8
TIME INDEX
RPL7A
Expression (log2)
26.0
26.5
27.0
27.5
2
4
6
8
TIME INDEX
YWHAQ
Expression (log2)
25.0
25.5
26.0
26.5
2
4
6
8
TIME INDEX
Expression (log2)
18
20
22
24
26
28
2
4
6
8
TIME INDEX
CCT4
Expression (log2)
25.0
25.5
26.0
26.5
2
4
6
8
TIME INDEX
EIF4H
Expression (log2)
23.0
24.0
25.0
26.0
2
4
6
8
TIME INDEX
Expression (log2)
15
20
25
30
2
4
6
8
TIME INDEX
PFKP
Expression (log2)
24.0
24.5
25.0
25.5
26.0
2
4
6
8
TIME INDEX
Expression (log2)
18
20
22
24
26
28
2
4
6
8
TIME INDEX
SUB1
Expression (log2)
24.0
25.0
26.0
2
4
6
8
TIME INDEX
KRT16
Expression (log2)
18
20
22
24
26
28
2
4
6
8
TIME INDEX
RPL3
Expression (log2)
26.0
26.4
26.8
2
4
6
8
TIME INDEX
WARS
Expression (log2)
23
24
25
26
27
2
4
6
8
TIME INDEX
PHB
Expression (log2)
24.5
25.5
26.5
27.5
2
4
6
8
TIME INDEX

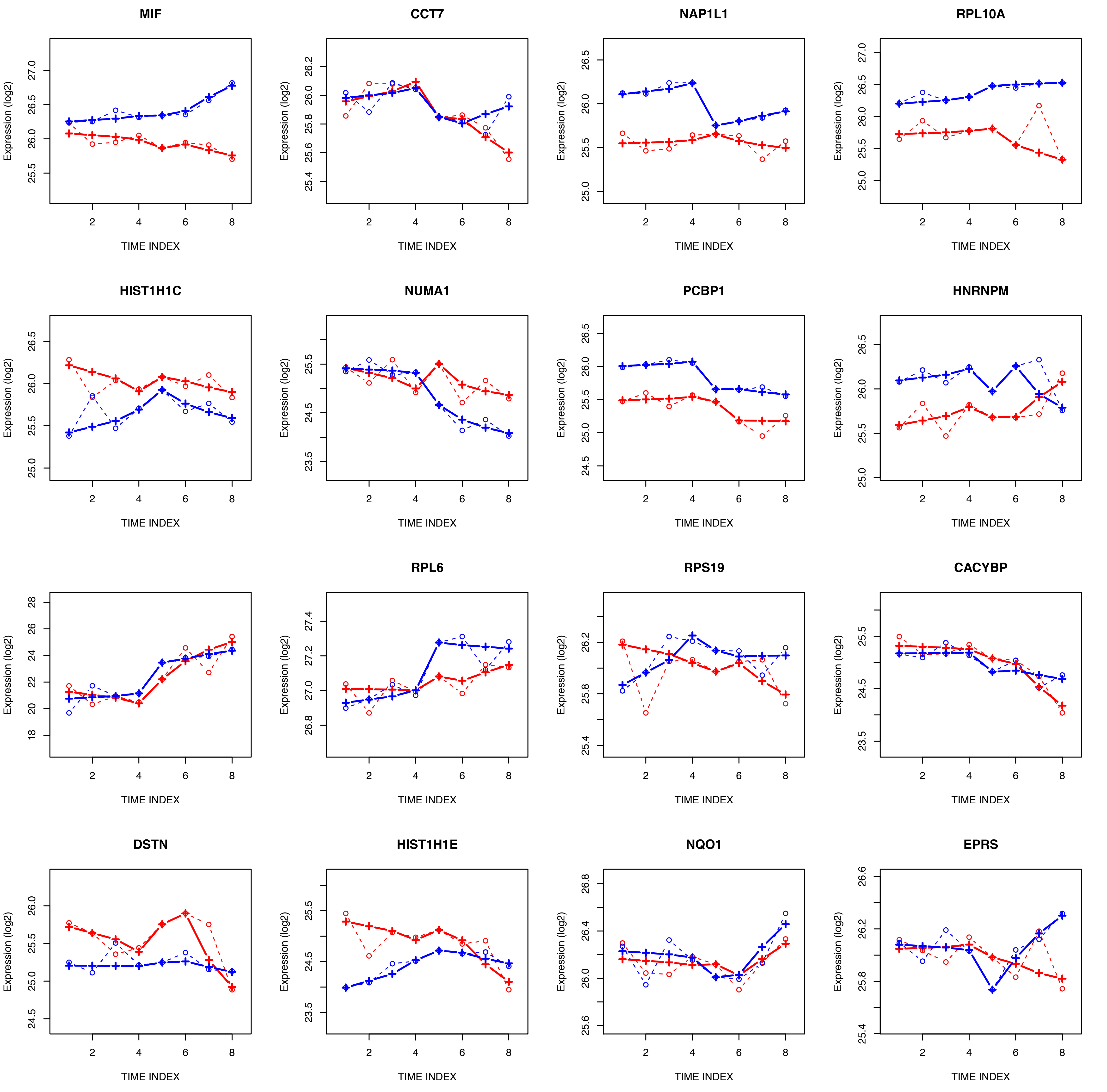
MIF
CCT7
NAP1L1
RPL10A
HIST1H1C
NUMA1
PCBP1
HNRNPM
RPL6
RPS19
CACYBP
DSTN
HIST1H1E
NQO1
EPRS
Expression (log2)
TIME INDEX

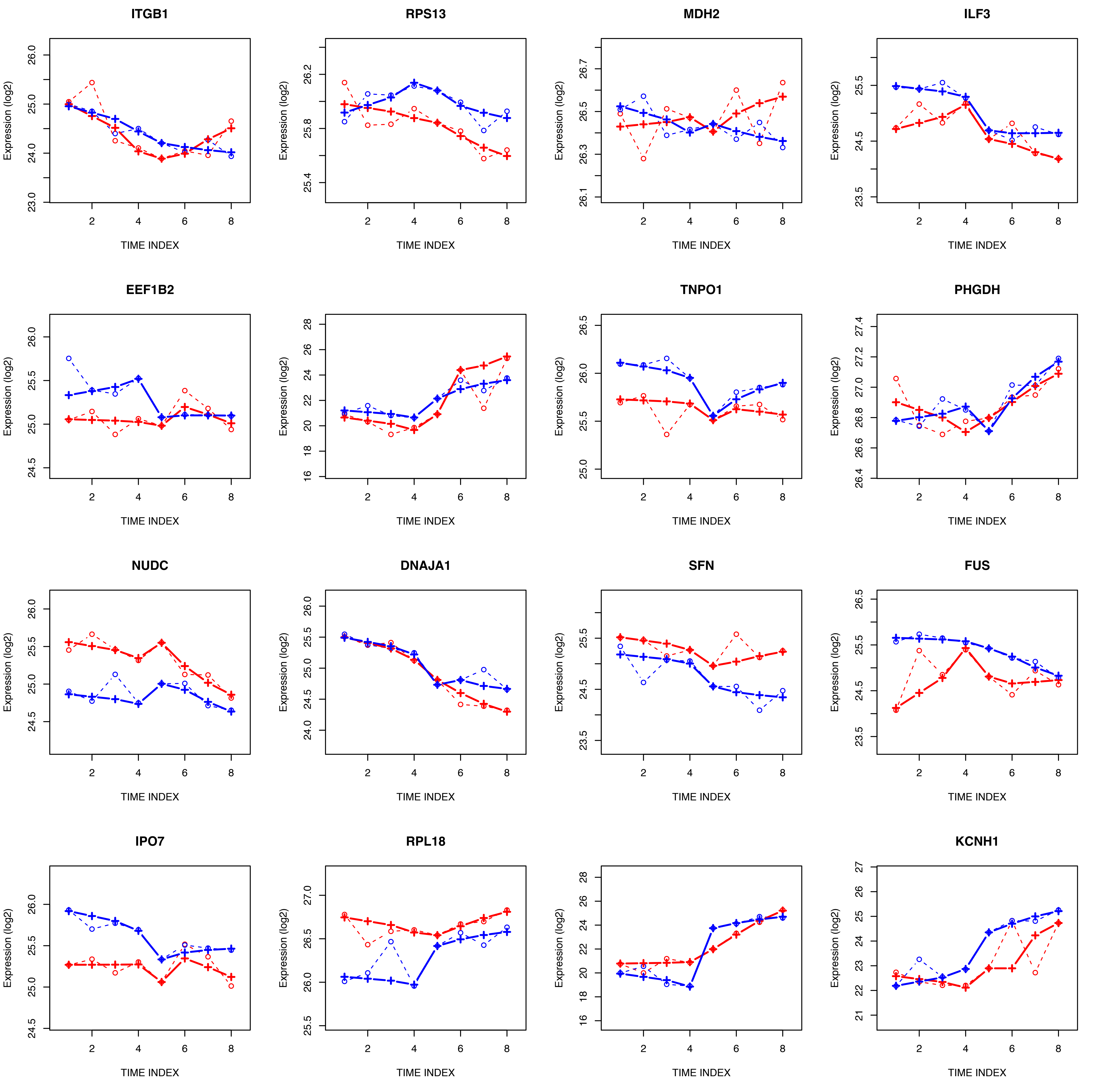
ITGB1
RPS13
MDH2
ILF3
EEF1B2
TNPO1
PHGDH
NUDC
DNAJA1
SFN
FUS
IPO7
RPL18
KCNH1
Expression (log2)
TIME INDEX

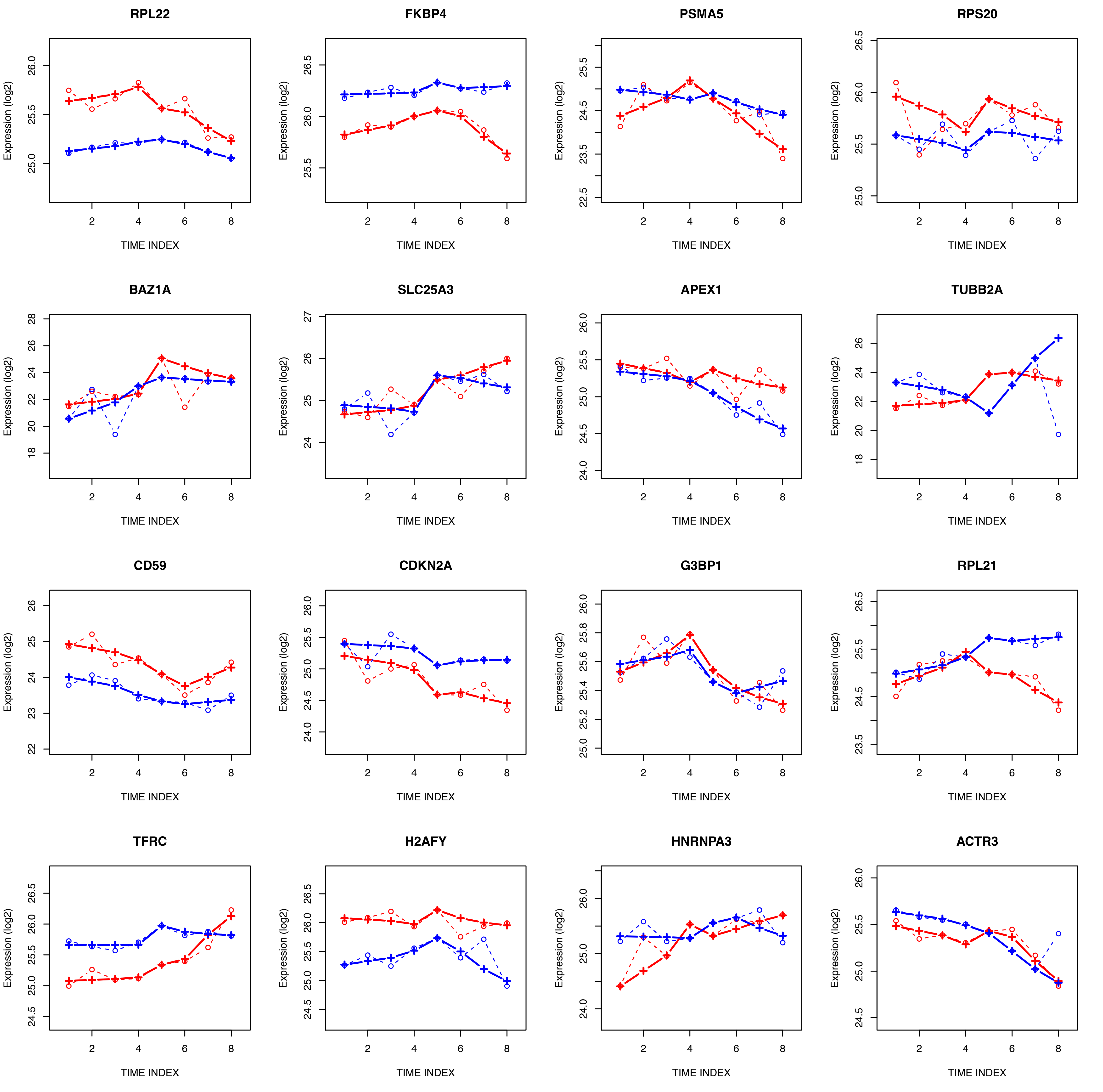
RPL22
FKBP4
PSMA5
RPS20
BAZ1A
SLC25A3
APEX1
TUBB2A
CD59
CDKN2A
G3BP1
RPL21
TFRC
H2AFY
HNRNPA3
ACTR3
Expression (log2)
TIME INDEX

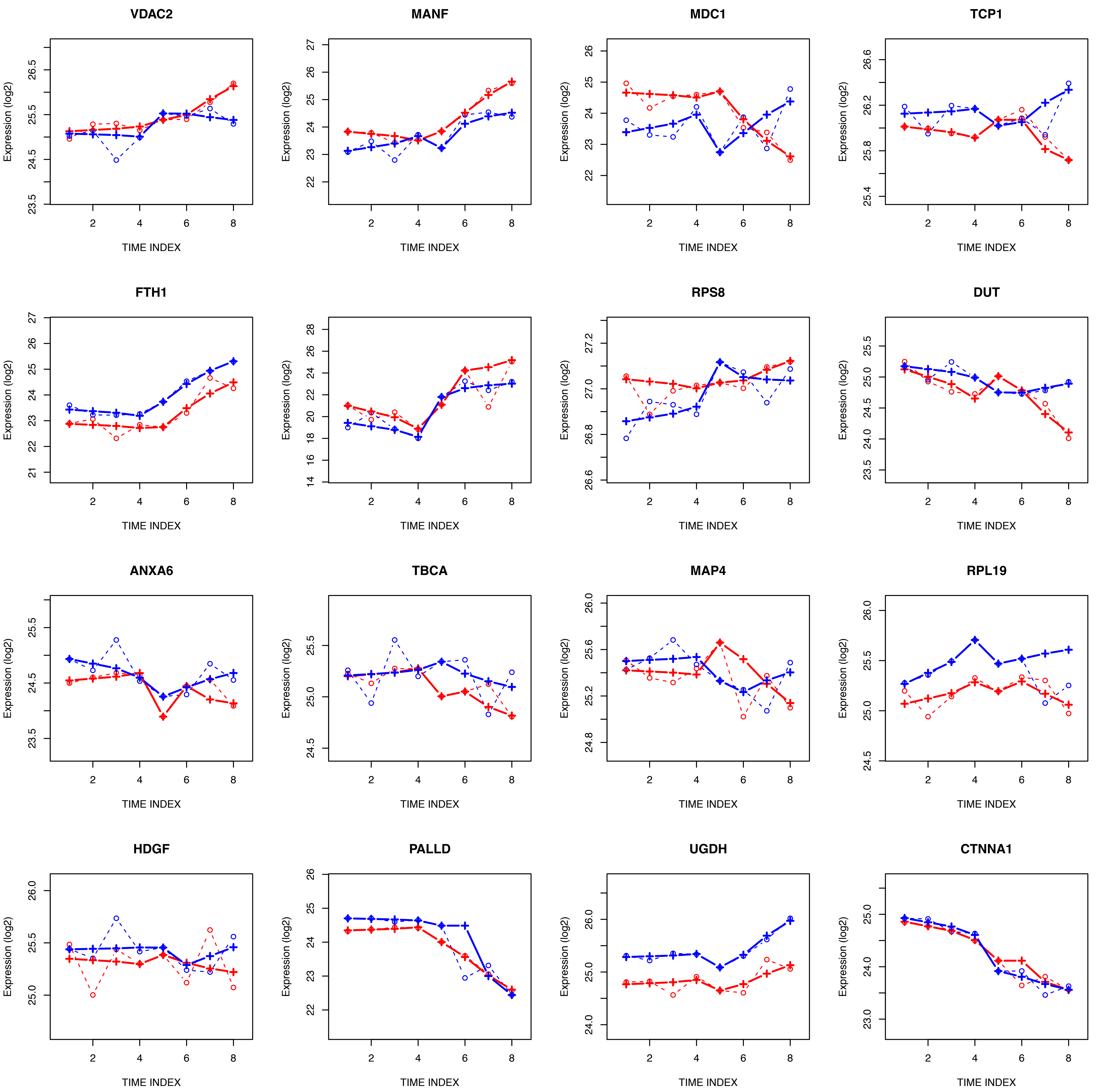
VDAC2
MANF
MDC1
TCP1
FTH1
RPS8
DUT
ANXA6
TBCA
MAP4
RPL19
HDGF
PALLD
UGDH
CTNNA1
Expression (log2)
TIME INDEX

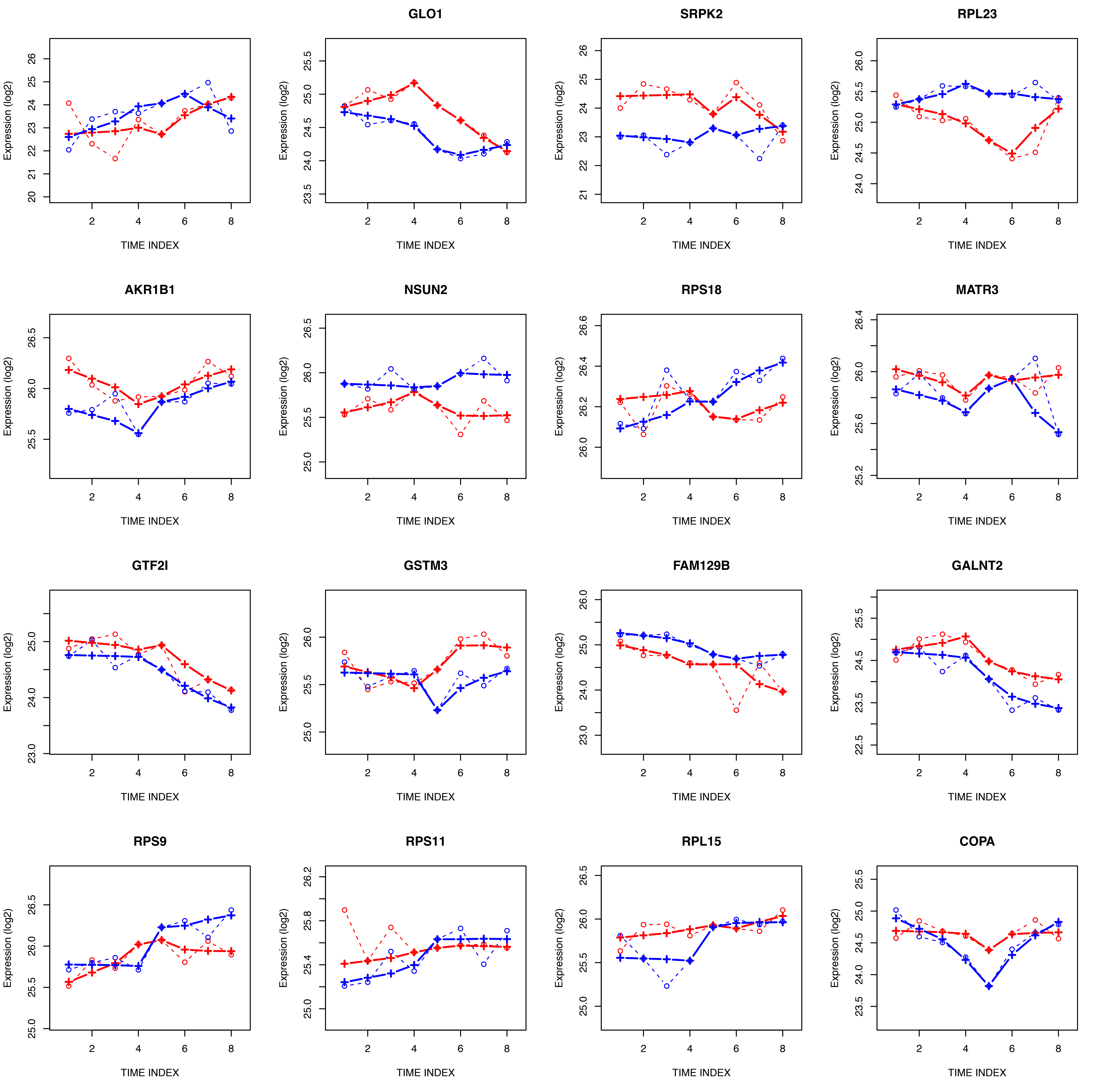
GLO1
SRPK2
RPL23
AKR1B1
NSUN2
RPS18
MATR3
GTF2I
GSTM3
FAM129B
GALNT2
RPS9
RPS11
RPL15
COPA
Expression (log2)
TIME INDEX

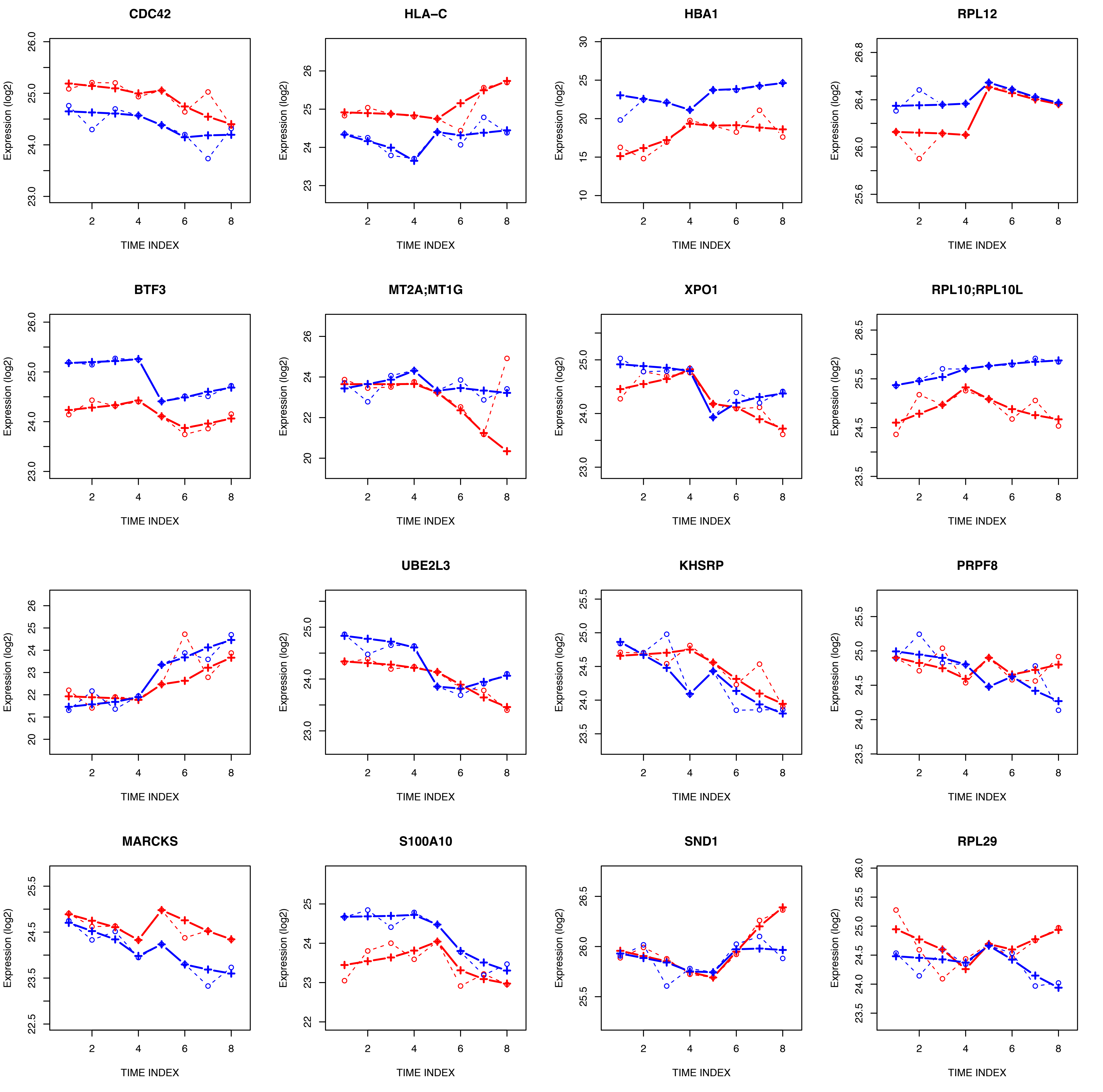
CDC42
HLA−C
HBA1
RPL12
BTF3
MT2A;MT1G
XPO1
RPL10;RPL10L
UBE2L3
KHSRP
PRPF8
MARCKS
S100A10
SND1
RPL29
Expression (log2)
TIME INDEX

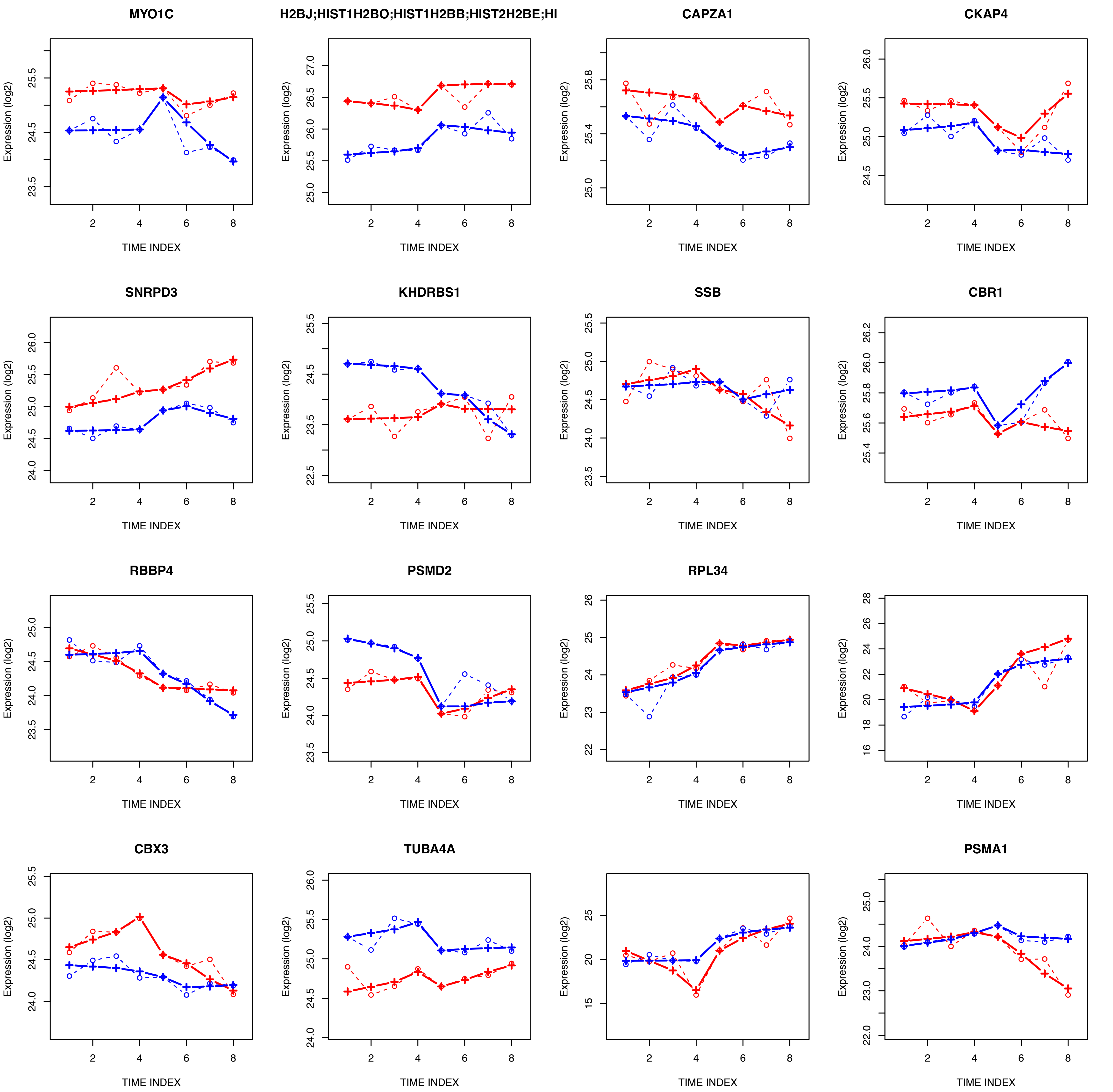
MYO1C
H2BJ;HIST1H2BO;HIST1H2BB;HIST2H2BE;HI
CAPZA1
CKAP4
SNRPD3
KHDRBS1
SSB
CBR1
RBBP4
PSMD2
RPL34
CBX3
TUBA4A
PSMA1
Expression (log2)
TIME INDEX

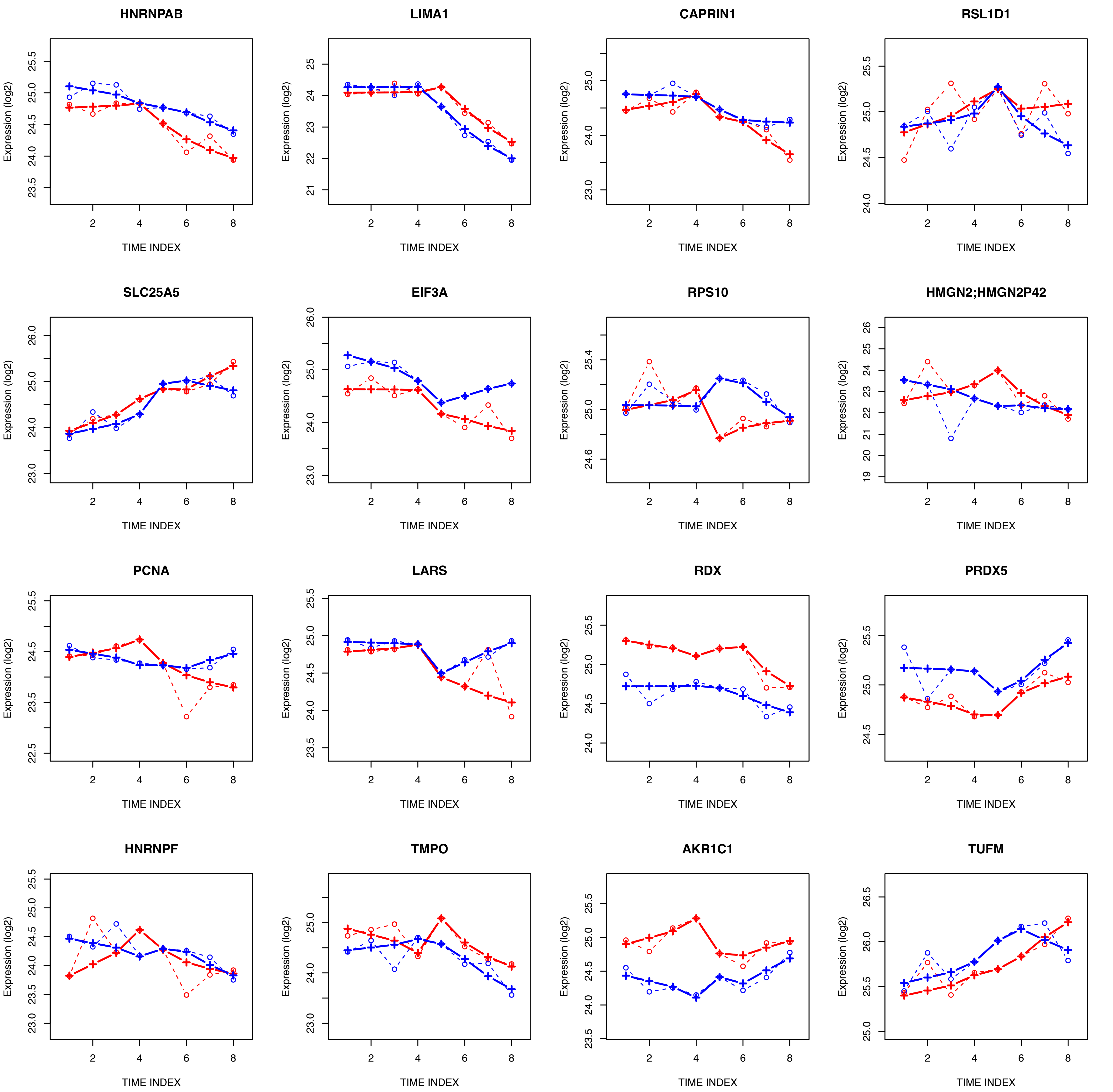
HNRNPAB
LIMA1
CAPRIN1
RSL1D1
SLC25A5
EIF3A
RPS10
HMGN2;HMGN2P42
PCNA
LARS
RDX
PRDX5
HNRNPF
TMPO
AKR1C1
TUFM
Expression (log2)
TIME INDEX

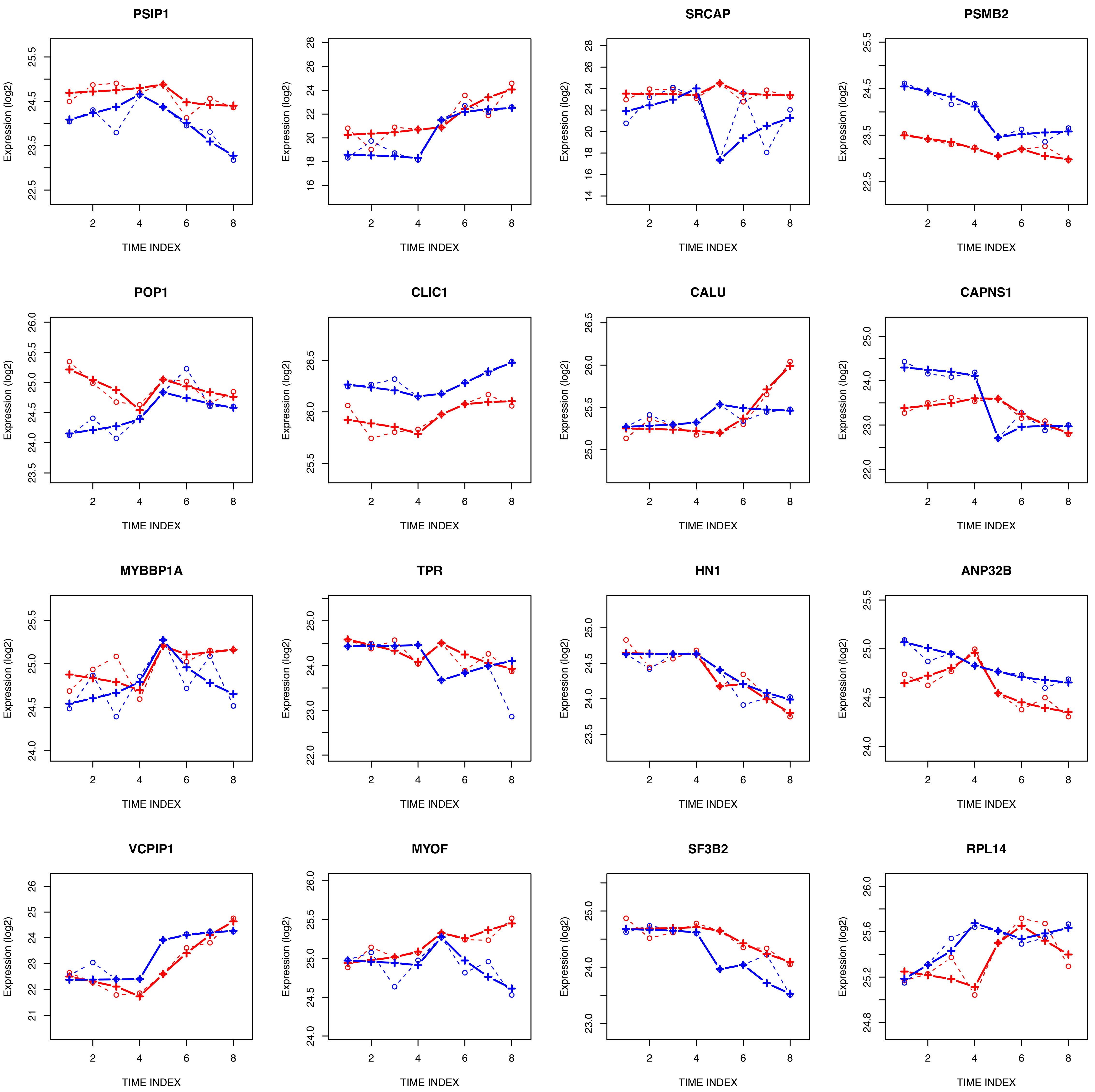
PSIP1
SRCAP
PSMB2
POP1
CLIC1
CALU
CAPNS1
MYBBP1A
TPR
HN1
ANP32B
VCPIP1
MYOF
SF3B2
RPL14
Expression (log2)
TIME INDEX

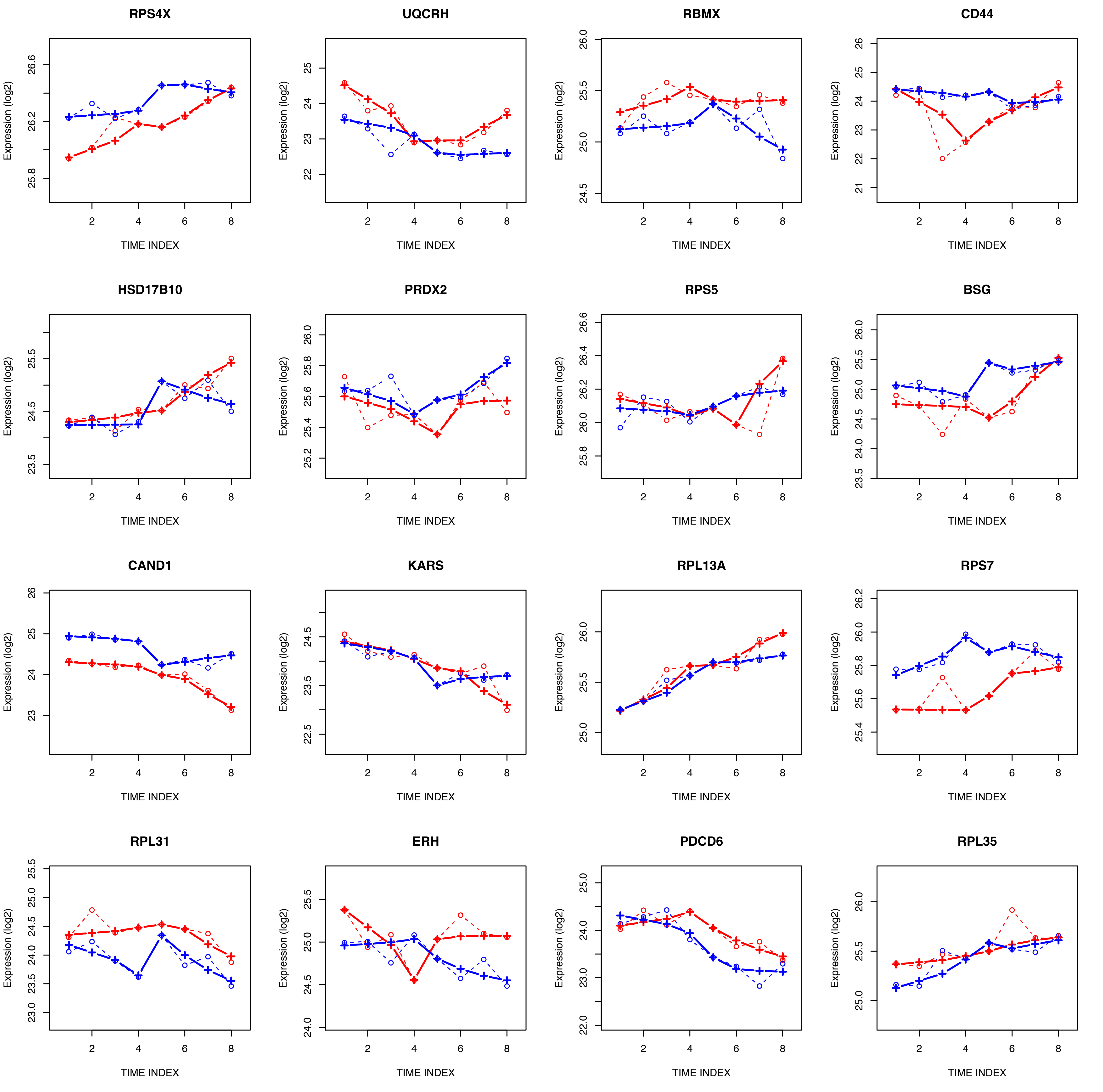

RPS4X
UQCRH
RBMX
CD44
HSD17B10
PRDX2
RPS5
BSG
CAND1
KARS
RPL13A
RPS7
RPL31
ERH
PDCD6
RPL35
Expression (log2)
TIME INDEX

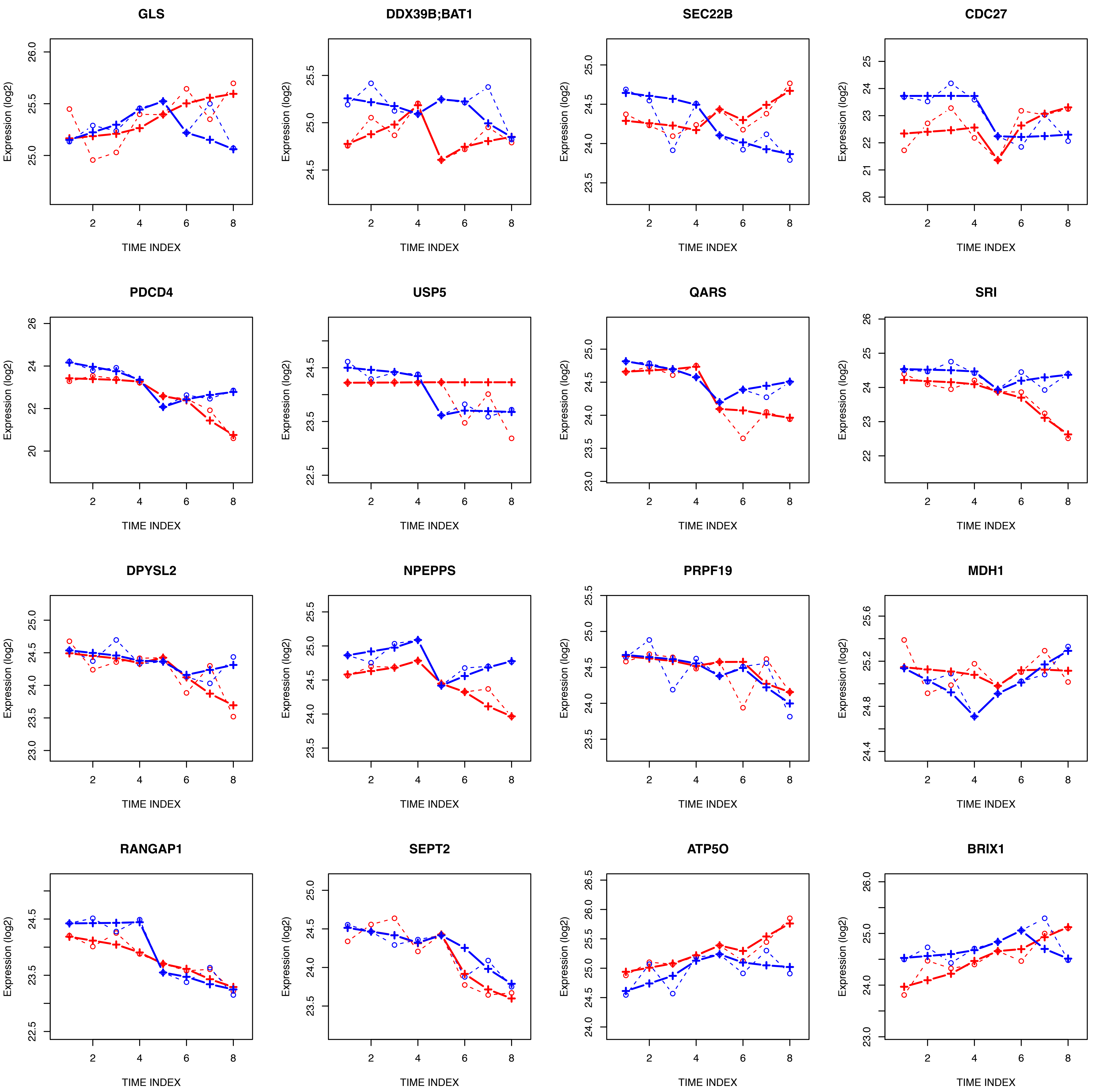
GLS
DDX39B;BAT1
SEC22B
CDC27
PDCD4
USP5
QARS
SRI
DPYSL2
NPEPPS
PRPF19
MDH1
RANGAP1
SEPT2
ATP5O
BRIX1
Expression (log2)
TIME INDEX

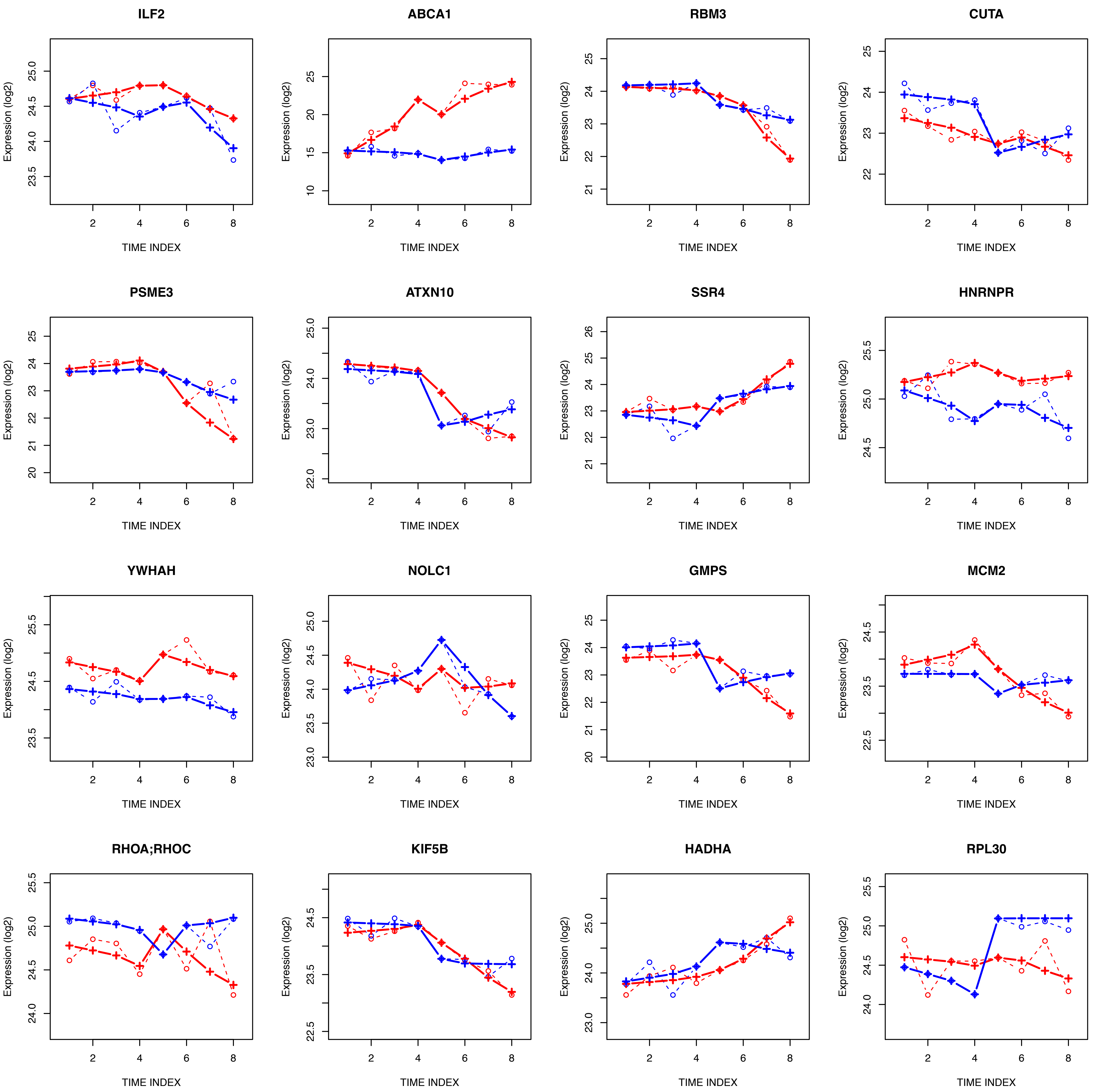
ILF2
ABCA1
RBM3
CUTA
PSME3
ATXN10
SSR4
HNRNPR
YWHAH
NOLC1
GMPS
MCM2
RHOA;RHOC
KIF5B
HADHA
RPL30
Expression (log2)
TIME INDEX

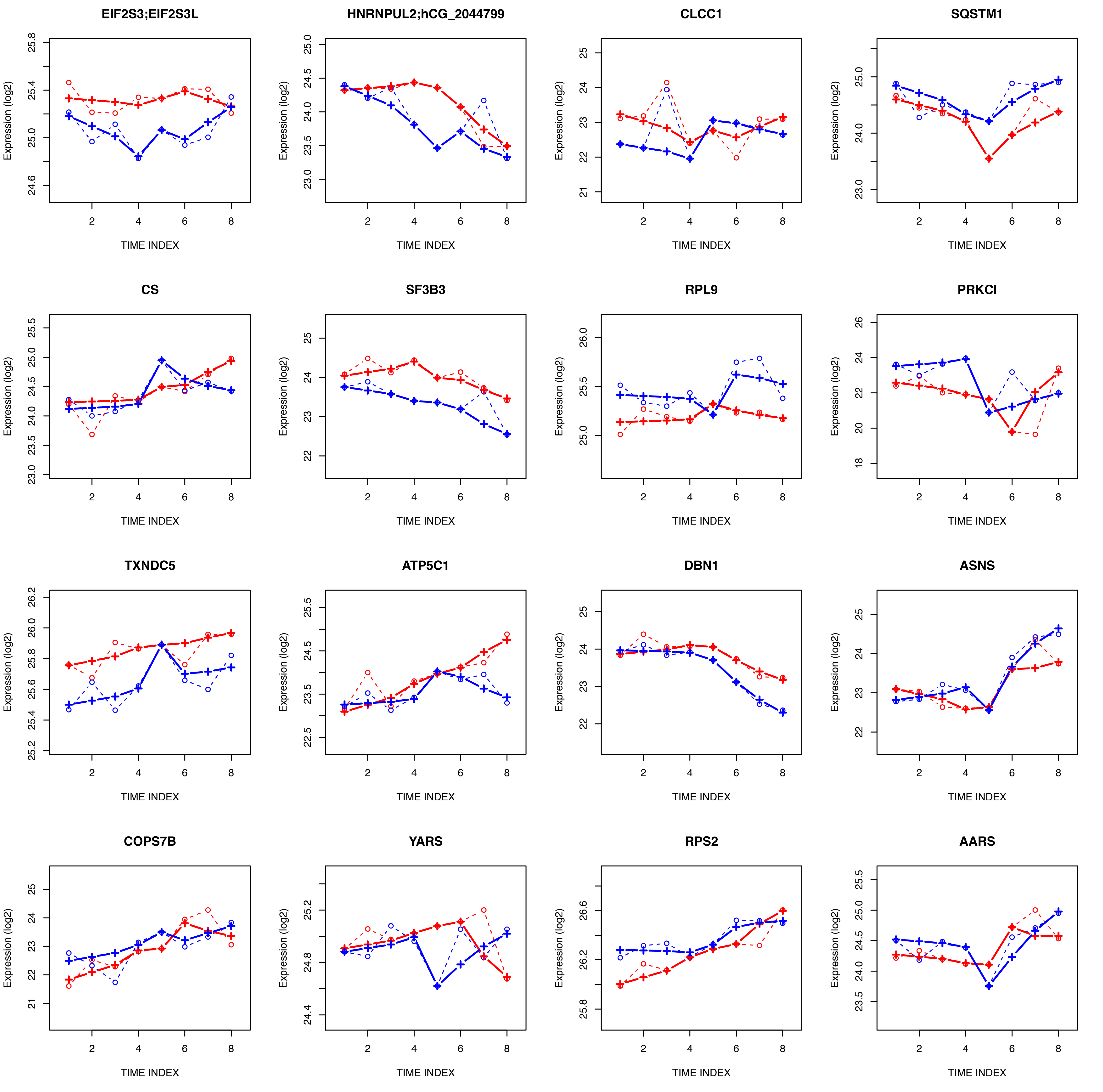

EIF2S3;EIF2S3L
HNRNPUL2;hCG_2044799
CLCC1
SQSTM1
CS
SF3B3
RPL9
PRKCI
TXNDC5
ATP5C1
DBN1
ASNS
COPS7B
YARS
RPS2
AARS
Expression (log2)
TIME INDEX

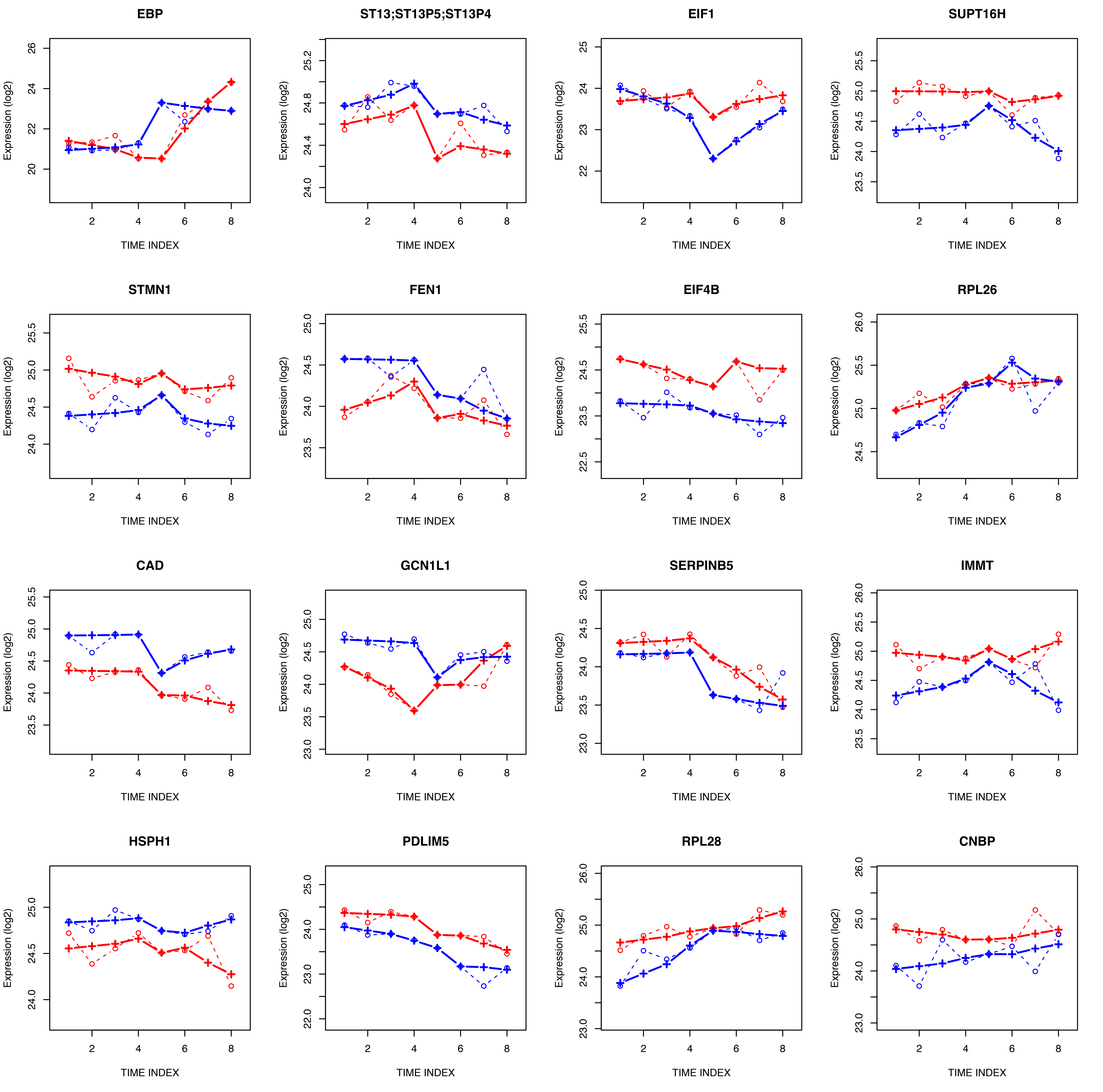

EBP
ST13;ST13P5;ST13P4
EIF1
SUPT16H
STMN1
FEN1
EIF4B
RPL26
CAD
GCN1L1
SERPINB5
IMMT
HSPH1
PDLIM5
RPL28
CNBP
Expression (log2)
TIME INDEX

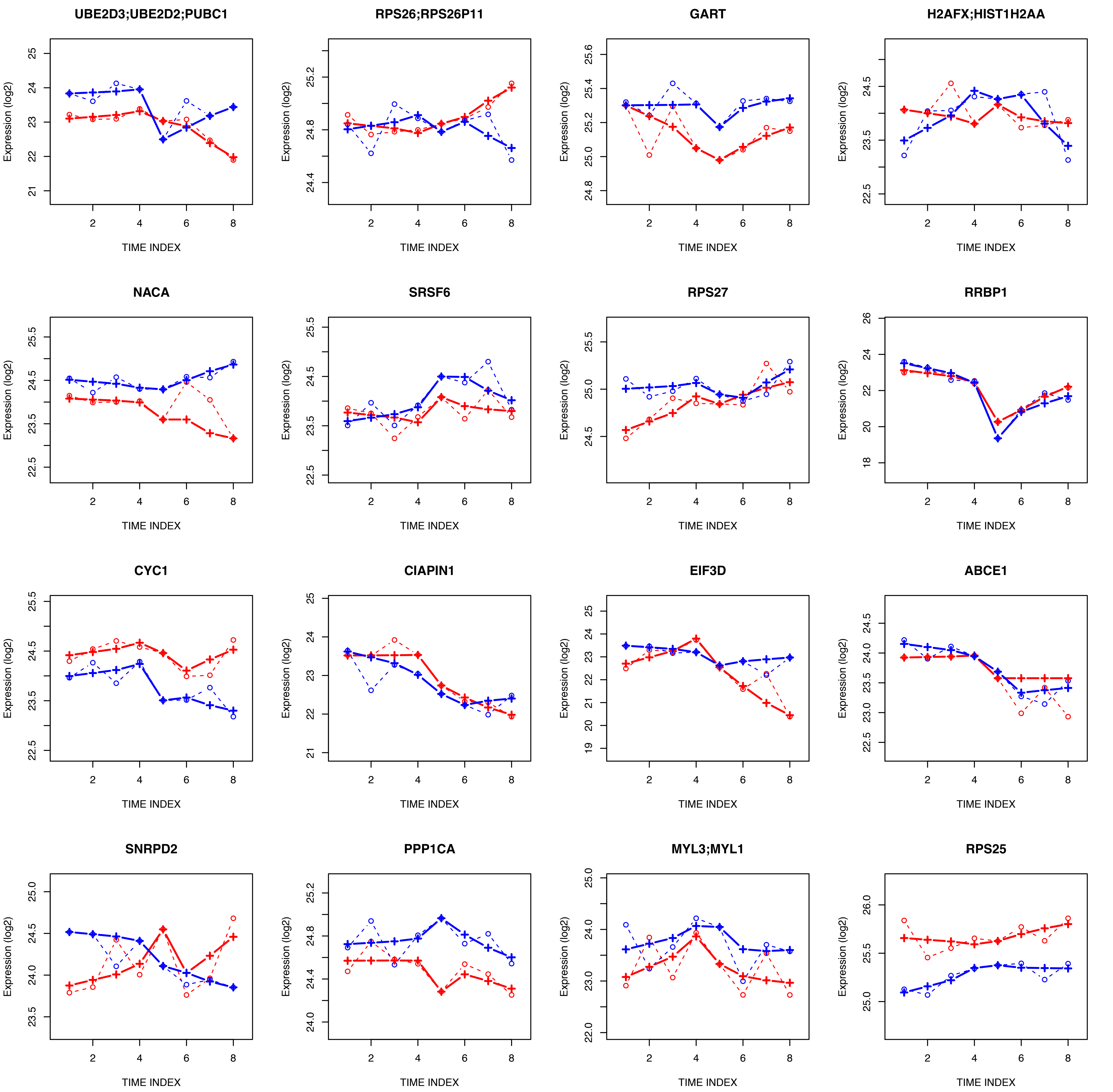
UBE2D3;UBE2D2;PUBC1
RPS26;RPS26P11
GART
H2AFX;HIST1H2AA
NACA
SRSF6
RPS27
RRBP1
CYC1
CIAPIN1
EIF3D
ABCE1
SNRPD2
PPP1CA
MYL3;MYL1
RPS25
Expression (log2)
TIME INDEX

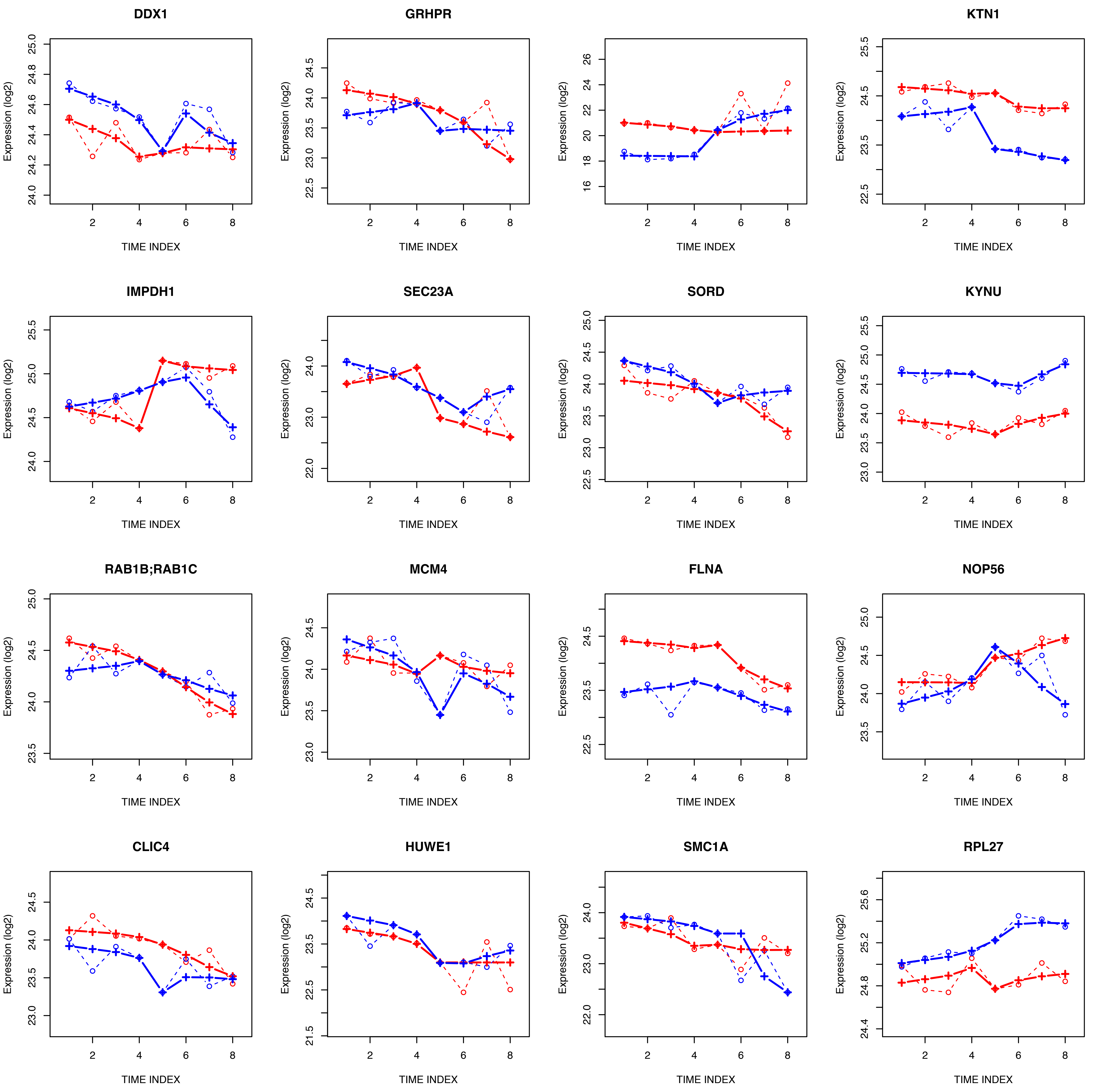
DDX1
GRHPR
KTN1
IMPDH1
SEC23A
SORD
KYNU
RAB1B;RAB1C
MCM4
FLNA
NOP56
CLIC4
HUWE1
SMC1A
RPL27
Expression (log2)
TIME INDEX

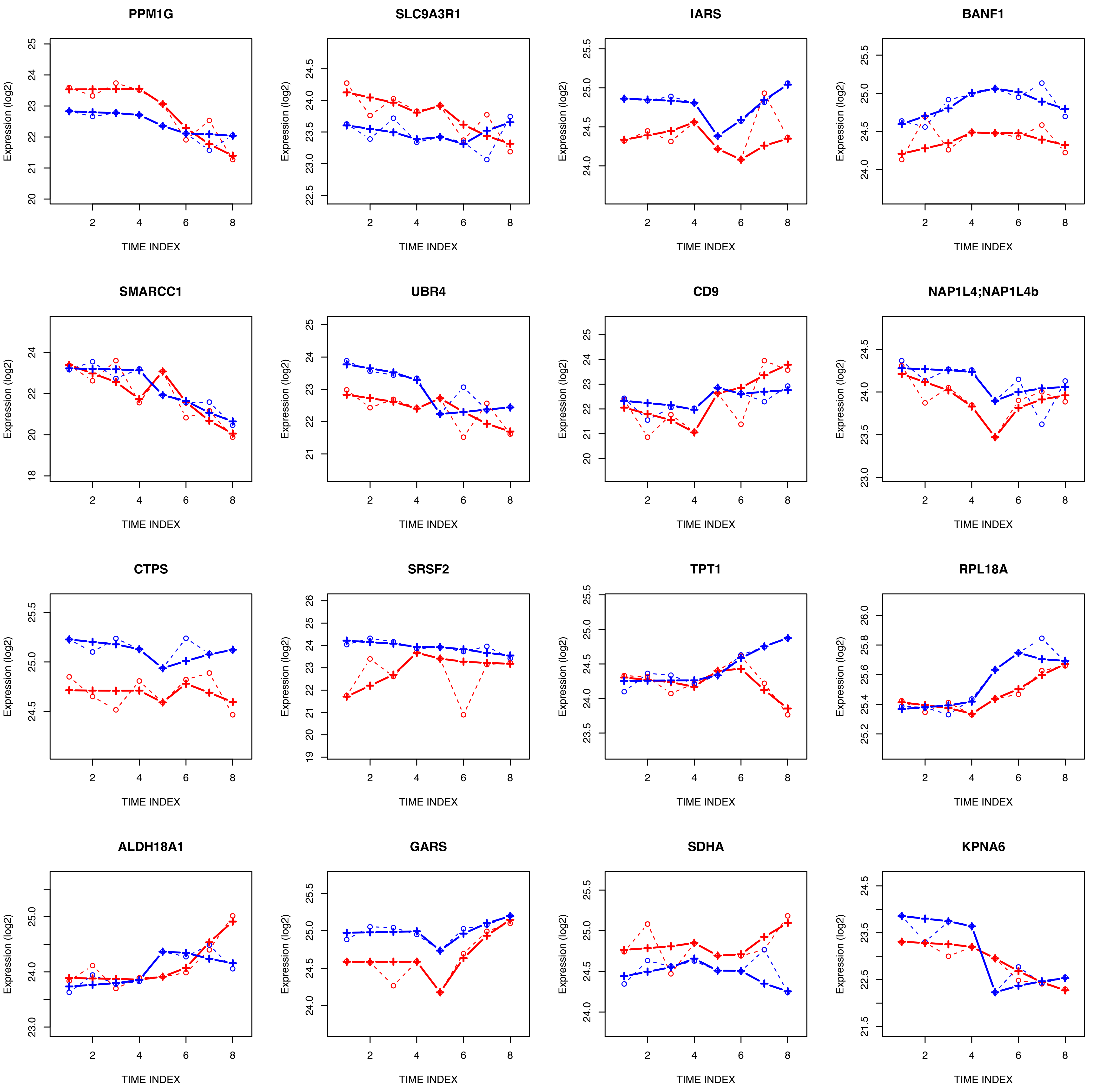
PPM1G
SLC9A3R1
IARS
BANF1
SMARCC1
UBR4
CD9
NAP1L4;NAP1L4b
CTPS
SRSF2
TPT1
RPL18A
ALDH18A1
GARS
SDHA
KPNA6
Expression (log2)
TIME INDEX

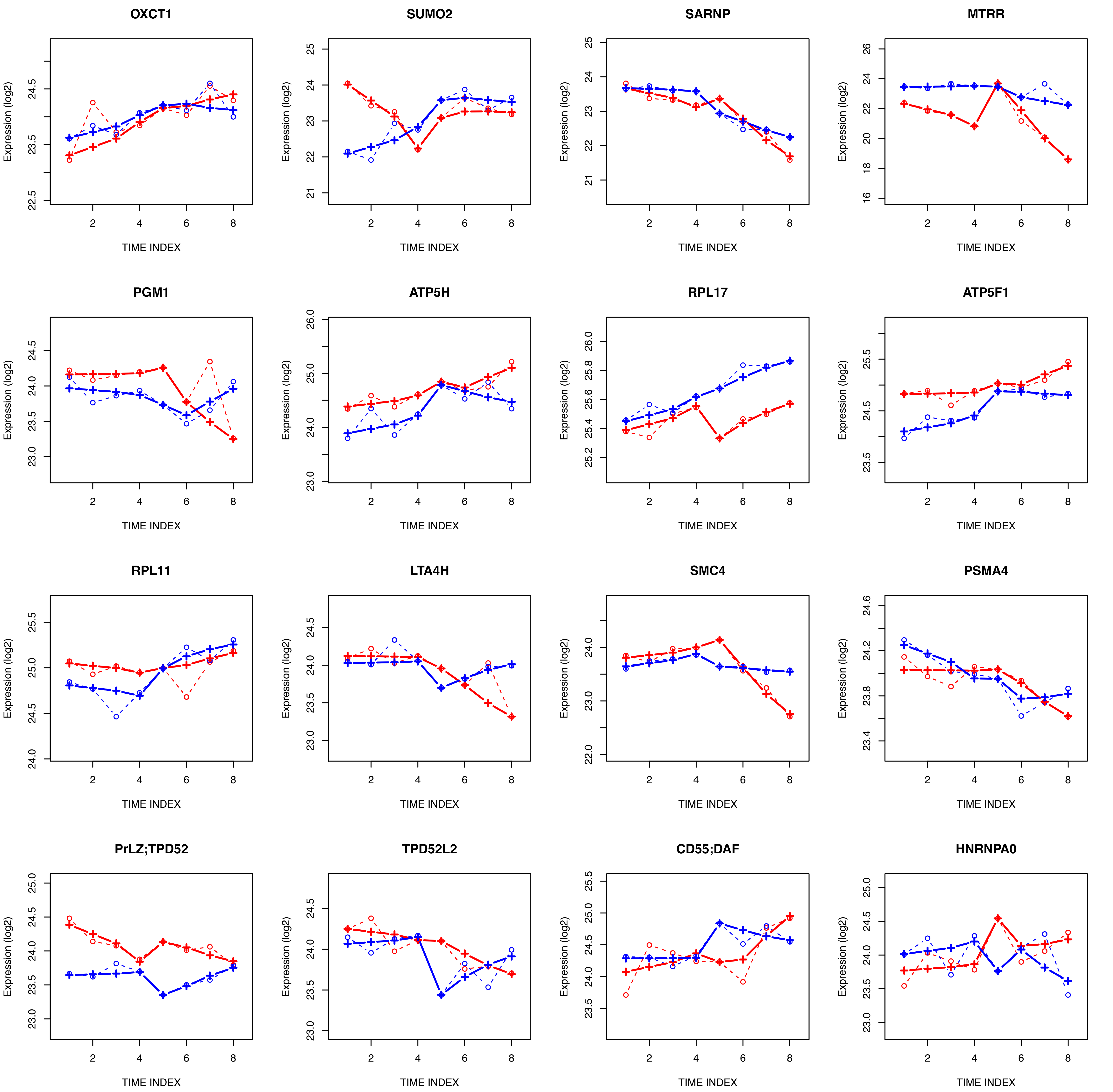

OXCT1
SUMO2
SARNP
MTRR
PGM1
ATP5H
RPL17
ATP5F1
RPL11
LTA4H
SMC4
PSMA4
PrLZ;TPD52
TPD52L2
CD55;DAF
HNRNPA0
Expression (log2)
TIME INDEX

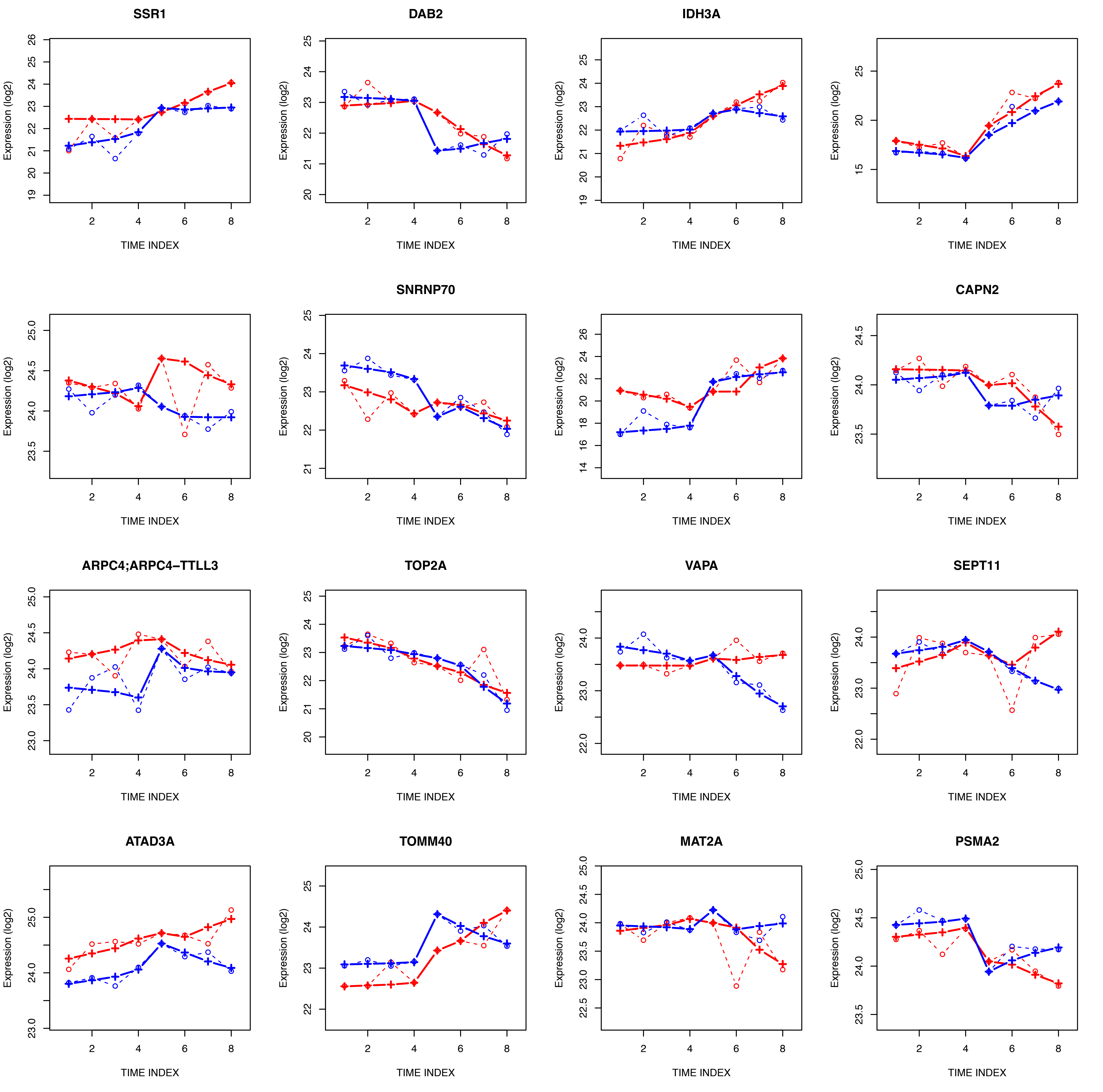
SSR1
DAB2
IDH3A
SNRNP70
CAPN2
ARPC4;ARPC4−TTLL3
TOP2A
VAPA
SEPT11
ATAD3A
TOMM40
MAT2A
PSMA2
Expression (log2)
TIME INDEX

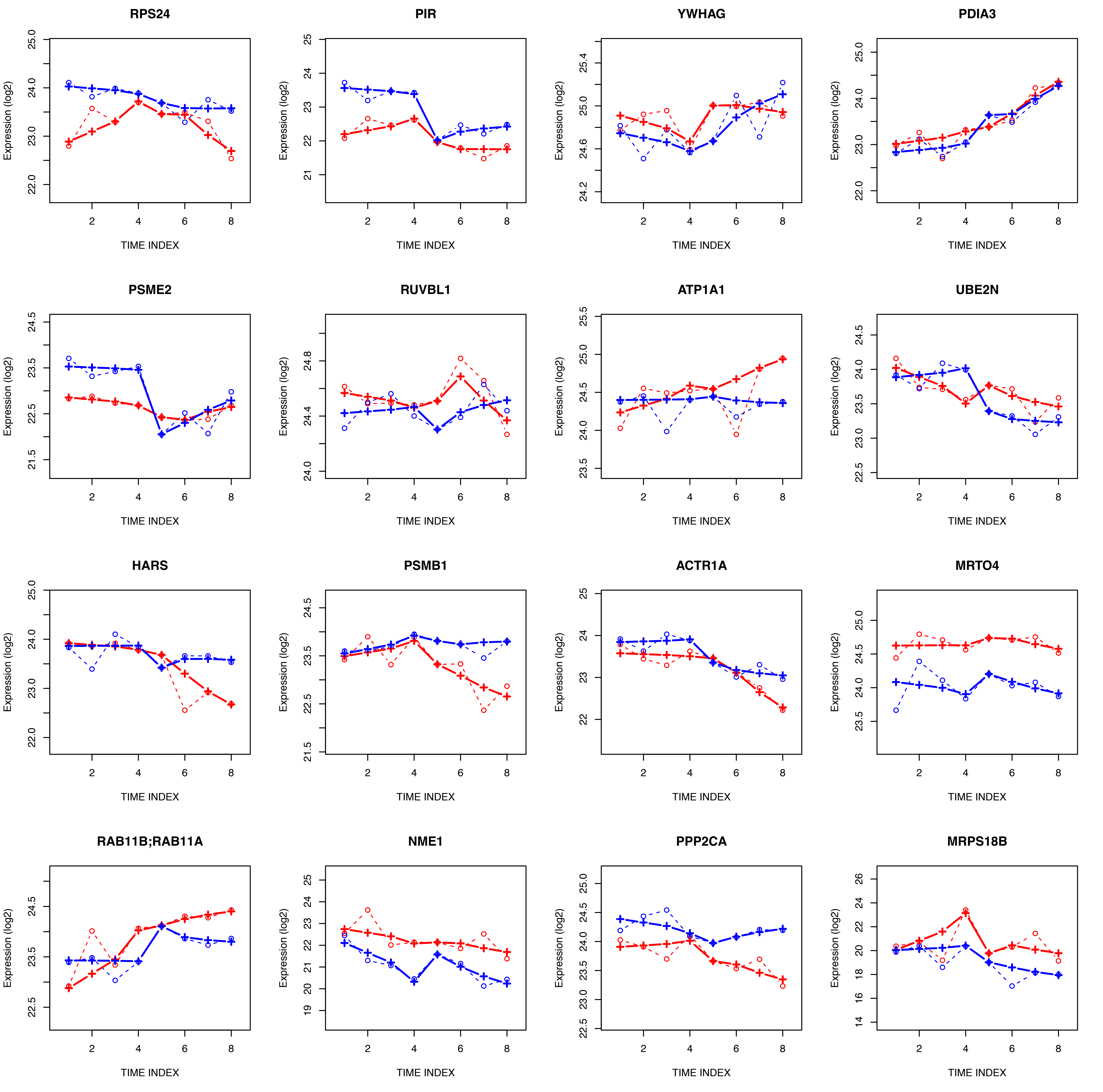

RPS24
PIR
YWHAG
PDIA3
PSME2
RUVBL1
ATP1A1
UBE2N
HARS
PSMB1
ACTR1A
MRTO4
RAB11B;RAB11A
NME1
PPP2CA
MRPS18B
Expression (log2)
TIME INDEX

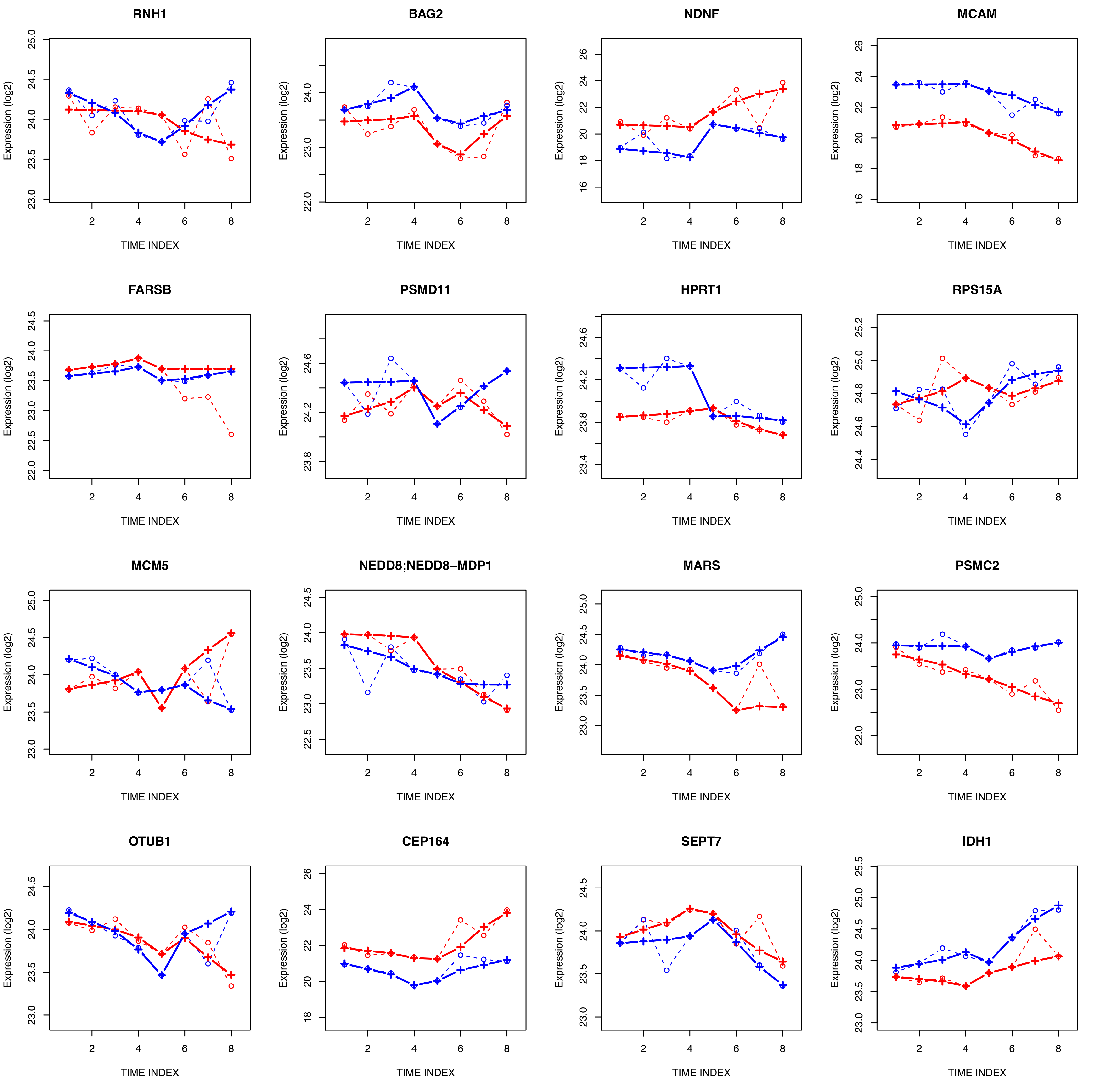
RNH1
BAG2
NDNF
MCAM
FARSB
PSMD11
HPRT1
RPS15A
MCM5
NEDD8;NEDD8–MDP1
MARS
PSMC2
OTUB1
CEP164
SEPT7
IDH1
Expression (log2)
TIME INDEX

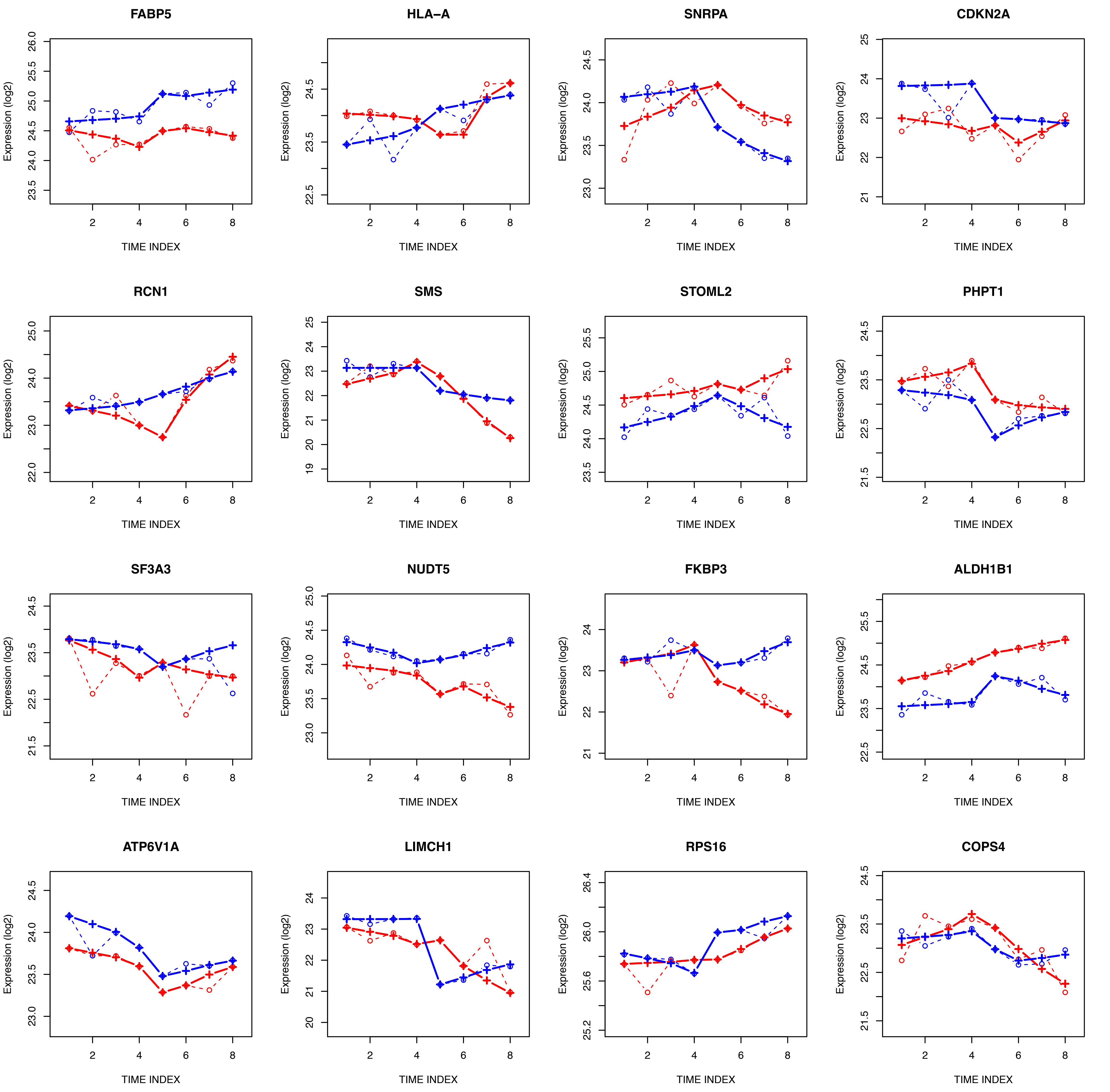
FABP5
HLA−A
SNRPA
CDKN2A
RCN1
SMS
STOML2
PHPT1
SF3A3
NUDT5
FKBP3
ALDH1B1
ATP6V1A
LIMCH1
RPS16
COPS4
Expression (log2)
TIME INDEX

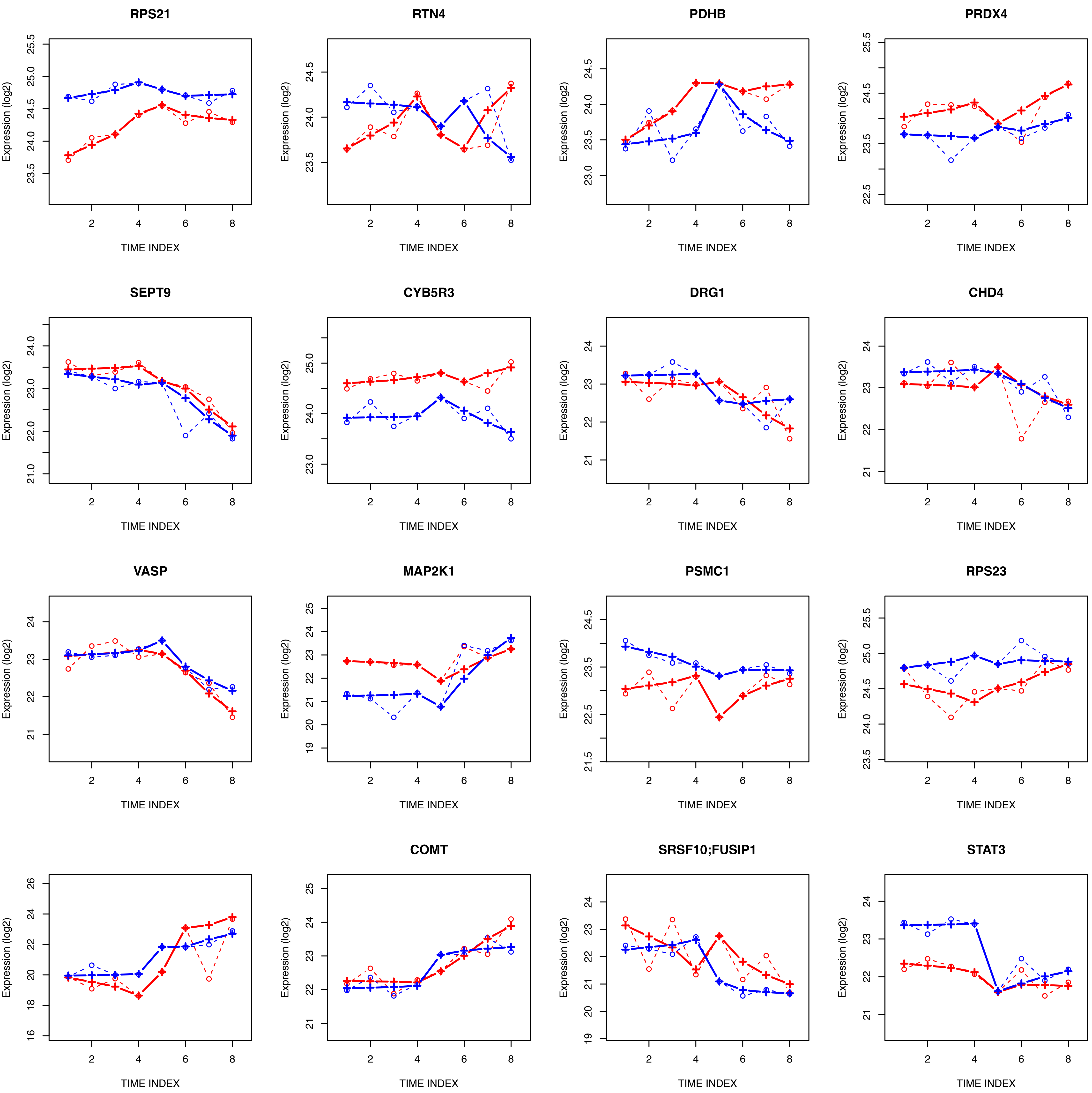
RPS21
RTN4
PDHB
PRDX4
SEPT9
CYB5R3
DRG1
CHD4
VASP
MAP2K1
PSMC1
RPS23
COMT
SRSF10;FUSIP1
STAT3
Expression (log2)
TIME INDEX

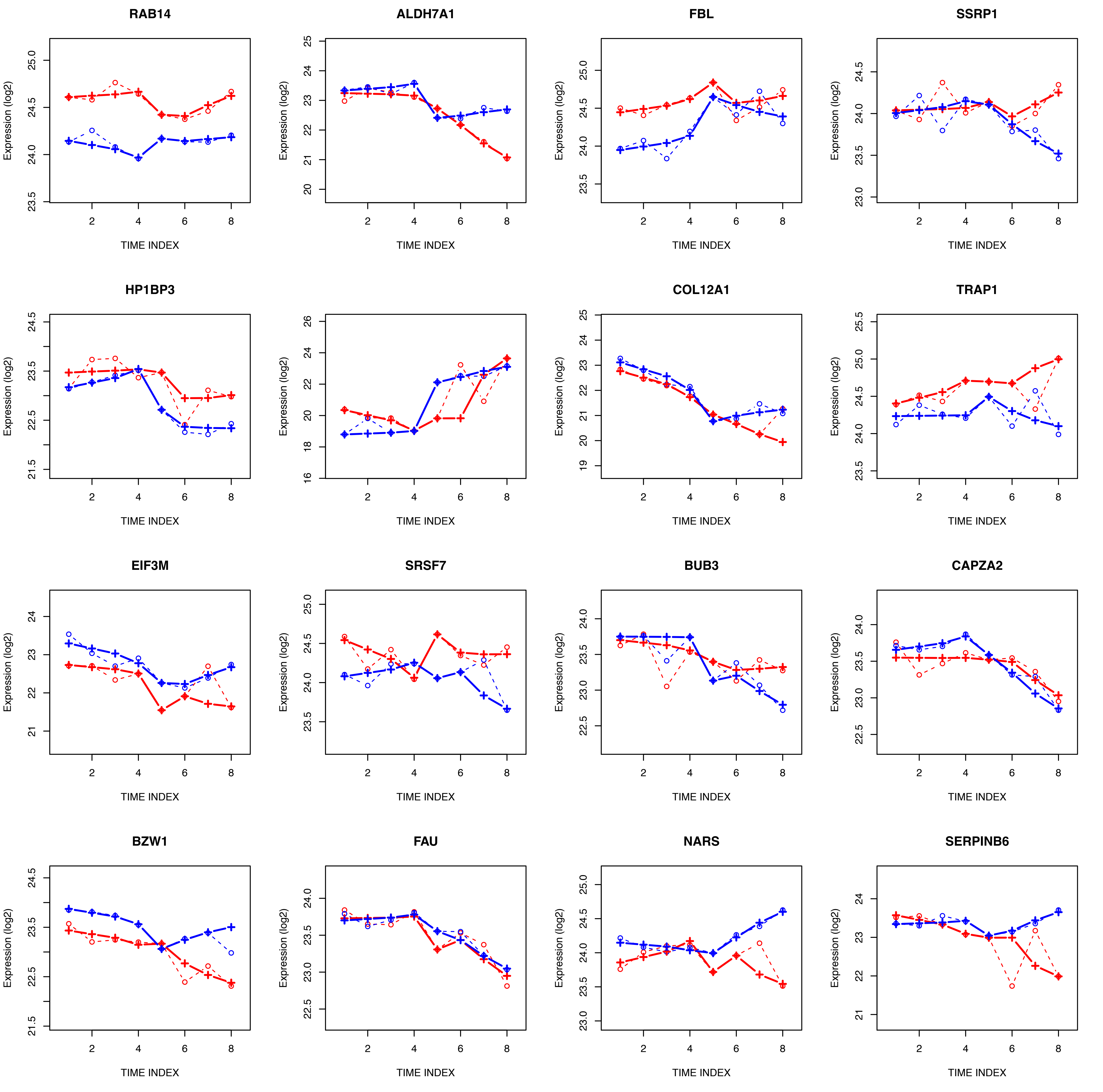

RAB14
ALDH7A1
FBL
SSRP1
HP1BP3
COL12A1
TRAP1
EIF3M
SRSF7
BUB3
CAPZA2
BZW1
FAU
NARS
SERPINB6
Expression (log2)
TIME INDEX

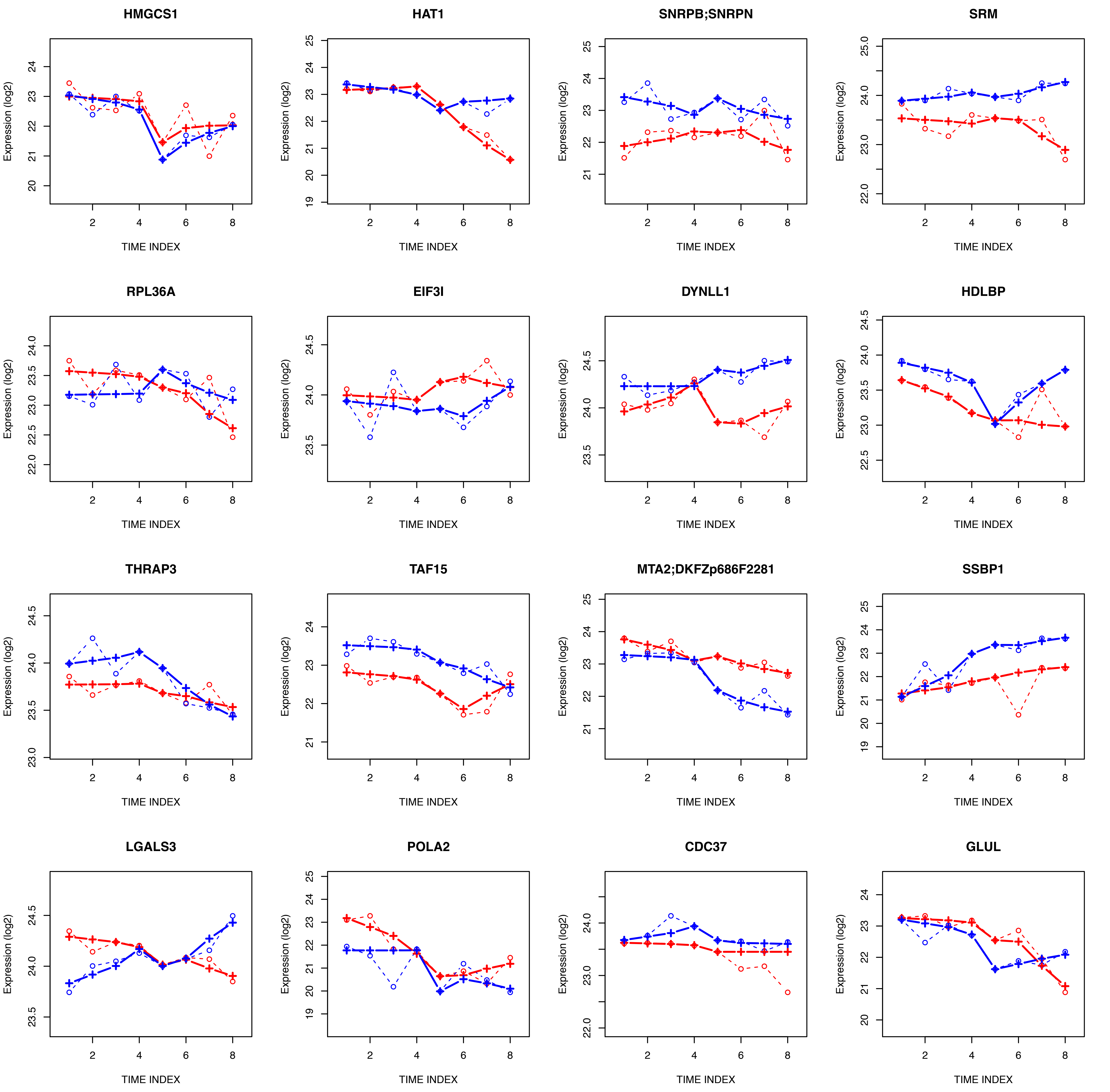
HMGCS1
HAT1
SNRPB;SNRPN
SRM
RPL36A
EIF3I
DYNLL1
HDLBP
THRAP3
TAF15
MTA2;DKFZp686F2281
SSBP1
LGALS3
POLA2
CDC37
GLUL
Expression (log2)
TIME INDEX

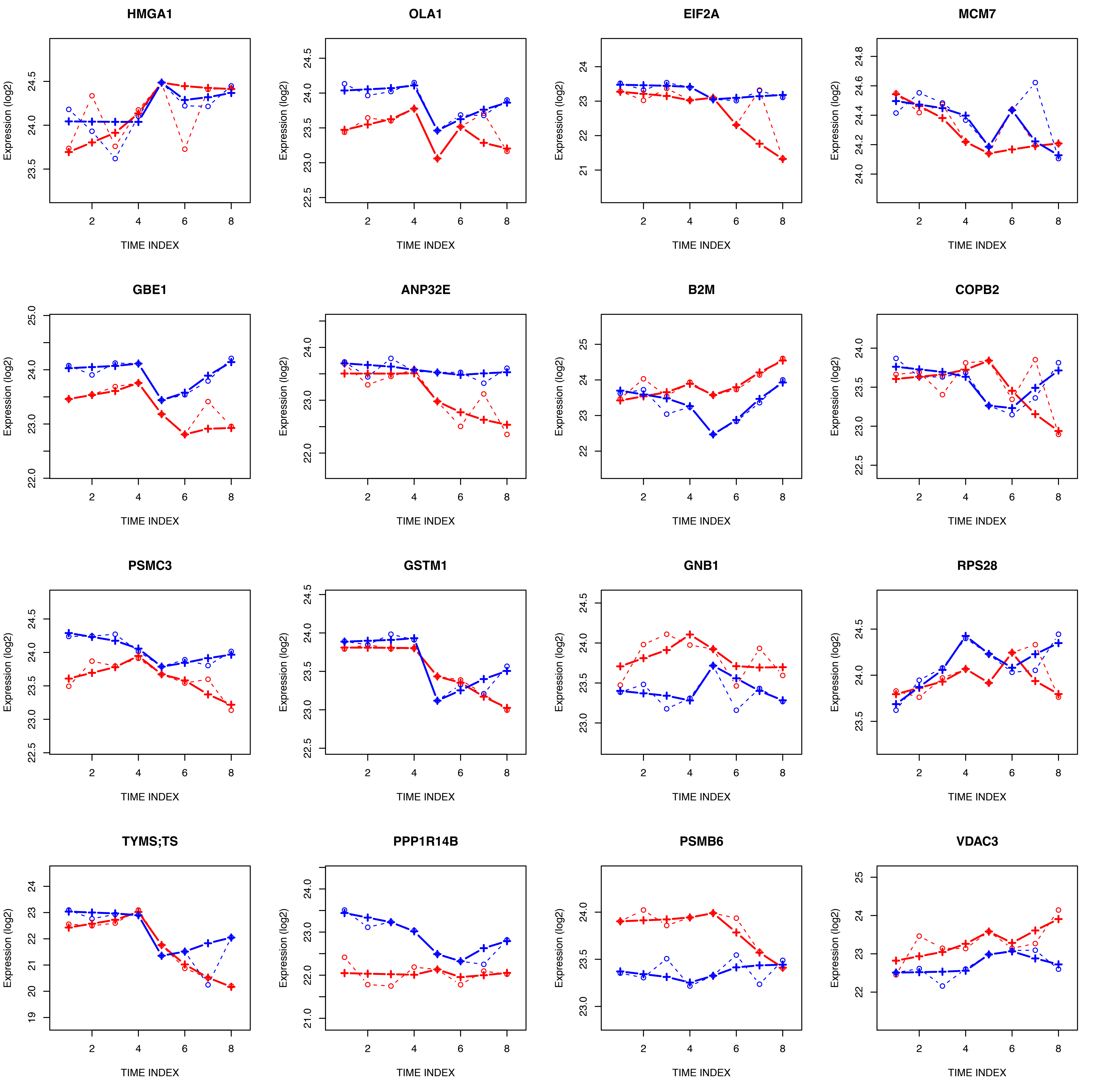

HMGA1
OLA1
EIF2A
MCM7
GBE1
ANP32E
B2M
COPB2
PSMC3
GSTM1
GNB1
RPS28
TYMS;TS
PPP1R14B
PSMB6
VDAC3
Expression (log2)
TIME INDEX

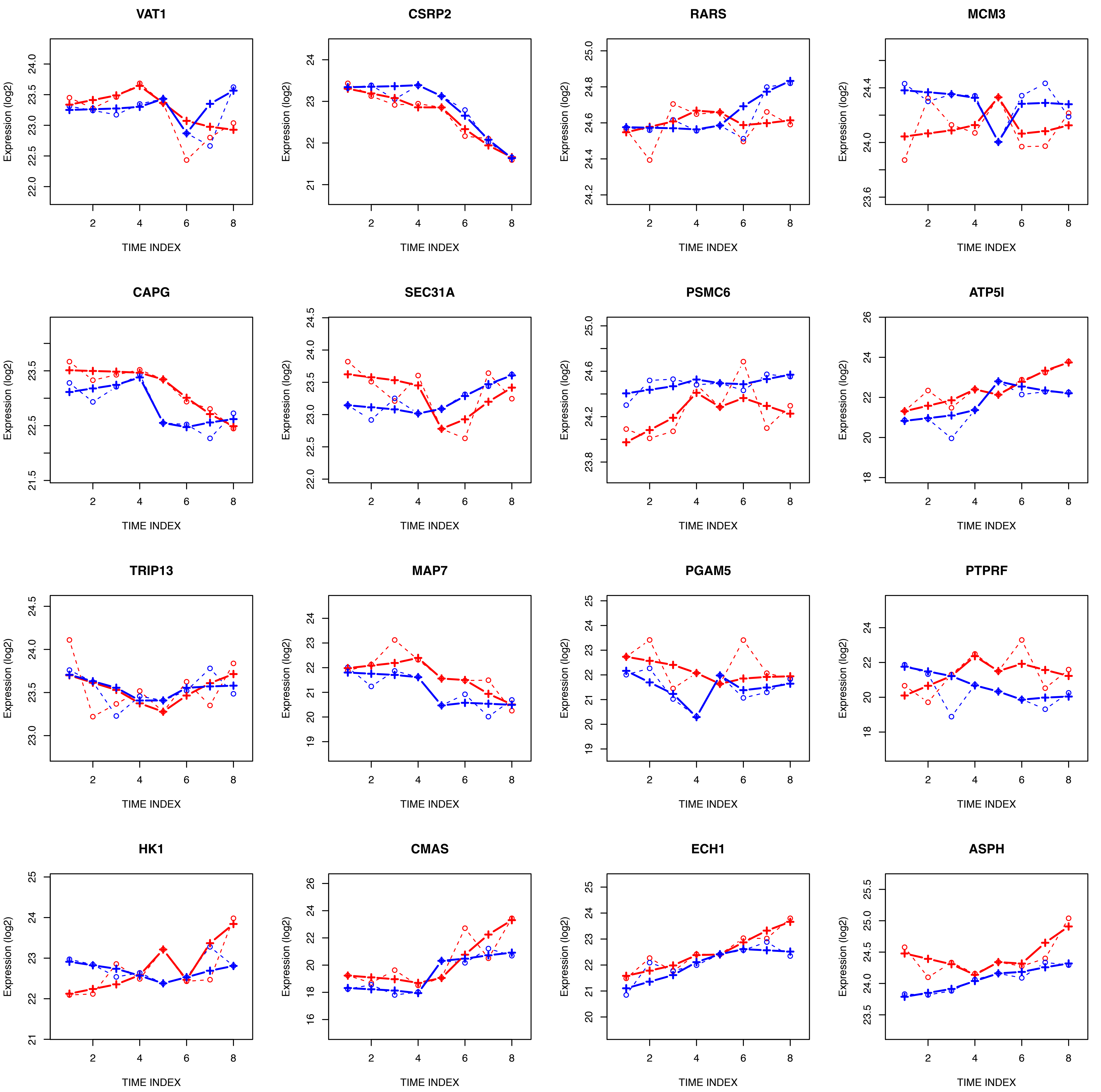
VAT1
CSRP2
RARS
MCM3
CAPG
SEC31A
PSMC6
ATP5I
TRIP13
MAP7
PGAM5
PTPRF
HK1
CMAS
ECH1
ASPH
Expression (log2)
TIME INDEX

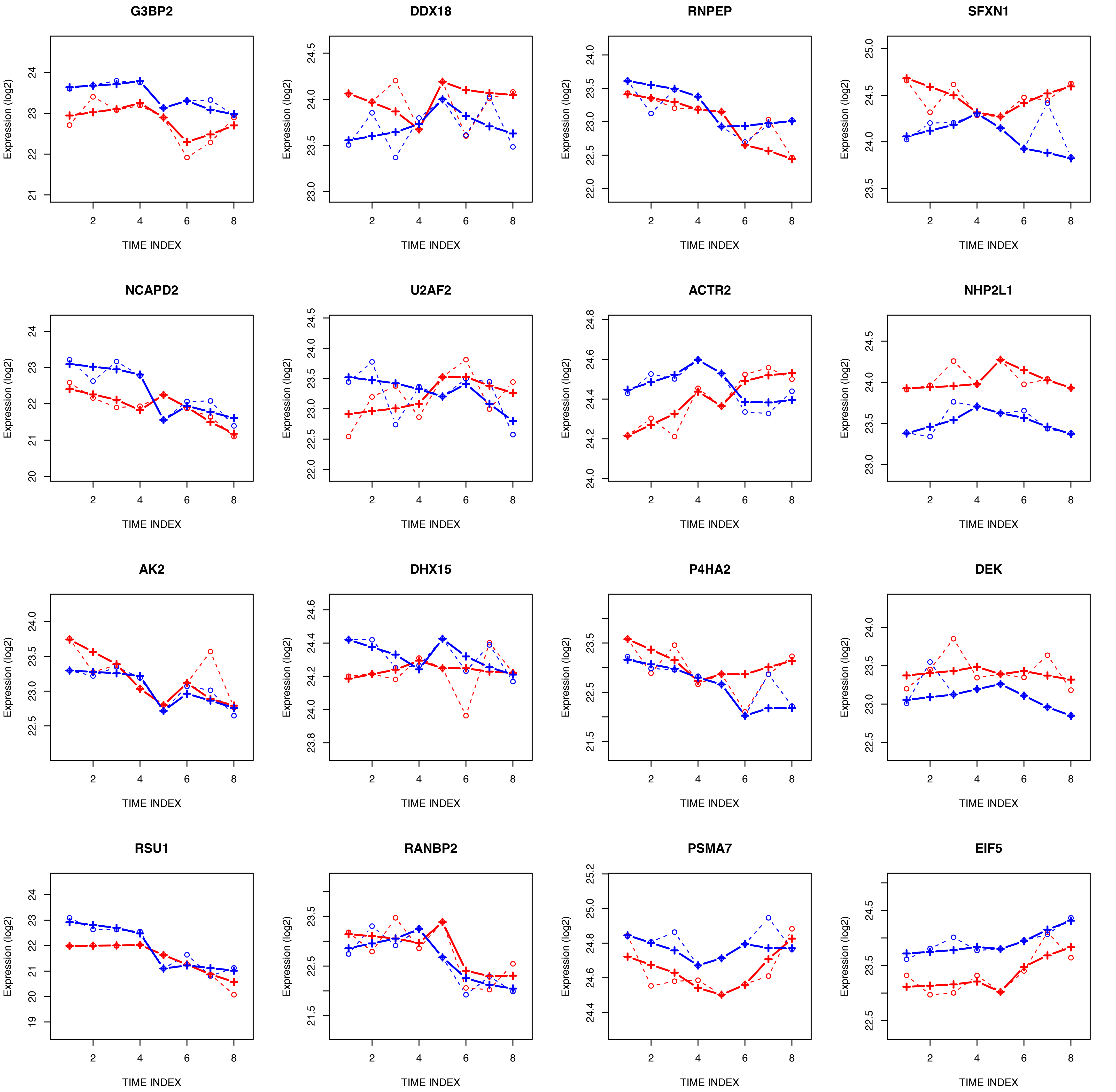
G3BP2
DDX18
RNPEP
SFXN1
NCAPD2
U2AF2
ACTR2
NHP2L1
AK2
DHX15
P4HA2
DEK
RSU1
RANBP2
PSMA7
EIF5
Expression (log2)
TIME INDEX

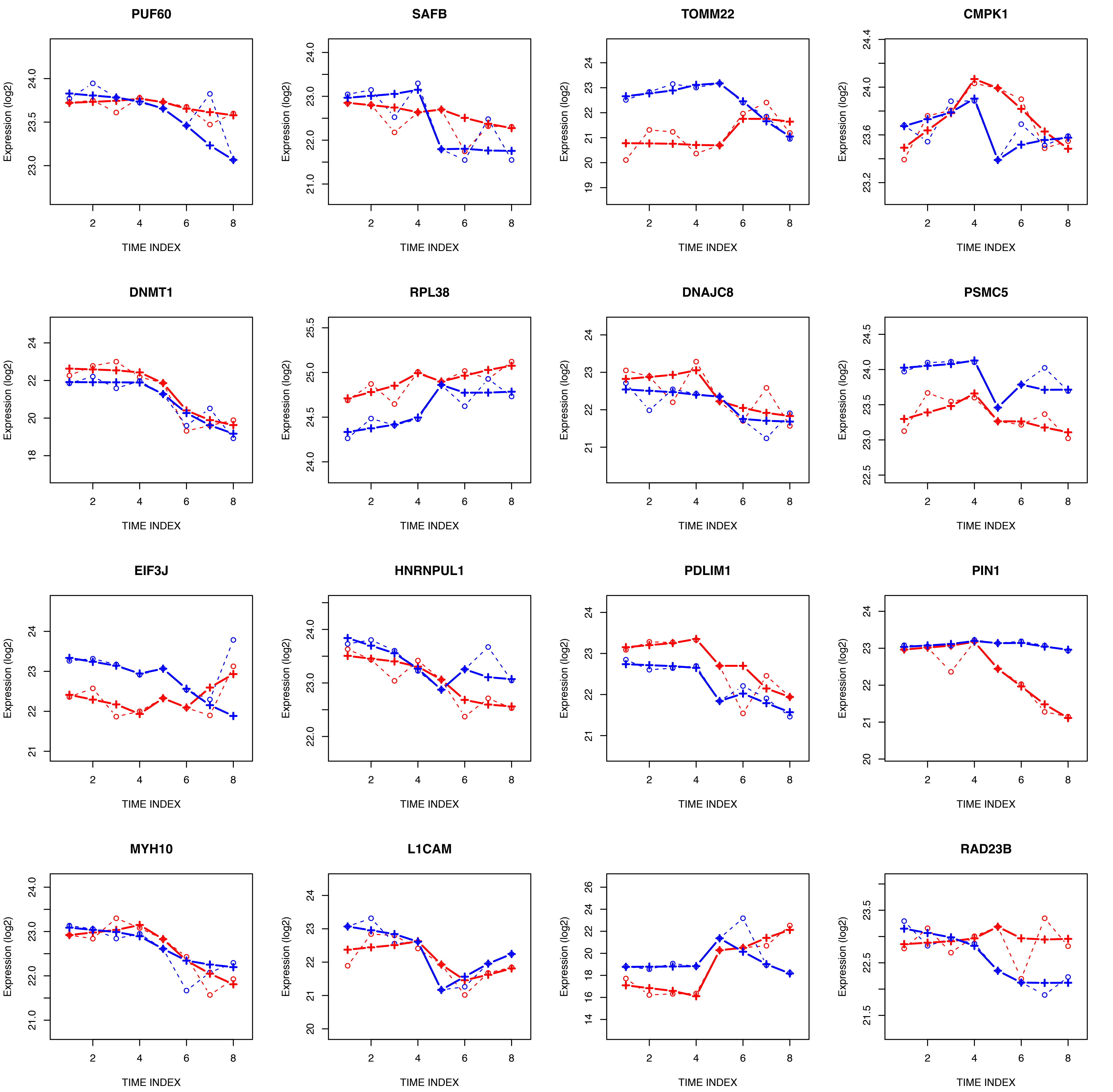
PUF60
SAFB
TOMM22
CMPK1
DNMT1
RPL38
DNAJC8
PSMC5
EIF3J
HNRNPUL1
PDLIM1
PIN1
MYH10
L1CAM
RAD23B
Expression (log2)
TIME INDEX

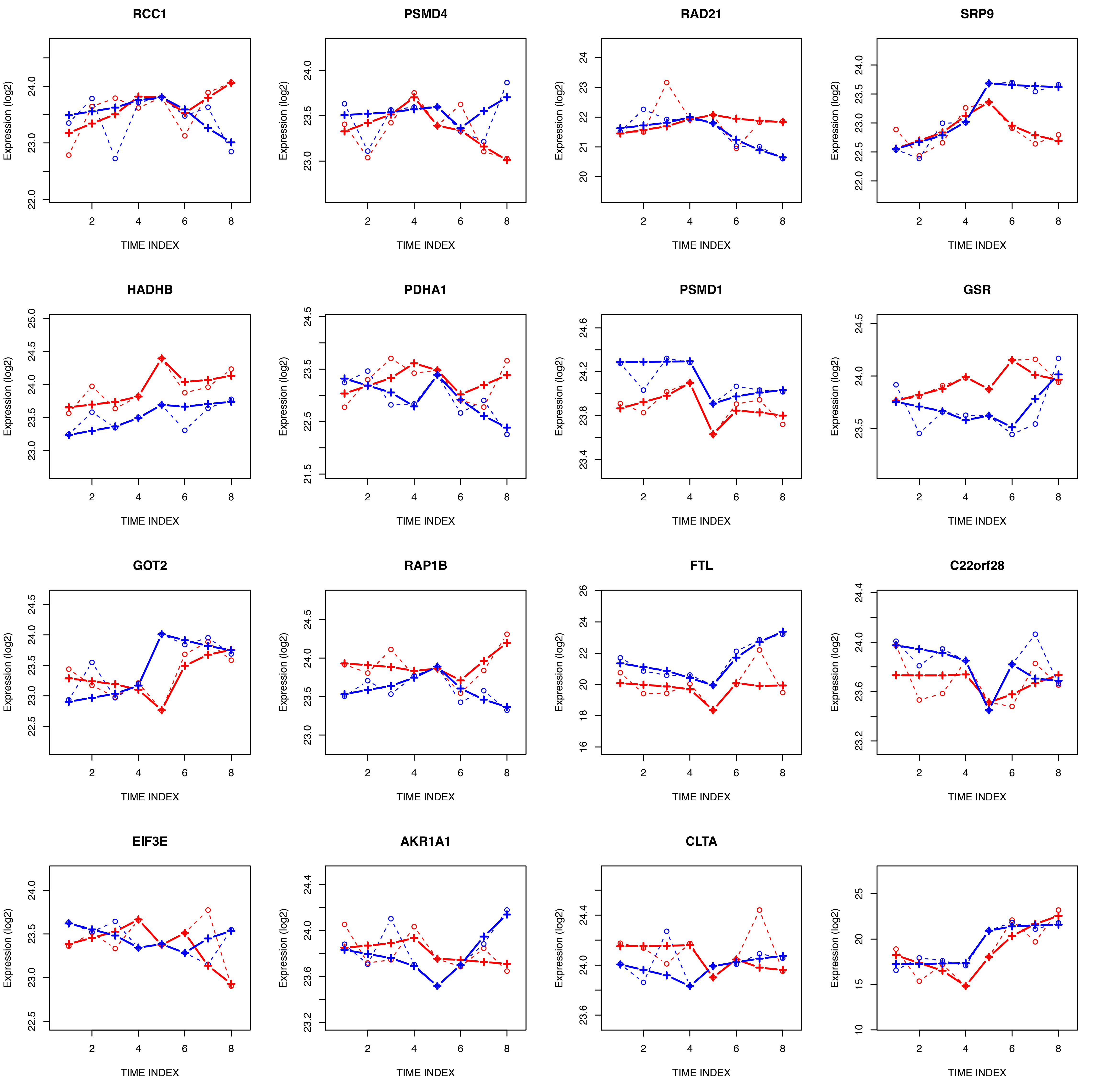
RCC1
PSMD4
RAD21
SRP9
HADHB
PDHA1
PSMD1
GSR
GOT2
RAP1B
FTL
C22orf28
EIF3E
AKR1A1
CLTA
Expression (log2)
TIME INDEX
2 4 6 8
22.0 23.0 24.0
23.0 23.5 24.0
20 21 22 23 24
22.0 22.5 23.0 23.5 24.0
23.0 23.5 24.0 24.5 25.0
21.5 22.5 23.5 24.5
23.4 23.8 24.2 24.6
23.5 24.0 24.5
22.5 23.0 23.5 24.0 24.5
23.0 23.5 24.0 24.5
16 18 20 22 24 26
23.2 23.6 24.0 24.4
22.5 23.0 23.5 24.0
23.2 23.6 24.0 24.4
23.6 24.0 24.4
10 15 20 25

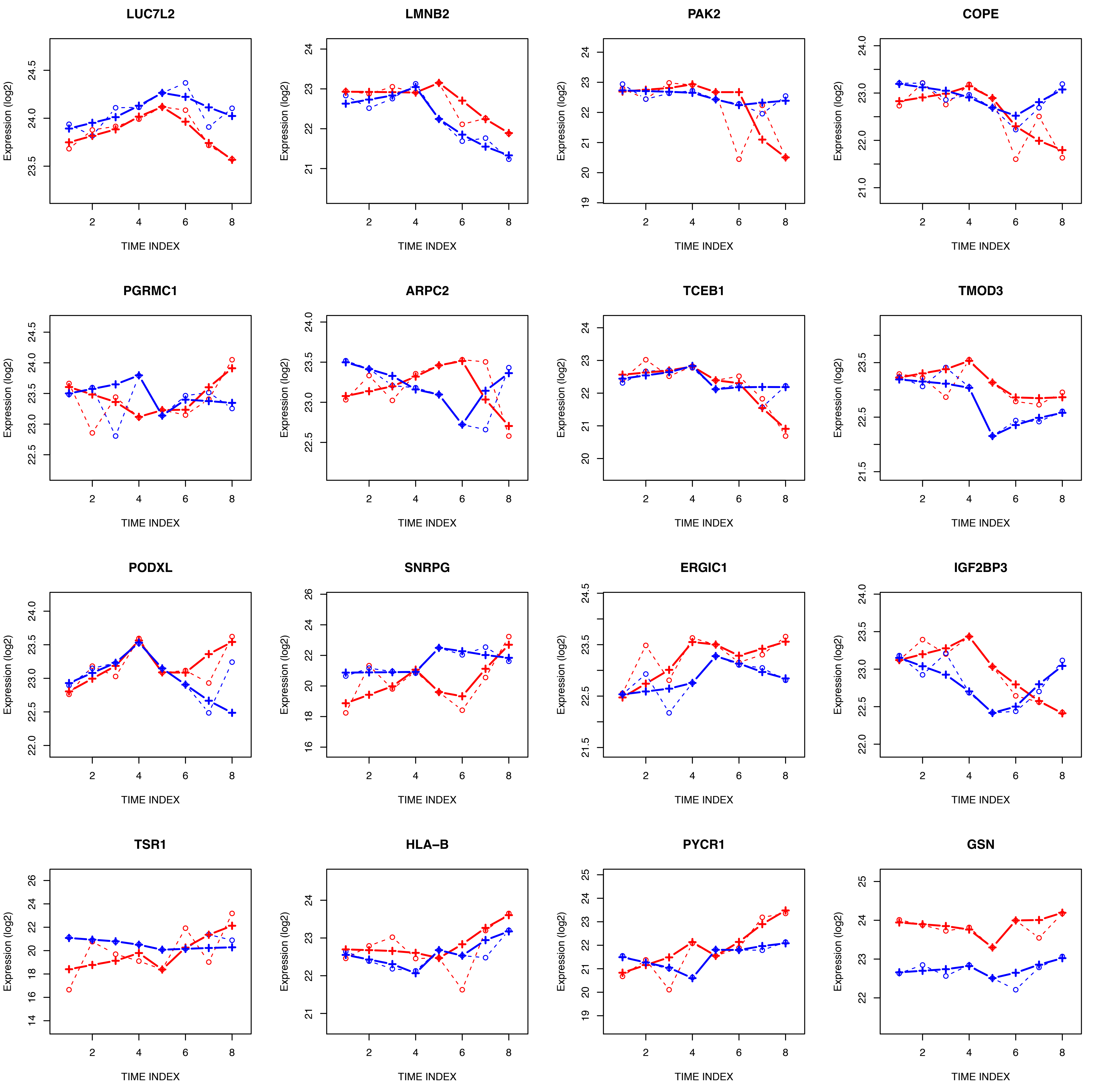
LUC7L2
LMNB2
PAK2
COPE
PGRMC1
ARPC2
TCEB1
TMOD3
PODXL
SNRPG
ERGIC1
IGF2BP3
TSR1
HLA–B
PYCR1
GSN
Expression (log2)
TIME INDEX

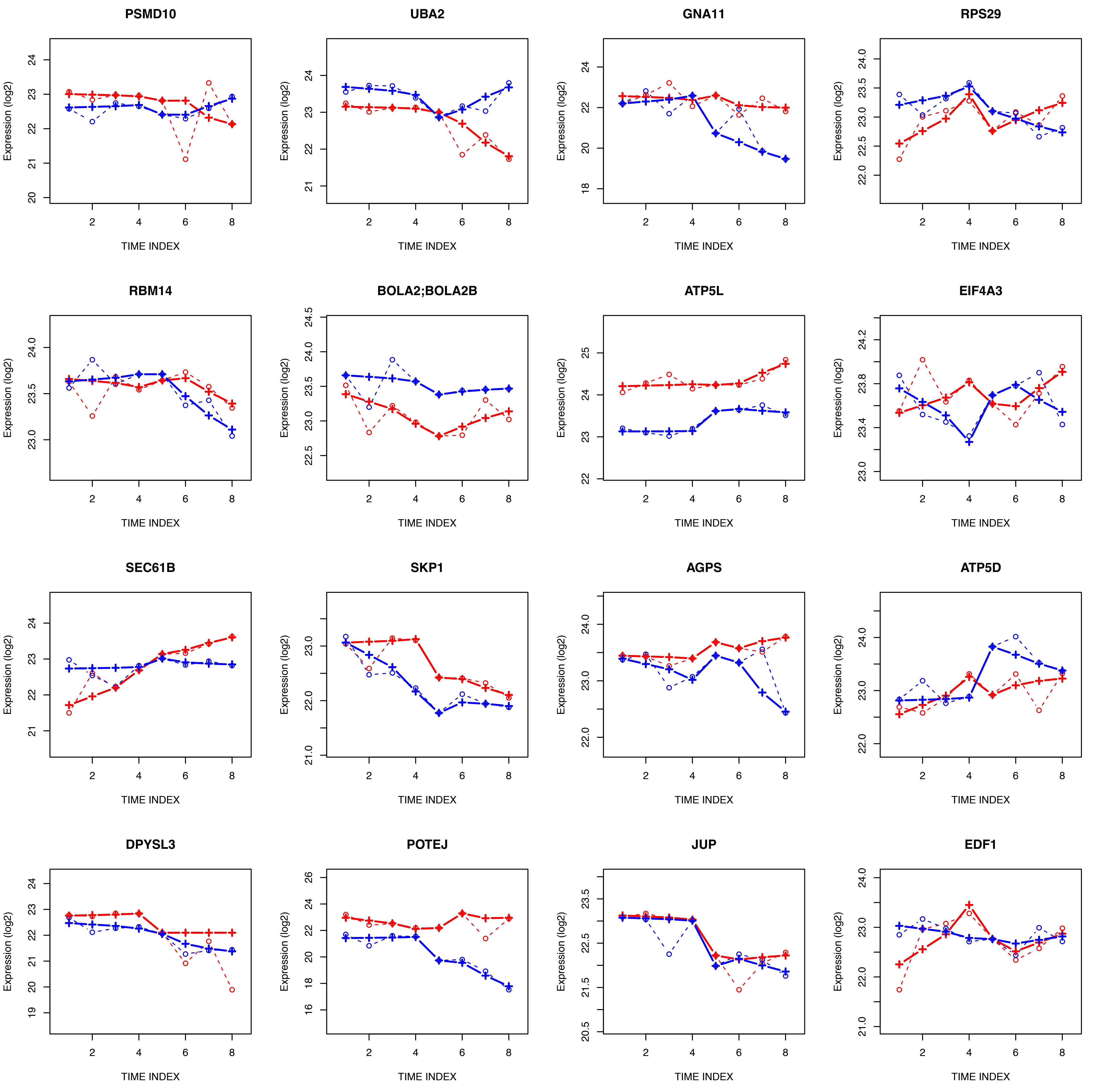
PSMD10
UBA2
GNA11
RPS29
RBM14
BOLA2;BOLA2B
ATP5L
EIF4A3
SEC61B
SKP1
AGPS
ATP5D
DPYSL3
POTEJ
JUP
EDF1
Expression (log2)
TIME INDEX

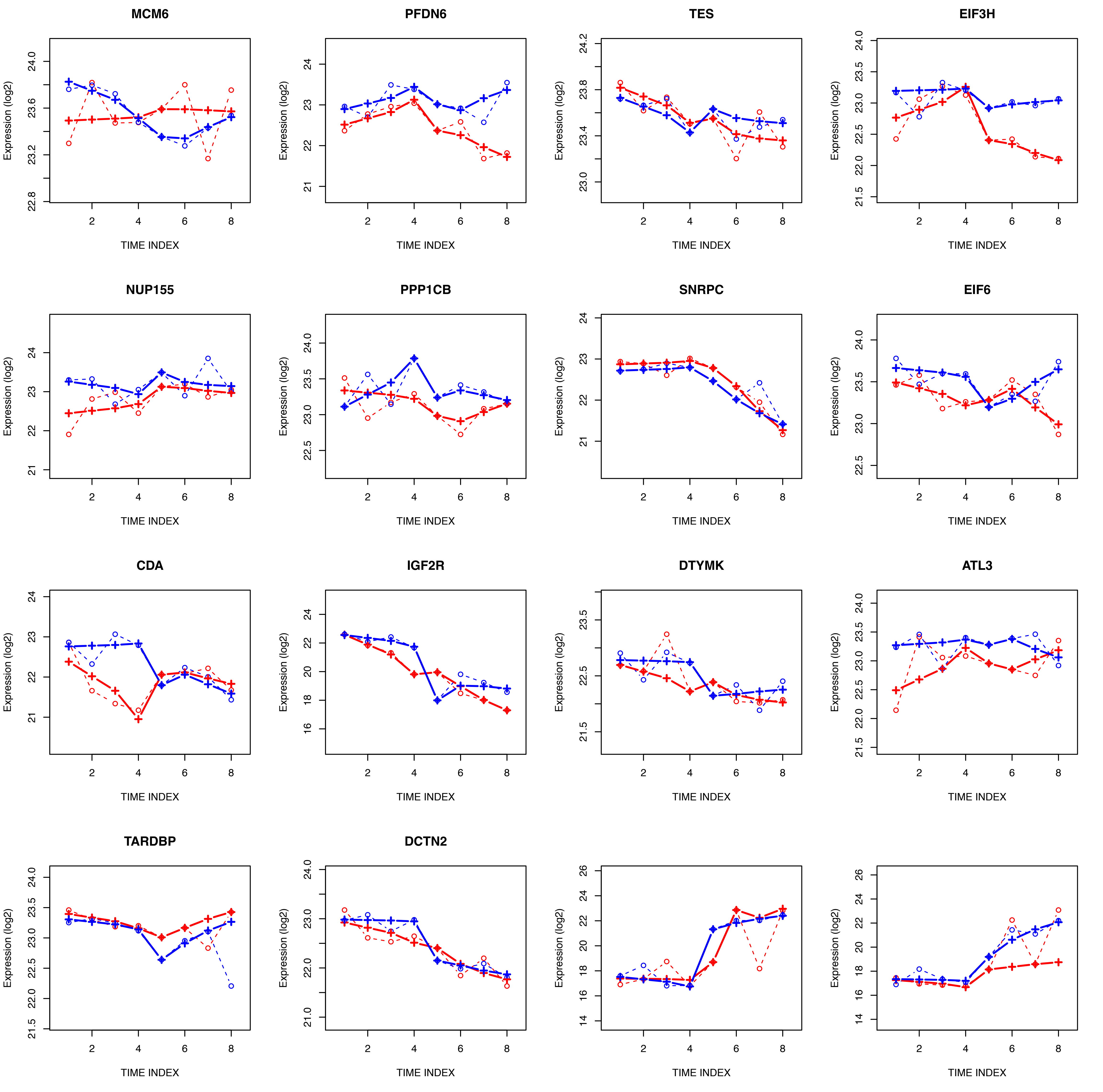
MCM6
PFDN6
TES
EIF3H
NUP155
PPP1CB
SNRPC
EIF6
CDA
IGF2R
DTYMK
ATL3
TARDBP
DCTN2
Expression (log2)
TIME INDEX

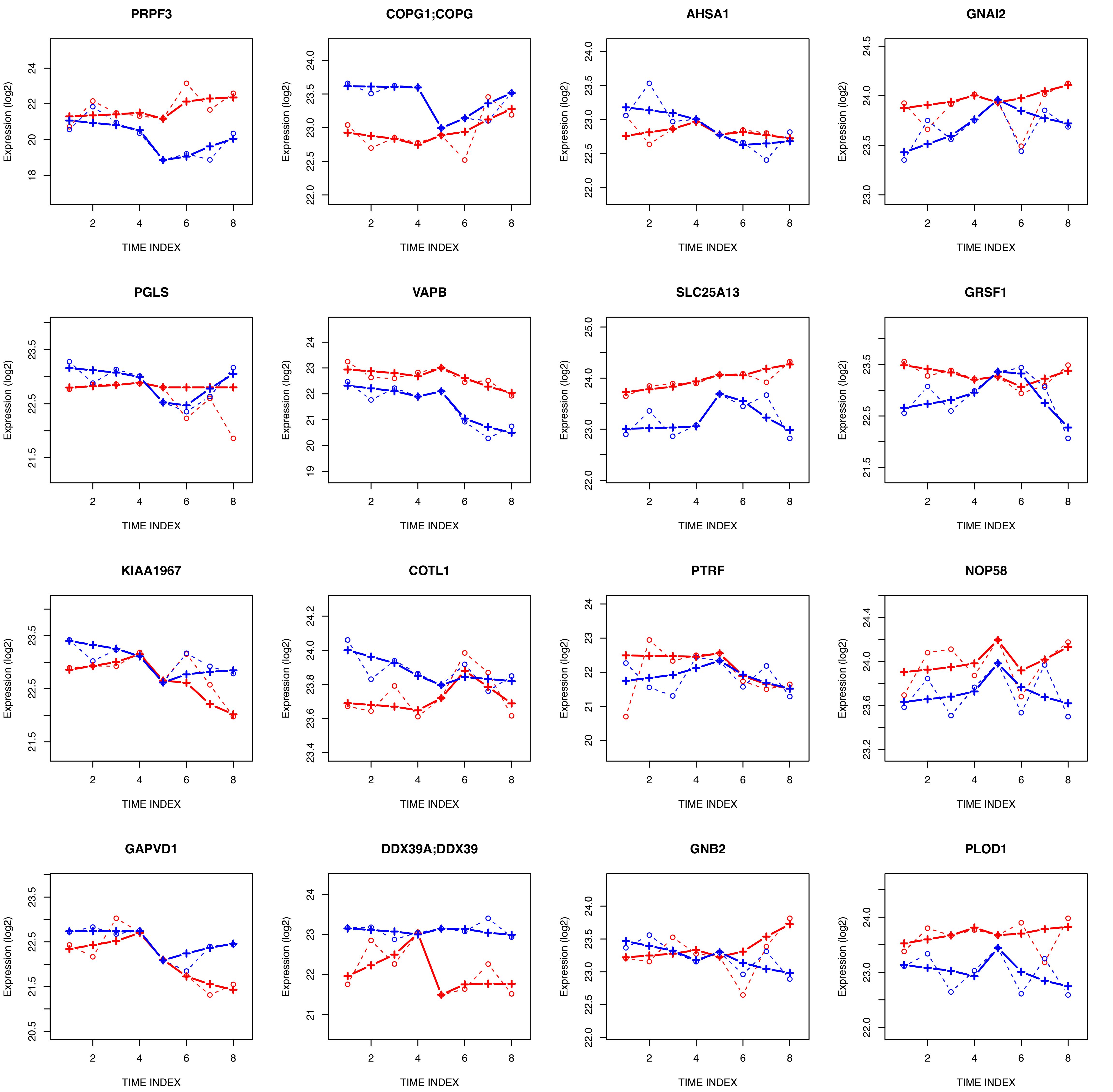
PRPF3
COPG1;COPG
AHSA1
GNAI2
PGLS
VAPB
SLC25A13
GRSF1
KIAA1967
COTL1
PTRF
NOP58
GAPVD1
DDX39A;DDX39
GNB2
PLOD1
Expression (log2)
TIME INDEX
2
4
6
8

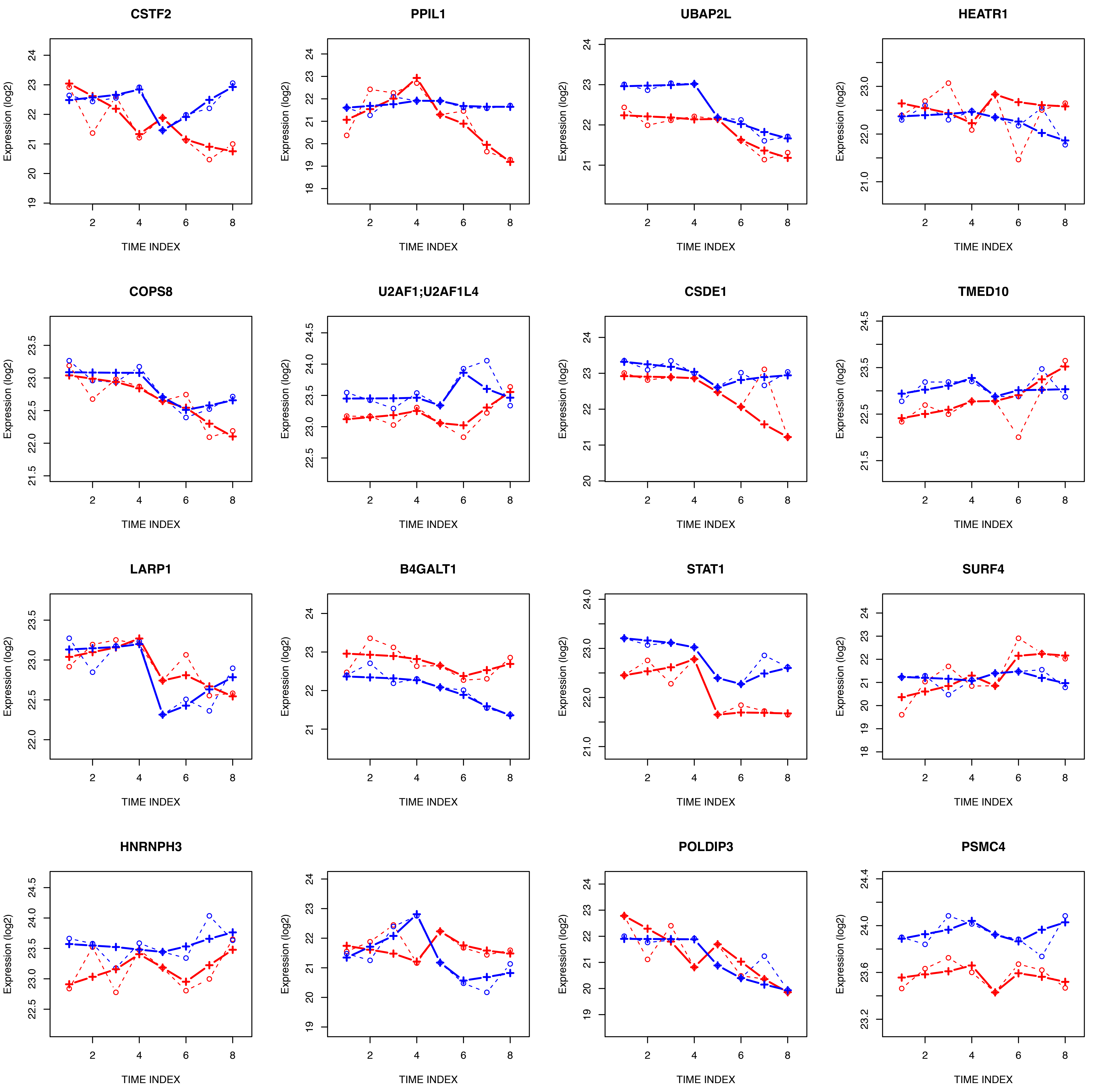
CSTF2
PPIL1
UBAP2L
HEATR1
COPS8
U2AF1;U2AF1L4
CSDE1
TMED10
LARP1
B4GALT1
STAT1
SURF4
HNRNPH3
POLDIP3
PSMC4
Expression (log2)
TIME INDEX

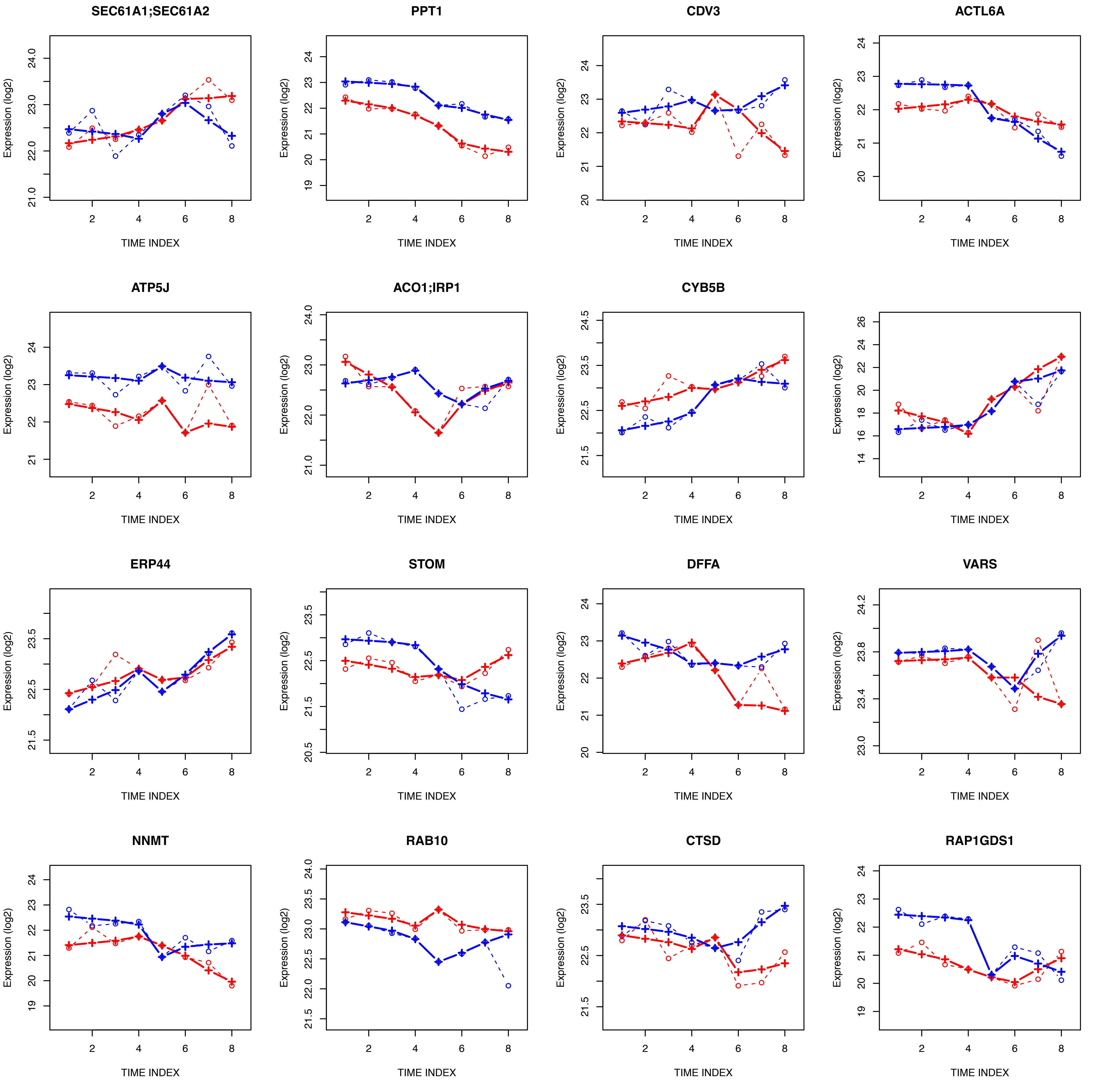

SEC61A1;SEC61A2
PPT1
CDV3
ACTL6A
ATP5J
ACO1;IRP1
CYB5B
ERP44
STOM
DFFA
VARS
NNMT
RAB10
CTSD
RAP1GDS1
Expression (log2)
TIME INDEX

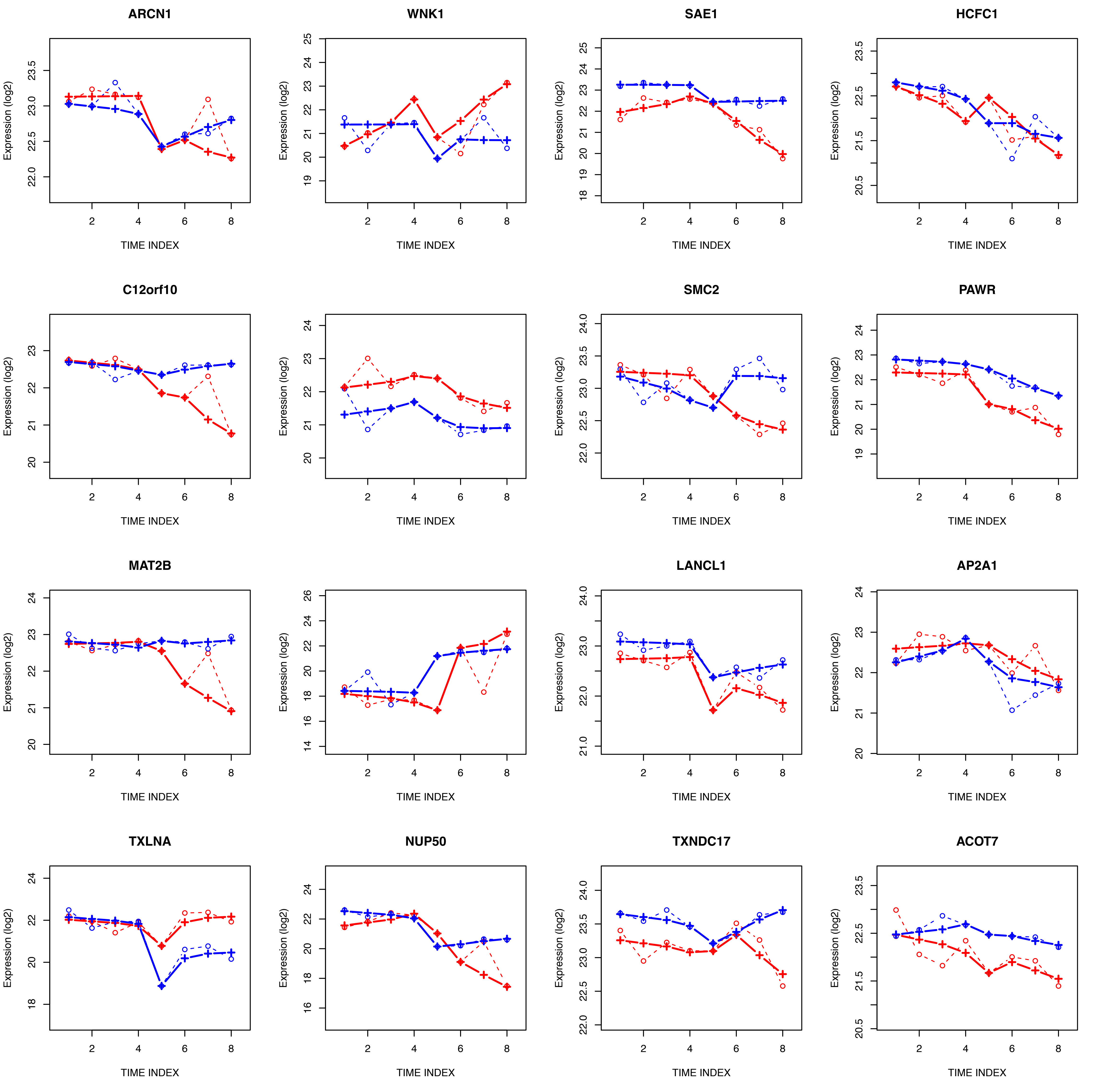
ARCN1
WNK1
SAE1
HCFC1
C12orf10
SMC2
PAWR
MAT2B
LANCL1
AP2A1
TXLNA
NUP50
TXNDC17
ACOT7
Expression (log2)
TIME INDEX

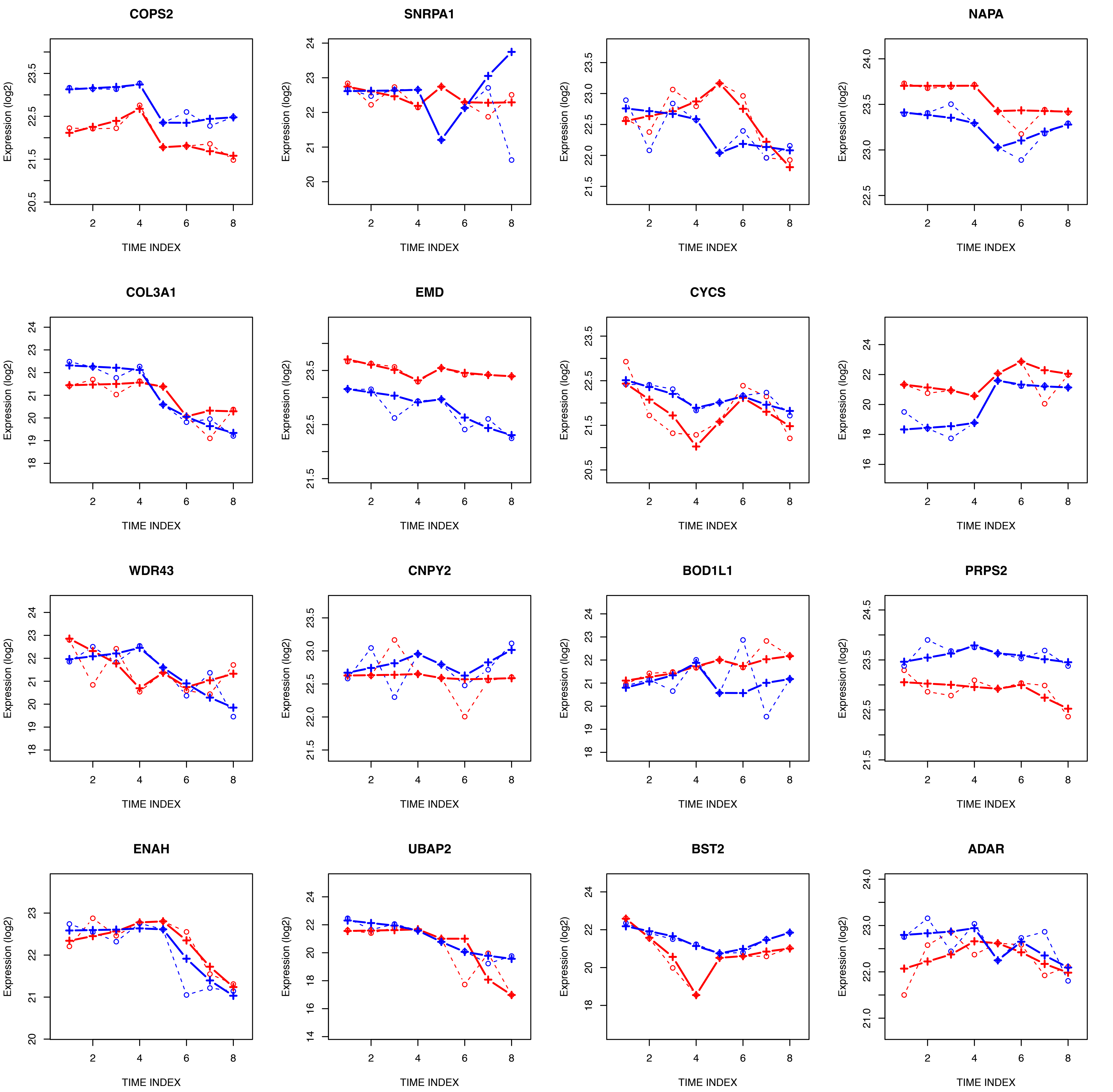
COPS2
SNRPA1
NAPA
COL3A1
EMD
CYCS
WDR43
CNPY2
BOD1L1
PRPS2
ENAH
UBAP2
BST2
ADAR
Expression (log2)
TIME INDEX

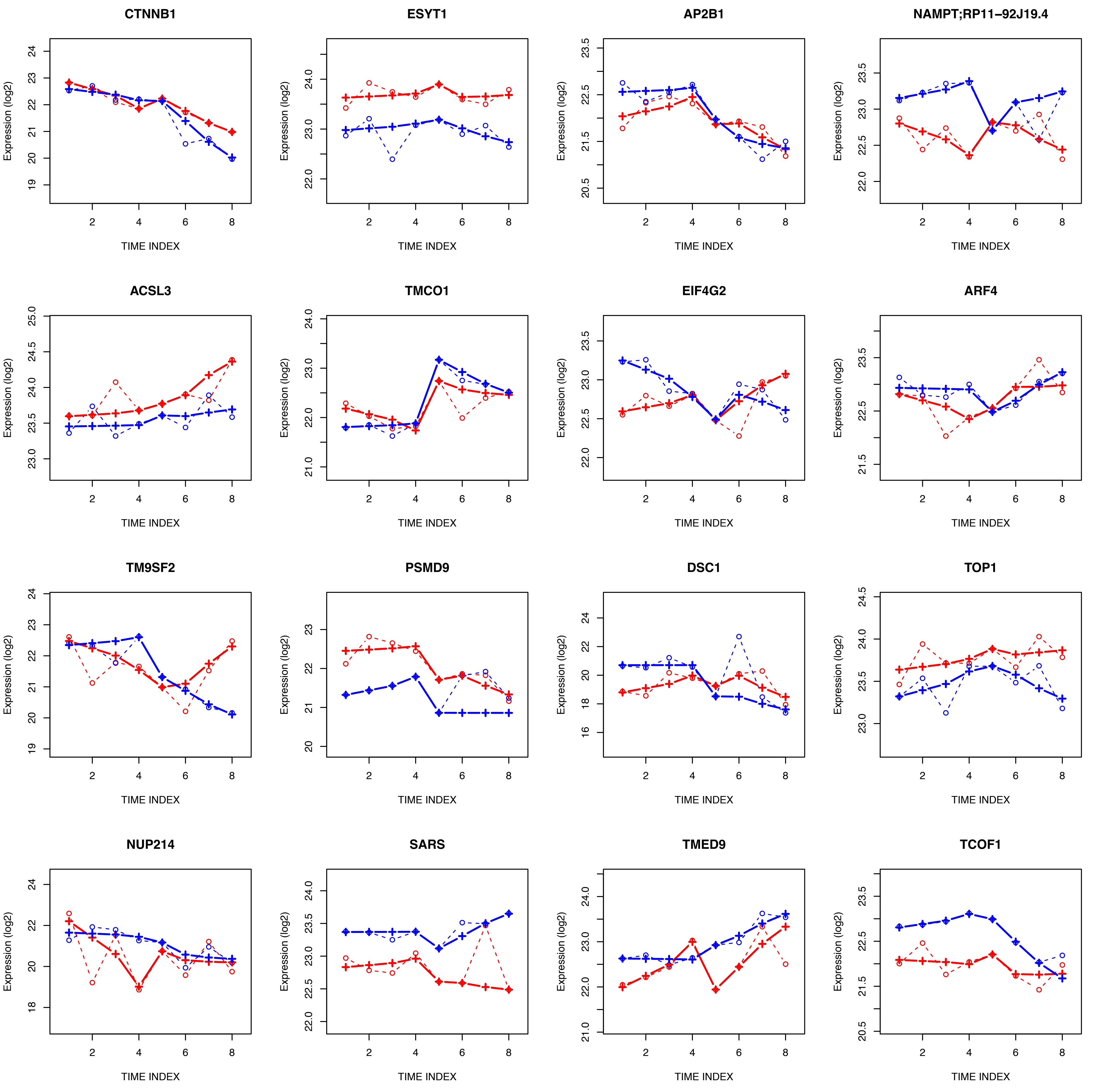
CTNNB1
ESYT1
AP2B1
NAMPT;RP11−92J19.4
ACSL3
TMCO1
EIF4G2
ARF4
TM9SF2
PSMD9
DSC1
TOP1
NUP214
SARS
TMED9
TCOF1
Expression (log2)
TIME INDEX

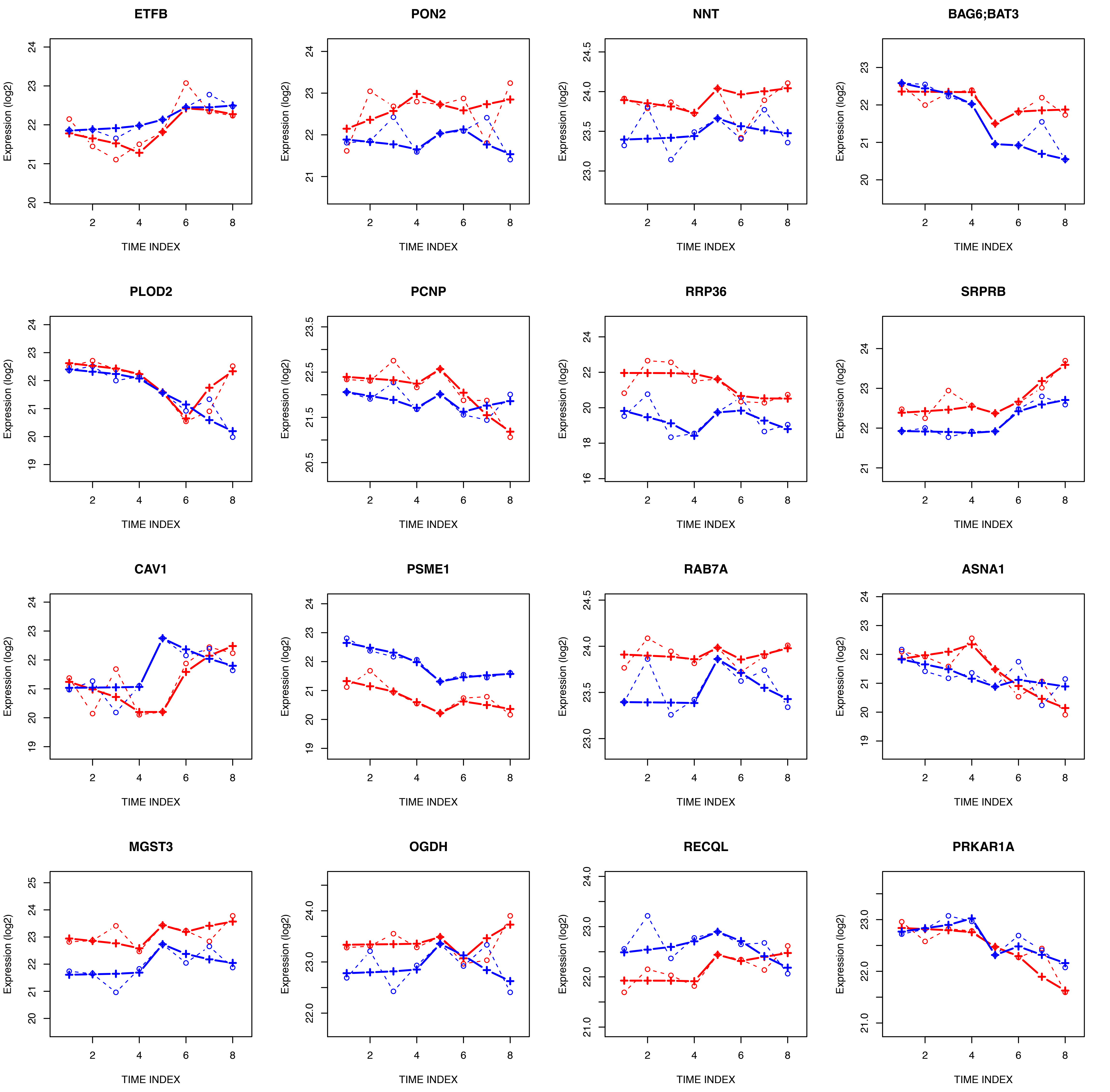

ETFB
PON2
NNT
BAG6;BAT3
PLOD2
PCNP
RRP36
SRPRB
CAV1
PSME1
RAB7A
ASNA1
MGST3
OGDH
RECQL
PRKAR1A
Expression (log2)
TIME INDEX

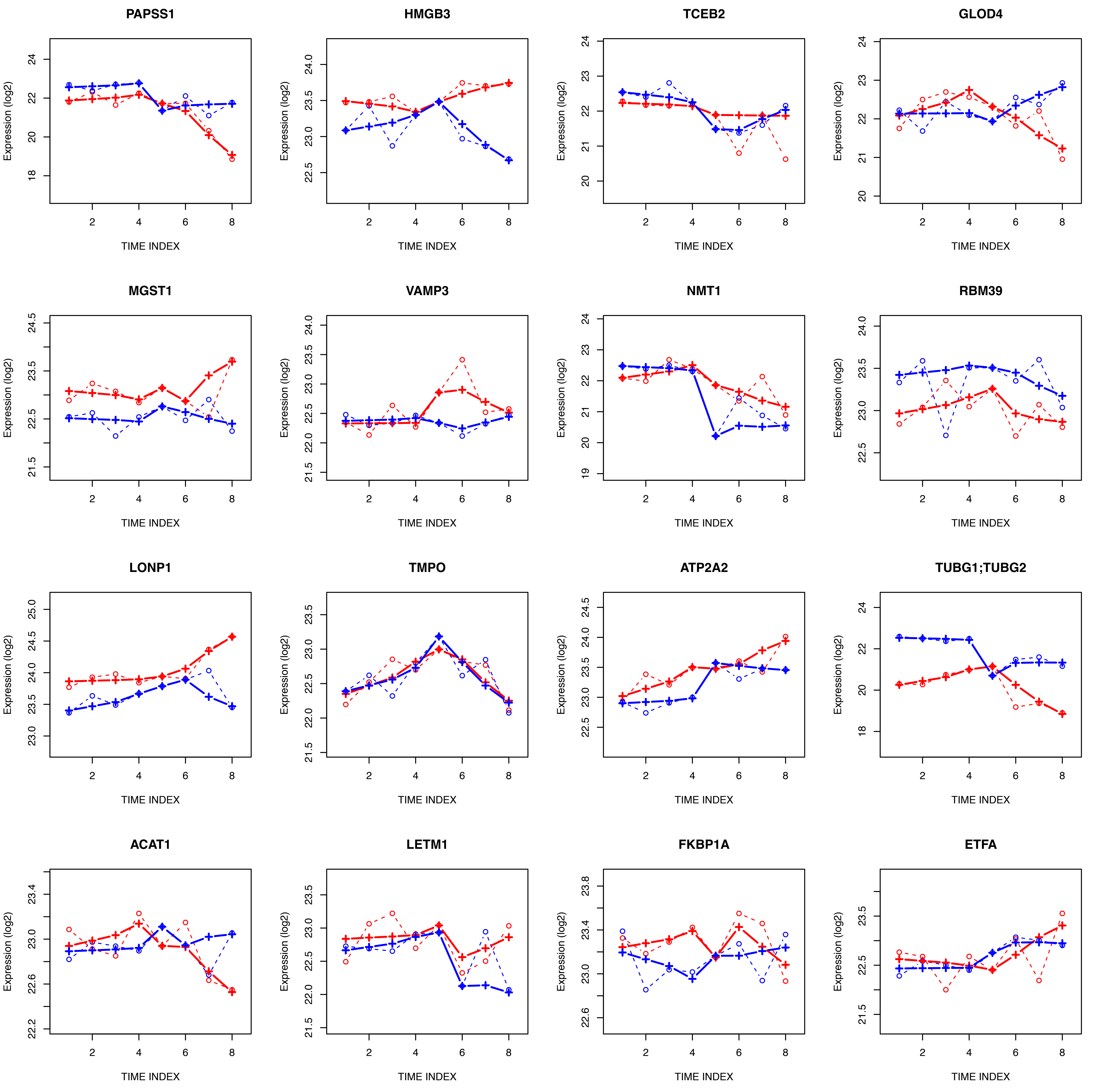
PAPSS1
HMGB3
TCEB2
GLOD4
MGST1
VAMP3
NMT1
RBM39
LONP1
TMPO
ATP2A2
TUBG1;TUBG2
ACAT1
LETM1
FKBP1A
ETFA
Expression (log2)
TIME INDEX
2
4
6
8
18
20
22
24
22.5
23.0
23.5
24.0
21
23
21.5
25.0
24.5
19
22.2
22.6
23.4
23.8

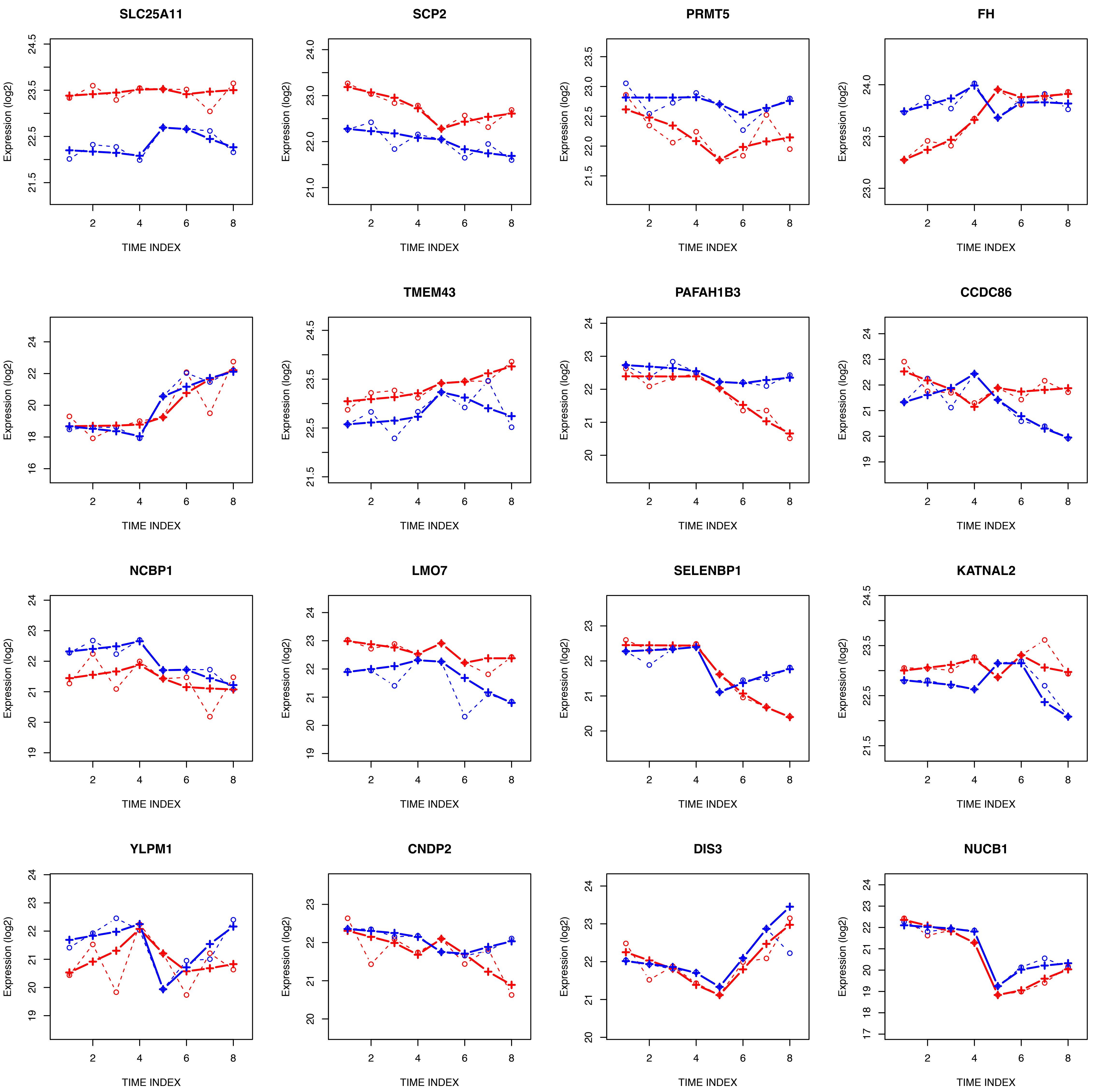
SLC25A11
SCP2
PRMT5
FH
TMEM43
PAFAH1B3
CCDC86
NCBP1
LMO7
SELENBP1
KATNAL2
YLPM1
CNDP2
DIS3
NUCB1
Expression (log2)
TIME INDEX

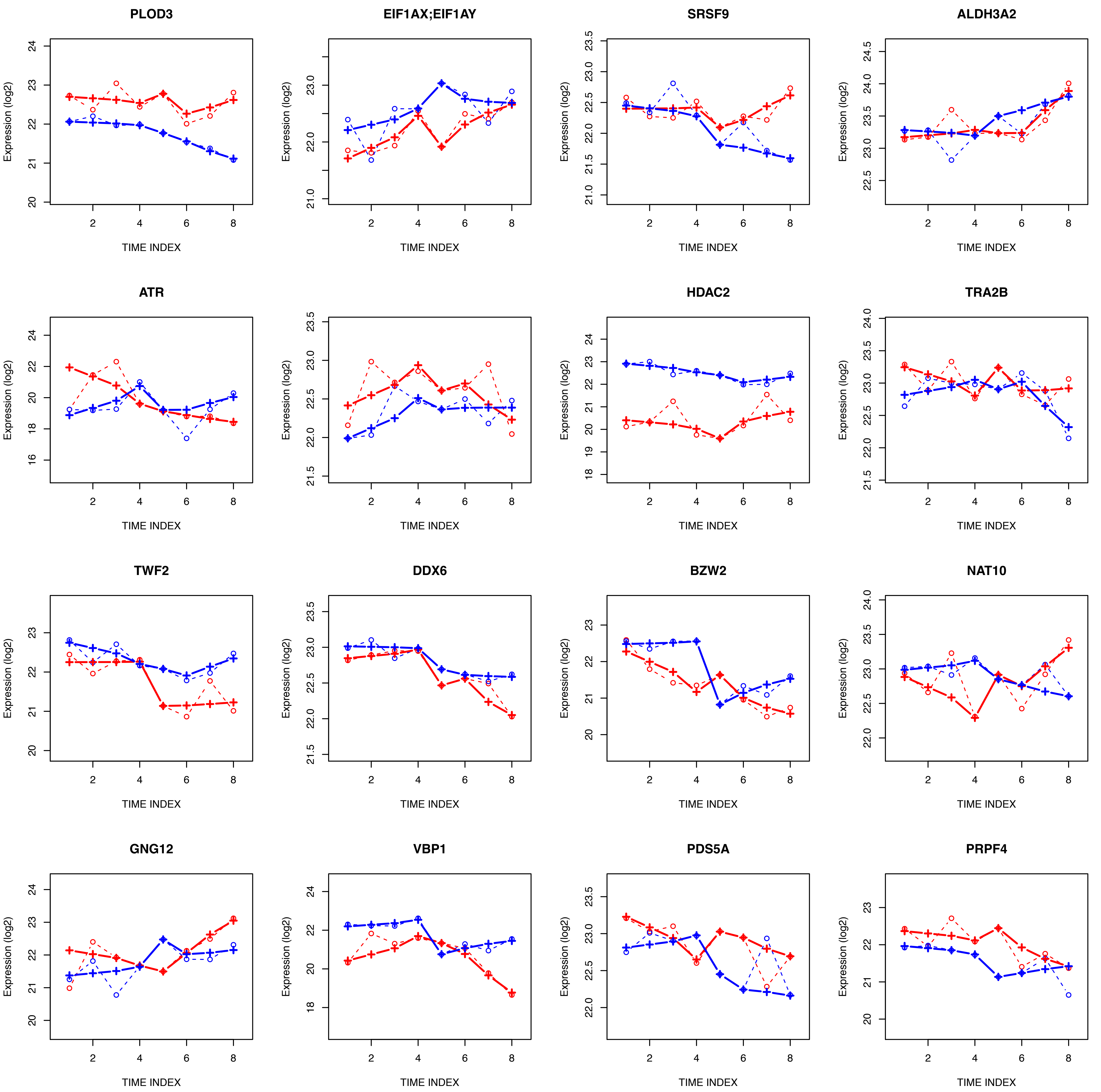

PLOD3
EIF1AX;EIF1AY
SRSF9
ALDH3A2
ATR
HDAC2
TRA2B
TWF2
DDX6
BZW2
NAT10
GNG12
VBP1
PDS5A
PRPF4
Expression (log2)
TIME INDEX
2
4
6
8

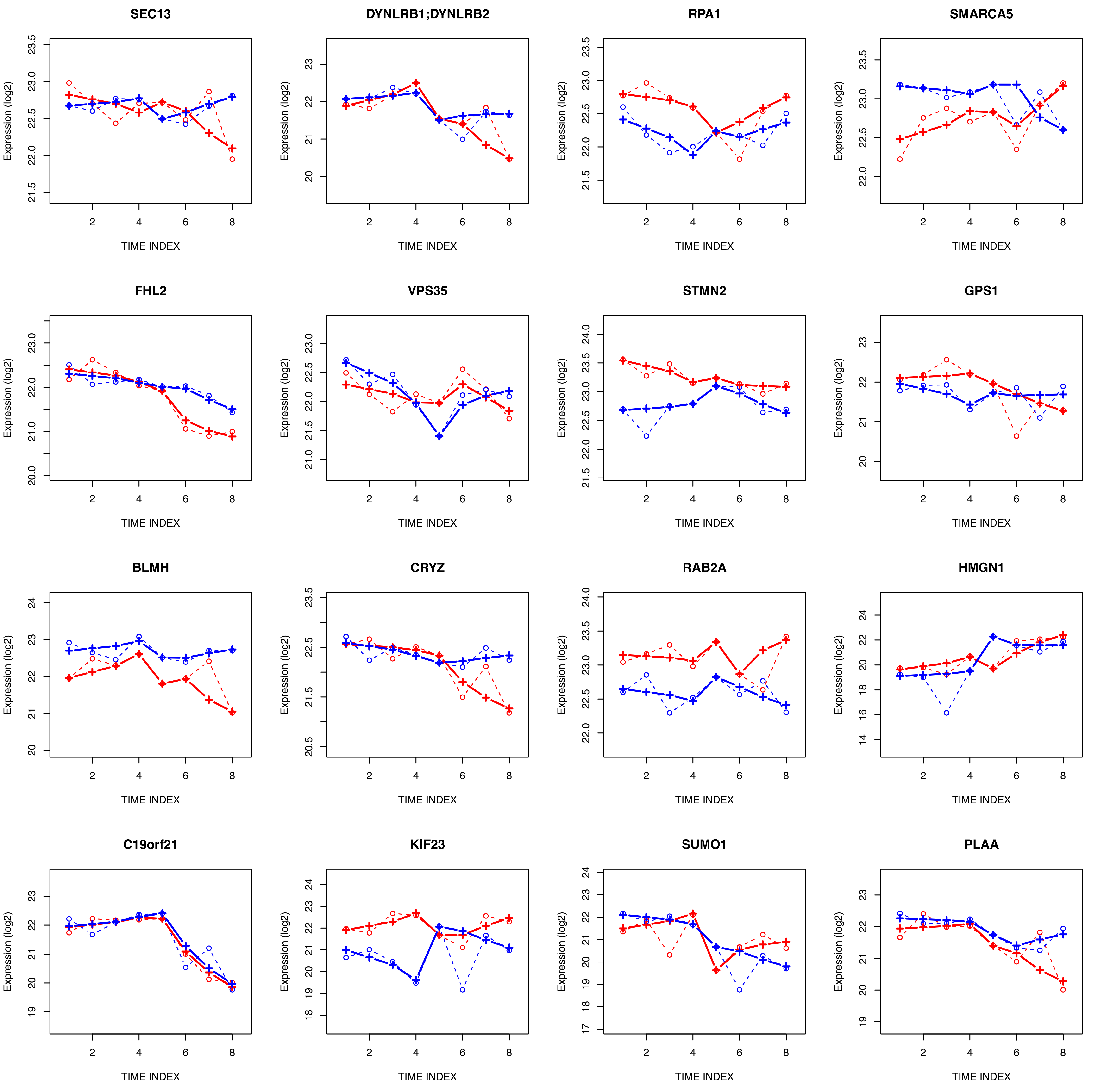
SEC13
DYNLRB1;DYNLRB2
RPA1
SMARCA5
FHL2
VPS35
STMN2
GPS1
BLMH
CRYZ
RAB2A
HMGN1
C19orf21
KIF23
SUMO1
PLAA
Expression (log2)
TIME INDEX

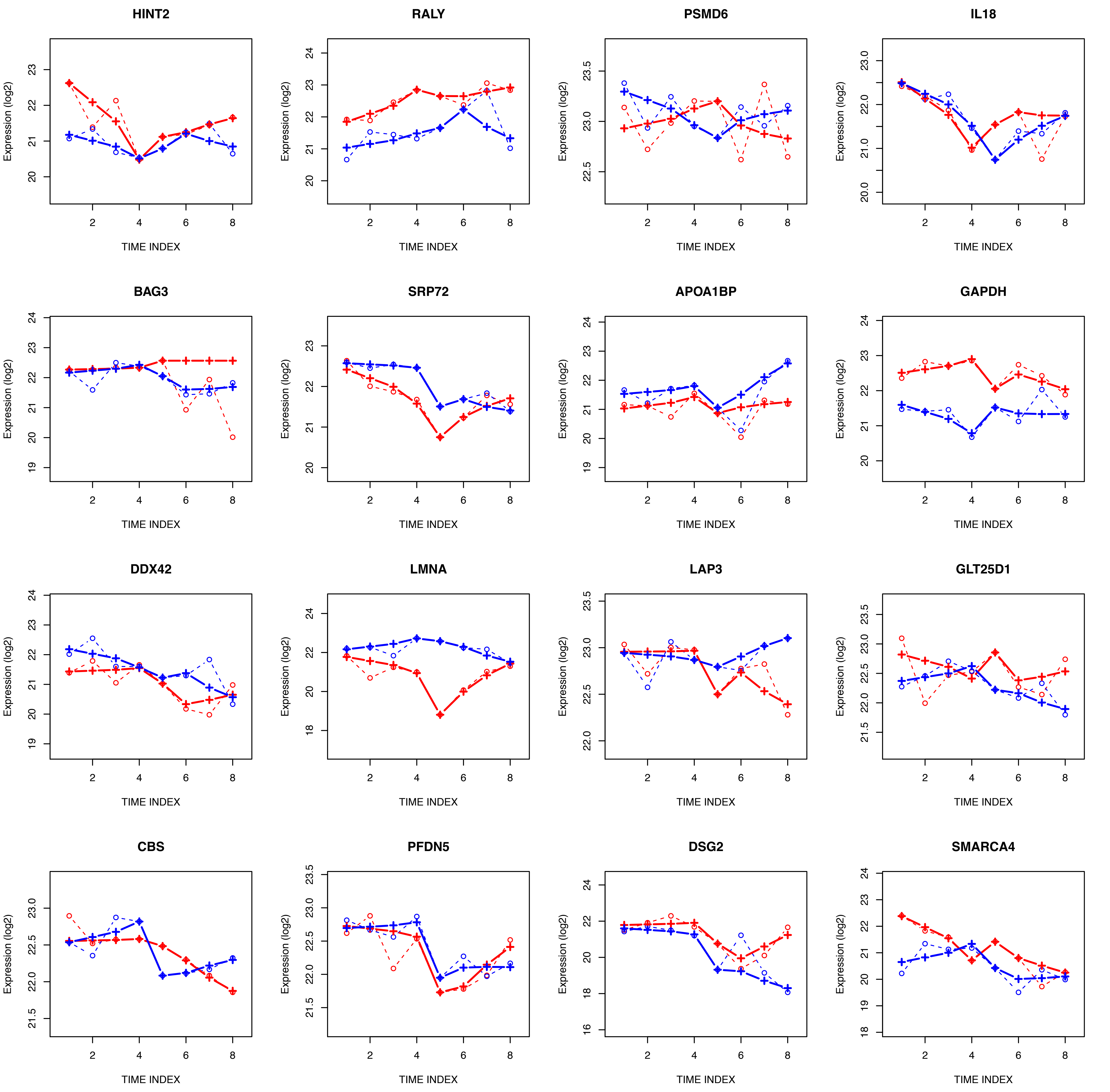
HINT2
RALY
PSMD6
IL18
BAG3
SRP72
APOA1BP
GAPDH
DDX42
LMNA
LAP3
GLT25D1
CBS
PFDN5
DSG2
SMARCA4
Expression (log2)
TIME INDEX

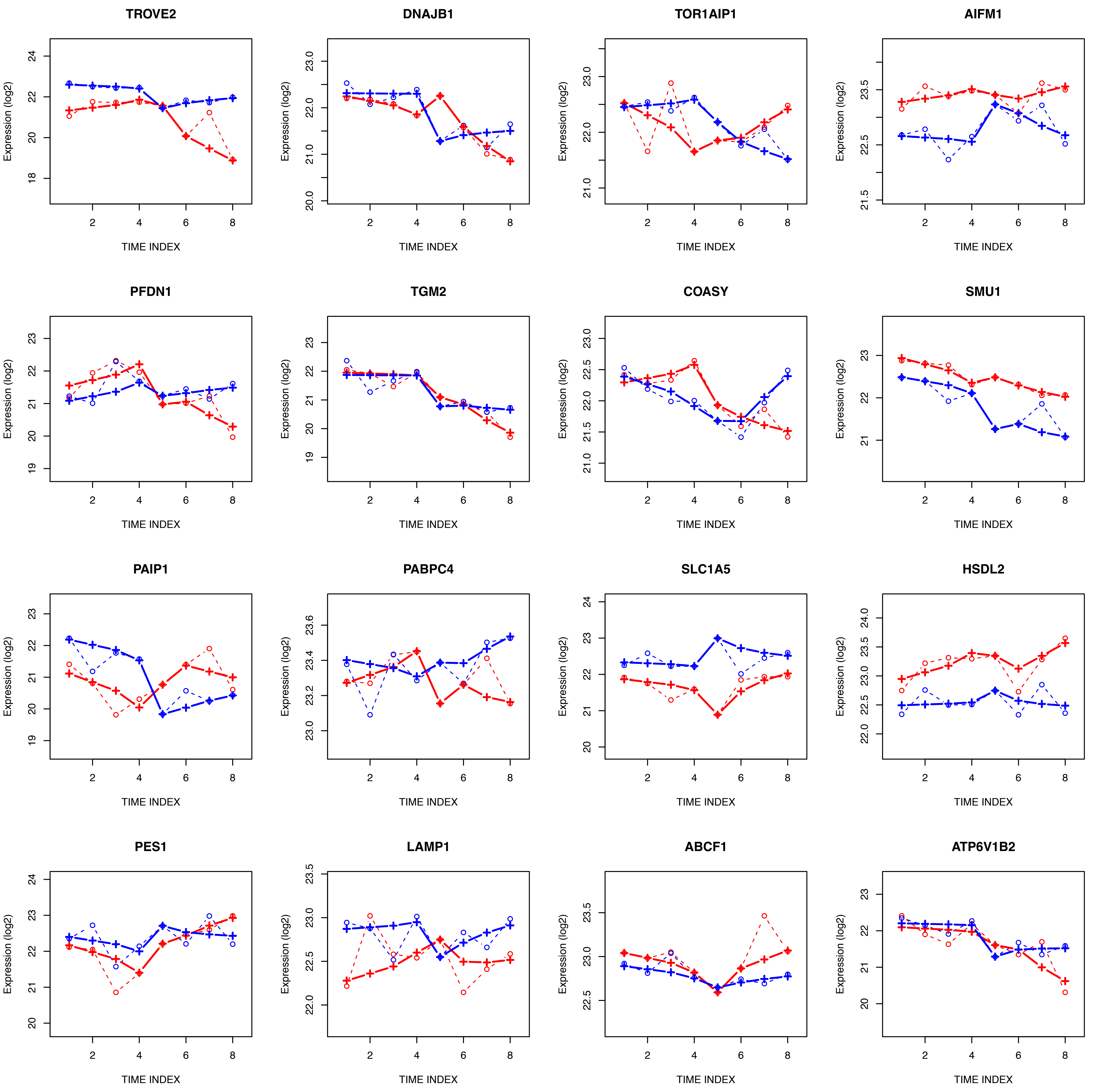
TROVE2
DNAJB1
TOR1AIP1
AIFM1
PFDN1
TGM2
COASY
SMU1
PAIP1
PABPC4
SLC1A5
HSDL2
PES1
LAMP1
ABCF1
ATP6V1B2
Expression (log2)
TIME INDEX

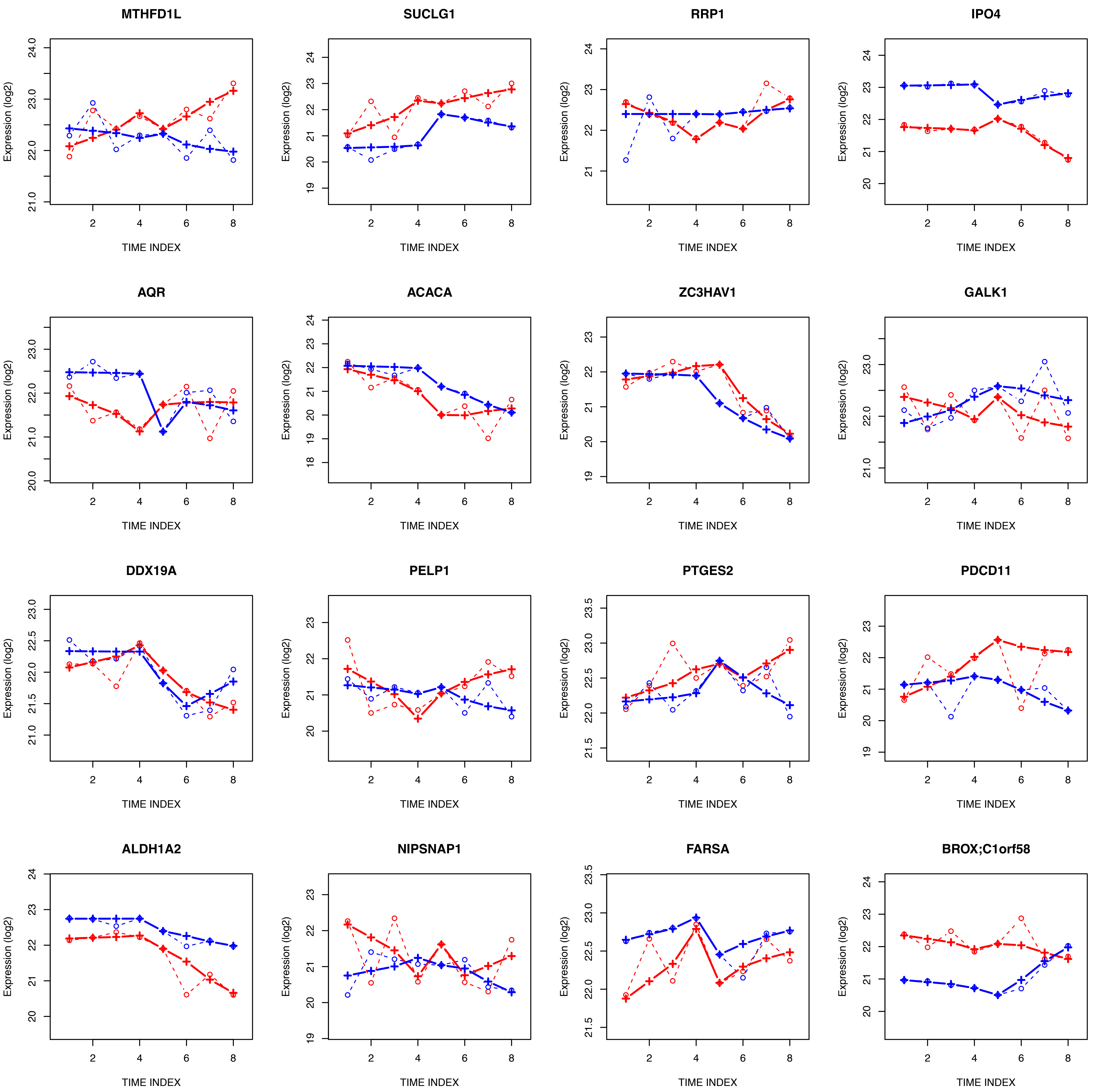
MTHFD1L
SUCLG1
RRP1
IPO4
AQR
ACACA
ZC3HAV1
GALK1
DDX19A
PELP1
PTGES2
PDCD11
ALDH1A2
NIPSNAP1
FARSA
BROX;C1orf58
Expression (log2)
TIME INDEX

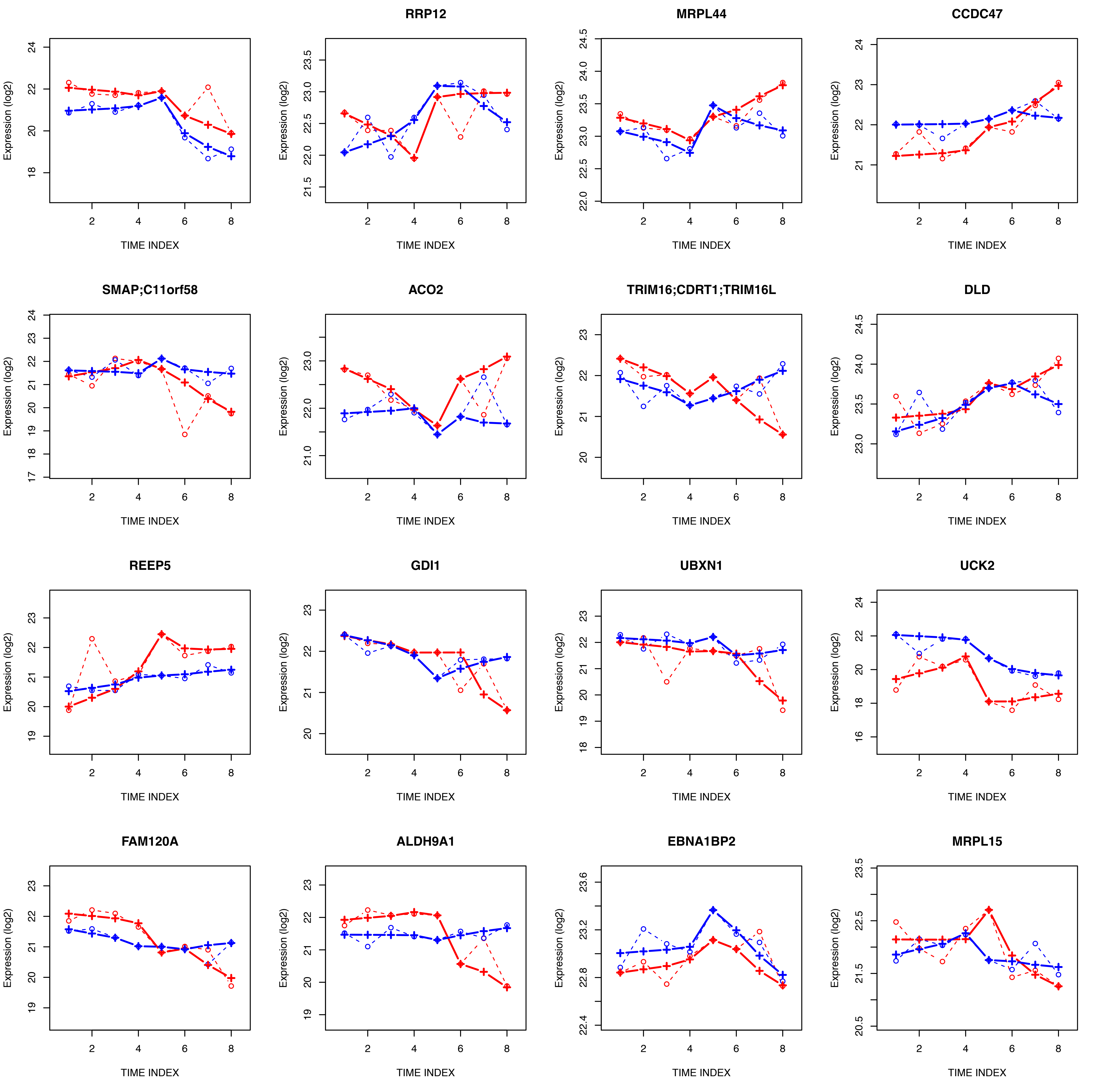
RRP12
MRPL44
CCDC47
SMAP;C11orf58
ACO2
TRIM16;CDRT1;TRIM16L
DLD
REEP5
GDI1
UBXN1
UCK2
FAM120A
ALDH9A1
EBNA1BP2
MRPL15
Expression (log2)
TIME INDEX

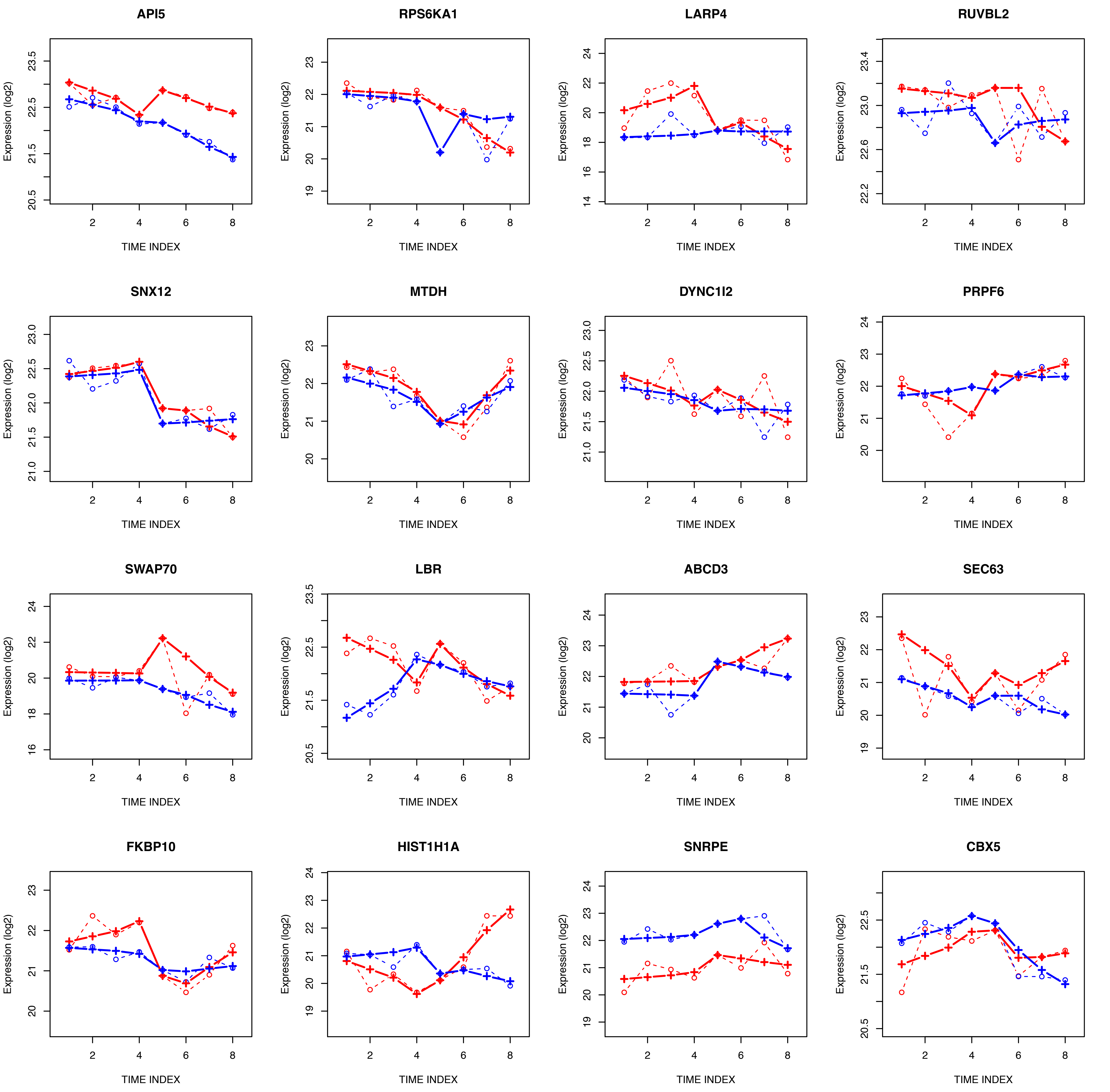
API5
RPS6KA1
LARP4
RUVBL2
SNX12
MTDH
DYNC1I2
PRPF6
SWAP70
LBR
ABCD3
SEC63
FKBP10
HIST1H1A
SNRPE
CBX5
Expression (log2)
TIME INDEX

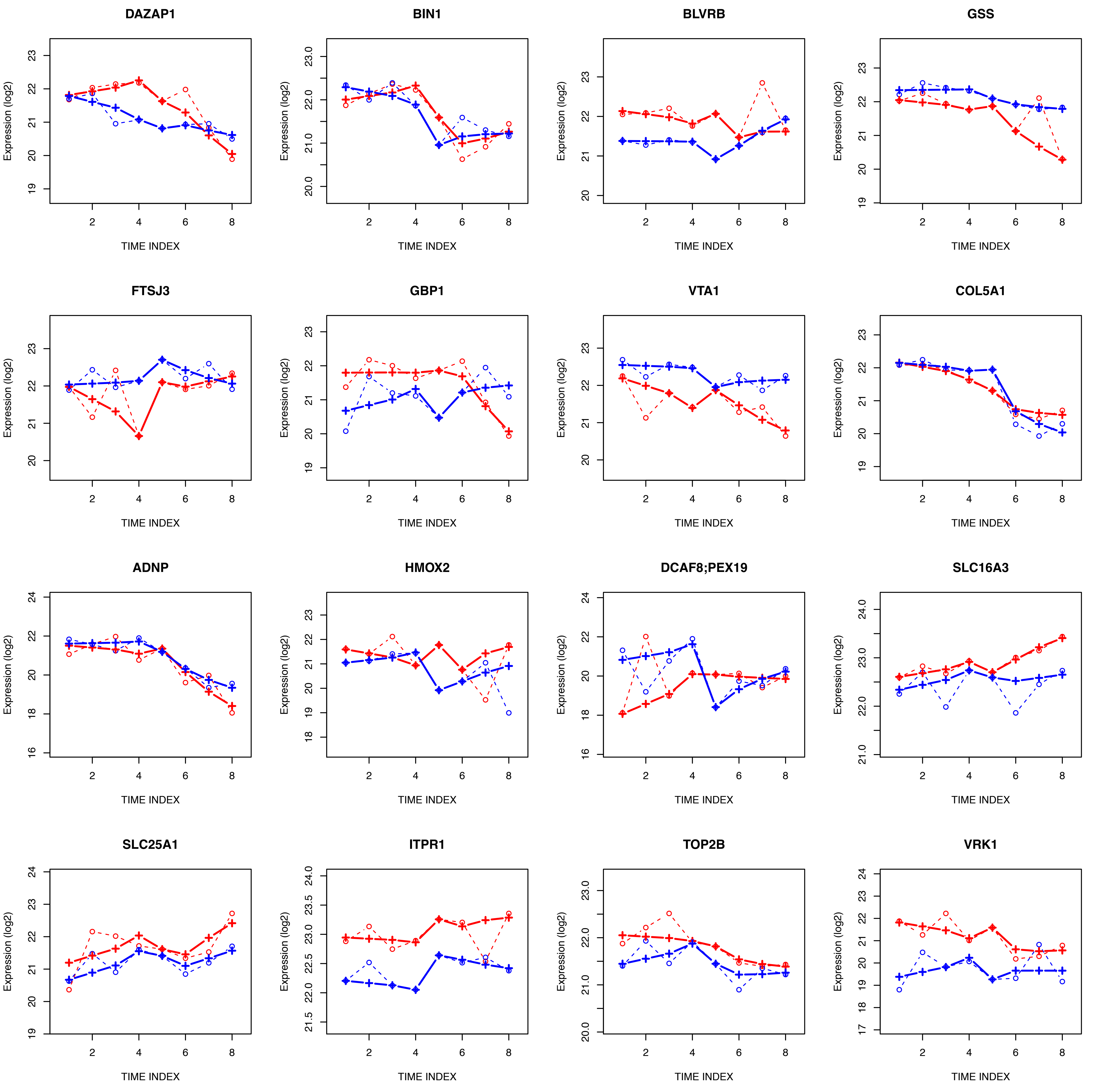

DAZAP1
BIN1
BLVRB
GSS
FTSJ3
GBP1
VTA1
COL5A1
ADNP
HMOX2
DCAF8;PEX19
SLC16A3
SLC25A1
ITPR1
TOP2B
VRK1
Expression (log2)
TIME INDEX

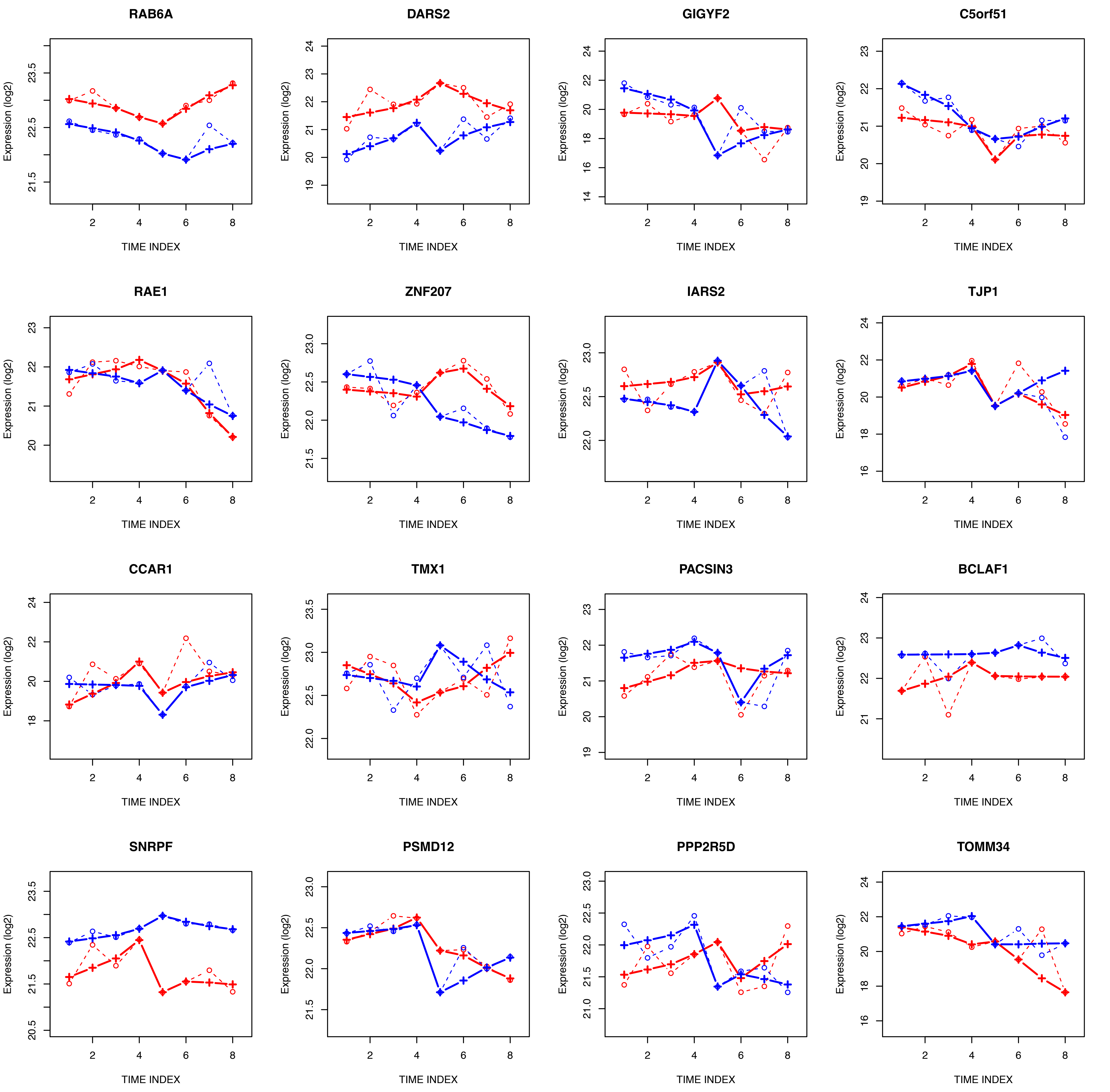
RAB6A
DARS2
GIGYF2
C5orf51
RAE1
ZNF207
IARS2
TJP1
CCAR1
TMX1
PACSIN3
BCLAF1
SNRPF
PSMD12
PPP2R5D
TOMM34
Expression (log2)
TIME INDEX

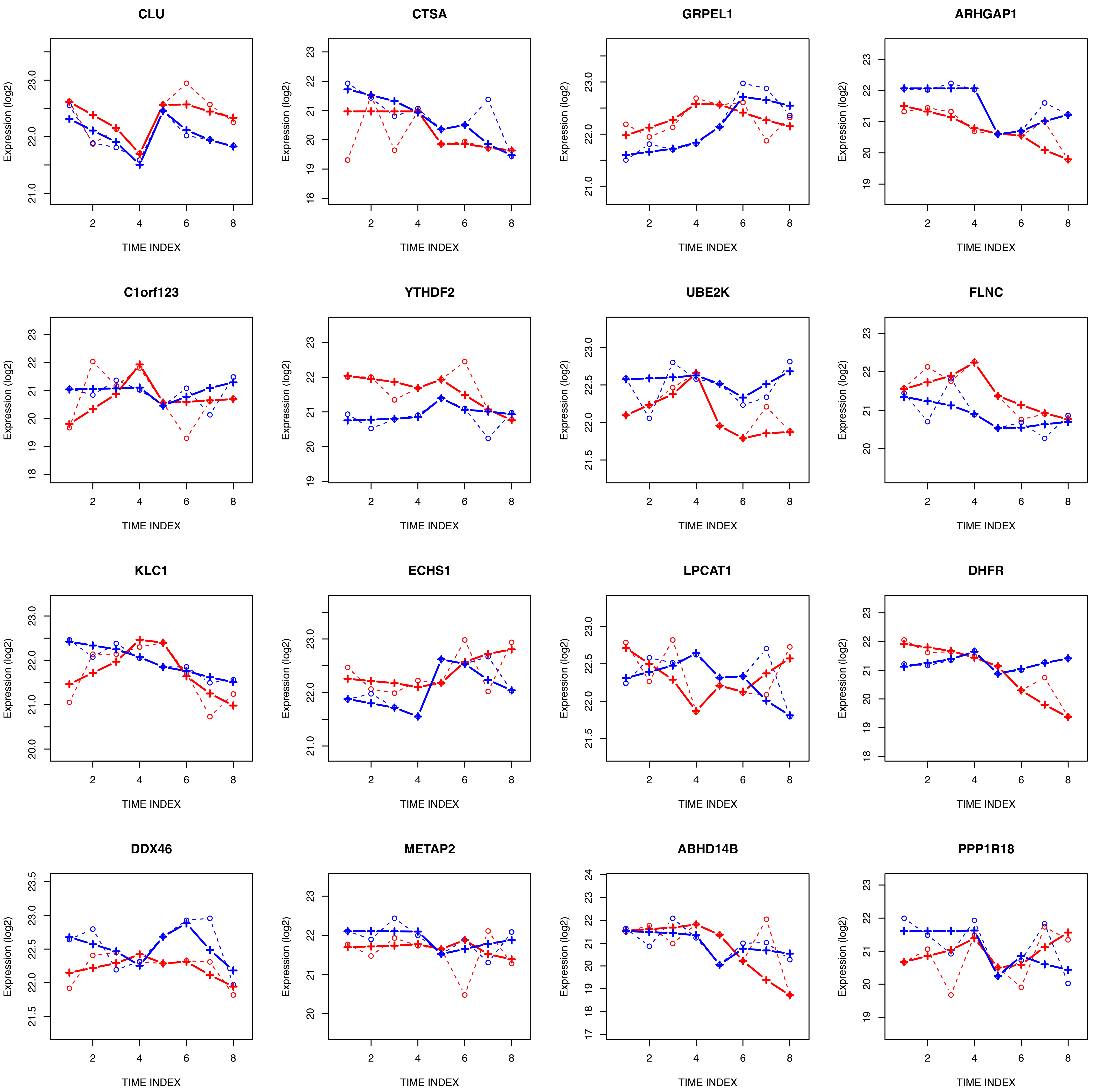
CLU
CTSA
GRPEL1
ARHGAP1
C1orf123
YTHDF2
UBE2K
FLNC
KLC1
ECHS1
LPCAT1
DHFR
DDX46
METAP2
ABHD14B
PPP1R18
Expression (log2)
TIME INDEX

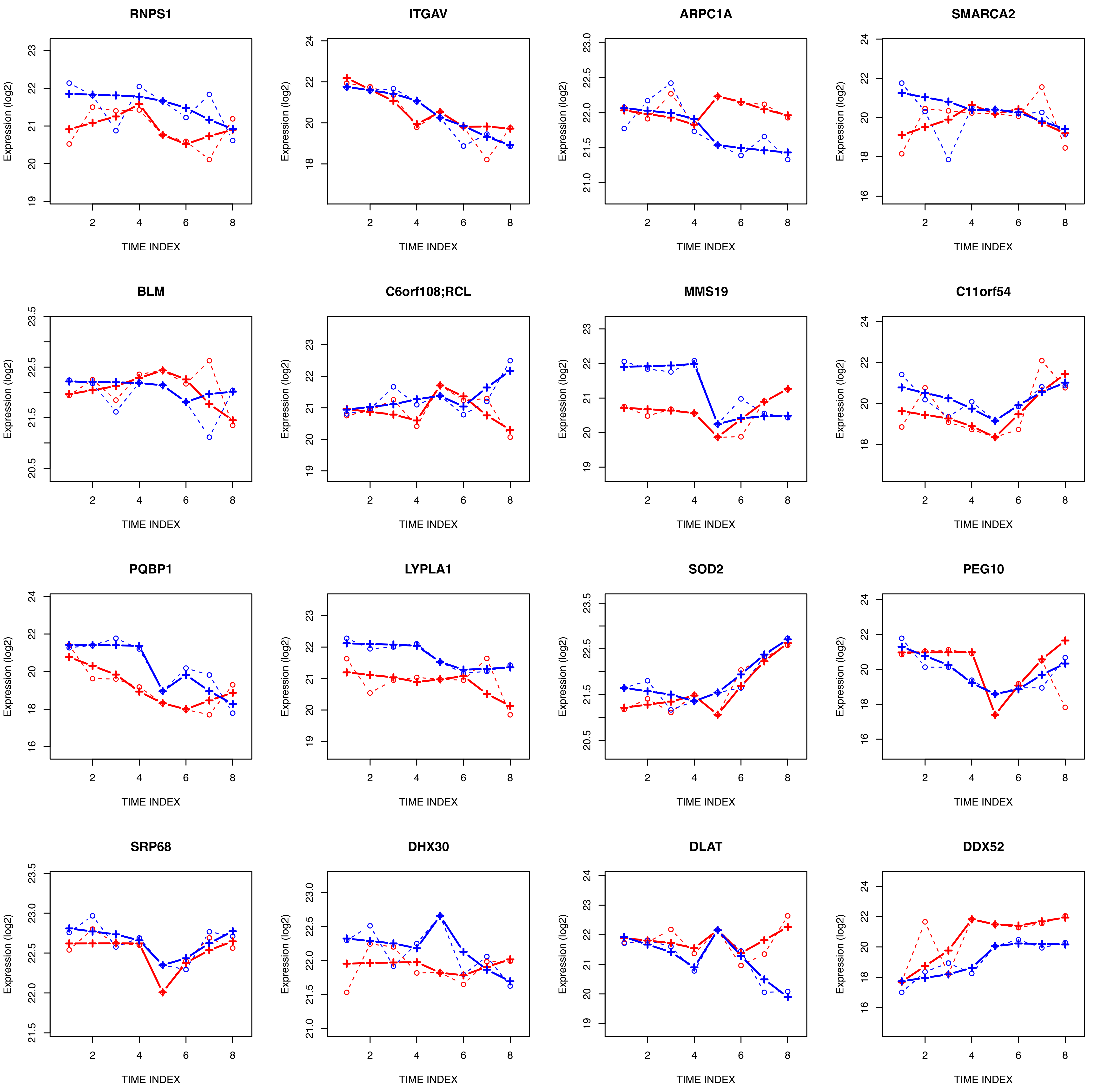
RNPS1
ITGAV
ARPC1A
SMARCA2
BLM
C6orf108;RCL
MMS19
C11orf54
PQBP1
LYPLA1
SOD2
PEG10
SRP68
DHX30
DLAT
DDX52
Expression (log2)
TIME INDEX

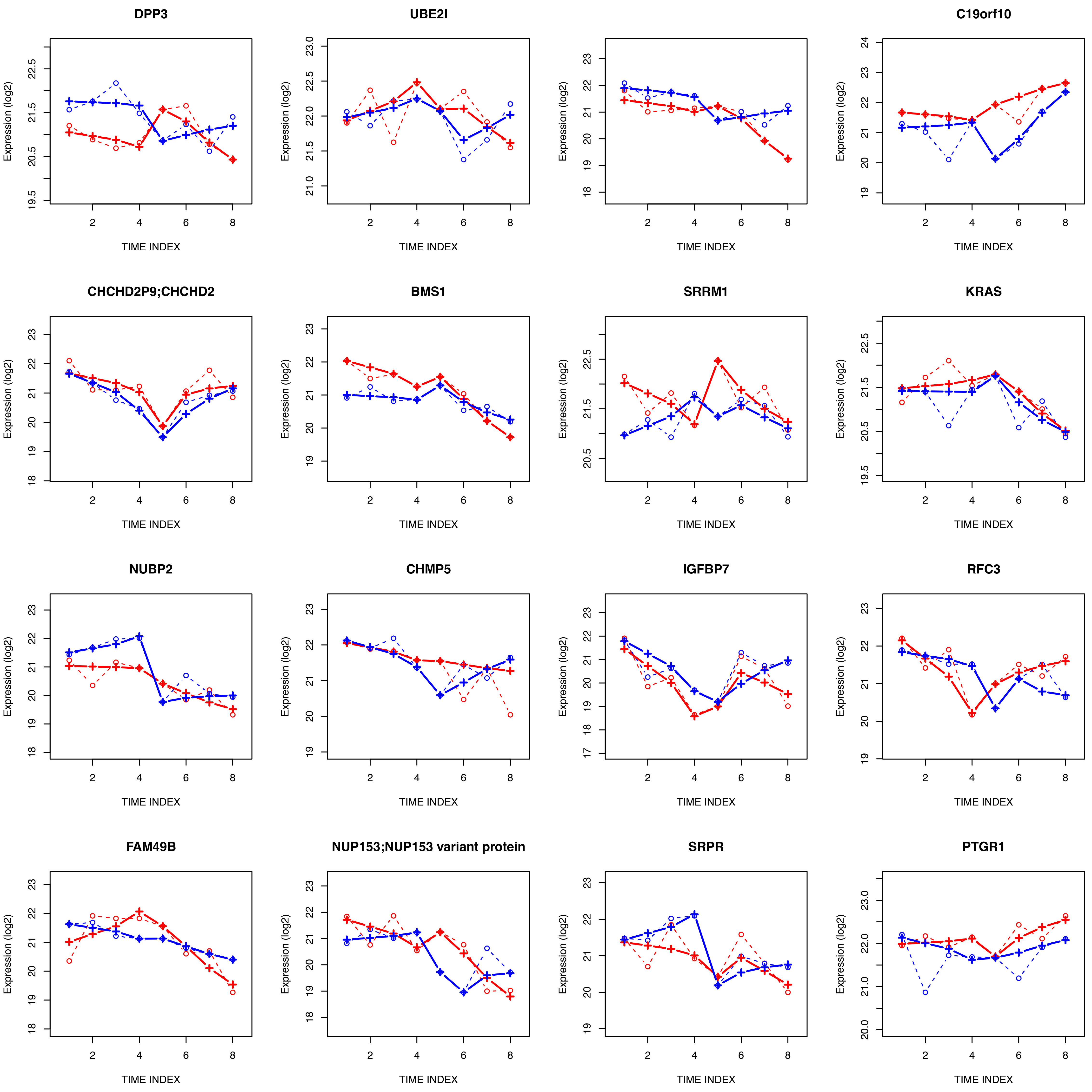
DPP3
UBE2I
C19orf10
CHCHD2P9;CHCHD2
BMS1
SRRM1
KRAS
NUBP2
CHMP5
IGFBP7
RFC3
FAM49B
NUP153;NUP153 variant protein
SRPR
PTGR1
Expression (log2)
TIME INDEX

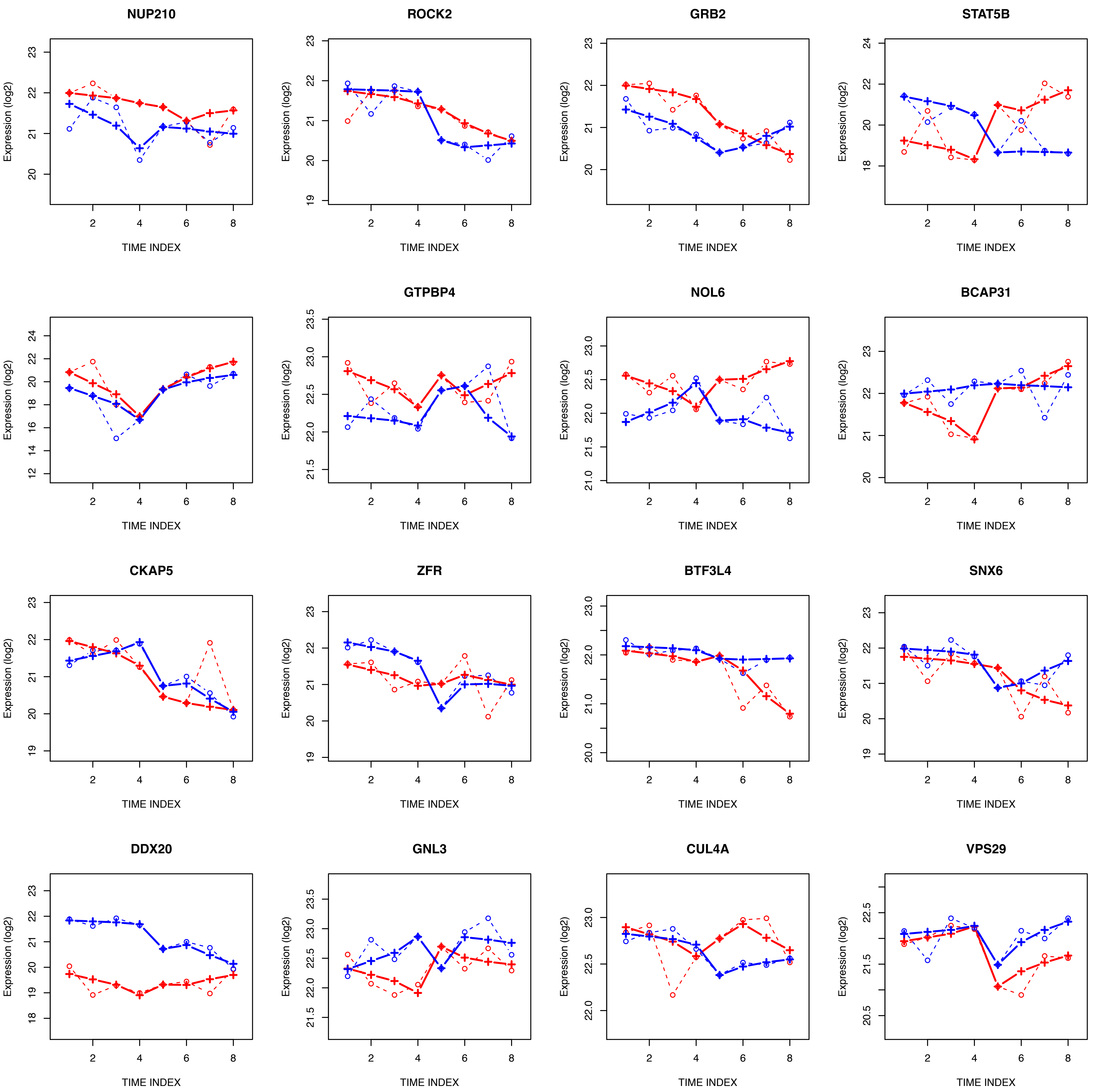
NUP210
ROCK2
GRB2
STAT5B
GTPBP4
NOL6
BCAP31
CKAP5
ZFR
BTF3L4
SNX6
DDX20
GNL3
CUL4A
VPS29
Expression (log2)
TIME INDEX

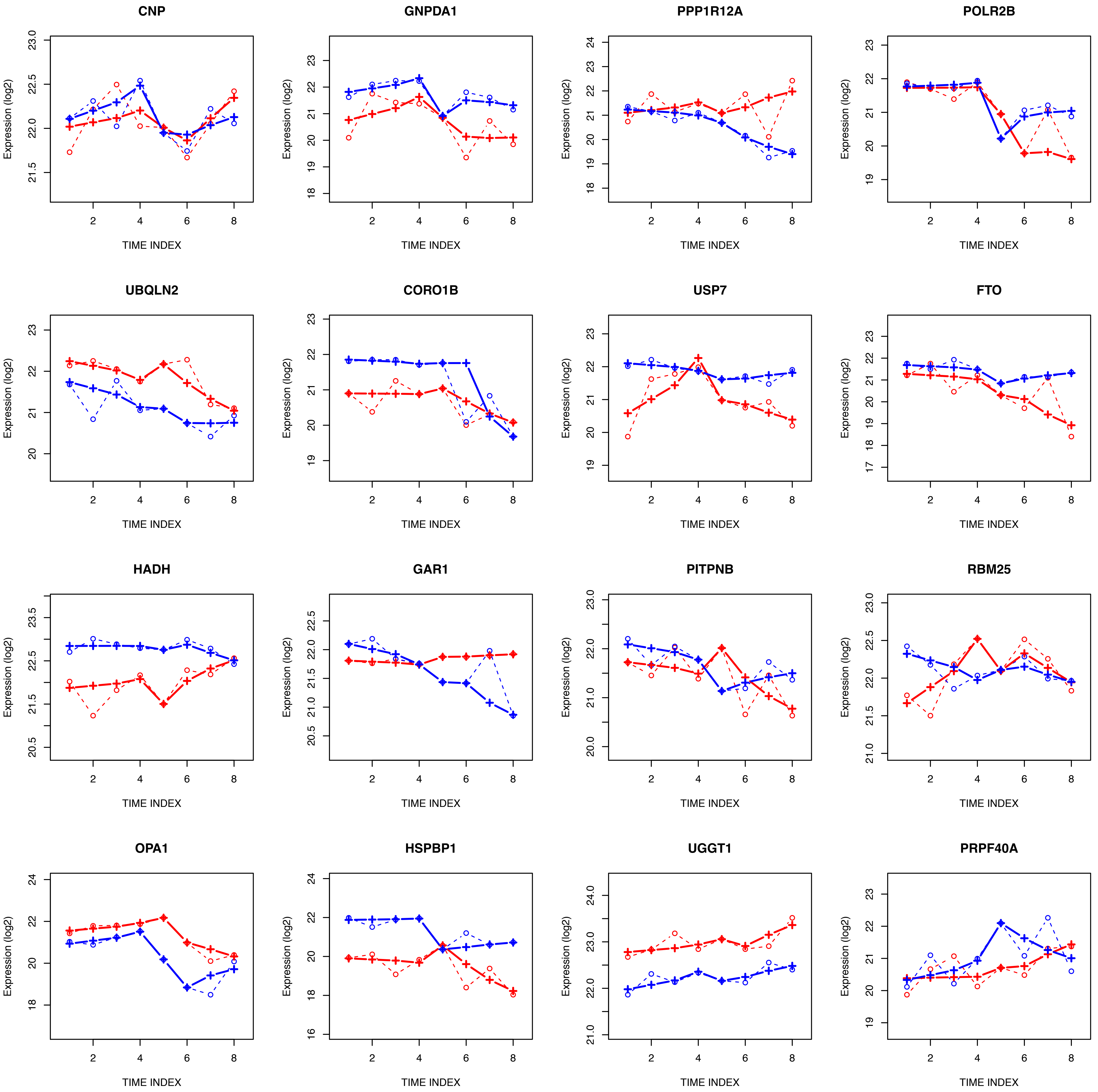
CNP
GNPDA1
PPP1R12A
POLR2B
UBQLN2
CORO1B
USP7
FTO
HADH
GAR1
PITPNB
RBM25
OPA1
HSPBP1
UGGT1
PRPF40A
Expression (log2)
TIME INDEX

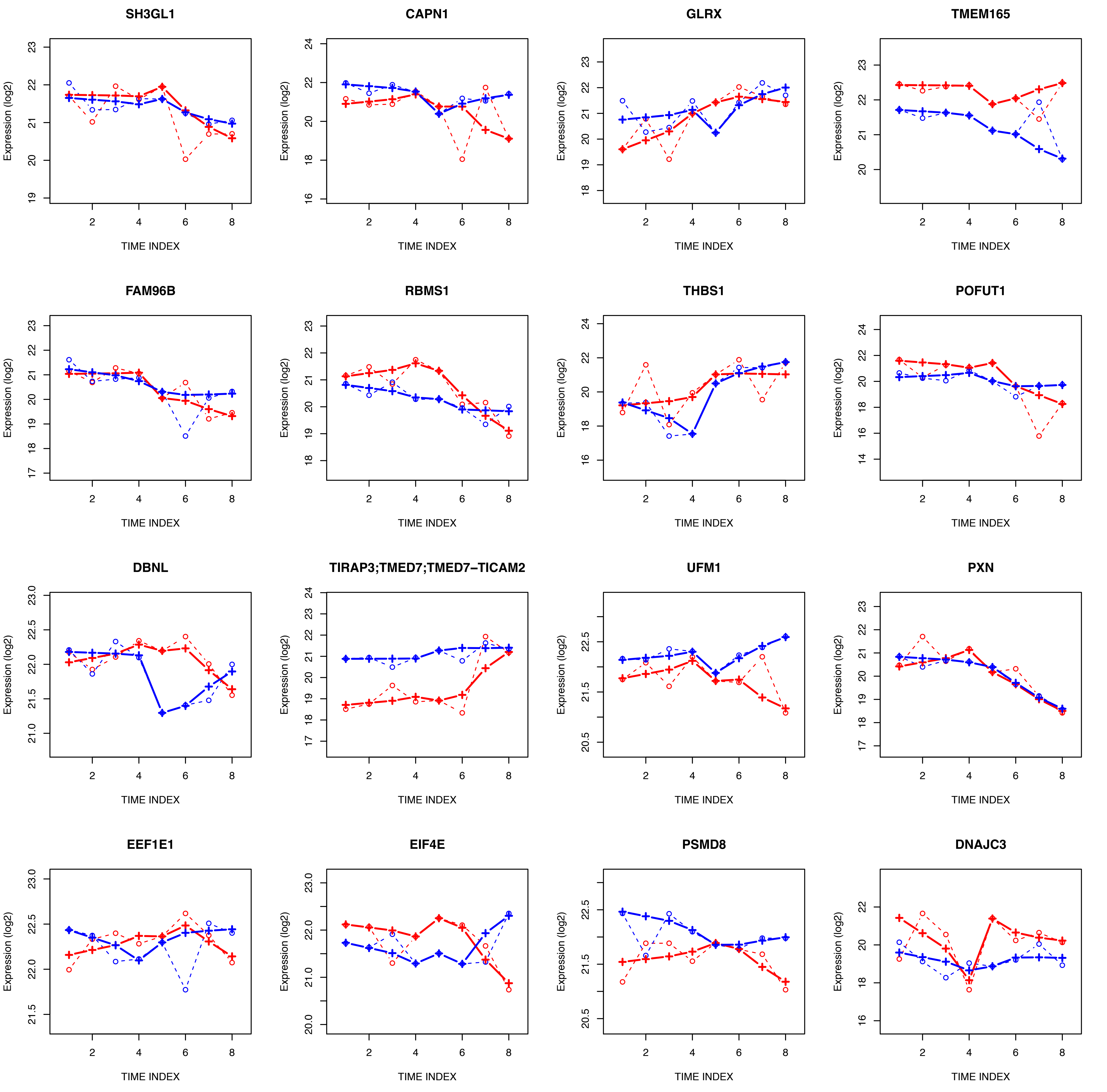
SH3GL1
Expression (log2)
19
20
21
22
23
TIME INDEX
2
4
6
8
CAPN1
Expression (log2)
16
18
20
22
24
TIME INDEX
GLRX
Expression (log2)
18
19
20
21
22
23
TIME INDEX
TMEM165
Expression (log2)
20
21
22
23
TIME INDEX
FAM96B
Expression (log2)
17
18
19
20
21
22
23
TIME INDEX
RBMS1
Expression (log2)
18
19
20
21
22
23
TIME INDEX
THBS1
Expression (log2)
16
18
20
22
24
TIME INDEX
POFUT1
Expression (log2)
14
16
18
20
22
24
TIME INDEX
DBNL
Expression (log2)
21.0
21.5
22.0
22.5
23.0
TIME INDEX
TIRAP3;TMED7;TMED7−TICAM2
Expression (log2)
17
18
19
20
21
22
23
24
TIME INDEX
UFM1
Expression (log2)
20.5
21.5
22.5
TIME INDEX
PXN
Expression (log2)
17
18
19
20
21
22
23
TIME INDEX
EEF1E1
Expression (log2)
21.5
22.0
22.5
23.0
TIME INDEX
EIF4E
Expression (log2)
20.0
21.0
22.0
23.0
TIME INDEX
PSMD8
Expression (log2)
20.5
21.5
22.5
TIME INDEX
DNAJC3
Expression (log2)
16
18
20
22
TIME INDEX

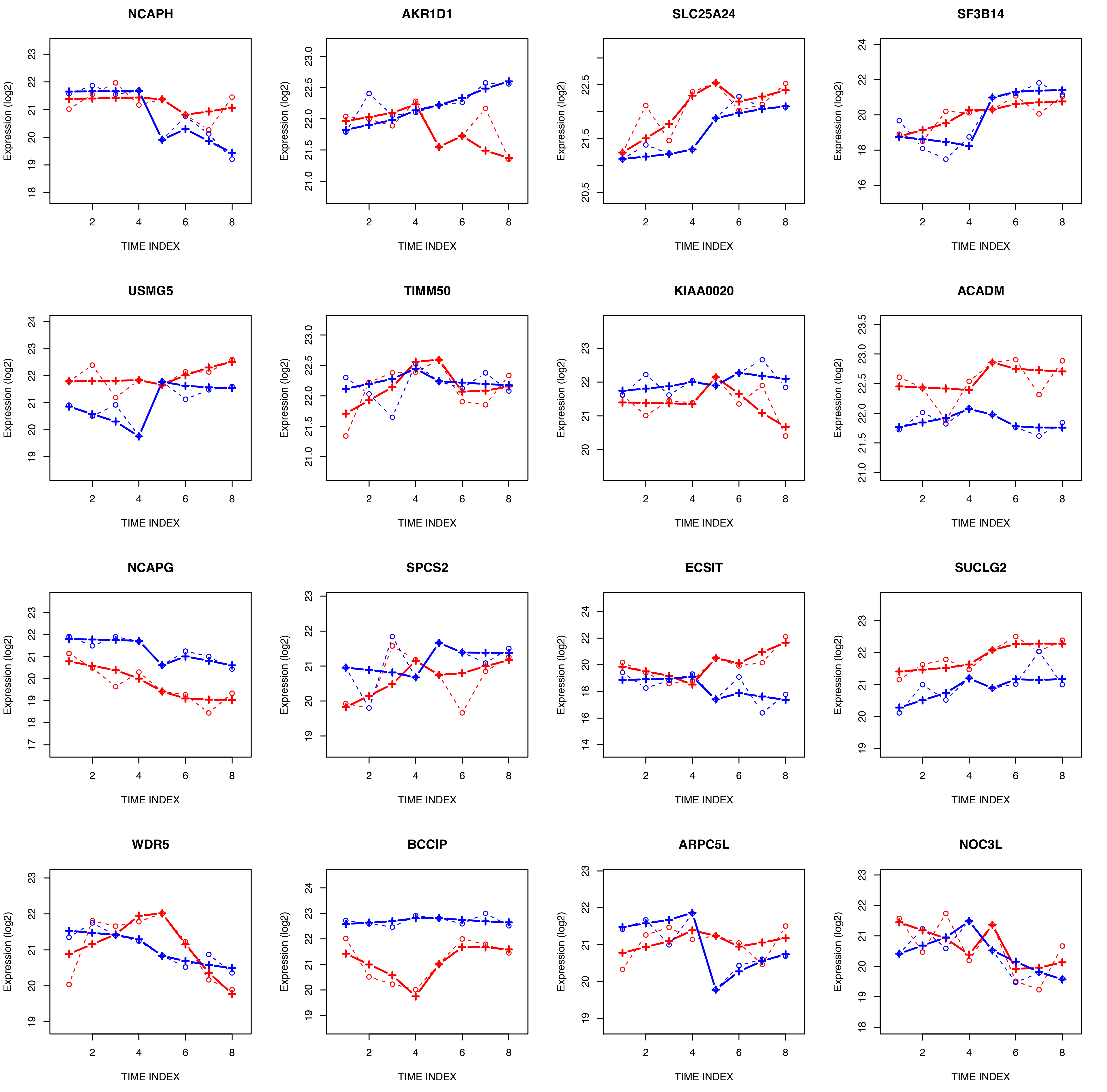
NCAPH
AKR1D1
SLC25A24
SF3B14
USMG5
TIMM50
KIAA0020
ACADM
NCAPG
SPCS2
ECSIT
SUCLG2
WDR5
BCCIP
ARPC5L
NOC3L
Expression (log2)
TIME INDEX

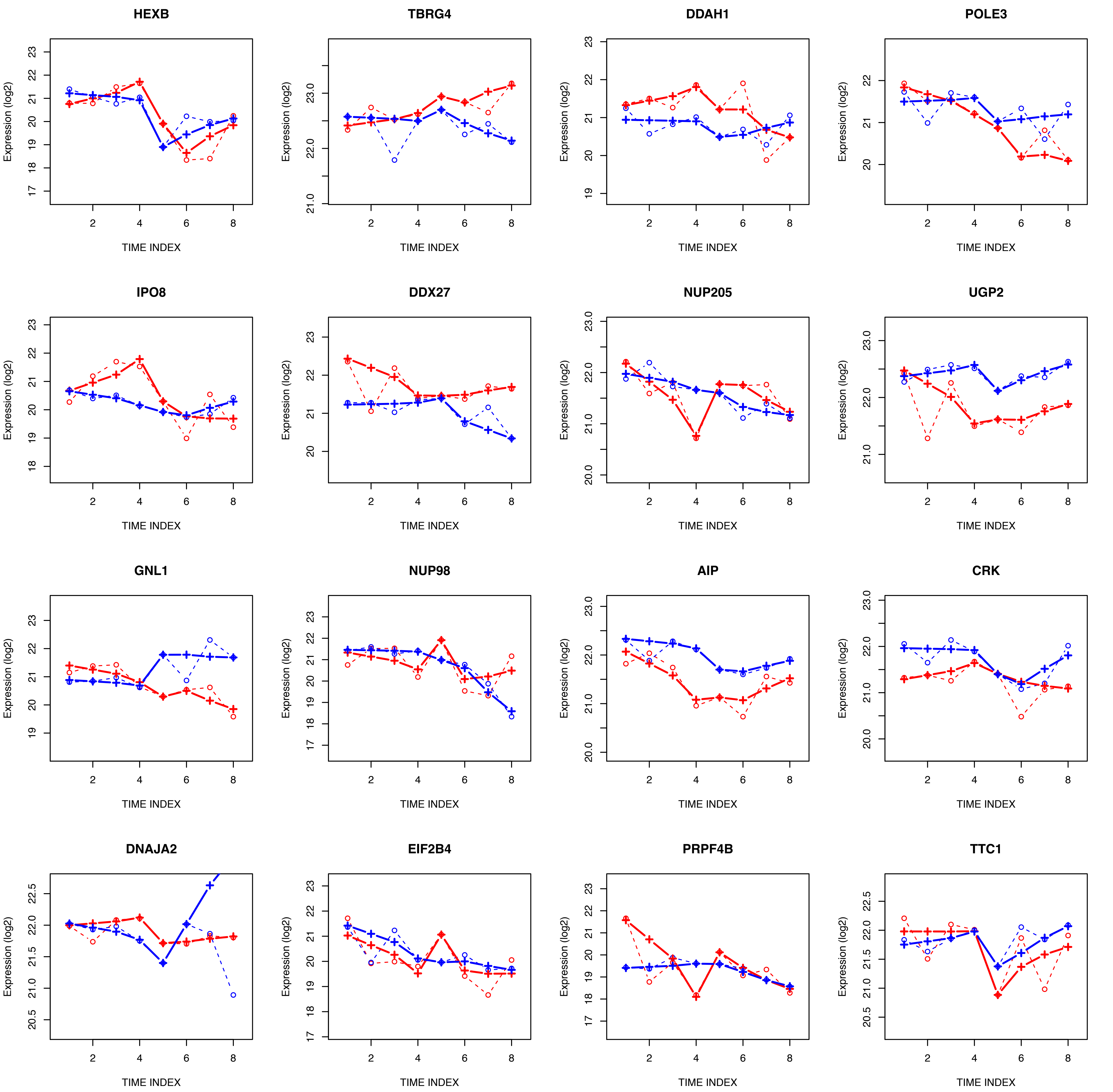
HEXB
TBRG4
DDAH1
POLE3
IPO8
DDX27
NUP205
UGP2
GNL1
NUP98
AIP
CRK
DNAJA2
EIF2B4
PRPF4B
TTC1
Expression (log2)
TIME INDEX

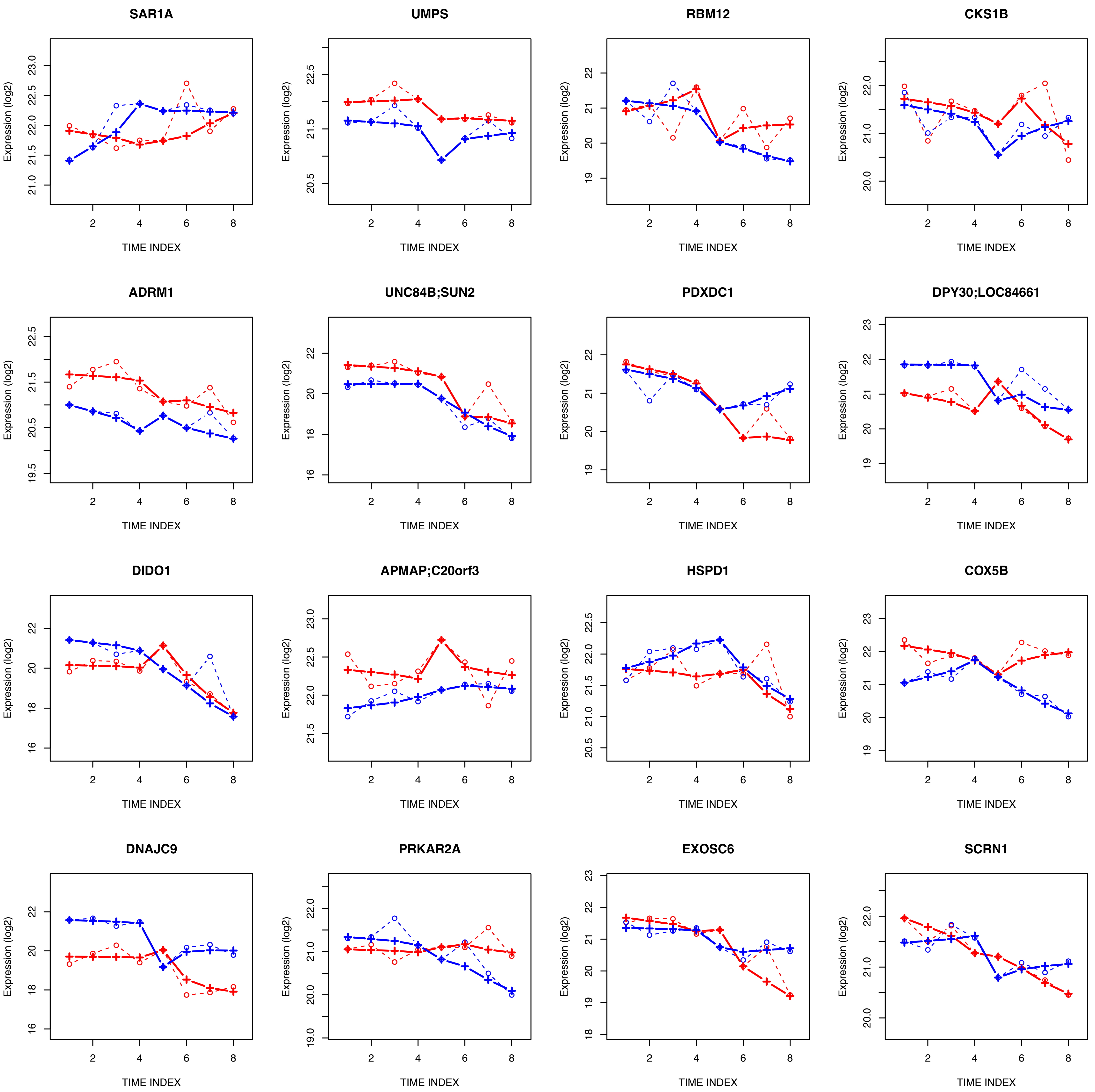
SAR1A
UMPS
RBM12
CKS1B
ADRM1
UNC84B;SUN2
PDXDC1
DPY30;LOC84661
DIDO1
APMAP;C20orf3
HSPD1
COX5B
DNAJC9
PRKAR2A
EXOSC6
SCRN1
Expression (log2)
TIME INDEX

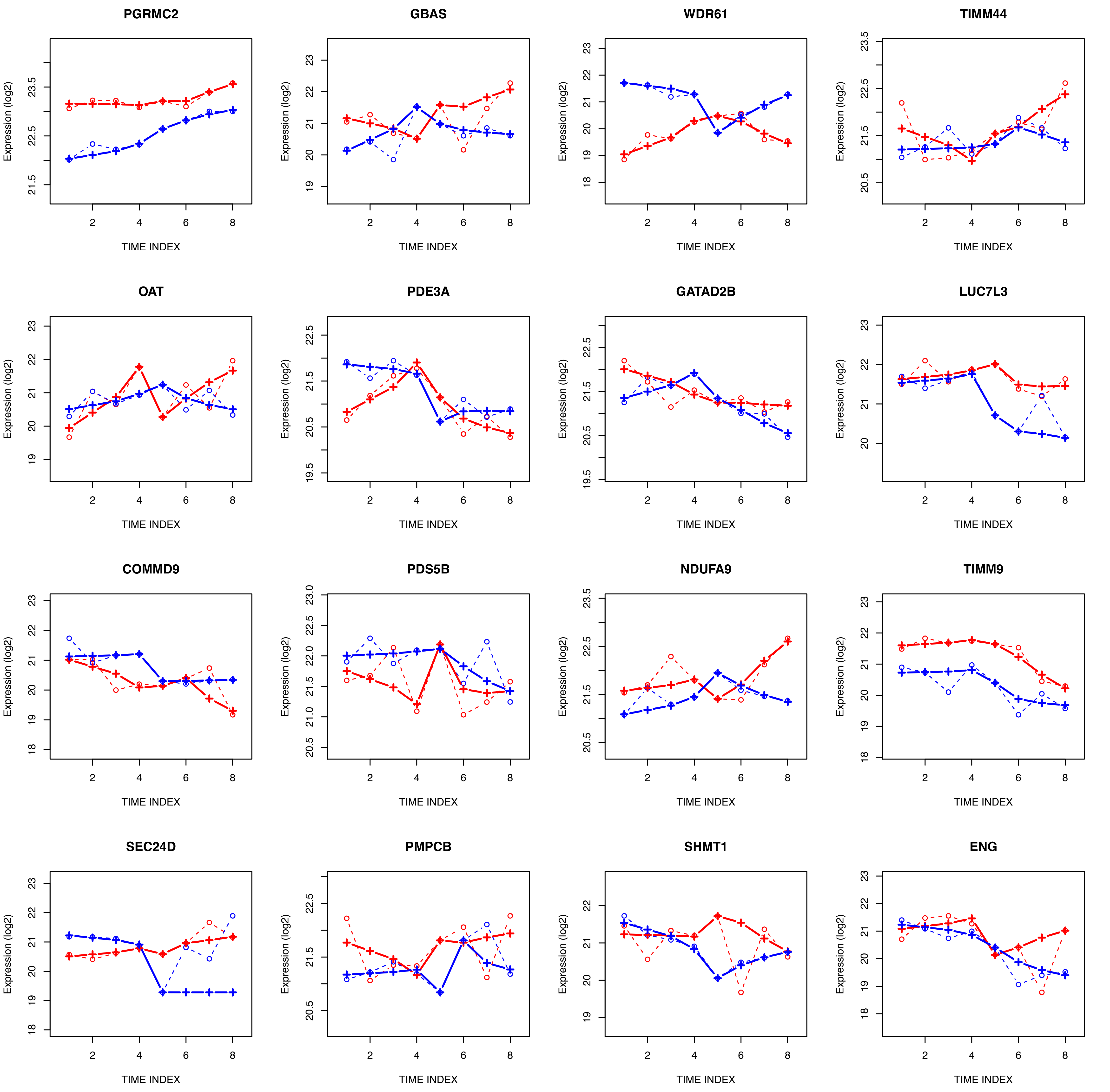
PGRMC2
GBAS
WDR61
TIMM44
OAT
PDE3A
GATAD2B
LUC7L3
COMMD9
PDS5B
NDUFA9
TIMM9
SEC24D
PMPCB
SHMT1
ENG
Expression (log2)
TIME INDEX

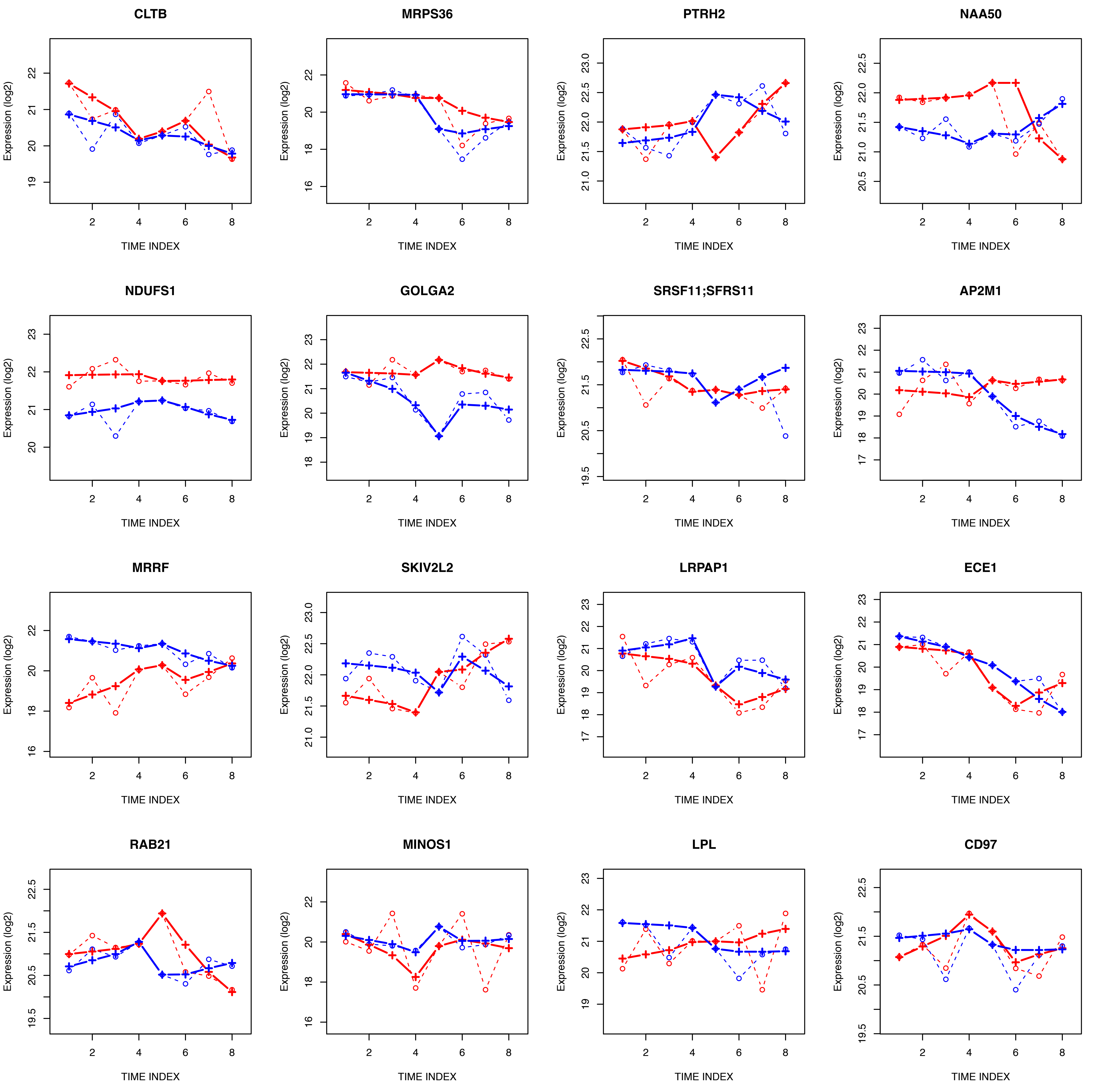

CLTB
MRPS36
PTRH2
NAA50
NDUFS1
GOLGA2
SRSF11;SFRS11
AP2M1
MRRF
SKIV2L2
LRPAP1
ECE1
RAB21
MINOS1
LPL
CD97
Expression (log2)
TIME INDEX

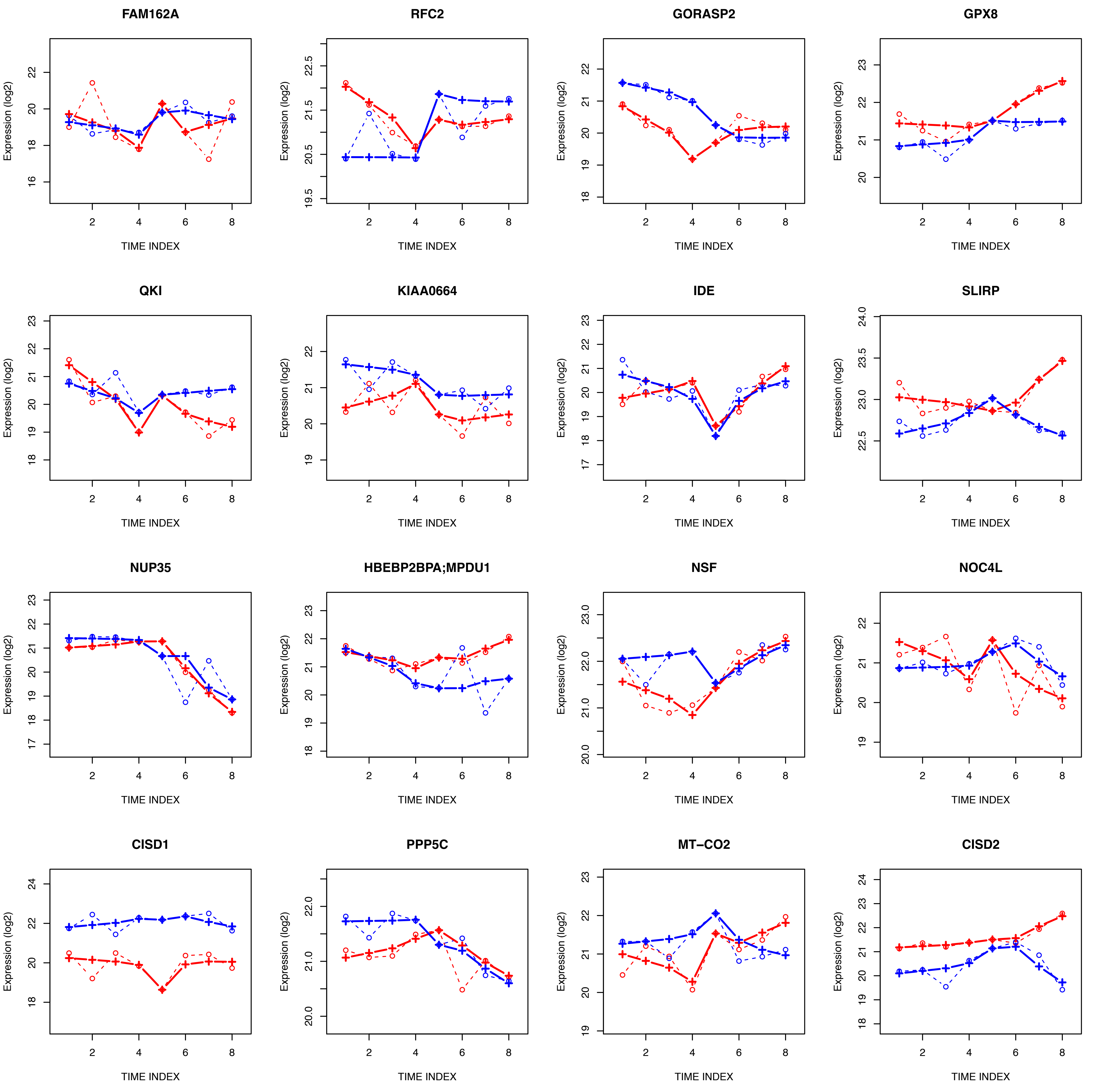
FAM162A
RFC2
GORASP2
GPX8
QKI
KIAA0664
IDE
SLIRP
NUP35
HBEBP2BPA;MPDU1
NSF
NOC4L
CISD1
PPP5C
MT−CO2
CISD2
Expression (log2)
TIME INDEX

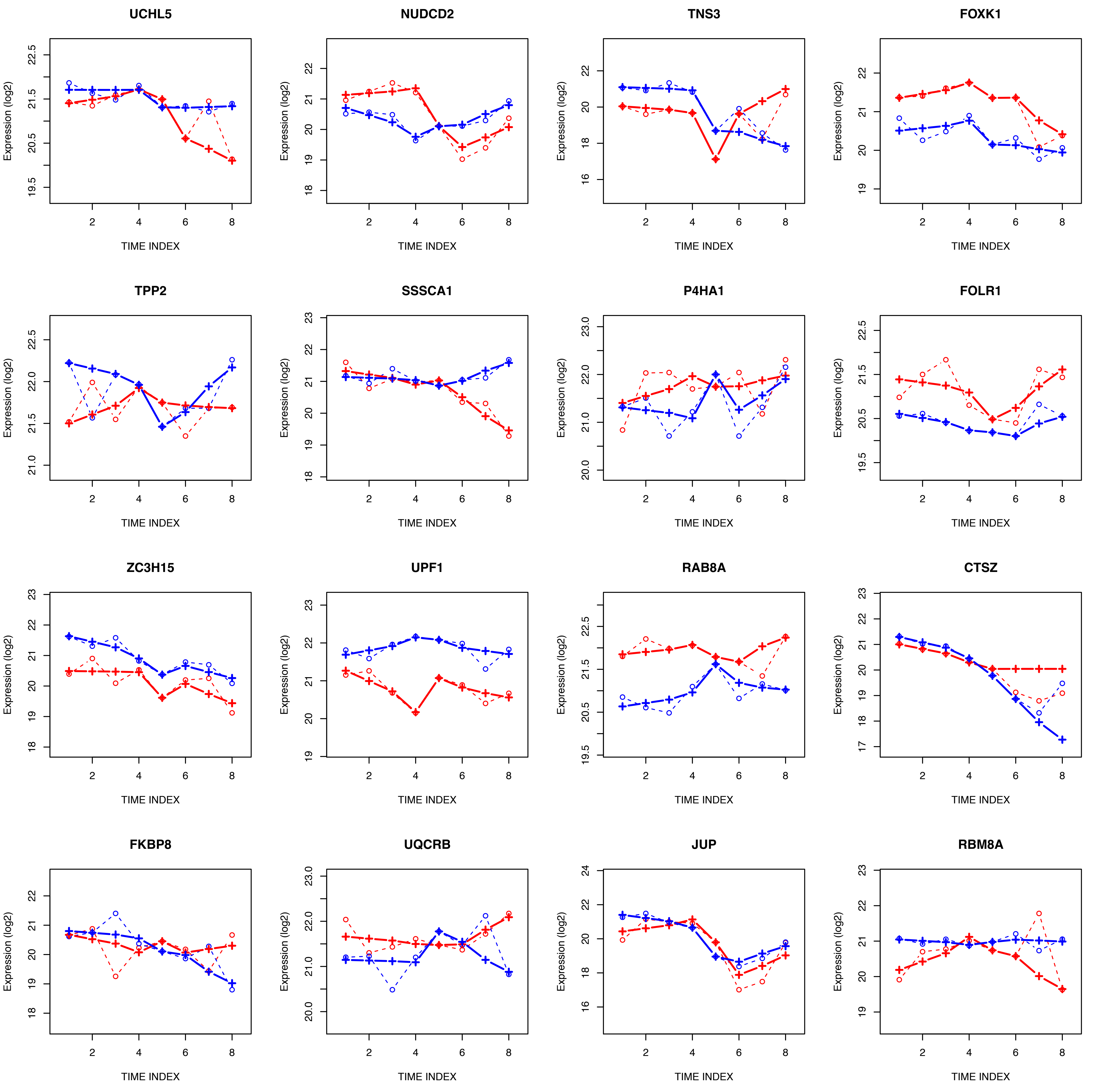
UCHL5
NUDCD2
TNS3
FOXK1
TPP2
SSSCA1
P4HA1
FOLR1
ZC3H15
UPF1
RAB8A
CTSZ
FKBP8
UQCRB
JUP
RBM8A
Expression (log2)
TIME INDEX

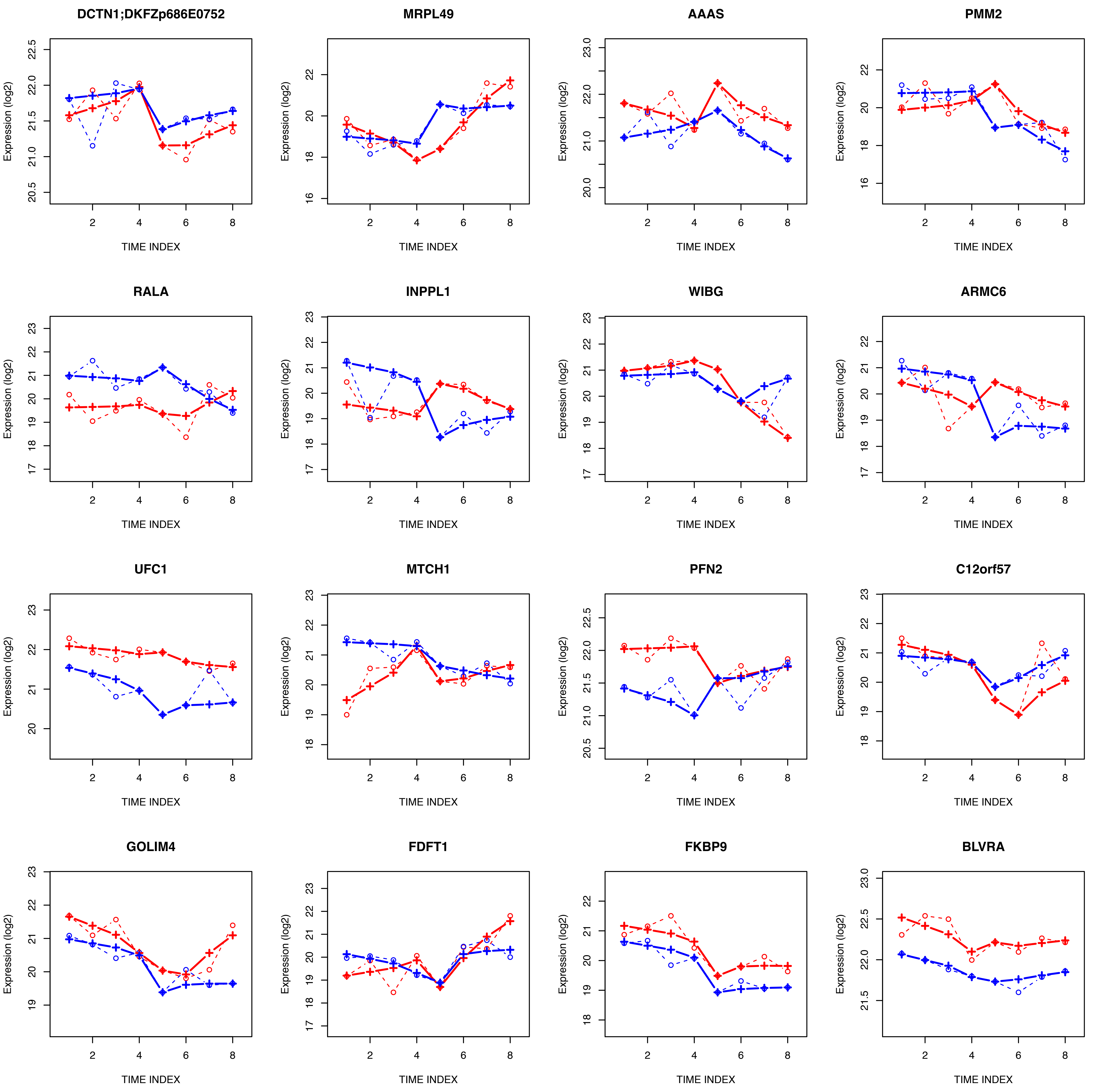
DCTN1;DKFZp686E0752
MRPL49
AAAS
PMM2
RALA
INPPL1
WIBG
ARMC6
UFC1
MTCH1
PFN2
C12orf57
GOLIM4
FDFT1
FKBP9
BLVRA
Expression (log2)
TIME INDEX

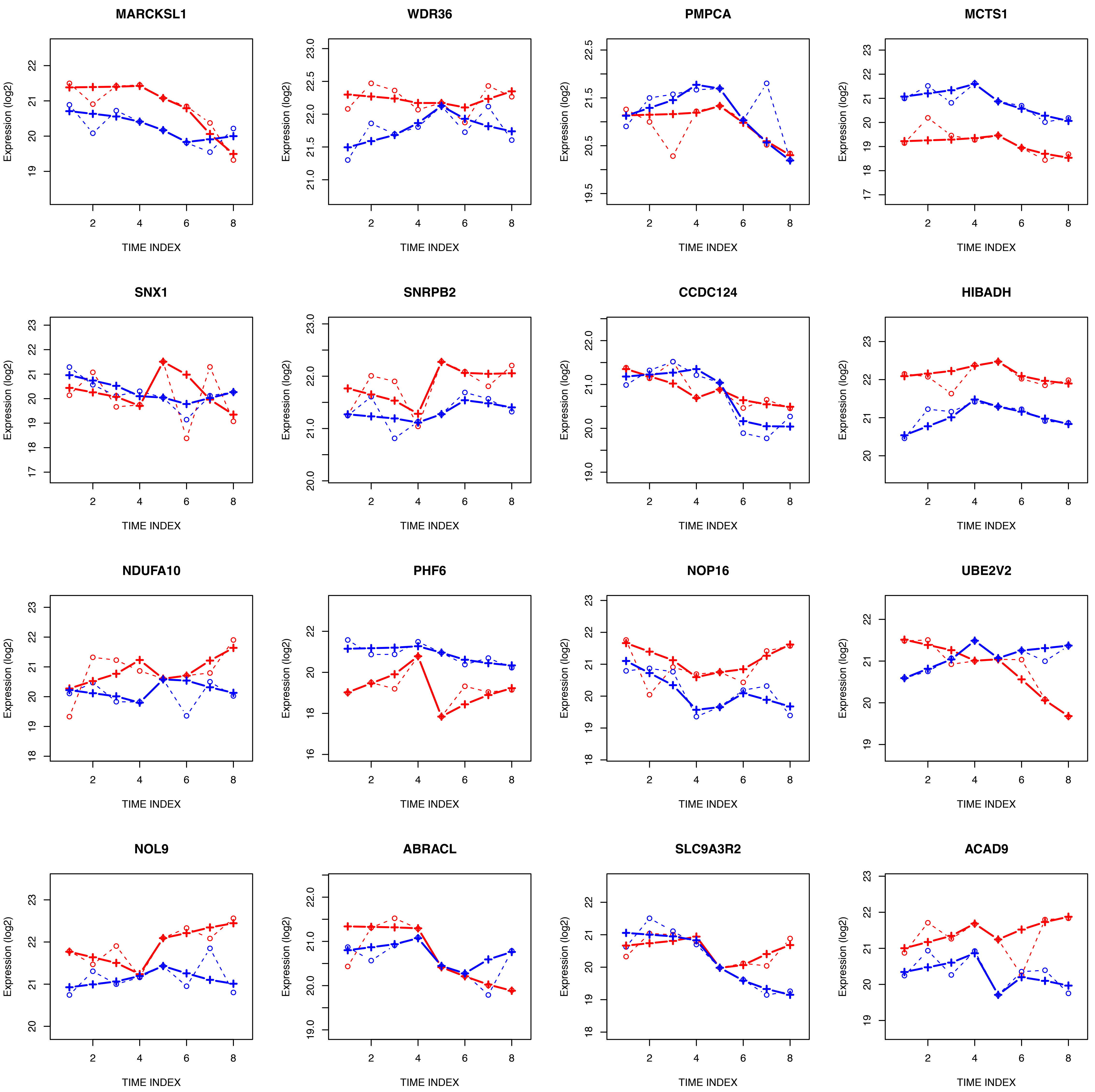
MARCKSL1
WDR36
PMPCA
MCTS1
SNX1
SNRPB2
CCDC124
HIBADH
NDUFA10
PHF6
NOP16
UBE2V2
NOL9
ABRACL
SLC9A3R2
ACAD9
Expression (log2)
TIME INDEX

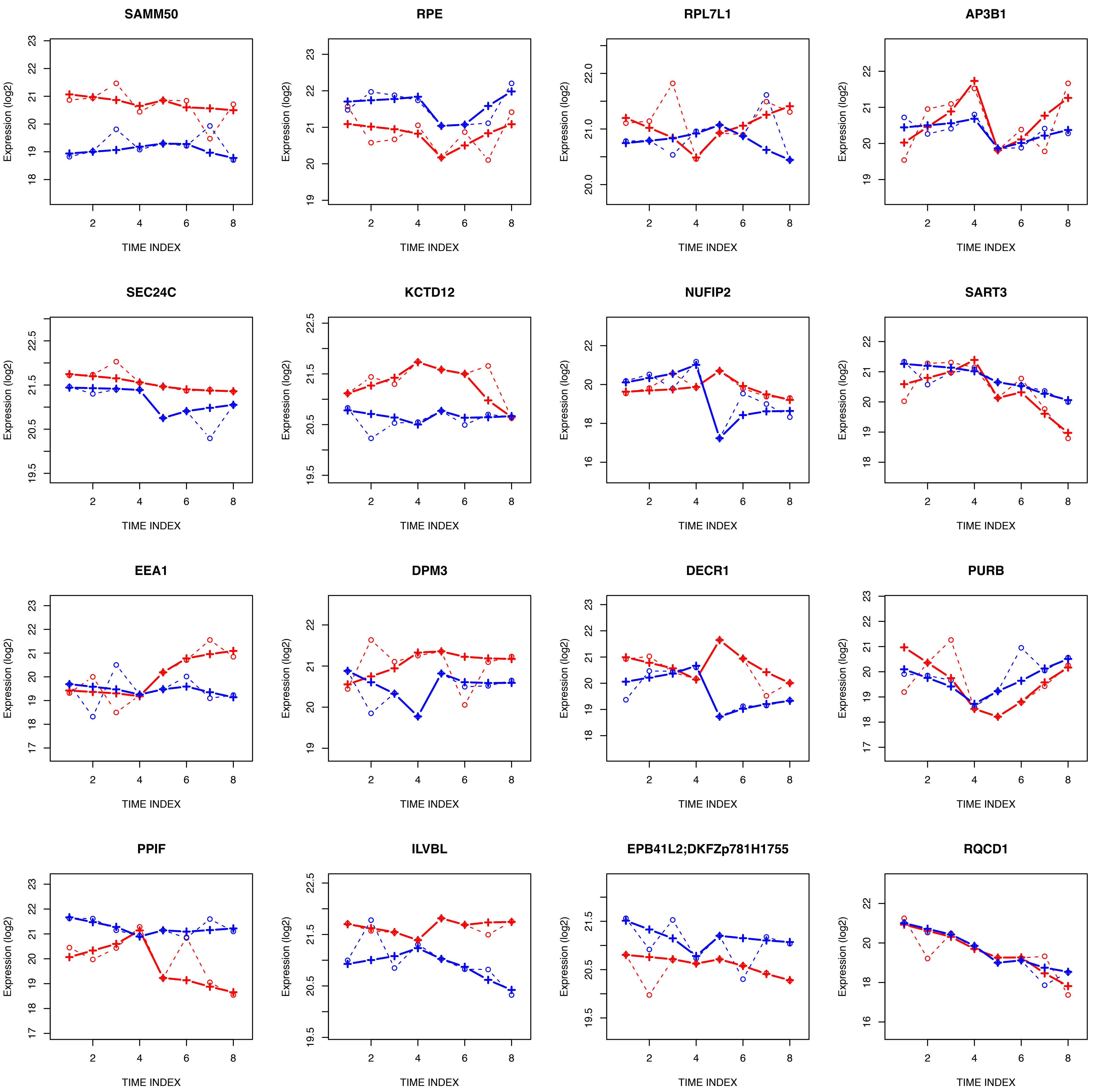
SAMM50
RPE
RPL7L1
AP3B1
SEC24C
KCTD12
NUFIP2
SART3
EEA1
DPM3
DECR1
PURB
PPIF
ILVBL
EPB41L2;DKFZp781H1755
RQCD1
Expression (log2)
TIME INDEX

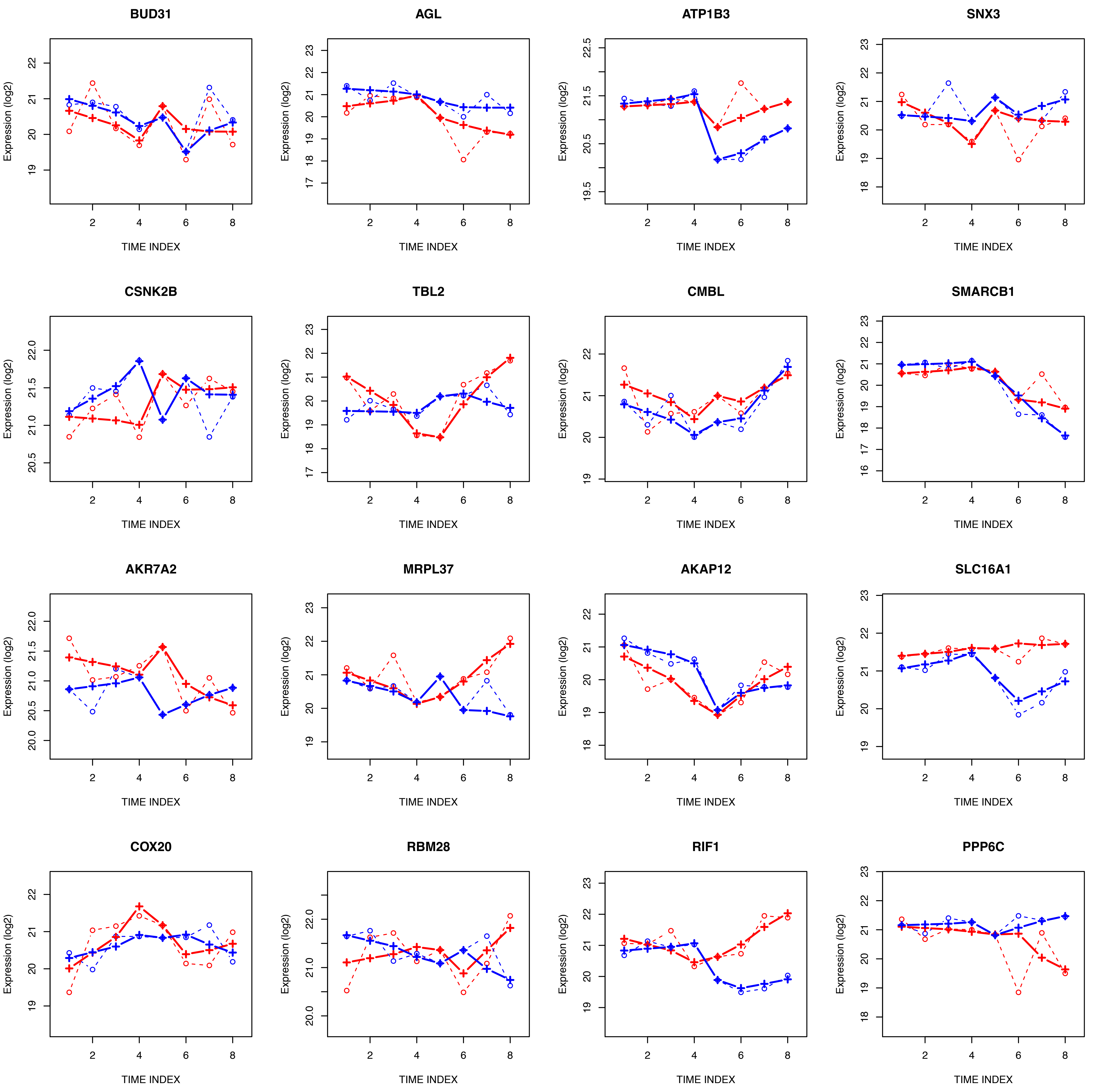
BUD31
AGL
ATP1B3
SNX3
CSNK2B
TBL2
CMBL
SMARCB1
AKR7A2
MRPL37
AKAP12
SLC16A1
COX20
RBM28
RIF1
PPP6C
Expression (log2)
TIME INDEX

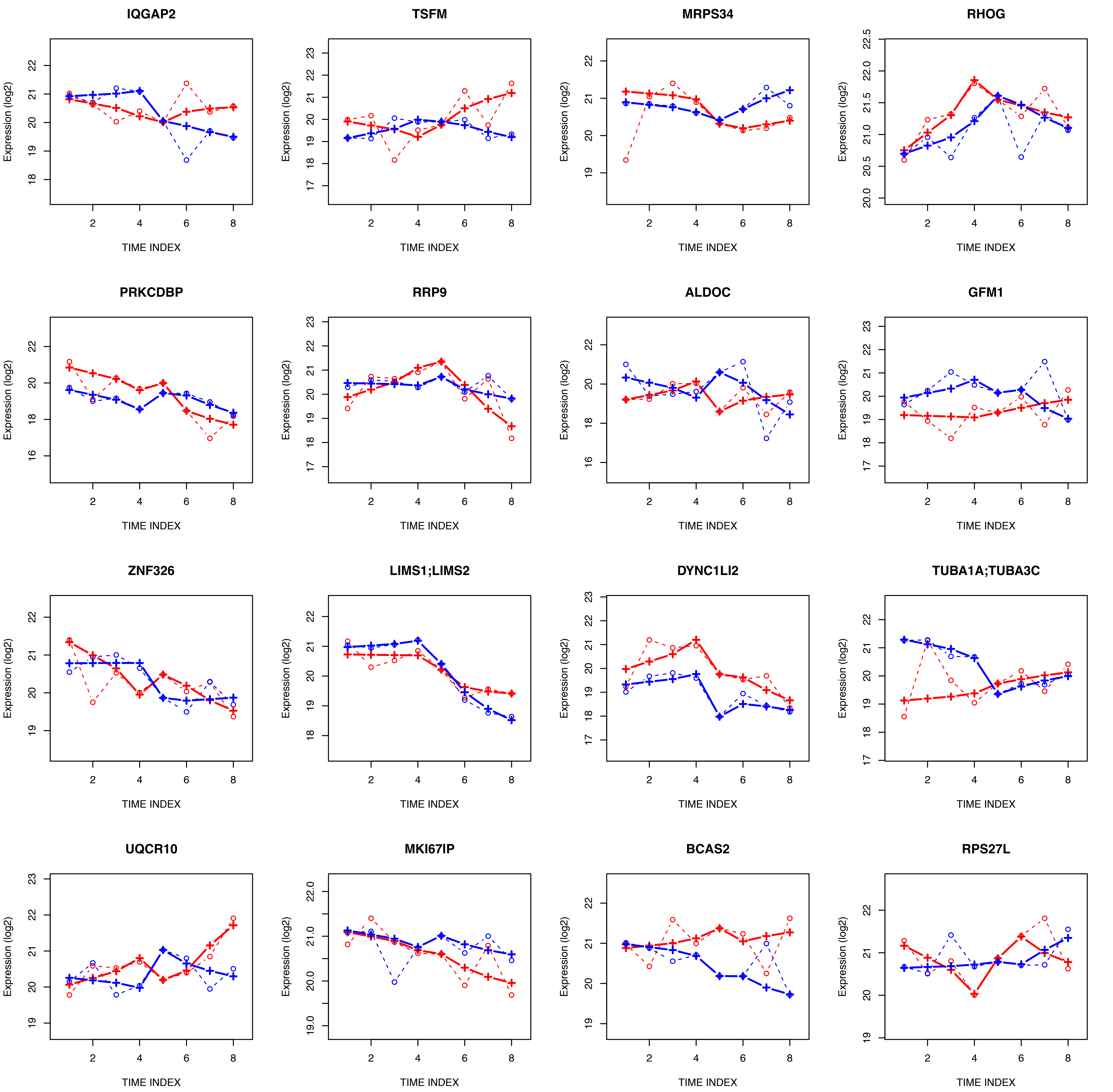
IQGAP2
TSFM
MRPS34
RHOG
PRKCDBP
RRP9
ALDOC
GFM1
ZNF326
LIMS1;LIMS2
DYNC1LI2
TUBA1A;TUBA3C
UQCR10
MKI67IP
BCAS2
RPS27L
Expression (log2)
TIME INDEX

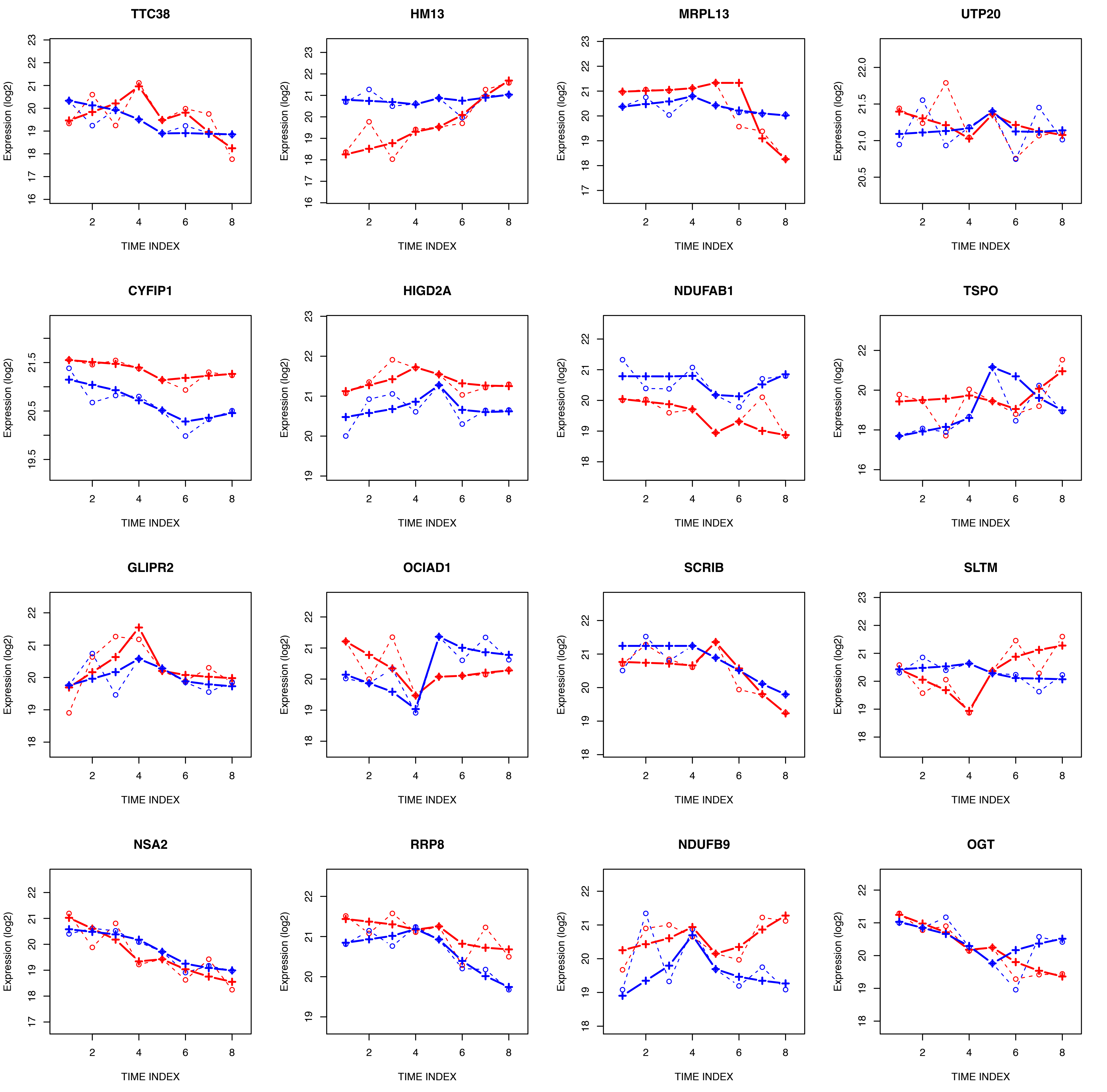
TTC38
Expression (log2)
TIME INDEX
HM13
Expression (log2)
TIME INDEX
MRPL13
Expression (log2)
TIME INDEX
UTP20
Expression (log2)
TIME INDEX
CYFIP1
Expression (log2)
TIME INDEX
HIGD2A
Expression (log2)
TIME INDEX
NDUFAB1
Expression (log2)
TIME INDEX
TSPO
Expression (log2)
TIME INDEX
GLIPR2
Expression (log2)
TIME INDEX
OCIAD1
Expression (log2)
TIME INDEX
SCRIB
Expression (log2)
TIME INDEX
SLTM
Expression (log2)
TIME INDEX
NSA2
Expression (log2)
TIME INDEX
RRP8
Expression (log2)
TIME INDEX
NDUFB9
Expression (log2)
TIME INDEX
OGT
Expression (log2)
TIME INDEX

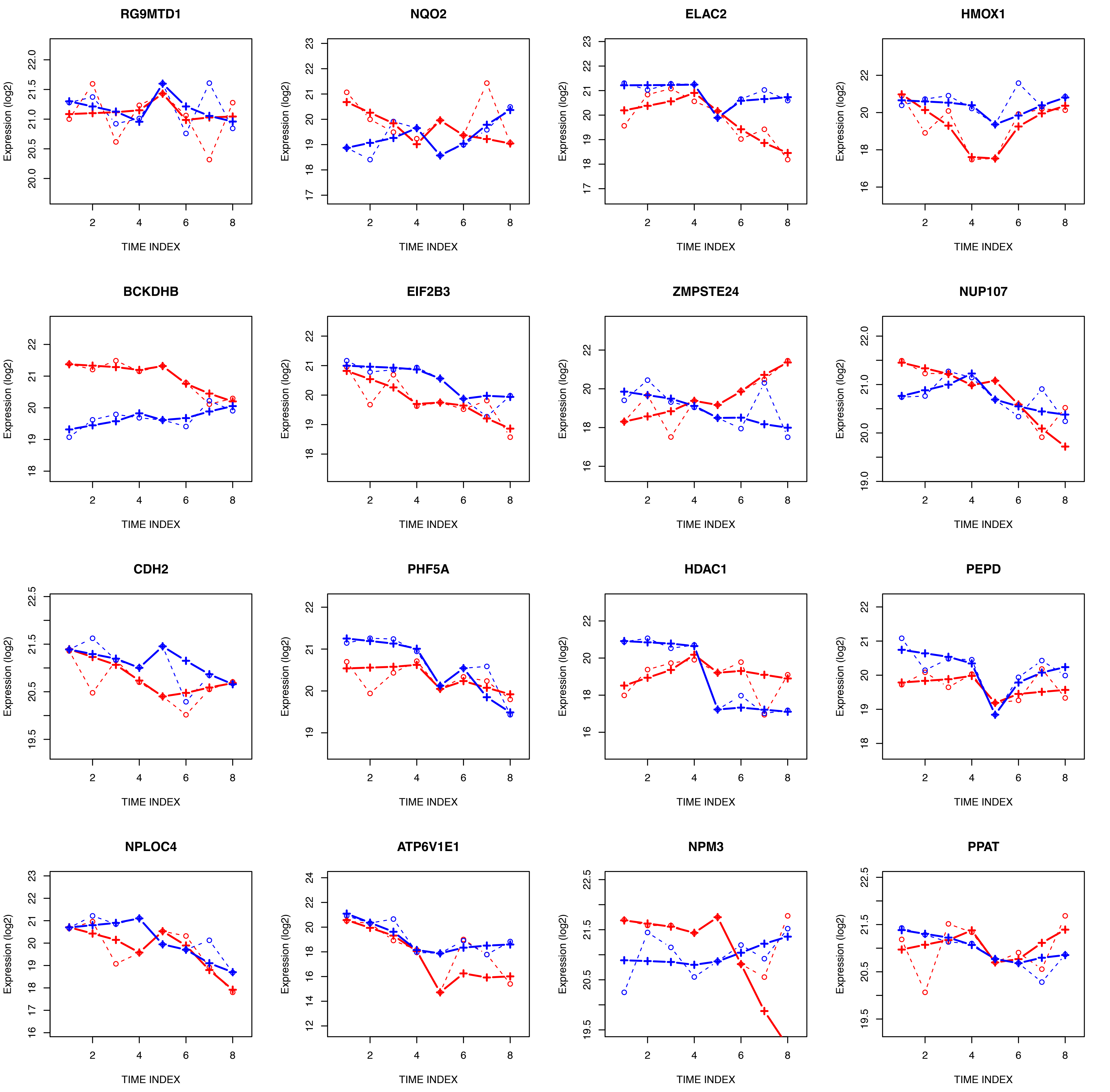
RG9MTD1
NQO2
ELAC2
HMOX1
BCKDHB
EIF2B3
ZMPSTE24
NUP107
CDH2
PHF5A
HDAC1
PEPD
NPLOC4
ATP6V1E1
NPM3
PPAT
Expression (log2)
TIME INDEX

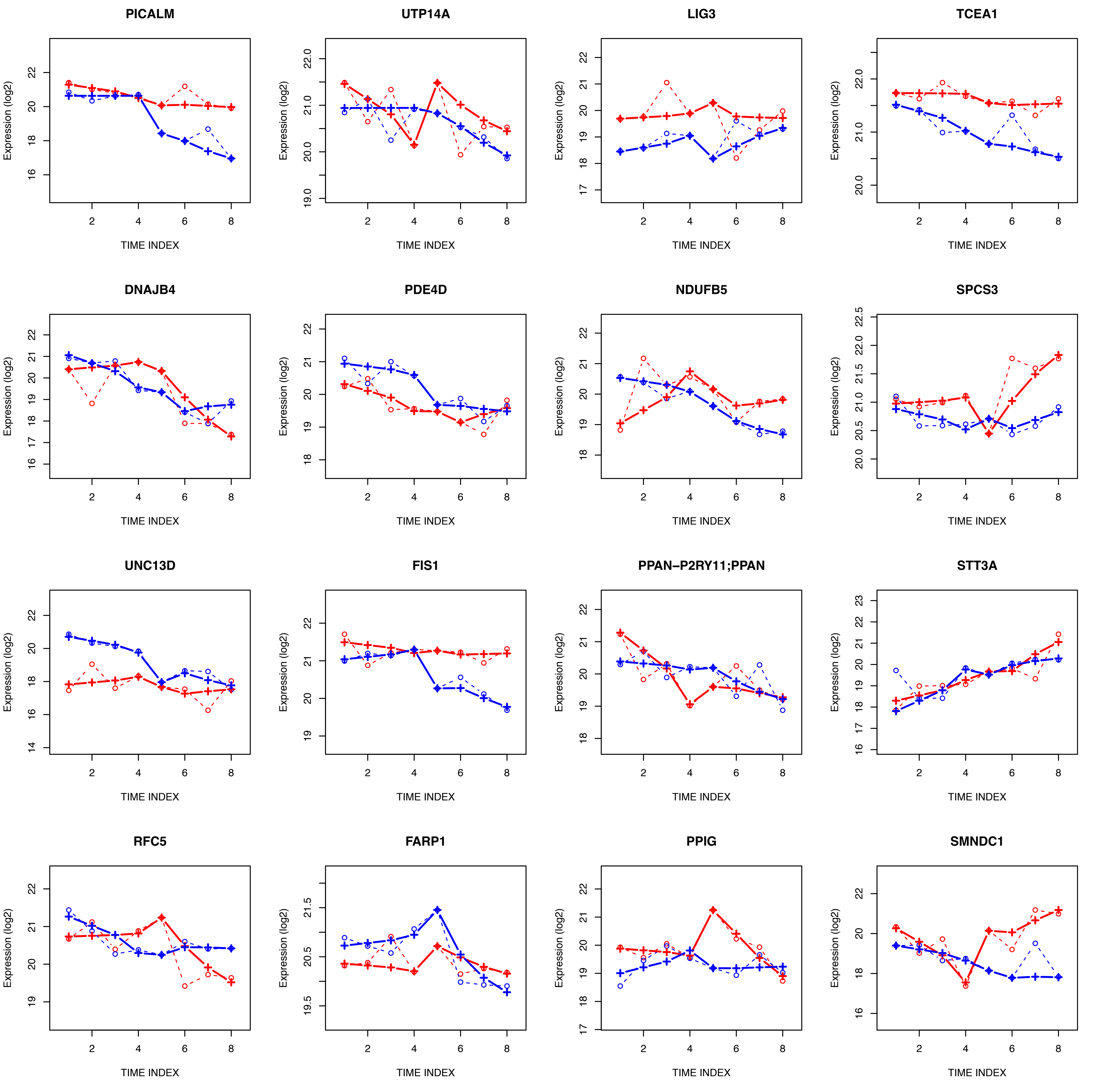
PICALM
UTP14A
LIG3
TCEA1
DNAJB4
PDE4D
NDUFB5
SPCS3
UNC13D
FIS1
PPAN–P2RY11;PPAN
STT3A
RFC5
FARP1
PPIG
SMNDC1
Expression (log2)
TIME INDEX

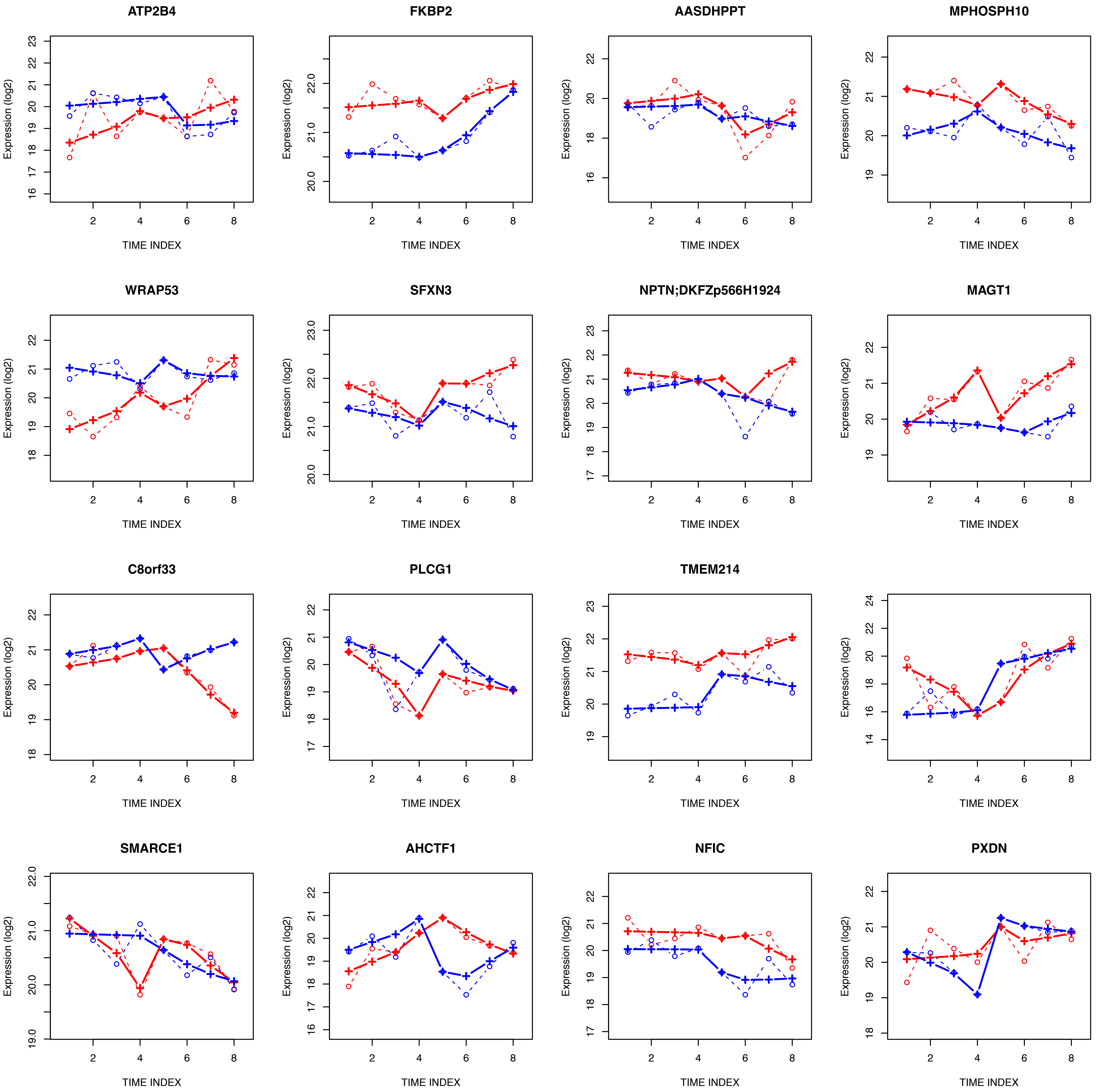
ATP2B4
FKBP2
AASDHPPT
MPHOSPH10
WRAP53
SFXN3
NPTN;DKFZp566H1924
MAGT1
C8orf33
PLCG1
TMEM214
SMARCE1
AHCTF1
NFIC
PXDN
Expression (log2)
TIME INDEX

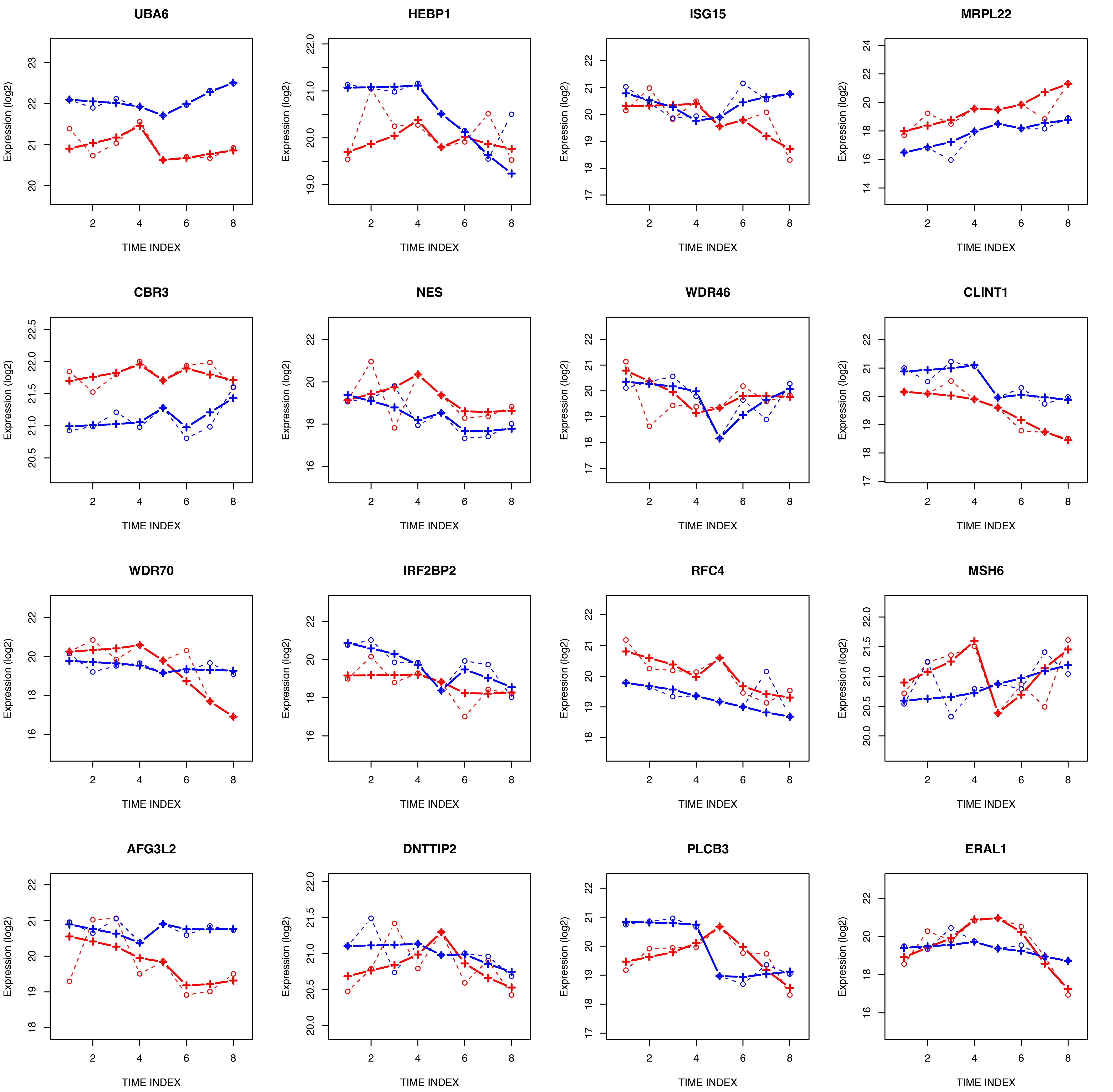
UBA6
HEBP1
ISG15
MRPL22
CBR3
NES
WDR46
CLINT1
WDR70
IRF2BP2
RFC4
MSH6
AFG3L2
DNTTIP2
PLCB3
ERAL1
Expression (log2)
TIME INDEX

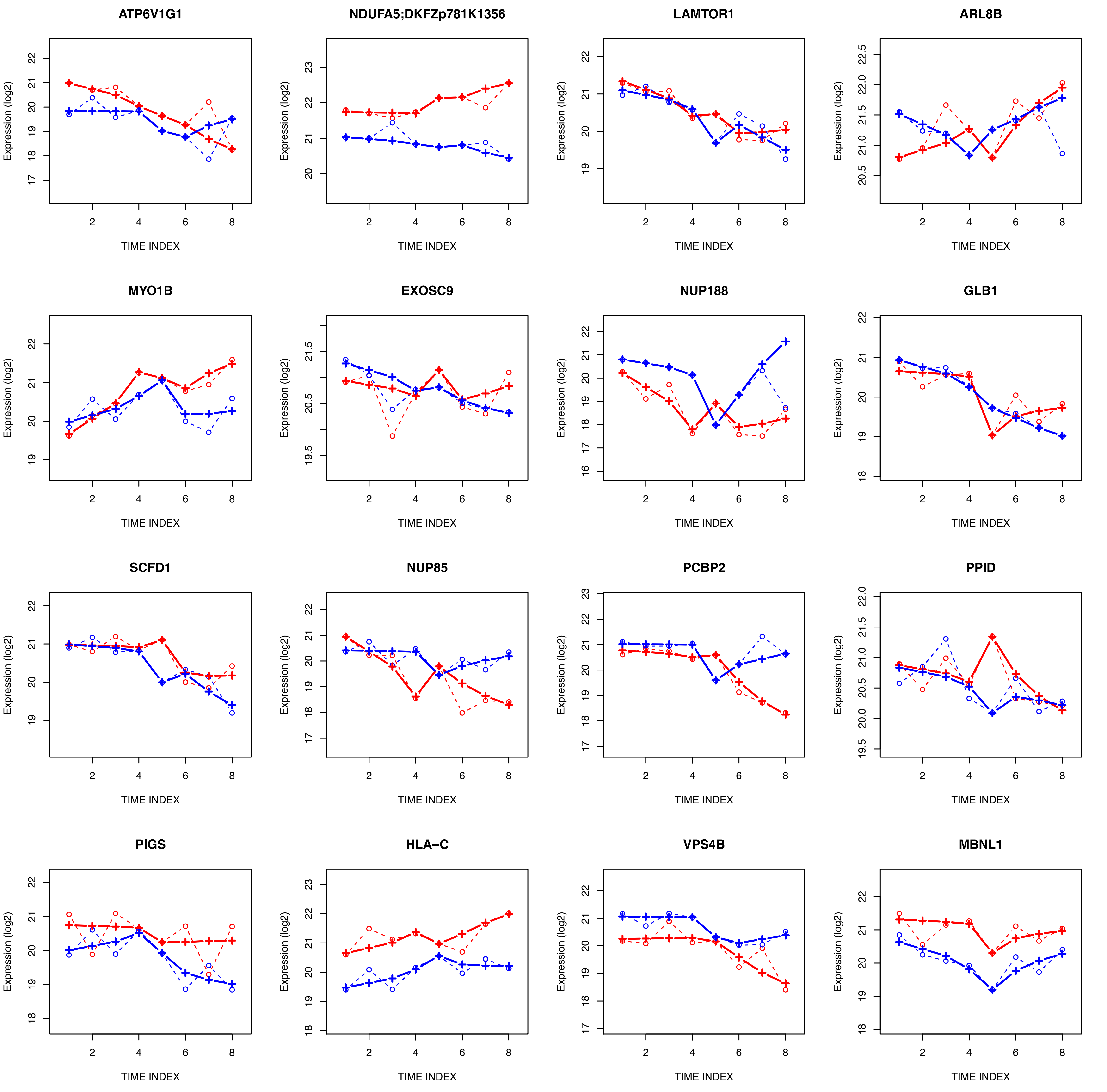

ATP6V1G1
NDUFA5;DKFZp781K1356
LAMTOR1
ARL8B
MYO1B
EXOSC9
NUP188
GLB1
SCFD1
NUP85
PCBP2
PPID
PIGS
HLA−C
VPS4B
MBNL1
Expression (log2)
TIME INDEX
2
4
6
8

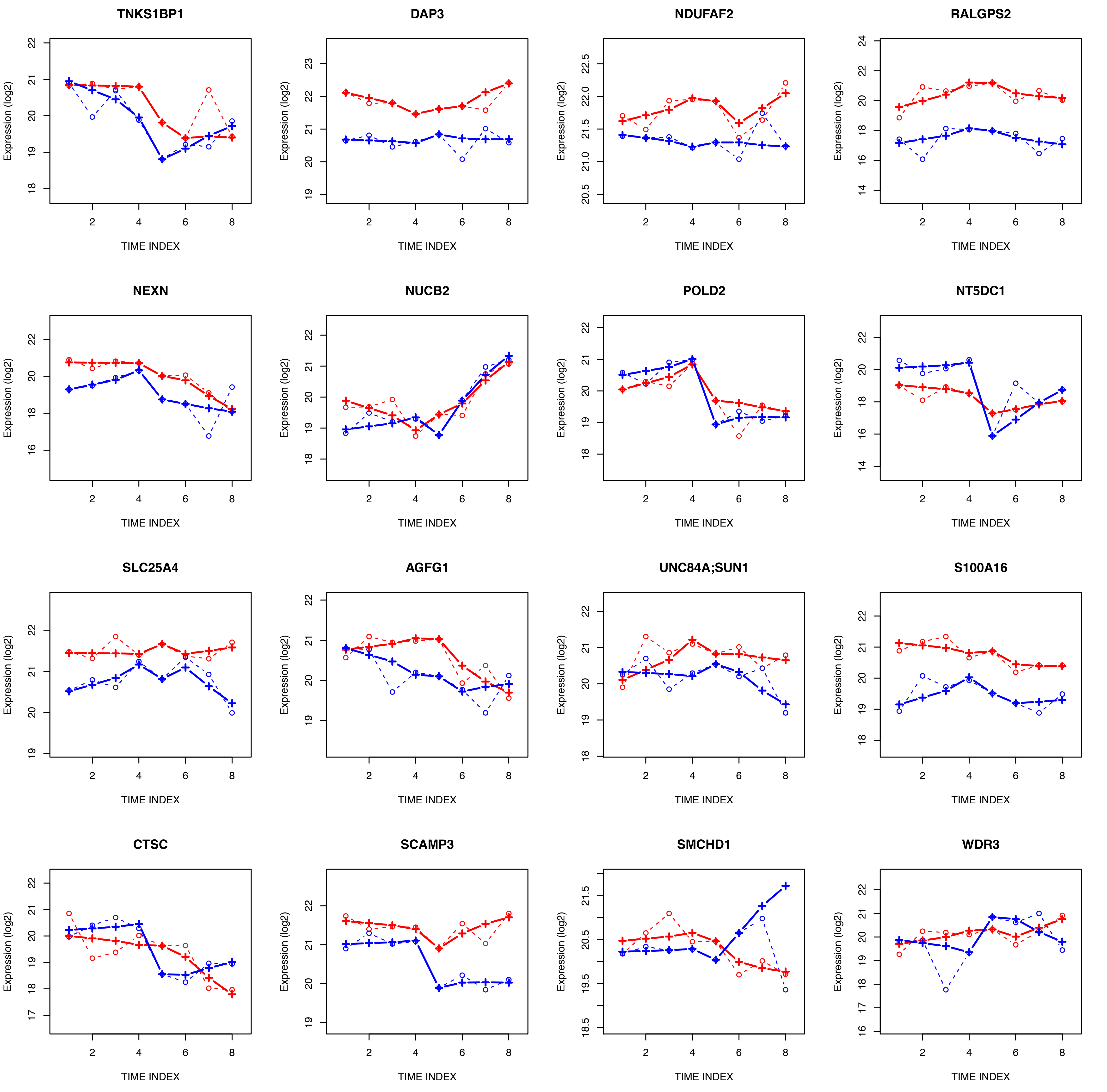
TNKS1BP1
DAP3
NDUFAF2
RALGPS2
NEXN
NUCB2
POLD2
NT5DC1
SLC25A4
AGFG1
UNC84A;SUN1
S100A16
CTSC
SCAMP3
SMCHD1
WDR3
Expression (log2)
TIME INDEX

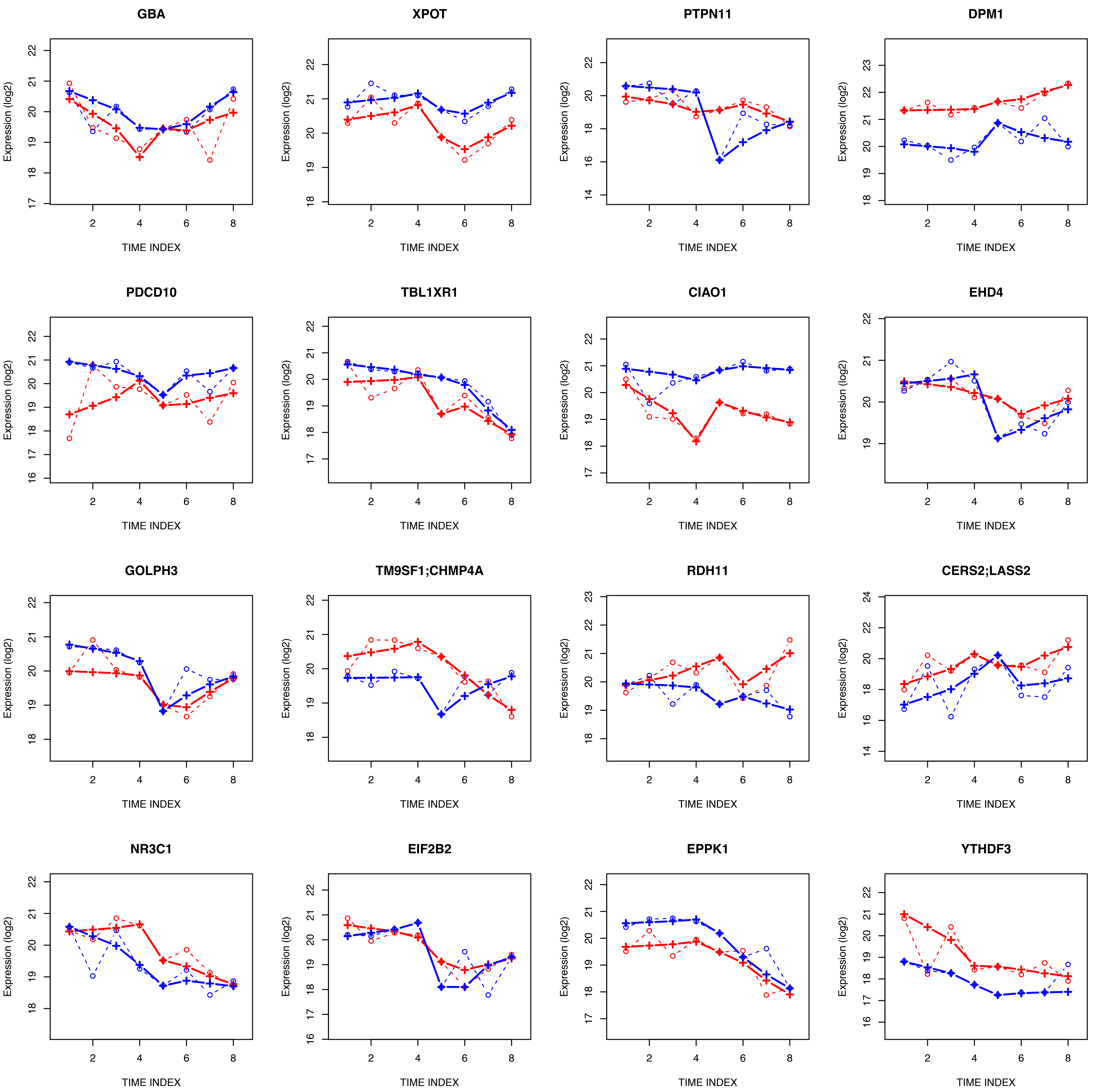
GBA
XPOT
PTPN11
DPM1
PDCD10
TBL1XR1
CIAO1
EHD4
GOLPH3
TM9SF1;CHMP4A
RDH11
CERS2;LASS2
NR3C1
EIF2B2
EPPK1
YTHDF3
Expression (log2)
TIME INDEX

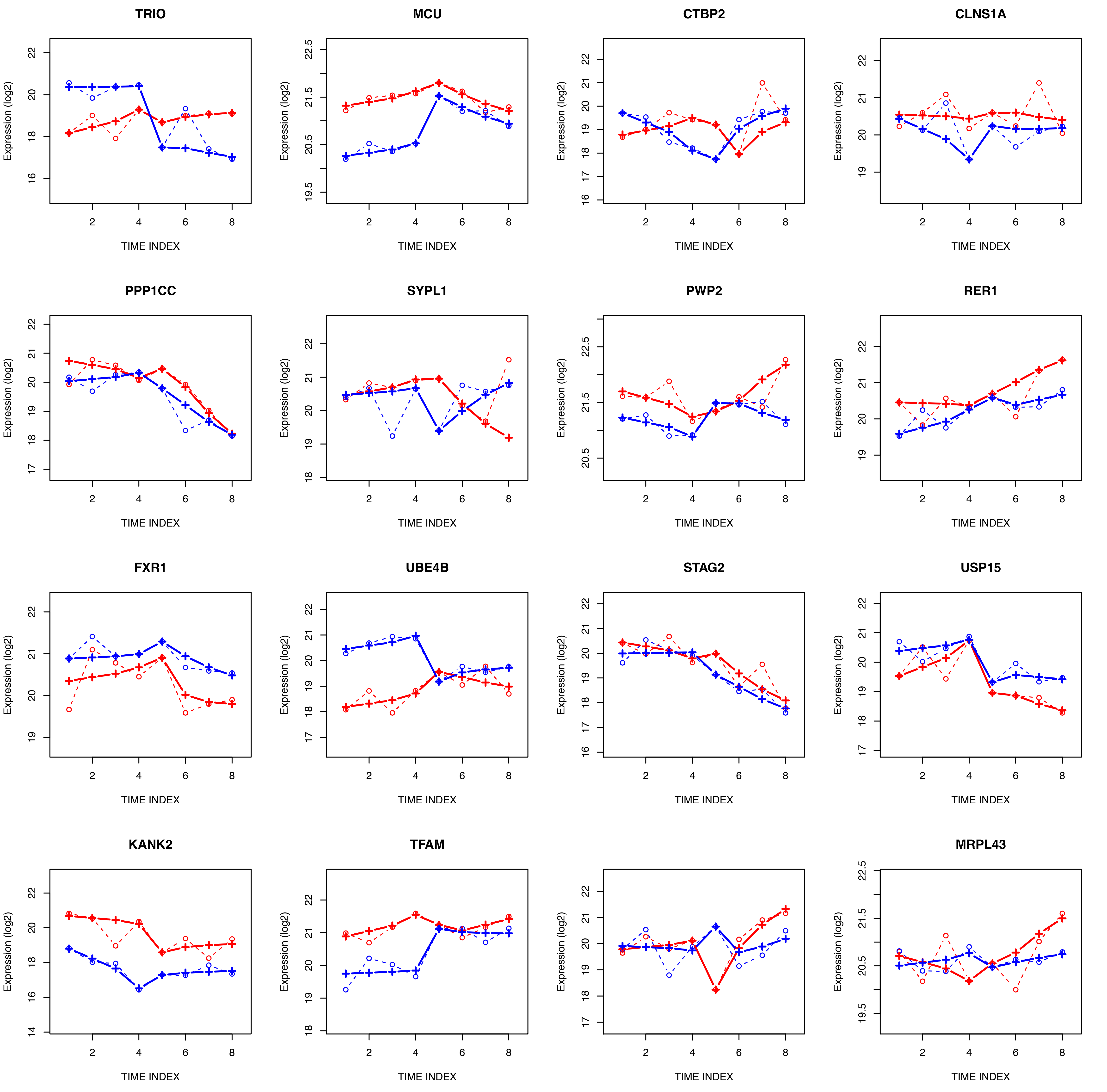

TRIO
MCU
CTBP2
CLNS1A
PPP1CC
SYPL1
PWP2
RER1
FXR1
UBE4B
STAG2
USP15
KANK2
TFAM
MRPL43
Expression (log2)
TIME INDEX

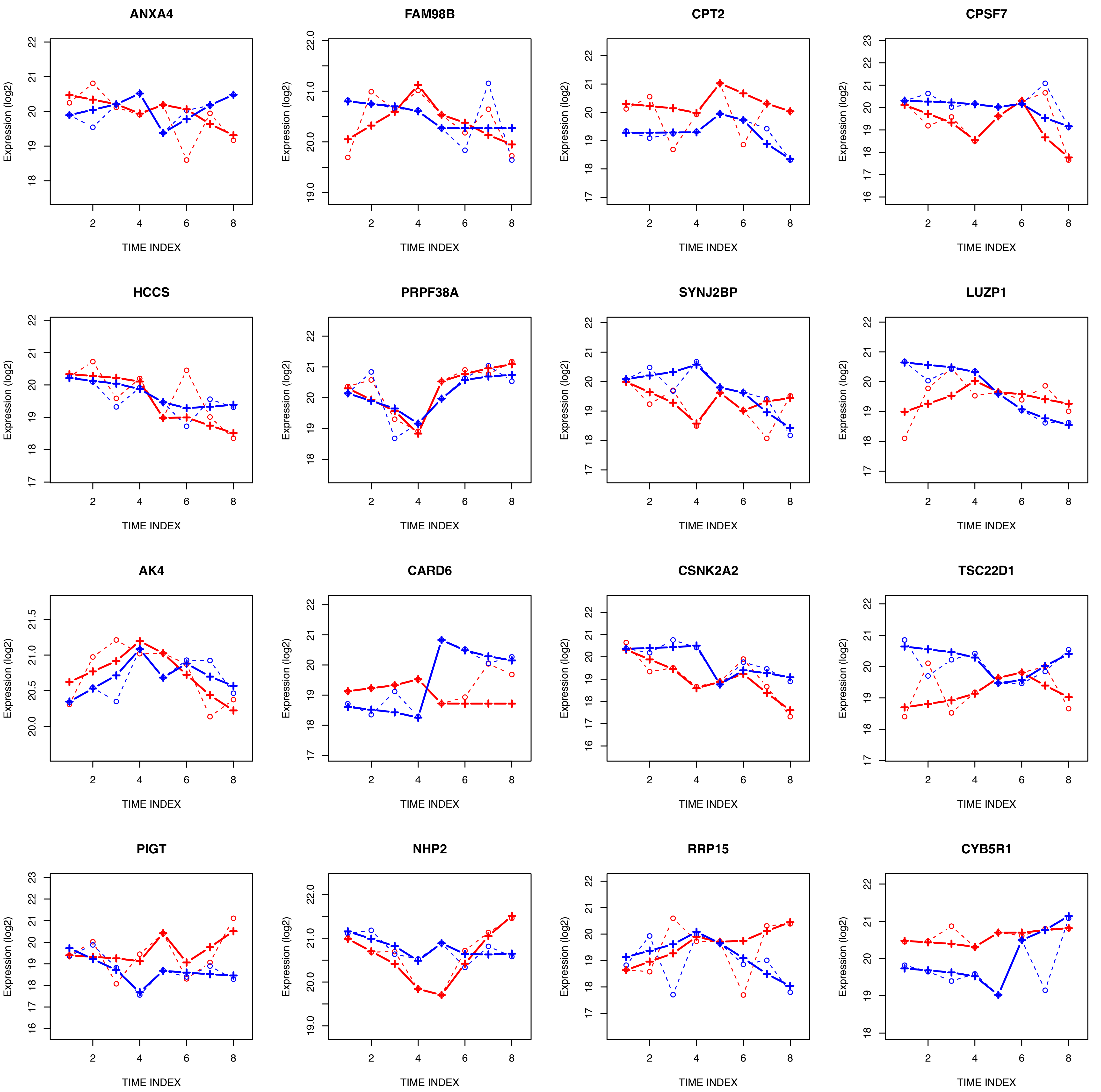
ANXA4
FAM98B
CPT2
CPSF7
HCCS
PRPF38A
SYNJ2BP
LUZP1
AK4
CARD6
CSNK2A2
TSC22D1
PIGT
NHP2
RRP15
CYB5R1
Expression (log2)
TIME INDEX

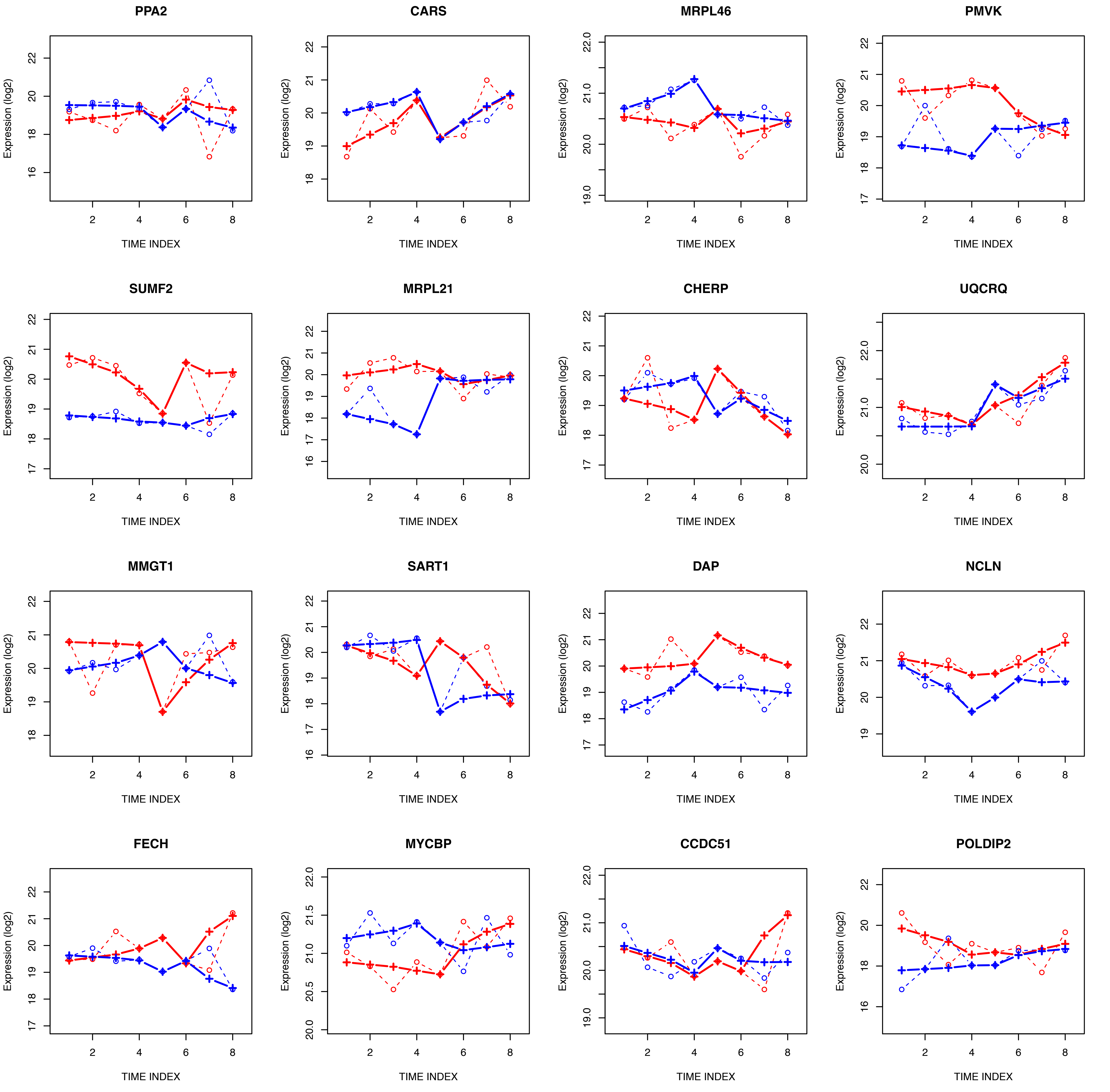
PPA2
CARS
MRPL46
PMVK
SUMF2
MRPL21
CHERP
UQCRQ
MMGT1
SART1
DAP
NCLN
FECH
MYCBP
CCDC51
POLDIP2
Expression (log2)
TIME INDEX

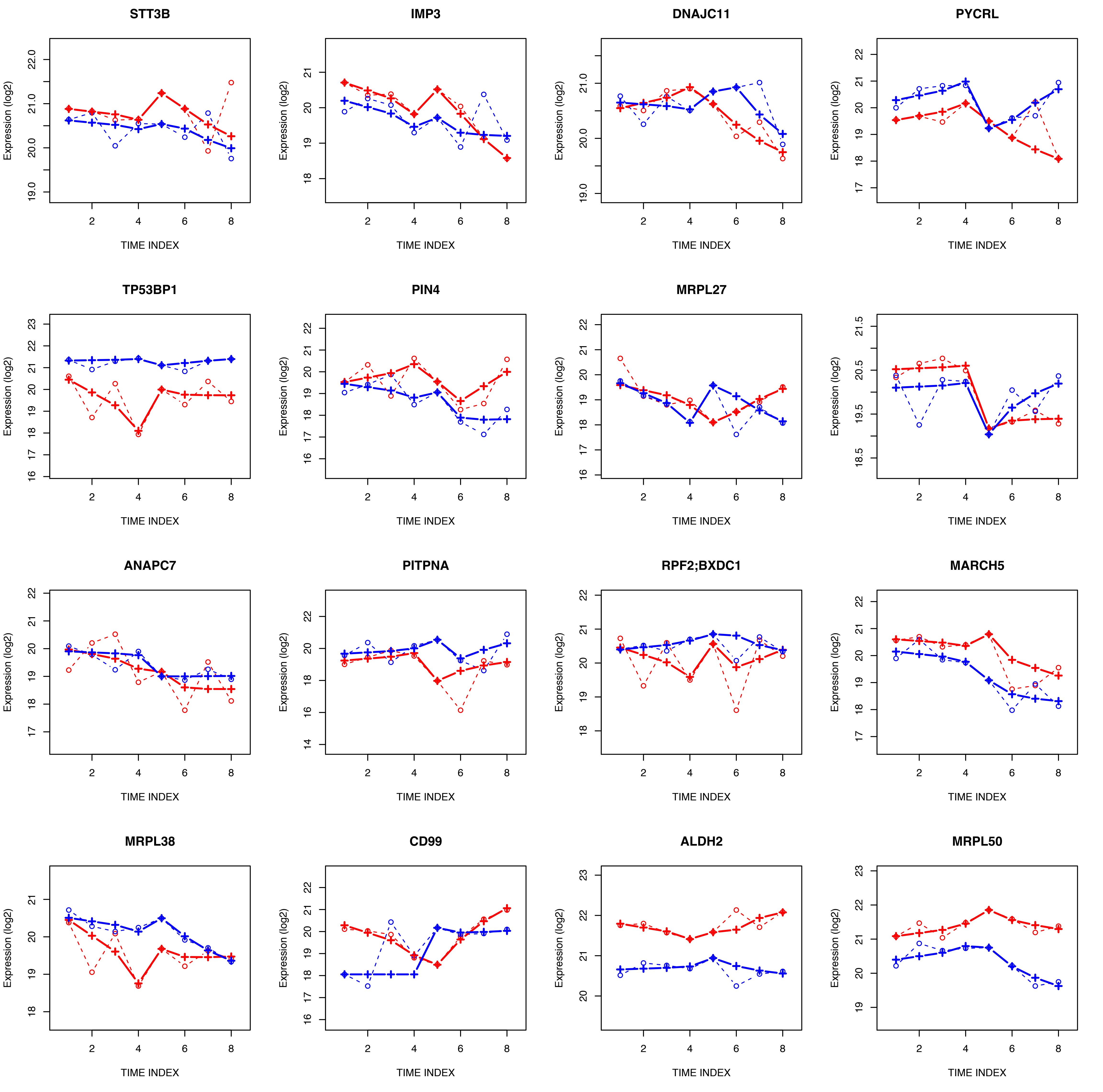
STT3B
IMP3
DNAJC11
PYCRL
TP53BP1
PIN4
MRPL27
ANAPC7
PITPNA
RPF2;BXDC1
MARCH5
MRPL38
CD99
ALDH2
MRPL50
Expression (log2)
TIME INDEX

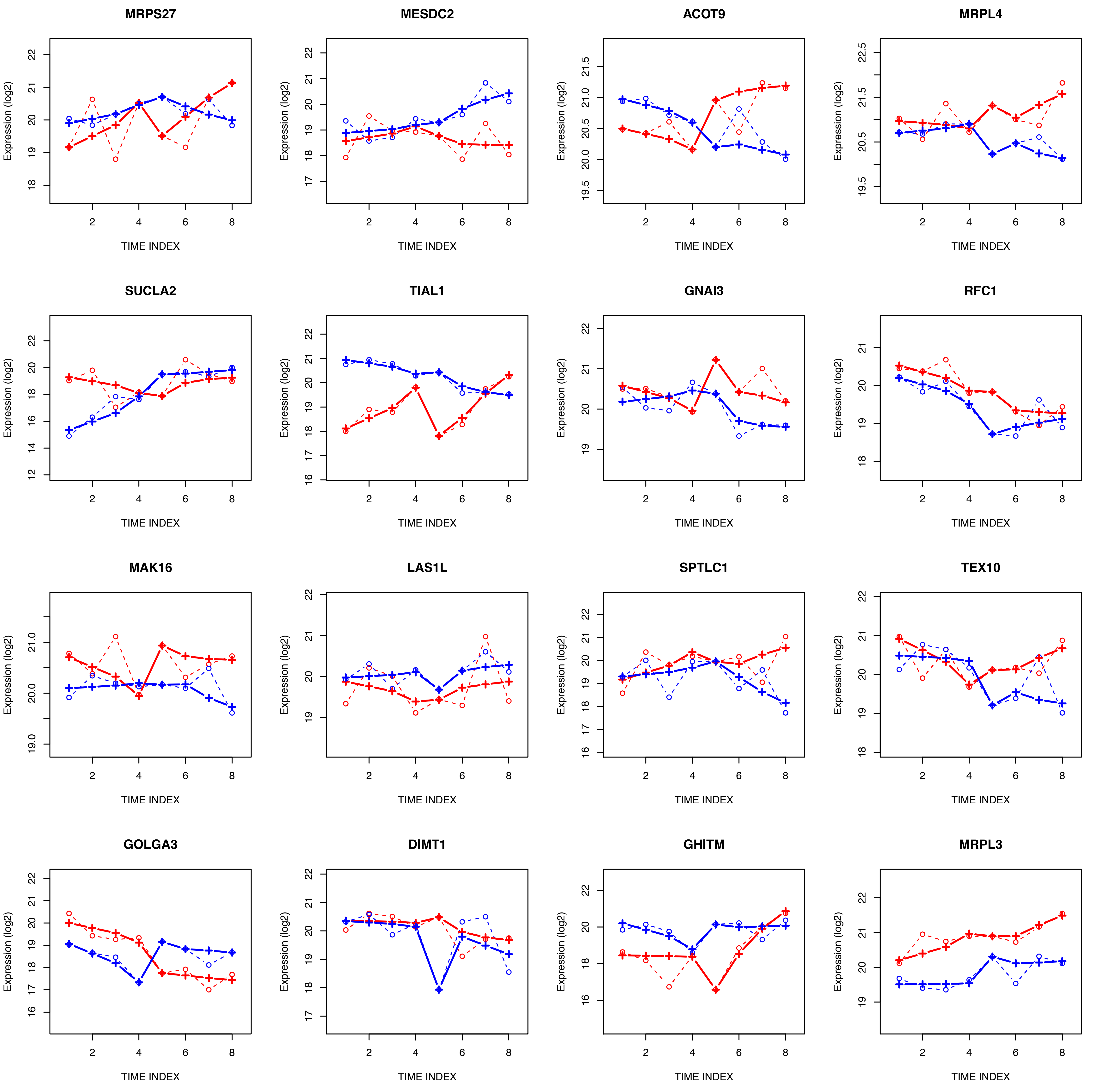
MRPS27
MESDC2
ACOT9
MRPL4
SUCLA2
TIAL1
GNAI3
RFC1
MAK16
LAS1L
SPTLC1
TEX10
GOLGA3
DIMT1
GHITM
MRPL3
Expression (log2)
TIME INDEX

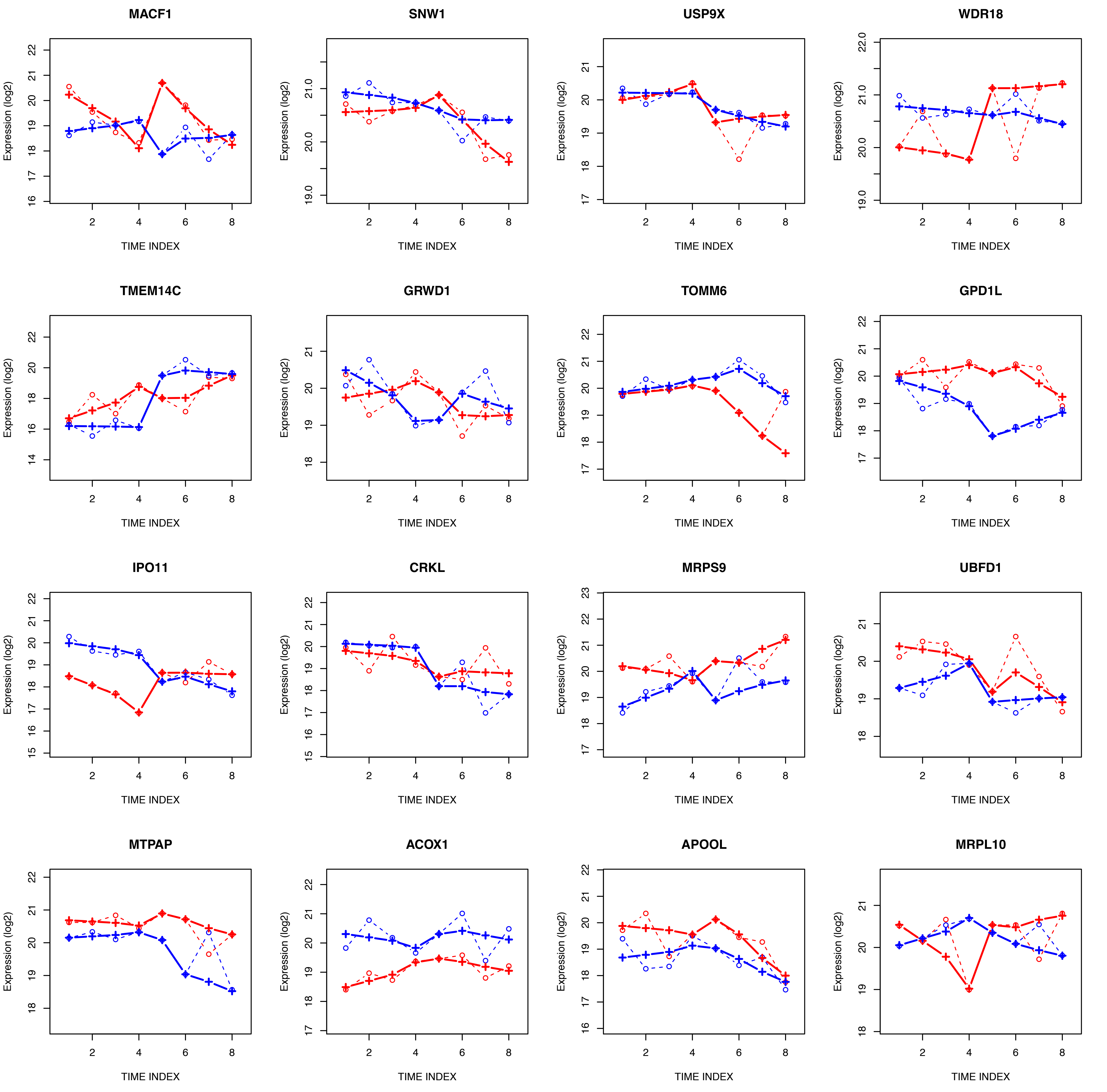
MACF1
SNW1
USP9X
WDR18
TMEM14C
GRWD1
TOMM6
GPD1L
IPO11
CRKL
MRPS9
UBFD1
MTPAP
ACOX1
APOOL
MRPL10
Expression (log2)
TIME INDEX

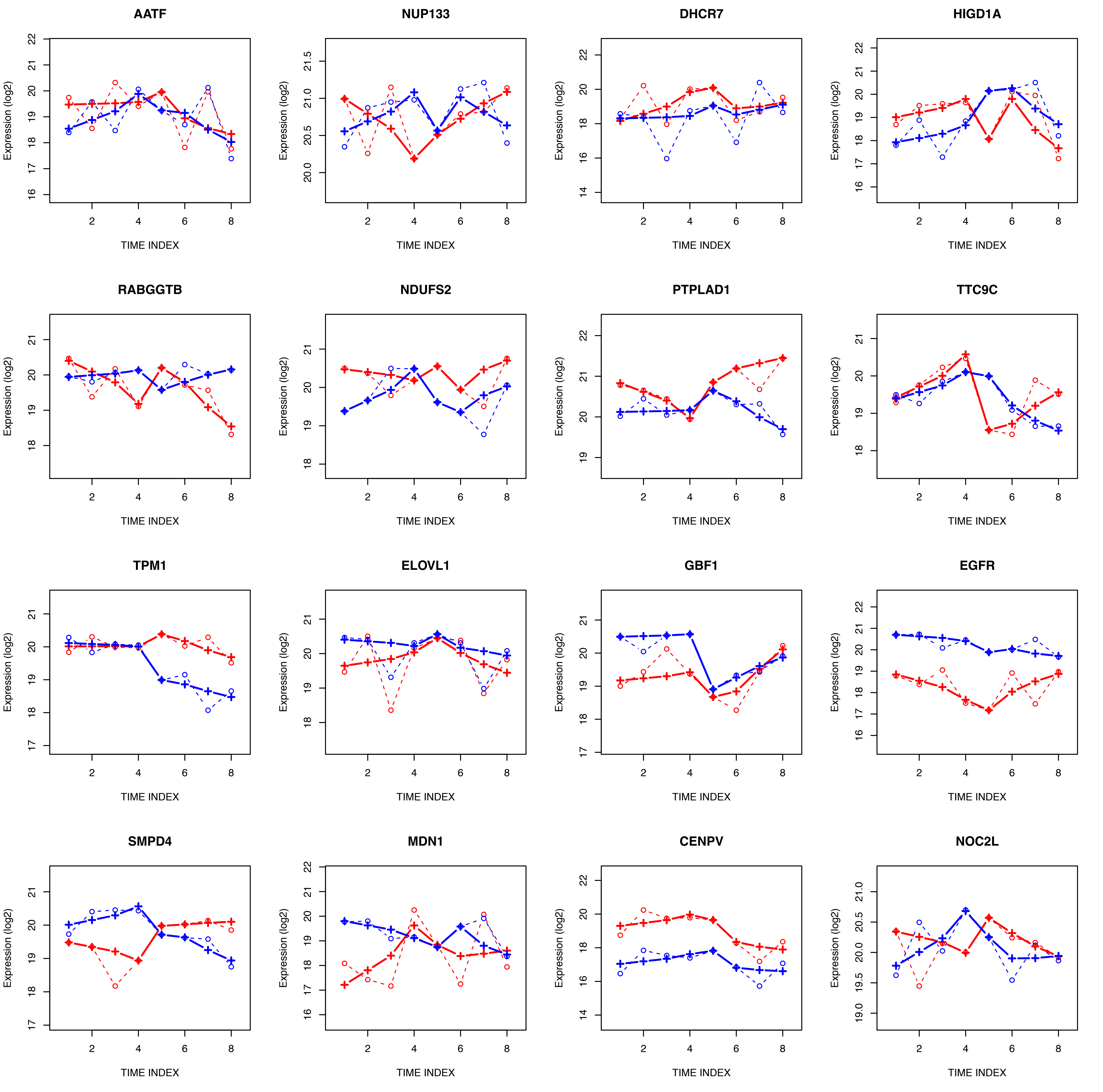
AATF
NUP133
DHCR7
HIGD1A
RABGGTB
NDUFS2
PTPLAD1
TTC9C
TPM1
ELOVL1
GBF1
EGFR
SMPD4
MDN1
CENPV
NOC2L
Expression (log2)
TIME INDEX

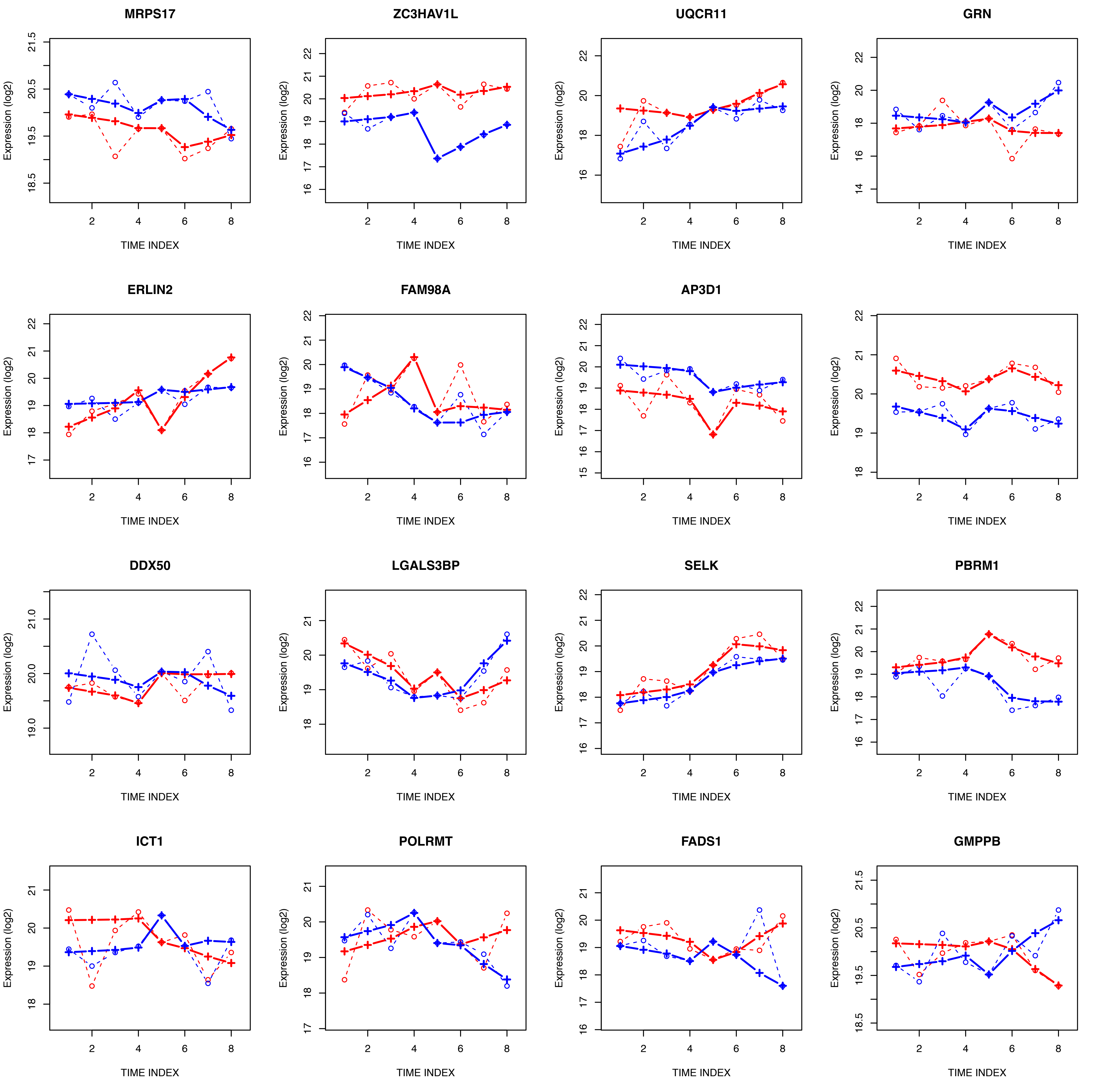
MRPS17
Expression (log2)
18.5 19.5 20.5 21.5
2 4 6 8
TIME INDEX
ZC3HAV1L
Expression (log2)
16 17 18 19 20 21 22
2 4 6 8
TIME INDEX
UQCR11
Expression (log2)
16 18 20 22
2 4 6 8
TIME INDEX
GRN
Expression (log2)
14 16 18 20 22
2 4 6 8
TIME INDEX
ERLIN2
Expression (log2)
17 18 19 20 21 22
2 4 6 8
TIME INDEX
FAM98A
Expression (log2)
16 17 18 19 20 21 22
2 4 6 8
TIME INDEX
AP3D1
Expression (log2)
15 16 17 18 19 20 21 22
2 4 6 8
TIME INDEX
Expression (log2)
18 19 20 21 22
2 4 6 8
TIME INDEX
DDX50
Expression (log2)
19.0 20.0 21.0
2 4 6 8
TIME INDEX
LGALS3BP
Expression (log2)
18 19 20 21
2 4 6 8
TIME INDEX
SELK
Expression (log2)
16 17 18 19 20 21 22
2 4 6 8
TIME INDEX
PBRM1
Expression (log2)
16 17 18 19 20 21 22
2 4 6 8
TIME INDEX
ICT1
Expression (log2)
18 19 20 21
2 4 6 8
TIME INDEX
POLRMT
Expression (log2)
17 18 19 20 21
2 4 6 8
TIME INDEX
FADS1
Expression (log2)
16 17 18 19 20 21
2 4 6 8
TIME INDEX
GMPPB
Expression (log2)
18.5 19.5 20.5 21.5
2 4 6 8
TIME INDEX

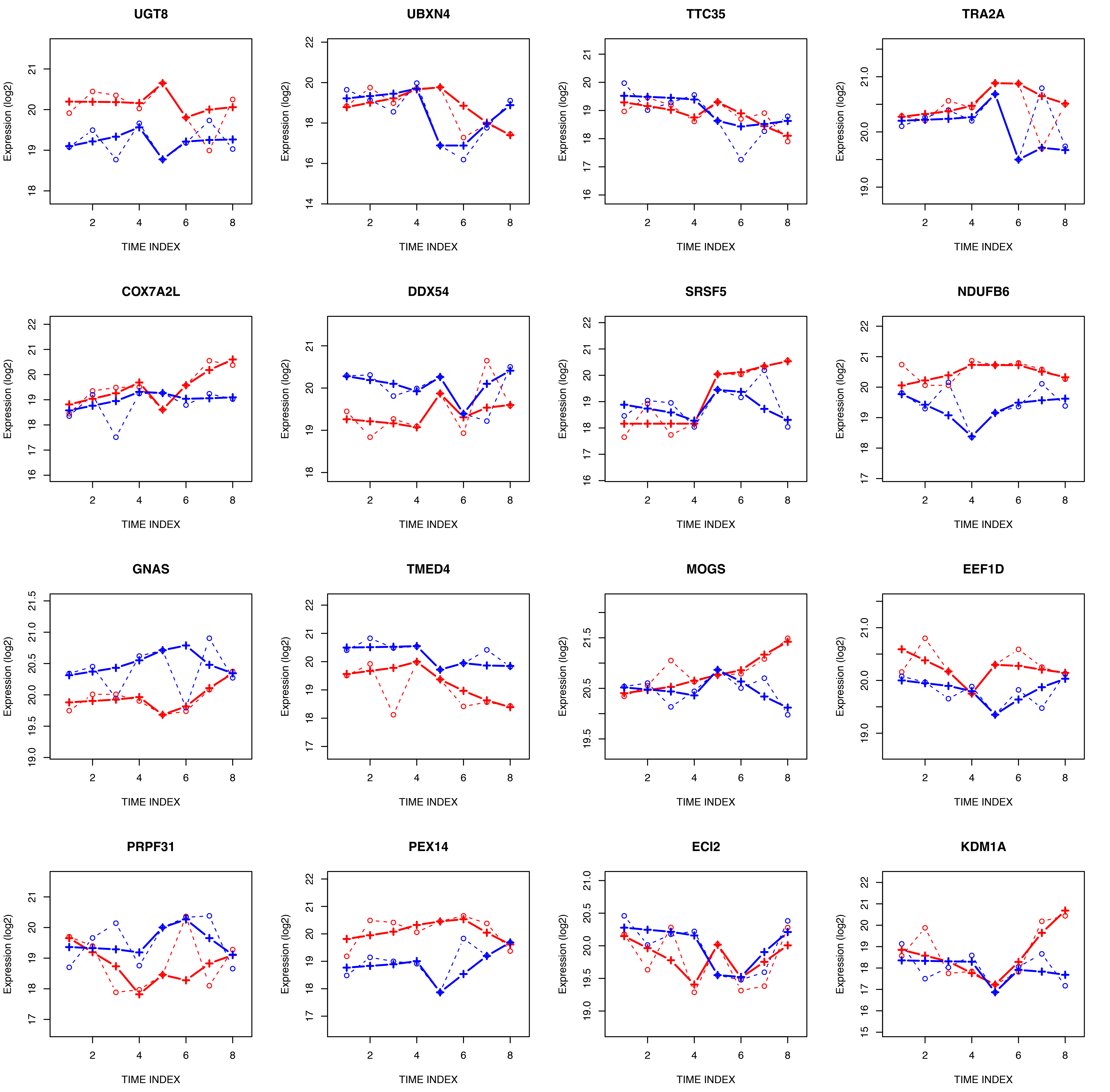
UGT8
Expression (log2)
TIME INDEX
UBXN4
Expression (log2)
TIME INDEX
TTC35
Expression (log2)
TIME INDEX
TRA2A
Expression (log2)
TIME INDEX
COX7A2L
Expression (log2)
TIME INDEX
DDX54
Expression (log2)
TIME INDEX
SRSF5
Expression (log2)
TIME INDEX
NDUFB6
Expression (log2)
TIME INDEX
GNAS
Expression (log2)
TIME INDEX
TMED4
Expression (log2)
TIME INDEX
MOGS
Expression (log2)
TIME INDEX
EEF1D
Expression (log2)
TIME INDEX
PRPF31
Expression (log2)
TIME INDEX
PEX14
Expression (log2)
TIME INDEX
ECI2
Expression (log2)
TIME INDEX
KDM1A
Expression (log2)
TIME INDEX

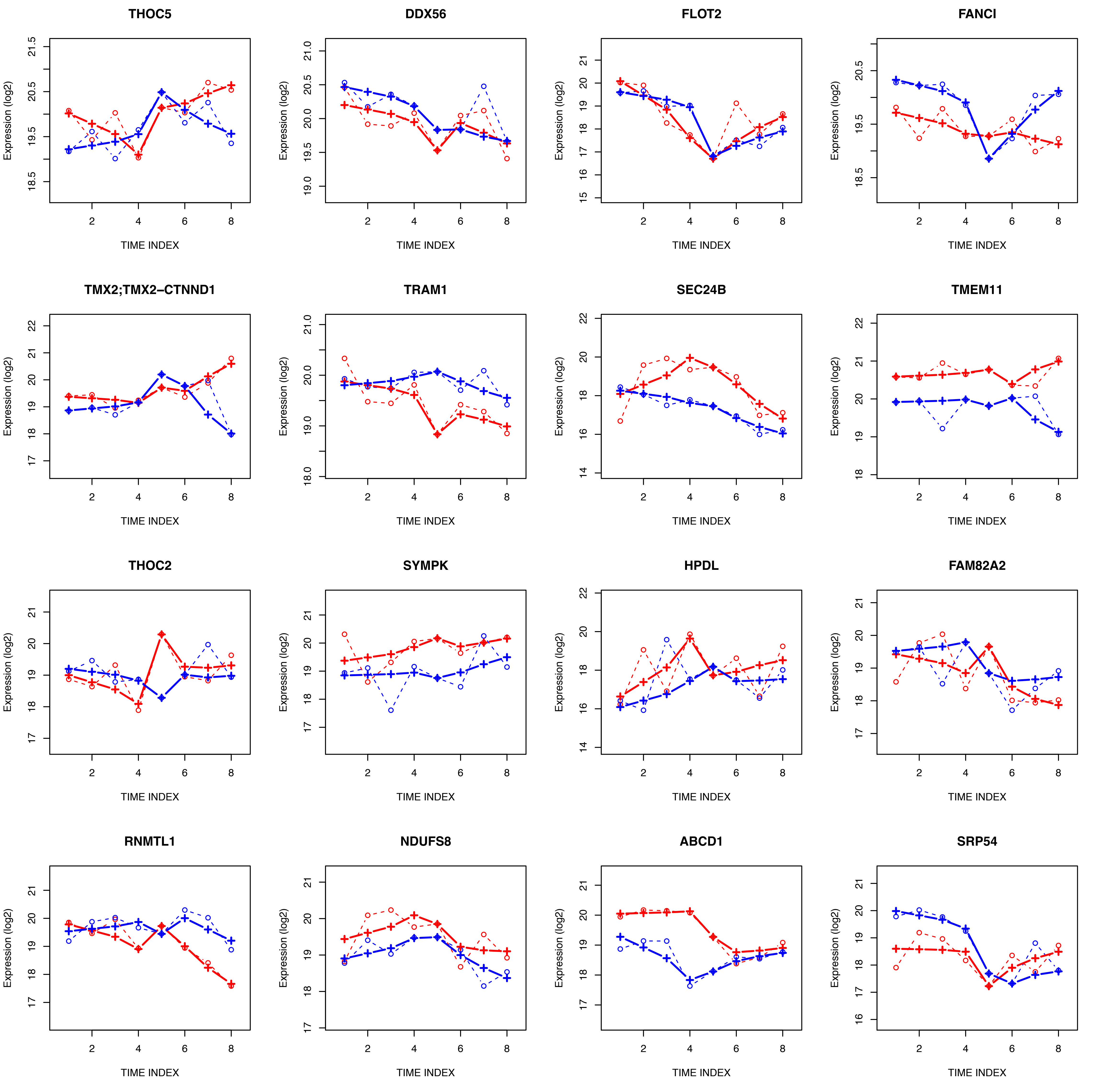
THOC5
DDX56
FLOT2
FANCI
TMX2;TMX2–CTNND1
TRAM1
SEC24B
TMEM11
THOC2
SYMPK
HPDL
FAM82A2
RNMTL1
NDUFS8
ABCD1
SRP54
Expression (log2)
TIME INDEX
2
4
6
8

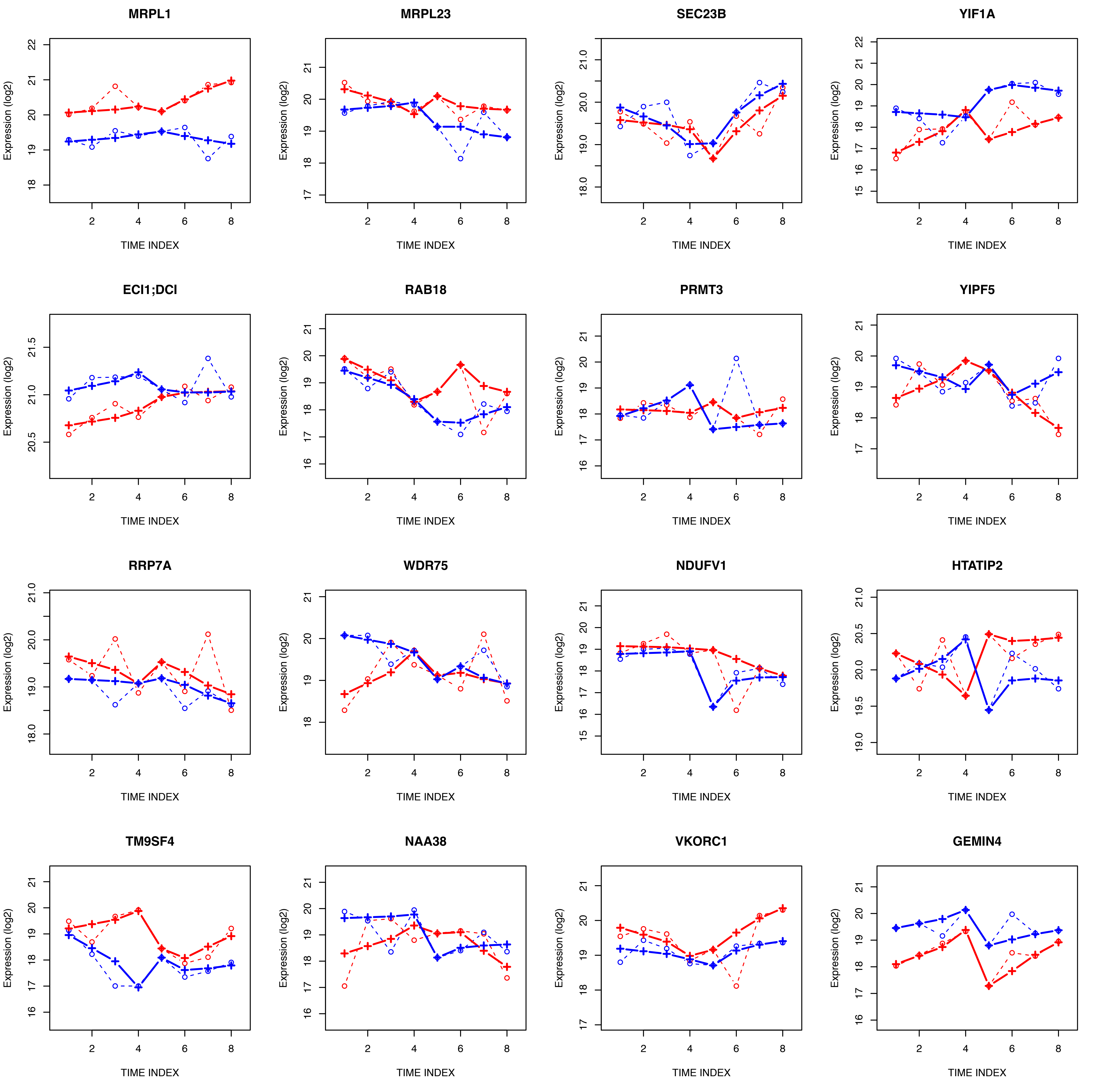

MRPL1
MRPL23
SEC23B
YIF1A
ECI1;DCI
RAB18
PRMT3
YIPF5
RRP7A
WDR75
NDUFV1
HTATIP2
TM9SF4
NAA38
VKORC1
GEMIN4
Expression (log2)
TIME INDEX

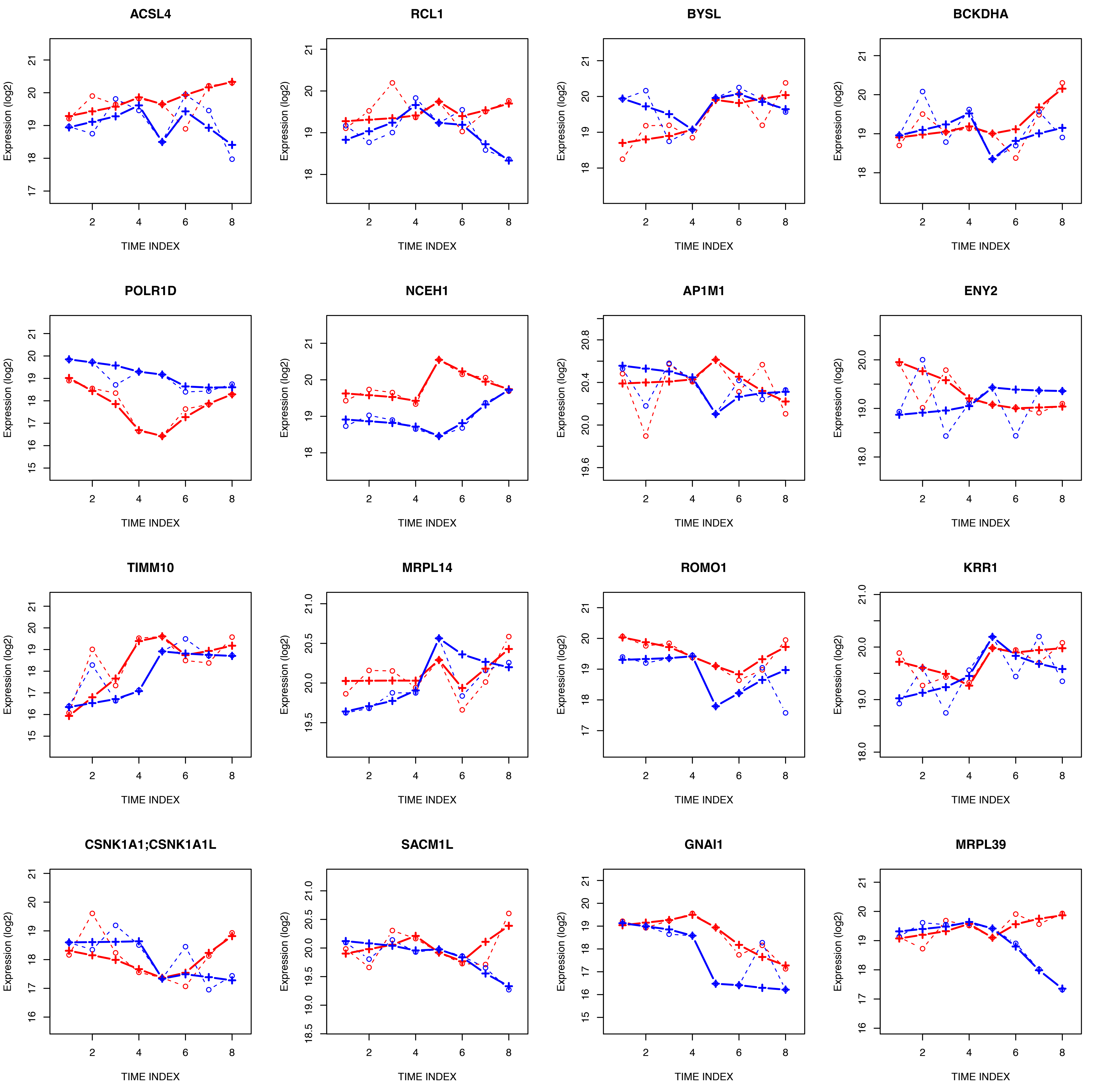
ACSL4
RCL1
BYSL
BCKDHA
POLR1D
NCEH1
AP1M1
ENY2
TIMM10
MRPL14
ROMO1
KRR1
CSNK1A1;CSNK1A1L
SACM1L
GNAI1
MRPL39
Expression (log2)
TIME INDEX

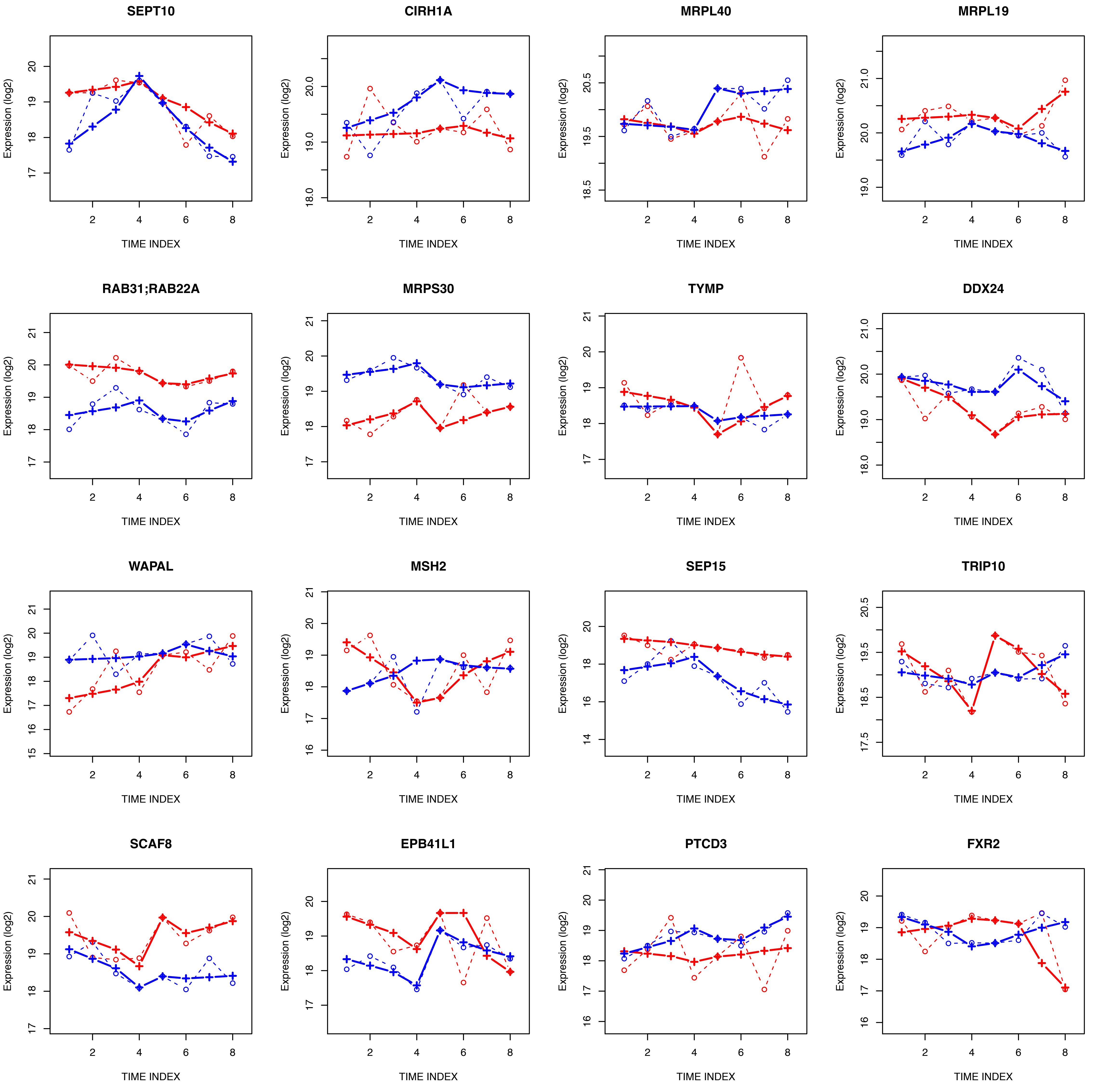
SEPT10
CIRH1A
MRPL40
MRPL19
RAB31;RAB22A
MRPS30
TYMP
DDX24
WAPAL
MSH2
SEP15
TRIP10
SCAF8
EPB41L1
PTCD3
FXR2
Expression (log2)
TIME INDEX

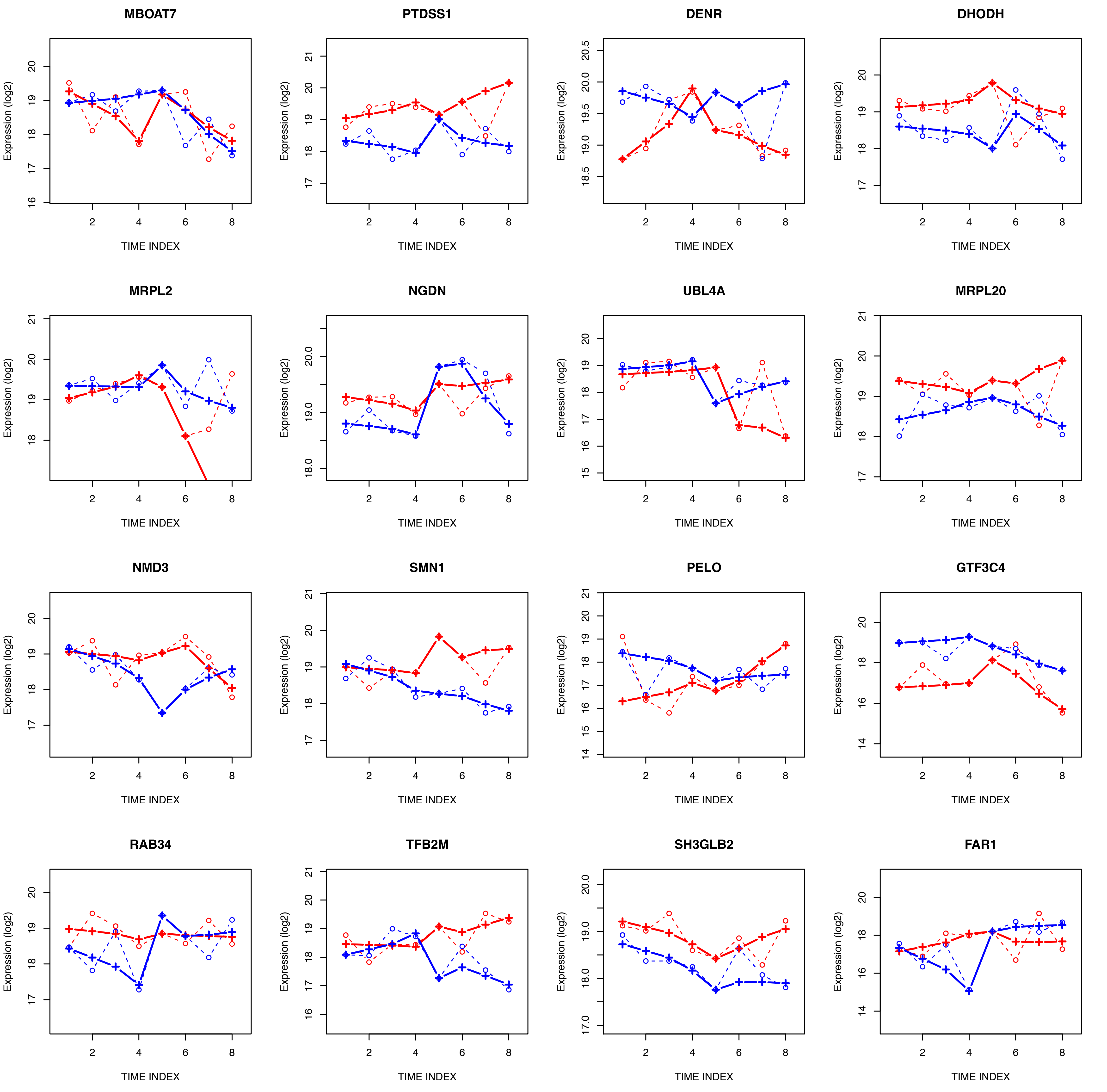
MBOAT7
PTDSS1
DENR
DHODH
MRPL2
NGDN
UBL4A
MRPL20
NMD3
SMN1
PELO
GTF3C4
RAB34
TFB2M
SH3GLB2
FAR1
Expression (log2)
TIME INDEX

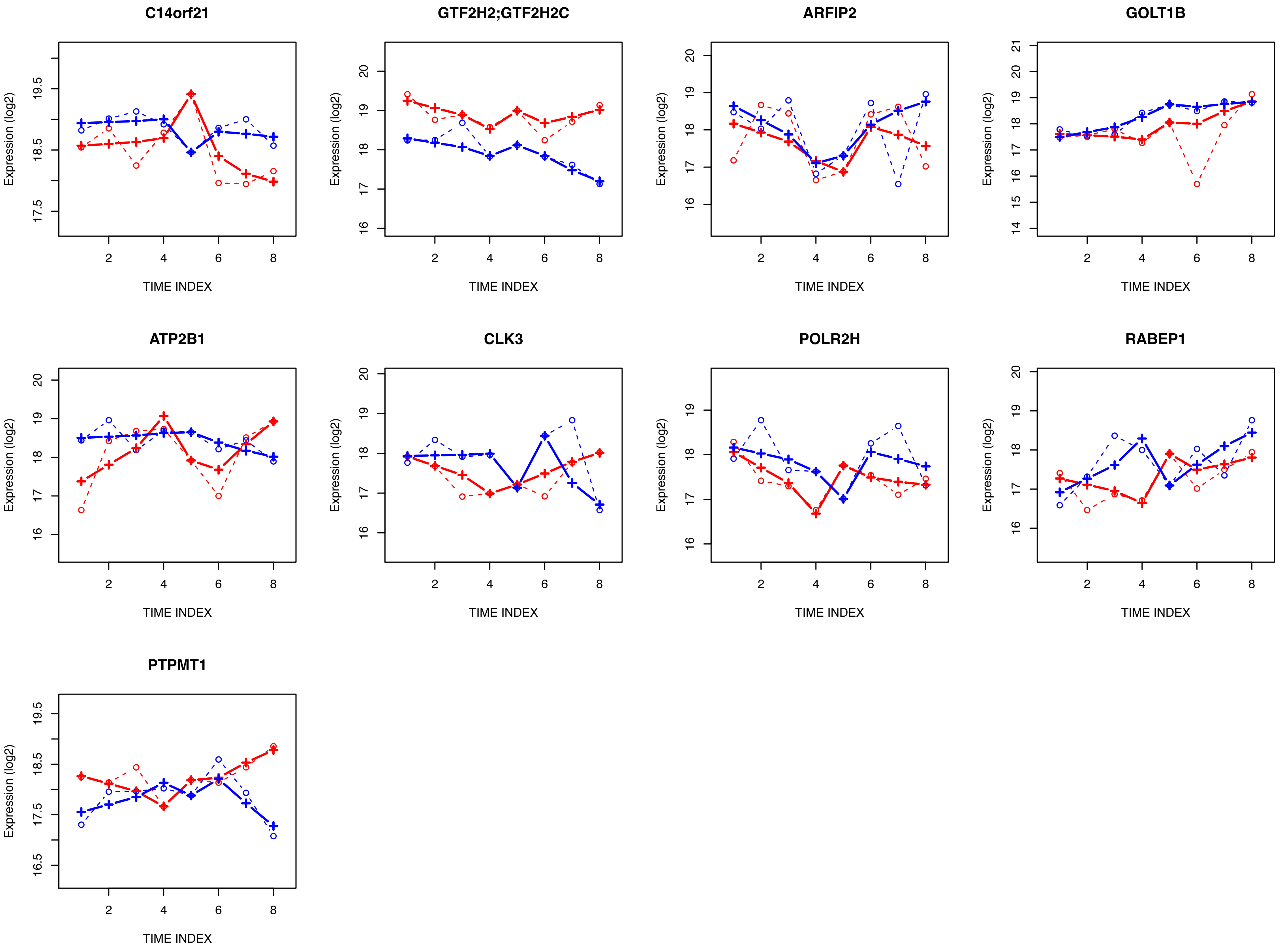

C14orf21
GTF2H2;GTF2H2C
ARFIP2
GOLT1B
ATP2B1
CLK3
POLR2H
RABEP1
PTPMT1
Expression (log2)
TIME INDEX
2
4
6
8
